# Machine learning-based prediction of glioma grading

**DOI:** 10.1371/journal.pone.0314831

**Published:** 2025-12-26

**Authors:** Shihong Liu, Yunfang Xie, Xuanli Gong, Jieyu He, Wei Zou

**Affiliations:** School of Public Health, Kunming Medical University, Kunming, Yunnan, China; Xiangya Hospital Central South University, CHINA

## Abstract

**Objective:**

Gliomas are among the most common and heterogeneous primary tumours of the central nervous system. Accurate grading is essential for treatment planning and prognosis, yet conventional histopathological approaches are limited by subjectivity and poor reproducibility. This study aimed to develop a machine learning–based prediction model that integrates clinical and molecular characteristics to improve early glioma grading, thereby enhancing diagnostic accuracy and supporting individualized treatment strategies.

**Methods:**

An efficient prediction model for low-grade gliomas (LGGs) and glioblastoma (grade IV, GBM) was developed by utilizing the clinical and molecular characteristics of gliomas from The Cancer Genome Atlas (TCGA) dataset. A novel integration of recursive feature elimination (RFE) with random forest (RF) and elastic net regression (ENR) was implemented to select features efficiently. Additionally, the synthetic minority oversampling technique (SMOTE) was applied to balance the training set, and K-nearest neighbours (KNN), support vector machine (SVM), and other algorithms were optimized through random-search hyper-parameter optimization (HPO) with five-fold cross-validation, yielding nine distinct machine learning (ML) models. Ultimately, by applying the voting and stacking algorithms, 34 ensemble learning models were constructed. Furthermore, all the models were externally validated using the Chinese Glioma Genome Atlas (CGGA) dataset. Finally, SHapley Additive exPlanations (SHAP) analysis was conducted to elucidate the prediction processes of the ensemble models.

**Results:**

Feature selection revealed 11 key grading features, including Tumour Protein 53 (TP53) and Isocitrate Dehydrogenase 1 (IDH1). Among the 9 basic models constructed by combining optimization techniques such as SMOTE, the RF model had the best performance (Area Under Curve (AUC) of 0.916 for TCGA and 0.797 for CGGA). Among the 34 integrated models constructed, the Voting25 model integrating RF, Extreme Gradient Boosting (XGBoost), and KNN achieved AUC values of 0.928 and 0.794, respectively, on the TCGA and CGGA datasets, demonstrating overall optimal predictive performance.

**Conclusion:**

Eleven key features have been identified that facilitate molecular detection and personalized targeted therapy for glioma. Nine models were developed and optimized, and the RF model was observed to provide the best performance, potentially guiding future ML-related research in glioma. Additionally, the voting ensemble method, which integrates RF, XGBoost, and KNN, was shown to achieve superior performance, thereby enhancing both accuracy and robustness. Finally, all the models were successfully validated on the CGGA dataset, indicating strong generalizability.

## Introduction

Glioma is a highly aggressive tumour associated with an extremely poor prognosis. In recent years, the global incidence of glioma has increased, and the affected population has become progressively younger, thereby posing a health challenge across multiple age groups [1–3]. In accordance with the World Health Organization (WHO) criteria, gliomas are classified as LGG or high-grade gliomas (HGGs). Among HGGs, GBM occurs in approximately 4.03 cases per 100 000 individuals and accounts for more than half of malignant central nervous system tumours [4,5]. Despite comprehensive treatment options—including surgical resection, radiotherapy, and chemotherapy—the five-year recurrence rate remains as high as 90%, and the median survival is approximately 15 months [6,7]. Tumour grading plays an essential role in guiding treatment plans and monitoring disease progression [8]. For example, patients with LGG generally receive conservative therapy or local resection, whereas those with HGG often undergo surgery followed by adjuvant radiotherapy and chemotherapy [9]. Therefore, accurate and robust early grading of glioma is paramount for predictive diagnosis. Nevertheless, traditional grading relies primarily on histopathological observation, and its inherent subjectivity and limited reproducibility prevent it from meeting the demands of precision medicine [10]. In recent years, genetic testing has been increasingly recognized as pivotal in the diagnosis of glioma. In 2016, the WHO recommended incorporating molecular phenotypes into the diagnostic framework for central nervous system tumours [5]. Studies have demonstrated that detecting tumour-derived DNA in cerebrospinal fluid can be used to effectively monitor the progression of certain gliomas, and this liquid biopsy approach provides a minimally invasive method for disease grading [11]. Concurrently, significant advancements have been made in glioma research utilizing the TCGA and CGGA datasets [12–14]. These datasets provide extensive clinical information and molecular mutational signatures pertaining to glioma. Consequently, characterizing the clinical and molecular attributes of glioma is essential for early-grade predictive diagnosis. ML technologies offer a novel avenue for constructing glioma grading models [15]. For instance, Su et al. [16] thoroughly analysed glioma characteristics by integrating clinical and molecular mutation data and applying univariate, multivariate, and ML methods. Building on this work, Guo et al. [17] combined multimodal medical imaging data with diverse ML algorithms to develop a high-precision glioma prediction model.

In recent years, the rise of big data has markedly expanded the number of variables analysed, increasing typical feature counts from dozens to hundreds or more [18,19]. This growth presents both challenges and opportunities, underscoring the need for feature selection. A larger feature set can theoretically provide richer information, potentially improving the predictive accuracy and generalizability of a model. For example, in image recognition, additional features capture finer image details and characteristics, thereby increasing recognition accuracy [20,21]. Nevertheless, excessive features also increase computational complexity and increase the risk of model overfitting [22]. Consequently, feature selection is a critical preprocessing step that retains informative variables relevant to the prediction task and discards redundant or irrelevant variables, thereby enhancing model performance and interpretability [23–27]. Data imbalance further hinders performance improvement [28,29], particularly by decreasing the predictive accuracy for minority-class samples [30,31]. To mitigate this issue, researchers have continually explored innovative techniques. Among these methods, SMOTE—proposed by Chawla et al. [32]—is the most widely used oversampling algorithm. SMOTE balances the class distribution in the training set by synthesizing new minority class samples rather than duplicating existing samples. This strategy not only reduces the overfitting associated with traditional oversampling but also increases sample diversity, thereby improving the ability of a model to identify minority instances [32]. Notably, SMOTE should be applied exclusively to the training data to ensure fair and accurate evaluation on the test set [32].

Ensemble learning (EL) is an ML approach that aggregates the predictions of multiple models, using supervised or unsupervised strategies, to produce improved outputs [33]. In their pioneering research, Hansen and Salamon [34] demonstrated that EL markedly improves model generalization by evaluating a series of neural network ensembles. In recent years, numerous studies have applied EL techniques to predictive modelling [35–37]. For instance, Hassan et al. [35] proposed EL-APMC, an EL algorithm that leverages novel magnetic resonance imaging (MRI) features for glioma grading. Vidyadharan et al. [36] combined diffusion tensor imaging (DTI)—which quantifies water diffusion in white-matter tissue—with several ML algorithms to classify low- and high-grade gliomas. Additionally, Joshi et al. [37] introduced a two-stage EL framework for glioma detection and grading. This study evaluated five biomarkers—human telomerase reverse transcriptase (hTERT), chitinase-like protein (YKL-40), interleukin-6 (IL-6), tissue inhibitor of metalloproteinases-1 (TIMP-1), and the neutrophil-to-lymphocyte ratio (NLR). Multiple EL classifiers and fusion strategies were employed to construct a computer-aided diagnostic system.

Despite considerable advances in glioma grading research, several critical gaps remain. Most previous studies have relied primarily on radiomic features from imaging data and overlooked clinical characteristics (e.g., age, gender, and symptoms) and molecular markers (e.g., IDH and EGFR). Such omission may limit the generalizability and robustness of prediction models across diverse patient populations, as imaging features alone may not fully capture patient heterogeneity, molecular variations, or cohort-specific differences in tumour biology [38]. In addition, the systematic integration of advanced feature selection, class balancing, and ensemble learning approaches has not been fully explored [39], and external validation on independent datasets has been relatively limited [38]. These limitations underscore the need for an integrative approach that leverages both clinical and molecular features to improve glioma grading accuracy. We hypothesize that a machine learning–based model incorporating clinical and molecular features, combined with optimized feature selection, class balancing, and ensemble learning, can achieve higher predictive performance and better generalizability than models relying on a single data type or conventional methods can achieve.

To address these gaps, this study aimed to develop a machine learning–based glioma grading prediction framework that integrates clinical and molecular features to improve early grading. The proposed framework is designed to increase diagnostic accuracy and facilitate individualized treatment strategies.

A high-performance glioma grading prediction model was developed using clinical and molecular data from the TCGA and was externally validated with the CGGA. The principal contributions of this study are summarized as follows:

- Recursive feature elimination (RFE) was integrated with random forest (RF) and elastic net regression (ENR) to select features efficiently, reduce redundancy and highlight variables with superior predictive value.
- SMOTE was applied to balance the training set by synthesizing additional minority class samples instead of duplicating existing observations.
- Random-search hyper-parameter optimization (HPO) was performed with five-fold cross-validation, improving parameter tuning and performance estimation.
- Voting and stacking ensemble strategies were employed to aggregate multiple base learners and decrease the error associated with any single model.
- External validation was performed on the CGGA to confirm the generalizability of the model across independent datasets and patient populations.

The remainder of this paper is structured as follows: The Materials and Methods section describes the data sources, feature selection procedures, and model construction processes, including hyperparameter optimization, SMOTE balancing, and ensemble strategies. The Results section presents the outcomes of feature selection and the performance of both the base models and the ensemble methods, together with the results of the calibration, decision curve, and SHAP analyses. The Discussion section interprets these findings in the context of the literature, highlights their biological and clinical implications, and addresses the limitations of this study. Finally, the Conclusion section summarizes the main contributions and potential applications of this study.

## Materials and methods

### Data collection and overview

The publicly available TCGA glioma dataset was used for model training and testing, whereas the CGGA dataset served as an external validation set. Independence of the CGGA validation was ensured by isolating the data, sourcing independent cohorts, and applying unified clinical inclusion criteria. Both datasets excluded low-quality cases and entries with missing key information; their cohorts were nonoverlapping across population, region, and temporal dimensions. The TCGA dataset contains 839 instances with three clinical variables and 20 high-frequency molecular mutation variables, with no missing or duplicate entries. The CGGA dataset includes 195 instances but lacks the clinical variable “race.” The remaining variables match across datasets, and no additional missing or duplicate entries are observed. Although race is not explicitly recorded in the CGGA, all the cases originated from a Chinese cohort. Consequently, the “race” variable in the CGGA was set to Asian for every case, and its feature dimension was aligned with that of the TCGA. The qualitative variables included two clinical factors—gender and race—and 20 molecular mutation markers (e.g., IDH1 and TP53). The sole quantitative variable was age, and mutation status was categorized as wild-type or mutant. The outcome label comprised two classes: LGG and GBM. An overview of the data is presented in Table 1.

**Table 1:** An overview of the data.

Characteristic variable	Total (n = 839)	LGG(n = 487)	GBM(n = 352)	*P*-Value
**Age, Median ± IQR**	51.55 ± 24.75	41.61 ± 20.22	61.40 ± 17.33	<0.001
**Gender,n(%)**				
Male	488(58.16%)	271(55.65%)	217(61.65%)	0.082
Famale	351(41.84%)	216(44.35%)	135(38.35%)	
**Race,n(%)**				
White	765(91.18%)	457(93.84%)	308(87.50%)	0.002
Black or African American	59(7.03%)	21(4.31%)	38(10.80%)	
Asian	14(1.67%)	8(1.64%)	6(1.70%)	
American Indian or Alaska Naive	1(0.12%)	1(0.21%)	0(0.00%)	
**Molecular/Mutation factors,n(%)**				
IDH1 Wild	435(51.85%)	106(21.77%)	329(93.47%)	<0.001
IDH1 Mutant	404(48.15%)	381(78.23%)	23(6.53%)	
TP53 Wild	491(58.52%)	252(51.75%)	239(67.90%)	<0.001
TP53 Mutant	348(41.48%)	235(48.25%)	113(32.10%)	
ATRX Wild	622(74.14%)	304(62.42%)	318(90.34%)	<0.001
ATRX Mutant	217(25.86%)	183(37.58%)	34(9.66%)	
PTEN Wild	698(83.19%)	462(94.87%)	236(67.05%)	<0.001
PTEN Mutant	141(16.81%)	25(5.13%)	116(32.95%)	
EGFR Wild	727(86.65%)	456(93.63%)	271(76.99%)	<0.001
EGFR Mutant	112(13.35%)	31(6.37%)	81(23.01%)	
CIC Wild	728(86.77%)	380(78.03%)	348(98.86%)	<0.001
CIC Mutant	111(13.23%)	107(21.97%)	4(1.14%)	
MUC16 Wild	741(88.32%)	446(91.58%)	295(83.81%)	<0.001
MUC16 Mutant	98(11.68%)	41(8.42%)	57(16.19%)	
PIK3CA Wild	766(91.30%)	448(91.99%)	318(90.34%)	0.402
PIK3CA Mutant	73(8.70%)	39(8.01%)	34(9.66%)	
NF1 Wild	772(92.01%)	458(94.05%)	314(89.20%)	0.011
NF1 Mutant	67(7.99%)	29(5.95%)	38(10.80%)	
PIK3R1 Wild	785(93.56%)	466(95.69%)	319(90.63%)	0.003
PIK3R1 Mutant	54(6.44%)	21(4.31%)	33(9.38%)	
FUBP1 Wild	794(94.64%)	444(91.17%)	350(99.43%)	<0.001
FUBP1 Mutant	45(5.36%)	43(8.83%)	2(0.57%)	
RB1 Wild	799(95.23%)	481(98.77%)	318(90.34%)	<0.001
RB1 Mutant	40(4.77%)	6(1.23%)	34(9.66%)	
NOTCH1 Wild	801(95.47%)	449(92.20%)	352(100.00%)	<0.001
NOTCH1 Mutant	38(4.53%)	38(7.80%)	0(0.00%)	
BCOR Wild	810(96.54%)	470(96.51%)	340(96.59%)	0.949
BCOR Mutant	29(3.46%)	17(3.49%)	12(3.41%)	
CSMD3 Wild	812(96.78%)	475(97.54%)	337(95.74%)	0.145
CSMD3 Mutant	27(3.22%)	12(2.46%)	15(4.26%)	
SMARCA4 Wild	812(96.78%)	464(95.28%)	348(98.86%)	0.004
SMARCA4 Mutant	27(3.22%)	23(4.72%)	4(1.14%)	
GRIN2A Wild	812(96.78%)	480(98.56%)	332(94.32%)	<0.001
GRIN2A Mutant	27(3.22%)	7(1.44%)	20(5.68%)	
IDH2 Wild	816(97.26%)	466(95.69%)	350(99.43%)	0.001
IDH2 Mutant	23(2.74%)	21(4.31%)	2(0.57%)	
FAT4 Wild	816(97.26%)	476(97.74%)	340(96.59%)	0.314
FAT4 Mutant	23(2.74%)	11(2.26%)	12(3.41%)	
PDGFRA Wild	817(97.38%)	481(98.77%)	336(95.45%)	0.003
PDGFRA Mutant	22(2.62%)	6(1.23%)	16(4.55%)	

This study relies on data extracted from two publicly accessible repositories: The Cancer Genome Atlas (TCGA) and the Chinese Glioma Genome Atlas (CGGA). Both repositories provide fully anonymized data that comply with established ethical guidelines for data sharing. Prior to data extraction and analysis, our research team thoroughly reviewed and formally agreed on the data usage policies of both repositories, ensuring full compliance with all the terms governing data access, processing, and reporting. The datasets are available at https://www.cancer.gov/tcga (TCGA) and https://www.cgga.org.cn (CGGA). TCGA Ethics and Policies provides relevant ethical explanations regarding the data in the TCGA database (https://www.cancer.gov/ccg/research/genome-sequencing/tcga/history/ethics-policies). The CGGA database also complied with relevant ethical regulations during its establishment [13]. The detailed, preprocessed clinical and mutation data for the TCGA and CGGA cohorts used in this analysis are provided in S1–S4 Tables.

### Feature selection

#### Description of the feature selection model.

RFE was applied in combination with RF and ENR to filter features.

RF involves the construction of multiple decision trees using the bagging technique. For each node in the trees, a subset of features is randomly selected to compute and accumulate the reduction in Gini impurity. The average reduction in Gini impurity for each feature across all the trees is then assigned as its importance score. The formula of the RF model can generally be expressed as follows:


$$VIM_{j}^{\left(Gini\right)}=\frac{\text{1}}{n}\sum_{i=\text{1}}^{n}VIM_{ij}^{\left(Gini\right)}$$ (1)

In the formula, $VIM_{j}^{\left(Gini\right)}\;$ denotes the Gini-based feature importance of the  j-th feature in the random forest model; n  represents the total number of decision trees in the random forest; $VIM_{ij}^{\left(Gini\right)}\;$ represents the Gini-based importance of the  j-th feature within the i-th decision tree, which quantifies the reduction in Gini impurity achieved by the  j-th feature during node splitting processes in that specific tree.

ENR incorporates both L1 and L2 regularization techniques. L1 regularization introduces sparsity, allowing for the compression of feature regression coefficients and facilitating feature selection; features with zero coefficients are deemed unimportant. Conversely, L2 regularization enhances the ability of the model to manage feature collinearity by imposing a penalty on the sum of the squares of the regression coefficients, thereby decreasing their overall magnitude. The formula of the ENR model can generally be expressed as follows:


$$Minimize\frac{\text{1}}{\text{2}N}\sum_{i=\text{1}}^{N}(y_{i}-{𝐗}_{i}\beta {)}^{\text{2}}+\lambda_{\text{1}}\left\|\beta {\|}_{\text{1}}+\lambda_{\text{2}}\right\|\beta {\|}_{\text{2}}^{\text{2}}$$ (2)

In the formula, $y_{i}\;$ represents the target value. ${𝐗}_{i}\;$ denotes the feature vector. β represents the regression coefficient. $\|\beta {\|}_{\text{1}\;}$ is the L1 regularization term (Lasso). $\|\beta {\|}_{\text{2}}^{\text{2}}\;$ is the L2 regularization term (Ridge). ${\;\lambda }_{\text{1}\;}$ and ${\;\lambda }_{\text{2}}\;$ are regularization parameters.

RFE is a model-based feature selection method. First, a machine learning model is specified or trained. Iterative training is subsequently performed on this designated model: in each training iteration, several of the least important features are eliminated, and the model is then retrained on the basis of the remaining feature subset. This process is repeated iteratively until a preset number of features or other stopping criteria are met. This method can gradually filter out noise among features, remove redundant features, reduce the feature dimension step by step, and retain the features that contribute the most to the model’s prediction performance.

#### Development of the feature selection model.

**Fig 1:**
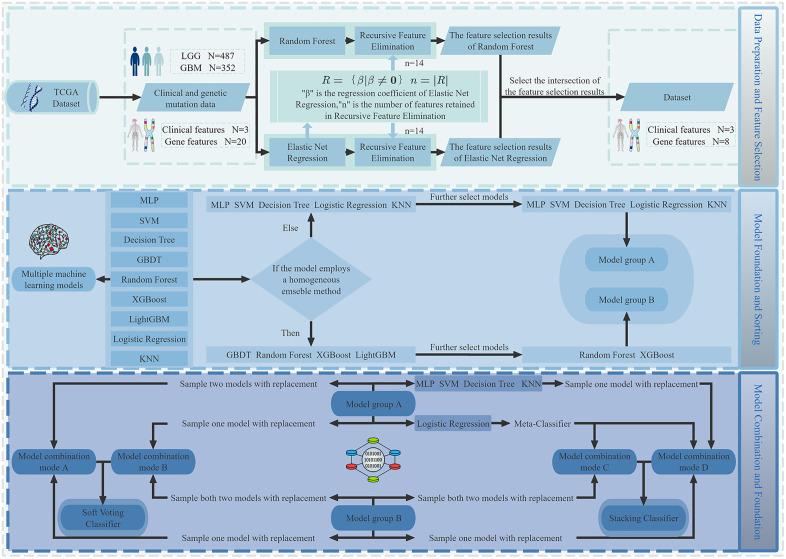
Technique roadmap. This flowchart illustrates the process from data acquisition and preprocessing, through feature selection (RFE + RF/ENR), model construction and optimization, to ensemble model integration using Voting and Stacking strategies. External validation was conducted using CGGA to assess model generalizability.

In this study, RF and ENR models were initially constructed for feature selection. The number of features with nonzero regression coefficients in the ENR is used as a benchmark, defined as the number of retained features for the RFE. The two models are subsequently retrained and subjected to feature selection again using RFE. Ultimately, the intersection of the feature subsets identified by both models is selected as the final feature set. The technical roadmap is illustrated in Fig 1.

### Model construction

#### Predictive model description.

The multilayer perceptron (MLP) is a feedforward neural network model that consists of an input layer, several hidden layers, and an output layer. Each neuron processes the input data by applying a weighted sum followed by nonlinear activation functions, and updates to the model parameters are performed using a backpropagation algorithm based on gradient descent.

A support vector machine (SVM) projects data into a high-dimensional space through a kernel function and identifies a hyperplane that maximizes the margin between data points of different classes, allowing it to handle both linear and nonlinear classification problems.

The decision tree (DT) model employs a tree structure to partition the dataset by selecting attributes and thresholds that maximize information gain (in ID3), the gain ratio (in C4.5), or the Gini index (in CART). New nodes are generated after each division, and this process continues until the predefined stopping conditions are met.

Gradient boosting trees (GBDT) employ the gradient boosting algorithm to iteratively train multiple decision tree models, with each tree trained to correct the prediction residuals (errors) from the previous trees. The final prediction is obtained by aggregating the predictions from all the trees.

The random forest (RF) method involves the construction of multiple decision trees using the bagging algorithm, and in classification tasks, the final prediction is determined by majority voting on the predictions from each decision tree.

eXtreme Gradient Boosting (XGBoost) employs an optimized gradient boosting algorithm, utilizing an efficient weak learner iteration strategy and regularization techniques, which improve both speed and overall performance.

The Light Gradient Boosting Machine (LightGBM) algorithm is also an optimization algorithm based on the gradient boosting framework and employs a histogram-based decision tree algorithm. Through parallelization strategies and sparse optimization techniques, it significantly improves training speed and model performance.

Logistic regression (LR) is a well-established linear classification model that maps the output of a linear model to a probability value between 0 and 1 using the sigmoid function.

The K-nearest neighbours (KNN) algorithm computes the distance between the input sample and the training samples in the feature space, selects the nearest K samples, and performs classification or regression tasks on the basis of the categories of these K samples.

The soft voting ensemble method derives the predicted probabilities for each classification result from multiple classifiers, performs a weighted or averaging operation, and ultimately selects the classification result with the highest corresponding probability as the final prediction.

The stacking ensemble method employs the prediction results of multiple base learners as input features for training a meta-learner. Both ensemble strategies increase the prediction accuracy and robustness of the model while mitigating the risk of overfitting.

#### Predictive model development.

In this study, nine classic prediction models, namely, the MLP, SVM, DT, GBDT, RF, XGBoost, LightGBM, LR, and KNN models, were constructed. On the basis of these nine models, 34 ensemble models were further developed using the voting and stacking ensemble methods. During the model construction process, with the random state set to 42, the dataset was split into a training set and a test set at an 8:2 ratio. The SMOTE algorithm was subsequently independently applied to the training set for oversampling to ensure the independence of the test dataset and the external validation dataset.

The random-search HPO method was employed to tune the parameters of nine base models, including the MLP and SVM, with iterative searches conducted within the specified parameter ranges. The parameter search ranges and configuration settings established prior to model training are provided in S7 Table, while the finalized parameter information of the optimized models is presented in S8 Table.

When the 34 ensemble models in this study were constructed, factors such as differences in the algorithmic principles, performance, and combination effects of the models were considered. For heterogeneous ensemble models, when all base models exhibit relatively excellent performance, diversity is crucial for improving the performance of the ensemble model [40]. Therefore, after comprehensively evaluating the performance of the base models, this study first categorized the base models into two groups on the basis of whether they adopted homogeneous ensemble algorithms (e.g., bagging and boosting algorithms). Models with similar algorithms within each group were subsequently further screened. Finally, when models were selected for combination, models from both groups were included simultaneously. This approach strengthens the diversity and differences among the algorithms of the combined models, integrates the advantages of different algorithms, and thereby enhances the overall robustness and generalization ability of the ensemble models. Specifically, the models were divided into two groups: (i) Group A: MLP, SVM, DT, LR, and KNN; and (ii) Group B: GBDT, RF, XGBoost, and LightGBM. Among these, GBDT, XGBoost, and LightGBM are all built on the basis of gradient boosting algorithms and share similar algorithmic frameworks. On the basis of the comprehensive performance of these three models, the one with the most balanced performance was retained in this study. For both the voting and stacking ensemble strategies, we combined three models each, aiming to improve model performance while conserving computational resources.

For the voting ensemble strategy, models were selected from Group A and Group B simultaneously to construct 25 combination schemes. The specific strategies are as follows: (i) one model was selected from Group A, and two models (RF and the top-performing boosting algorithm model) were selected from Group B, resulting in the formation of 5 combination schemes; (ii) two models were selected from Group A, and one model (either RF or the top-performing boosting algorithm model, exclusively) was selected from Group B, resulting in 20 combination schemes. Additionally, the voting ensemble models in this study adopted an equal weighting strategy of 1:1:1 to integrate the output results of all the learners.

For the stacking ensemble strategy, the LR from Group A was designated the meta-learner. This streamlined binary classification linear model can effectively integrate the outputs of different base learners, enhance model performance, and ensure computational efficiency. The same meta-learner selection method has also been reported in multiple studies [41–50]. Two base learners were selected from the remaining models in Groups A and B, generating a total of 9 combination schemes. The specific strategies are as follows: (i) one model was selected from Group A and one from Group B simultaneously, producing 8 combination schemes; (ii) both models in Group B (RF and the top-performing boosting algorithm model) were selected, leading to 1 combination scheme. In total, 9 combination schemes for the stacking ensemble strategy were generated.

### Evaluation metrics

This study focused on the classification prediction task. After comparing the prediction results of the model on the test set and the external validation set with the distribution of real samples, we identified four key relationships: (1) true positive (TP), (2) false-positive (FP), (3) true negative (TN), and (4) false-negative (FN). We then calculate the following evaluation metrics:

(1) Accuracy: The proportion of correctly predicted samples among the total number of samples is calculated as follows: $\frac{TN+TP}{TP+TN+FP+FN}$**.** It indicates the overall accuracy of predictions.
(2) Precision: The proportion of samples predicted as positive that are actually positive is calculated as follows: $\frac{TP}{TP+FP}$. It reflects the accuracy of positive predictions.
(3) Recalling: The proportion of actual positive samples that are correctly identified as positive is calculated as follows: $\frac{TP}{TP+FN}.\;\text{I}$t reflects the ability of the model to identify positive cases.
(4) F1 score: $\;2\times \frac{Precision\times Recall}{\;Precision+\;Recall}$.

The AUC was chosen as the primary performance metric because it summarises the classification ability across all the thresholds. AUC values were computed for all the models, and 95% confidence intervals were derived by bootstrapping with 1000 resamples. Because very high precision can underdiagnose, whereas very high recall can overdiagnose, the F1 score was also emphasized. This approach provides a balanced view of under- and overdiagnosis. Learning curves for the nine base models were plotted to assess the risk of overfitting. A calibration curve was used to assess probability calibration, and decision curve analysis (DCA) was used to quantify clinical utility. Finally, SHAP analysis elucidated the ensemble decision processes. The confusion matrices corresponding to the predictions of all models on both the TCGA test set and CGGA validation set are provided in S9 and S10 Files.

### Statistical analysis

Statistical analyses and model construction were performed in R 4.4.1 and Python 3.12.2. The complete analysis code is provided in S1 File (Python) and S2 File (R). Qualitative variables are summarized as frequencies and percentages (n%), whereas quantitative variables are reported as the means ± standard deviations (medians ± IQRs). Correlation hypotheses were tested for each variable against the label with α = 0.05. For qualitative variables, Fisher’s exact test was applied when any expected cell count was < 5; otherwise, the chi-square test was used. For quantitative variables, significant departures from normality detected by the Anderson–Darling test prompted the use of the Mann–Whitney U test. Cramér’s V was calculated to visualise correlations among categorical variables, and the point–biserial coefficient was computed to depict associations between quantitative and binary variables.

## Results

### Feature selection

**Fig 2:**
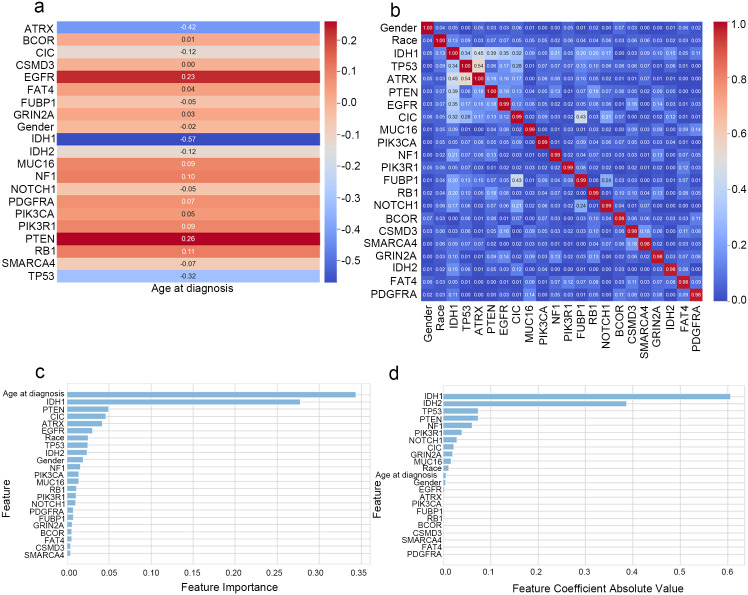
The multi-dimensional feature correlation and importance analysis. **(A)** Age correlations with binary traits (point-biserial). **(B)** Categorical feature correlations (Cramer’s V). **(C)** Feature importance ranking based on RF. **(D)** Feature importance ranking by ENR coefficients. These charts evaluate feature relationships and model contributions.

This study assesses correlations between continuous-to-binary and categorical pairs with the point–biserial coefficient and Cramér’s V, and visualises the results in two heat maps. As illustrated in Fig 2A, the associations of markers such as PTEN, EGFR, IDH1, and ATRX differ with age. The data in Fig 2B show predominantly weak feature correlations, although TP53 and ATRX display a relatively strong association.

Additionally, correlation hypothesis tests were run for every variable against glioma grade (label). These tests revealed significant associations for most features with glioma grade (p < 0.05).

In summary, RFE was integrated with RF and ENR for feature screening. This strategy mitigates collinearity, reduces redundancy, and isolates predictors with high discriminative value. The data in Fig 2C indicate that the top three RF importance scores were assigned to age, IDH1, and PTEN, whereas Fig 2D shows that the largest absolute ENR coefficients belonged to IDH1, IDH2, and TP53. Fourteen predictors were retained after recursive elimination, and intersection analysis yielded 11 final inputs: EGFR, TP53, Race, IDH2, gender, NF1, MUC16, age, PTEN, IDH1, and CIC. Each model was externally validated on the CGGA under identical conditions.

### Performance of the base model

**Fig 3:**
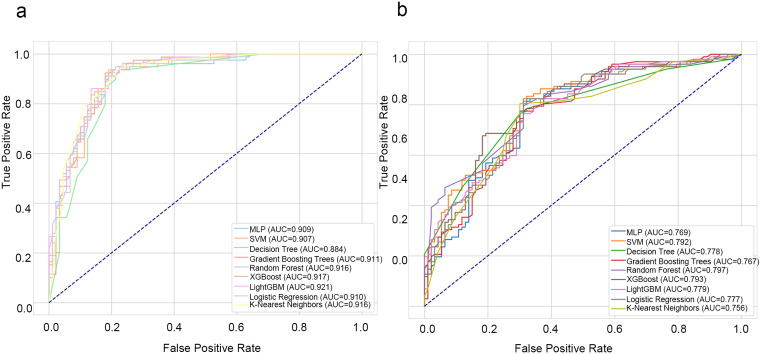
ROC curves of base models in test and validation sets. **(A)** ROC curves from the TCGA test set. **(B)** ROC curves from the CGGA validation set. These curves assess the predictive performance of base models across different cohorts.

Nine baseline models—MLP, SVM, DT, GBDT, RF, XGBoost, LightGBM, LR and KNN—were trained on the screened dataset. Learning curves were plotted for each model to evaluate potential overfitting; the curves suggested mild overfitting in several baselines (S1 Fig). The ROC curves for internal and external validation are shown in Fig 3A and 3B, respectively. Most curves lie near the upper-left corner, demonstrating high sensitivity, specificity and overall discriminative ability. The performance metrics are summarised in Table 2. During internal validation, the AUC of every test set exceeded 0.900, with LightGBM achieving the highest value (0.921), whereas DT yielded a lower AUC of 0.884 and an F1 score of 0.847. With respect to external validation, RF produced the greatest AUC (0.797), followed by XGBoost (0.793) and SVM (0.792). RF also obtained a test-set AUC of 0.916 and an external F1 score of 0.860, confirming robust generalisation; its ROC curve is close to the upper-left corner. The other indicators are also satisfactory. Moreover, the RF effectively controlled the model complexity and diversity through parameters such as n_estimators, criterion, and max_features. It achieved better generalisation in terms of the trade-off between bias and variance, highlighting the overall superior performance of RF among these nine baselines. The hyperparameter search spaces and optimal configurations are listed in Supplementary Tables S7 and S8, respectively.

**Table 2:** Performance of the basis models.

Model	Precision	Recall	F1-score	Accuracy	AUC-TCGA	AUC-CGGA
MLP	0.800	0.911	0.852	0.851	0.909	0.769
SVM	0.796	0.937	0.860	0.857	0.907	0.792
DT	0.791	0.911	0.847	0.845	0.884	0.778
GBDT	0.791	0.911	0.847	0.845	0.911	0.767
RF	0.796	0.937	0.860	0.857	0.916	0.797
XGBoost	0.811	0.924	0.864	0.863	0.917	0.793
LightGBM	0.800	0.911	0.852	0.851	0.921	0.779
LR	0.800	0.911	0.852	0.851	0.910	0.777
KNN	0.796	0.937	0.860	0.857	0.916	0.756

Precision, Recall, F1-score, Accuracy, and AUC-TCGA are all based on the output of the TCGA test set. AUC-CGGA is based on the output of the validation set on CGGA.

Calibration curves were used to assess the accuracy of the predicted probabilities. In both the TCGA test set and the CGGA external validation set, the calibration curves of most base models fluctuated near the ideal calibration curve, indicating a reasonable level of calibration in the predictive probabilities of the models. Decision curve analysis (DCA) was used to quantify clinical utility. On the TCGA test set, the curves for the MLP, RF, and some of the models above both treat – all and treat – no lines are observed when the threshold probabilities are between 0.2 and 0.8. Similarly, on the CGGA, similar benefits are observed for thresholds between 0.3 and 0.7. The calibration curves for all 43 models are provided in S3 File (TCGA) and S4 File (CGGA); the corresponding DCA results appear in S5 File (TCGA) and S6 File (CGGA). The 95% confidence intervals for the AUC values of all models on both the TCGA test set and CGGA validation set are provided in S5 and S6 Tables.

### Performance of the ensemble model

Considering algorithmic diversity and performance metrics, nine baseline learners were grouped and combined to construct multiple ensemble models. The test-set AUC of XGBoost (0.917) approximated that of LightGBM (0.921); however, its validation-set AUC reached 0.793, and its F1 score (0.864) exceeded those of all the individual baselines. Furthermore, the gamma parameter of XGBoost better uses the minimum reduction in loss brought about by the splitting of leaf nodes as the threshold, accurately suppressing unprofitable splits and preventing overfitting. Owing to its strong discrimination and generalisability, XGBoost outperformed GBDT and LightGBM and ranked immediately below RF. Accordingly, XGBoost was retained as the sole gradient-boosting learner, yielding a second group composed of RF and XGBoost. Ensemble models were subsequently assembled on the basis of this grouping, and the combination schemes are detailed in Table 3.

**Table 3:** Detailed combinations of ensemble models.

Model	Model Combination
Voting1	MLP + SVM + XGBoost
Voting2	MLP + DC + XGBoost
Voting3	MLP + LR + XGBoost
Voting4	MLP + KNN + XGBoost
Voting5	SVM + DC + XGBoost
Voting6	SVM + LR + XGBoost
Voting7	SVM + KNN + XGBoost
Voting8	DC + LR + XGBoost
Voting9	DC + KNN + XGBoost
Voting10	LR + KNN + XGBoost
Voting11	MLP + SVM + RF
Voting12	MLP + DC + RF
Voting13	MLP + LR + RF
Voting14	MLP + KNN + RF
Voting15	SVM + DC + RF
Voting16	SVM + LR + RF
Voting17	SVM + KNN + RF
Voting18	DC + LR + RF
Voting19	DC + KNN + RF
Voting20	LR + KNN + RF
Voting21	MLP + XGBoost+RF
Voting22	SVM + XGBoost+RF
Voting23	DC + XGBoost+RF
Voting24	LR + XGBoost+RF
Voting25	KNN + XGBoost+RF
Stacking1	MLP + XGBoost+LR(meta-learner)
Stacking2	MLP + RF + LR(meta-learner)
Stacking3	SVM + XGBoost+LR(meta-learner)
Stacking4	SVM + RF + LR(meta-learner)
Stacking5	DC + XGBoost+LR(meta-learner)
Stacking6	DC + RF + LR(meta-learner)
Stacking7	KNN + XGBoost+LR(meta-learner)
Stacking8	KNN + RF + LR(meta-learner)
Stacking9	XGBoost+RF + LR(meta-learner)

The table details the ensemble configurations, with voting using permutation combinations and stacking employing LR as the meta-learner.

**Fig 4:**
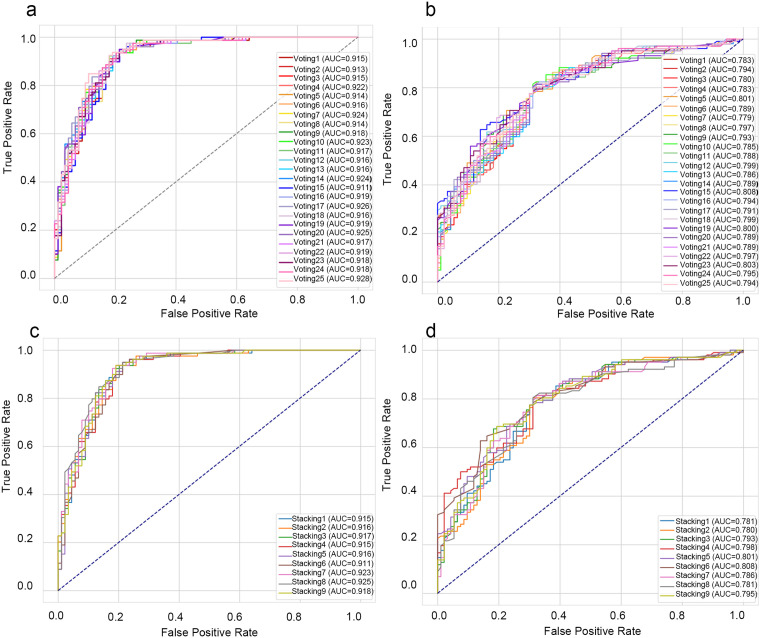
ROC curves of voting and Stacking ensemble models in test and validation sets. **(A)-(B)** Voting models’ ROC curves for TCGA test set and CGGA validation set, respectively. **(C)-(D)** Stacking models’ ROC curves for TCGA test and CGGA validation sets, evaluating ensemble performance.

The ROC curves for the voting ensembles in internal and external validation are summarised in Fig 4A and 4B, respectively, and the corresponding performance metrics are listed in Table 4. All the voting combinations show strong internal performance, with the external validation performance only marginally lower. Notably, voting25 yields the highest internal test-set AUC (0.928), underscoring its superior predictive capability among the voting ensembles. In contrast, voting15 has the greatest external AUC (0.808), which is relatively high for the validation set. Voting25 also has an F1 score of 0.860 on the test set and an external AUC of 0.794, confirming solid generalization. A formal statistical comparison of the ROC curves between the top-performing Voting25 model and the RF base model is presented in S9 Table. Overall, considering accuracy and generalisability, voting25 provides the most balanced and robust predictive performance.

**Table 4:** Performance of voting ensemble models.

Model	Precision	Recall	F1-score	Accuracy	AUC-TCGA	AUC-CGGA
Voting1	0.796	0.937	0.860	0.857	0.915	0.783
Voting2	0.793	0.924	0.854	0.851	0.913	0.794
Voting3	0.800	0.911	0.852	0.851	0.915	0.780
Voting4	0.804	0.937	0.865	0.863	0.922	0.783
Voting5	0.793	0.924	0.854	0.851	0.914	0.801
Voting6	0.796	0.937	0.860	0.857	0.916	0.789
Voting7	0.796	0.937	0.860	0.857	0.924	0.779
Voting8	0.793	0.924	0.854	0.851	0.914	0.797
Voting9	0.793	0.924	0.854	0.851	0.918	0.793
Voting10	0.804	0.937	0.865	0.863	0.923	0.785
Voting11	0.796	0.937	0.860	0.857	0.917	0.788
Voting12	0.793	0.924	0.854	0.851	0.916	0.799
Voting13	0.804	0.937	0.865	0.863	0.916	0.786
Voting14	0.796	0.937	0.860	0.857	0.924	0.789
Voting15	0.796	0.937	0.860	0.857	0.911	0.808
Voting16	0.796	0.937	0.860	0.857	0.919	0.794
Voting17	0.796	0.937	0.860	0.857	0.926	0.791
Voting18	0.793	0.924	0.854	0.851	0.916	0.799
Voting19	0.796	0.937	0.860	0.857	0.919	0.800
Voting20	0.796	0.937	0.860	0.857	0.925	0.789
Voting21	0.804	0.937	0.865	0.863	0.917	0.789
Voting22	0.796	0.937	0.860	0.857	0.919	0.797
Voting23	0.793	0.924	0.854	0.851	0.918	0.803
Voting24	0.804	0.937	0.865	0.863	0.918	0.795
Voting25	0.796	0.937	0.860	0.857	0.928	0.794

Precision, Recall, F1-score, Accuracy, and AUC-TCGA are all based on the output of the TCGA test set. AUC-CGGA is based on the output of the validation set on CGGA.

The ROC curves for all stacking ensembles in the internal and external validation are summarised in Fig 4C and 4D, respectively. Although the curves trend towards the upper-left corner, several crossings indicate that the AUC offers a clearer basis for comparison. As shown in Table 5, stacking8 achieves the highest internal AUC (0.925), reflecting strong discriminative power on the test set. Conversely, stacking6 attains an external AUC of 0.808, indicating good generalization. Notably, stacking7 has a test-set AUC of 0.923—comparable with stacking8—and an F1 score of 0.865, along with a favourable external AUC. The other metrics remain stable, suggesting that Stacking7 provides consistently strong overall performance.

**Table 5:** Performance of stacking ensemble models.

Model	Precision	Recall	F1-score	Accuracy	AUC-TCGA	AUC-CGGA
Stacking1	0.800	0.911	0.852	0.851	0.915	0.781
Stacking2	0.800	0.911	0.852	0.851	0.916	0.780
Stacking3	0.796	0.937	0.860	0.857	0.917	0.793
Stacking4	0.796	0.937	0.860	0.857	0.915	0.798
Stacking5	0.791	0.911	0.847	0.845	0.916	0.801
Stacking6	0.791	0.911	0.847	0.845	0.911	0.808
Stacking7	0.804	0.937	0.865	0.863	0.923	0.786
Stacking8	0.796	0.937	0.860	0.857	0.925	0.781
Stacking9	0.796	0.937	0.860	0.857	0.918	0.795

Precision, Recall, F1-score, Accuracy, and AUC-TCGA are all based on the output of the TCGA test set. AUC-CGGA is based on the output of the validation set on CGGA.

**Fig 5:**
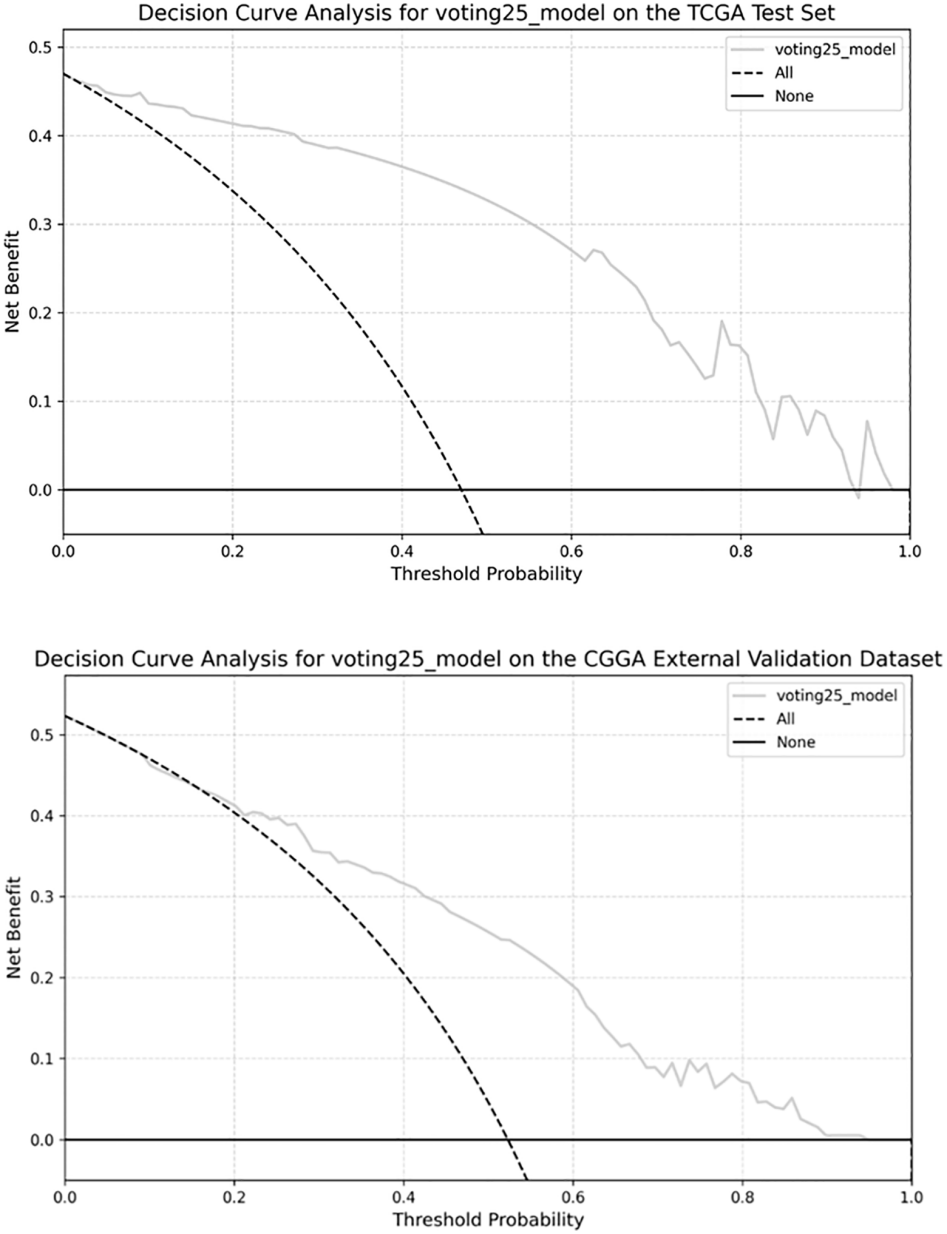
DCA of voting25 model.

In both the TCGA test set and the CGGA validation set, the calibration curves for most ensembles fluctuated only slightly around the ideal line. Decision curve analysis (DCA) revealed that in the TCGA cohort, several ensembles outperform the treat-all and treat-none strategies for threshold probabilities between 0.1 and 0.9, demonstrating clinical utility across this range. With respect to the CGGA, a similar benefit appears for thresholds between 0.3 and 0.7. The DCA curve for voting25, which highlights its clinical utility within the pertinent threshold range, is shown in Fig 5.

**Fig 6:**
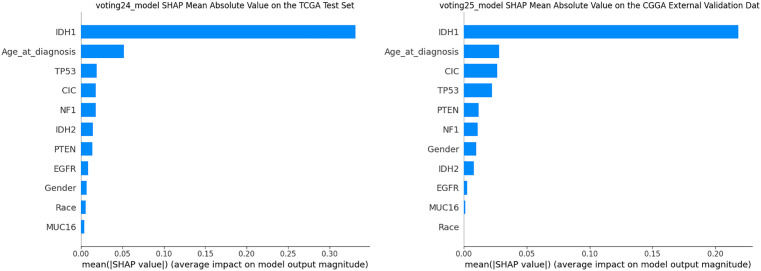
SHAP mean absolute value curves of voting25 model.

SHAP analysis was applied to every model to interpret its decision process. The depicts feature-importance rankings for voting25 in the TCGA test set and the CGGA validation set are shown in Fig 6. IDH1, age at diagnosis, CIC and PTEN received the high scores SHAP scores, indicating substantial contributions to model output. The complete SHAP results for all ensemble models on both the TCGA and CGGA datasets are provided in S7 and S8 Files, respectively.

## Discussion

To circumvent the haemorrhage, infection, and sampling error risks inherent in conventional pathology-based grading [51,52], a minimally invasive glioma-grading model was developed with the TCGA dataset. The model was built via feature selection, five-fold cross-validation, random HPO, SMOTE oversampling, and ensemble learning, and its robustness was validated on the CGGA dataset. By integrating molecular information with morphological information, the model overcomes single-modality limits and provides reproducible, objective evidence for pathological grading because of its demonstrated stability and generalisability. Potential clinical uses include (i) molecular pregrading to avoid unnecessary biopsy in contraindicated patients [53] and (ii) decision curve analysis (DCA), which is useful as an adjunct for borderline cases [54], while final management still requires individualised clinical judgement.

The least absolute shrinkage and selection operator (LASSO) is an established feature-selection technique [55]. Strong collinearity was observed between several predictors, including TP53 and ATRX. The aggressive sparsity imposed by LASSO can bias coefficient estimates when predictors are highly correlated [56,57]. Elastic-net regression (ENR), which combines the L1 and L2 penalties, unites the benefits of ridge regression and LASSO [58,59]. ENR therefore controls both shrinkage and sparsity, a useful compromise when feature selection is needed under high collinearity. Accordingly, recursive feature elimination (RFE) coupled with RF and ENR was adopted to mitigate collinearity and remove redundancy. Previous studies have confirmed that the retained features are strongly linked to glioma grade. For instance, IDH1 mutations occur in > 70% of low-grade gliomas (LGGs) [60], EGFR amplification appears in > 50% of glioblastomas (GBMs) [61], and TP53 is an independent prognostic marker for LGG, although its mechanism remains unclear [62]. These observations underpin the biological and clinical rationale for the chosen feature set.

The 11-feature model—IDH1, PTEN, TP53, and eight others—yielded AUC values > 0.90, indicating strong predictive capability. It integrates key biomarkers and clinical variables to facilitate grade prediction. Zhang et al. [63] used multiparametric MRI with RF to distinguish LGG from HGG, achieving an AUC of 0.81 and an F1 score of 0.88. In the present work, RF achieved an AUC of 0.916 for the TCGA cohort and 0.797 for the CGGA cohort, confirming its advantage in grade prediction. Hao et al. [64] combined RF and XGBoost on cuprotosis-related lncRNAs/mRNAs to predict survival in LGG and GBM patients. Tasci et al. [65] reported that a soft-voting ensemble of RF, SVM and AdaBoost performed well on the TCGA (AUC 0.914; F1 score 0.842). Here, the optimal voting ensemble (RF + XGBoost + KNN) yielded an AUC of 0.928 and an F1 score of 0.860 for the TCGA cohort, further improving performance. Du et al. [66] constructed a multimodal MR-radiomics model; the best single classifier reached an AUC of 0.85, whereas our RF (AUC of 0.916) and voting25 (AUC of 0.928) models outperformed that model.

Ensemble learning is widely recognized for its ability to improve robustness. For example, it has been applied to deep Gaussian mixture models [67], mirroring the ensemble-based strategy adopted here. Waqas et al. [68,69] reported that stacking refines decision thresholds and bolsters robustness in complex multi-instance learning (MIL) settings. Accordingly, this work adopts a combined stacking + voting framework. The hybrid design strengthens base-model complementarity and enhances generalisation. Numerous studies report an ensemble performance that surpasses that of individual models [70–72]; however, the ensembles here provided only marginal gains over the already optimised single models. This outcome likely reflects the high baseline performance achieved after targeted optimisation, leaving limited room for ensemble uplift, a phenomenon also noted elsewhere [73,74]. Even when accuracy gains are minor, ensembles show greater resistance to overfitting and better generalisability than baselines do, benefits that remain valuable [33,75,76].

### Limitations of our study

Several limitations should be acknowledged. First, the TCGA and CGGA datasets are retrospective public cohorts without prospective, multicentre, or real-world clinical data, limiting the generalisability and clinical applicability of the models [13,77]. Although independent external validation was performed, residual bias may persist because of source limitations and small sample sizes—especially the paucity of rare glioma subtypes in the TCGA—thereby reducing the predictive accuracy for these categories [70]. Moreover, the TCGA is heavily skewed towards Caucasian patients, further constraining its transferability to other ethnicities [78]. Future work should therefore recruit large, ethnically diverse, prospective multicentre cohorts to improve generalisability and translational potential.

Second, to correct class imbalance, SMOTE was applied. However, SMOTE can create overly smooth synthetic cases, inflating performance estimates and heightening overfitting risk [79]. Future studies might test advanced augmentations—for example, GAN-based synthesis [80]—or validate the model on real-world data to capture greater biological diversity and increase robustness.

Finally, the lack of comprehensive radiomics prevented head-to-head comparisons with emerging deep-learning or multimodal fusion models, partially limiting the assessment of novelty. Nevertheless, the present clinically and molecularly driven model retains strong biological interpretability and practical utility [80].

Integrating machine learning with medical imaging offers a new avenue for glioma grading. Multiple-instance learning (MIL), a weakly supervised paradigm, has achieved breakthroughs in imaging analysis [81,82] and serves as a methodological reference for the present work. Radiomic prediction of molecular traits—for example, 1p/19q codeletion [83,84]—confirms the clinical value of multimodal integration. Accordingly, future efforts will systematically merge imaging genomics with clinical data and apply advanced machine-learning techniques to increase performance and expedite clinical translation.

## Conclusions

For models built with 11 key features—including IDH1, PTEN, and TP53—the AUC for the TCGA dataset exceeded 0.90 in nearly every case. During the external validation of the CGGA dataset, both the baseline and the ensemble models performed robustly, with RF and the soft-voting ensemble (RF + XGBoost + KNN) delivering the best overall results. These findings provide a novel strategy for glioma grading and diagnosis.

## Supporting information

S1 Fig AUC learning curves for base models on TCGA data. (TIF)

S1 Table CGGA clinical and genetic mutation data (uncoded). (CSV)

S2 Table CGGA clinical and genetic mutation data (coded). (CSV)

S3 Table TCGA clinical and genetic mutation data (uncoded). (CSV)

S4 Table TCGA clinical and genetic mutation data (coded). (CSV)

S5 Table 95% confidence intervals of AUC values for models on the TCGA test set. (XLSX)

S6 Table 95% confidence intervals of AUC values for models on the CGGA external validation dataset. (XLSX)

S7 Table Random search hyperparameter ranges for models. (XLSX)

S8 Table Model parameters after training completion. (XLSX)

S9 Table DeLong test result for voting25 model and RF. (XLSX)

S1 File Code for building predictive models. (IPYNB)

S2 File Code for statistical analysis. (R)

S3 File Calibration curve for models on the TCGA test set. (ZIP)

S4 File Calibration curve for models on the CGGA external validation dataset. (ZIP)

S5 File Decision curve analysis for models on the TCGA test set. (ZIP)

S6 File Decision curve analysis for models on the CGGA external validation dataset. (ZIP)

S7 File SHAP results for models on the TCGA test set. (ZIP)

S8 File SHAP results for models on the CGGA external validation dataset. (ZIP)

S9 File The confusion matrix of models on the TCGA test set. (ZIP)

S10 File The confusion matrix of models on the CGGA external validation dataset. (ZIP)

S11 File The deployment program of the voting25 model. (PKL)

## Abbreviations

LGG: Low-grade glioma
HGG: High-grade gliomas
WHO: World Health Organization
GBM: Glioblastoma
ML: Machine Learning
TCGA: The Cancer Genome Atlas
RFE: Recursive Feature Elimination
RF: Random Forest
ENR: Elastic Net Regression
SMOTE: Synthetic Minority Over-sampling Technique
KNN: K-Nearest Neighbors
SVM: Support Vector Machine
HPO: Hyper-Parameter Optimization
CGGA: Chinese Glioma Genome Atlas
SHAP: SHapley Additive exPlanations
XGBoost: eXtreme Gradient Boosting
EL-APMC: Adaptive Power Mean Combiner
DTI: Diffusion tensor imaging
MLP: Multi-Layer Perceptron
DT: Decision Tree
GBDT: Gradient Boosting Decision Tree
LightGBM: Light Gradient Boosting Machine
LR: Logistic Regression
ROC: Receiver Operating Characteristic
AUC: Area Under the Curve
DCA: Decision Curve Analysis.
