# Supplementary figures and images for: Machine learning-based prediction of glioma grading (part 1 of 5)
Source: PLoS One. 2025 Dec 26;20(12):e0314831. doi: 10.1371/journal.pone.0314831 (PMC12742763; doi:10.1371/journal.pone.0314831)

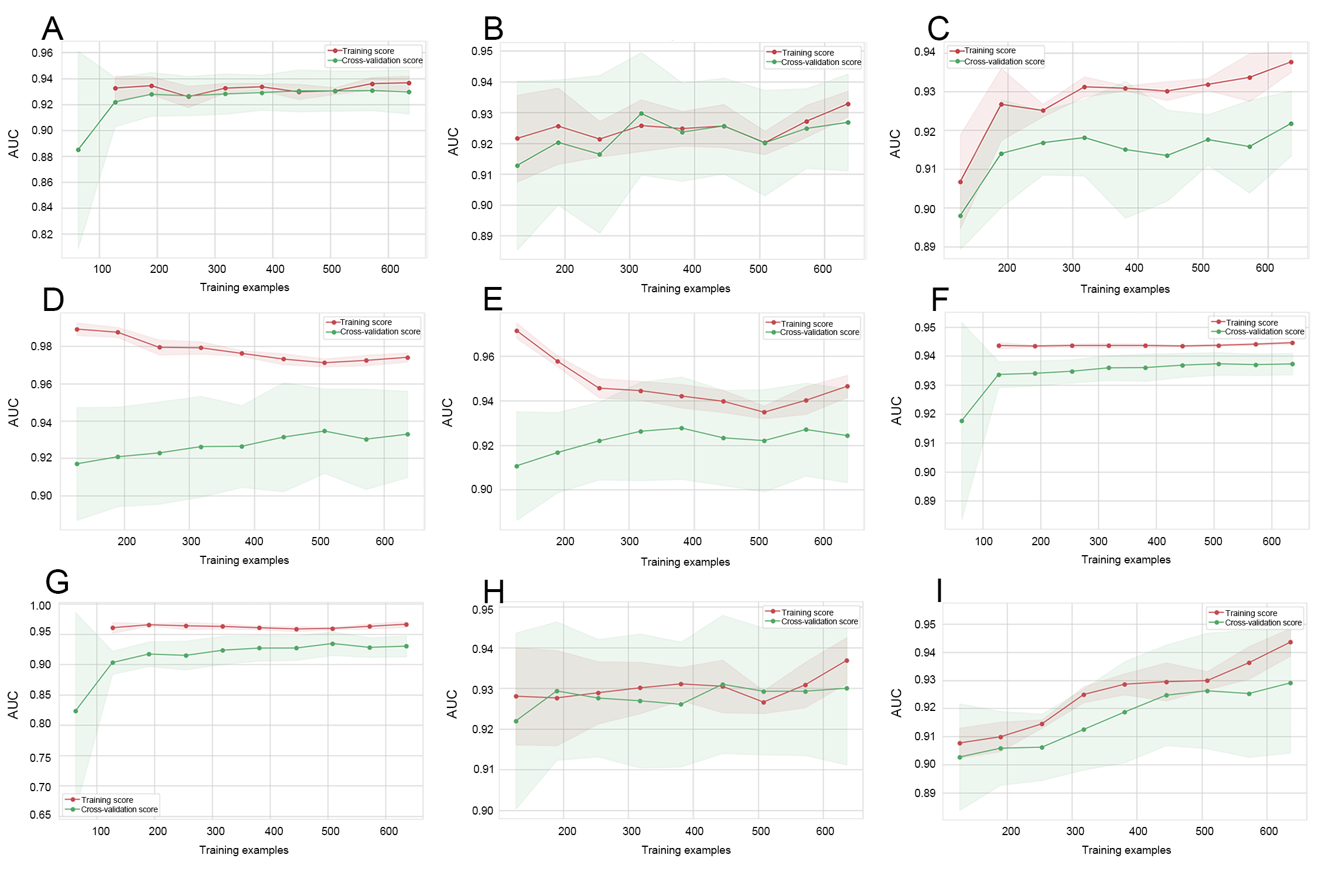

Supplement: S1 Fig — (TIF) [file pone.0314831.s001.tif]

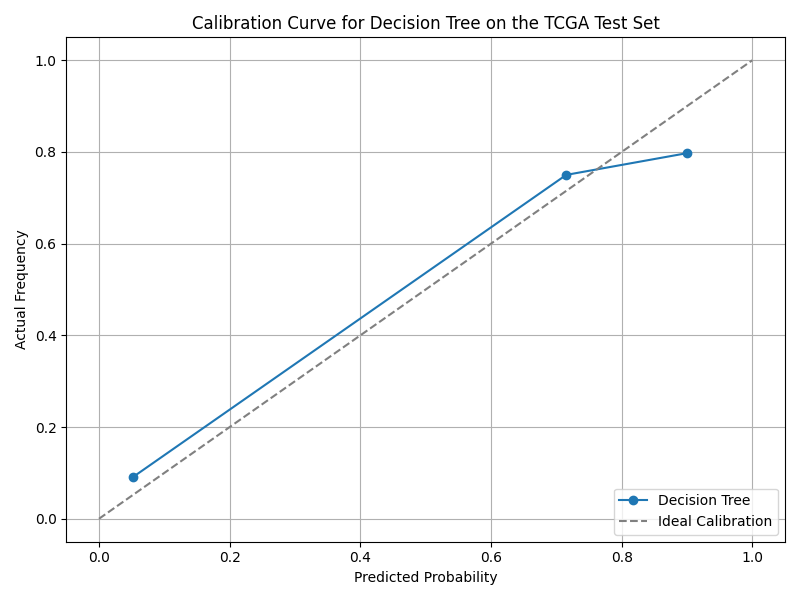

Supplement: S3 File — (ZIP) [file pone.0314831.s013.zip › S3 File/Decision Tree_calibration_curve.png]

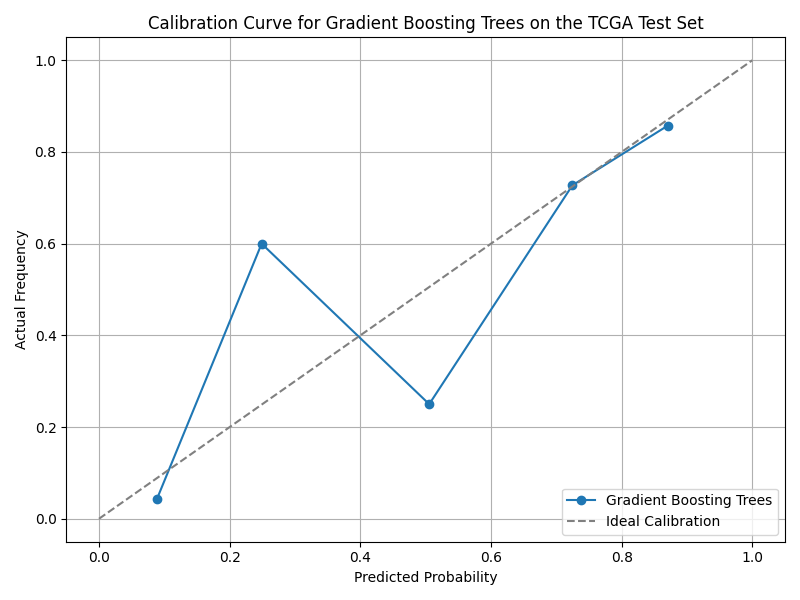

Supplement: S3 File — (ZIP) [file pone.0314831.s013.zip › S3 File/Gradient Boosting Trees_calibration_curve.png]

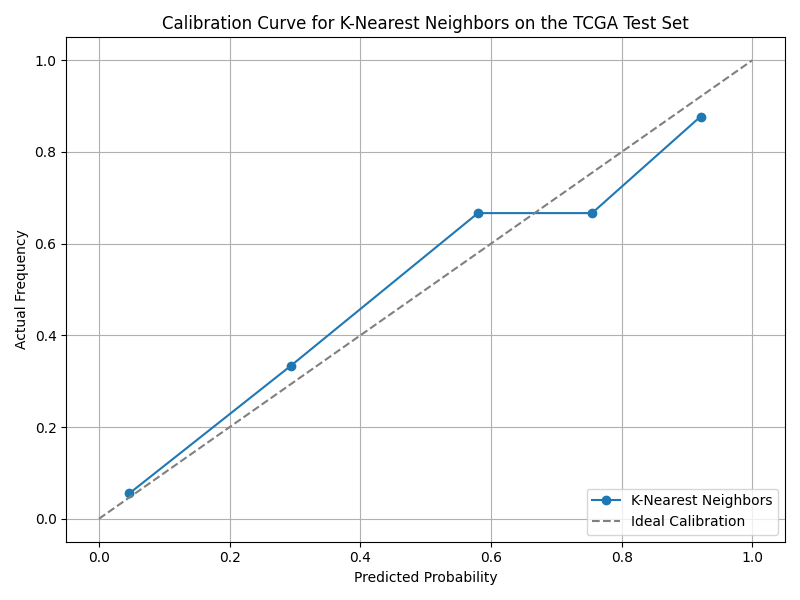

Supplement: S3 File — (ZIP) [file pone.0314831.s013.zip › S3 File/K-Nearest Neighbors_calibration_curve.png]

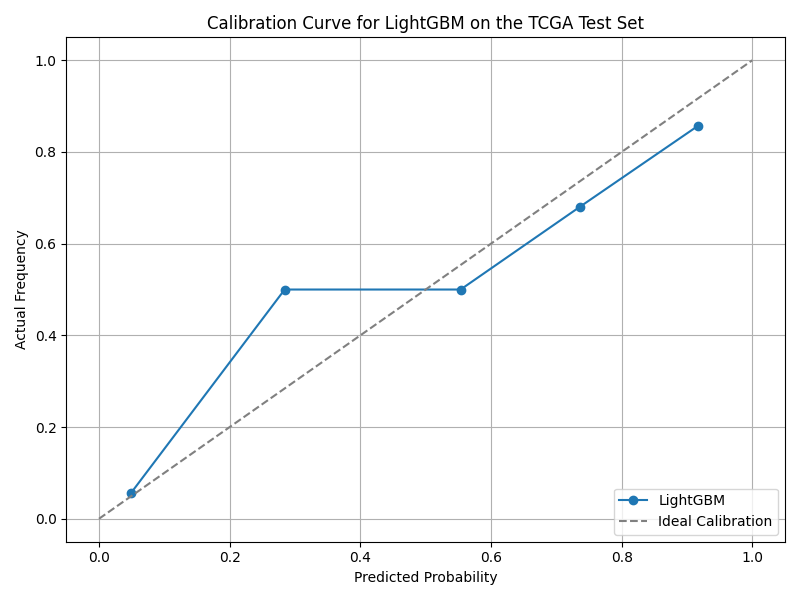

Supplement: S3 File — (ZIP) [file pone.0314831.s013.zip › S3 File/LightGBM_calibration_curve.png]

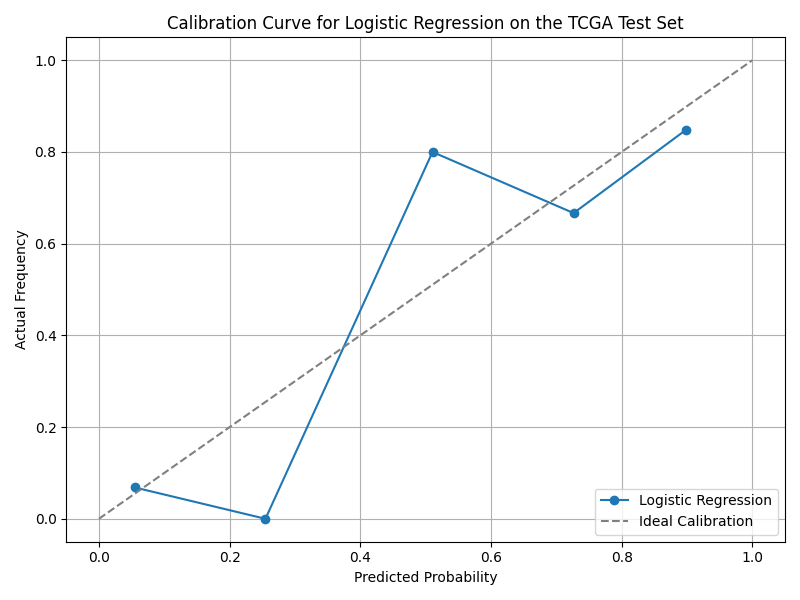

Supplement: S3 File — (ZIP) [file pone.0314831.s013.zip › S3 File/Logistic Regression_calibration_curve.png]

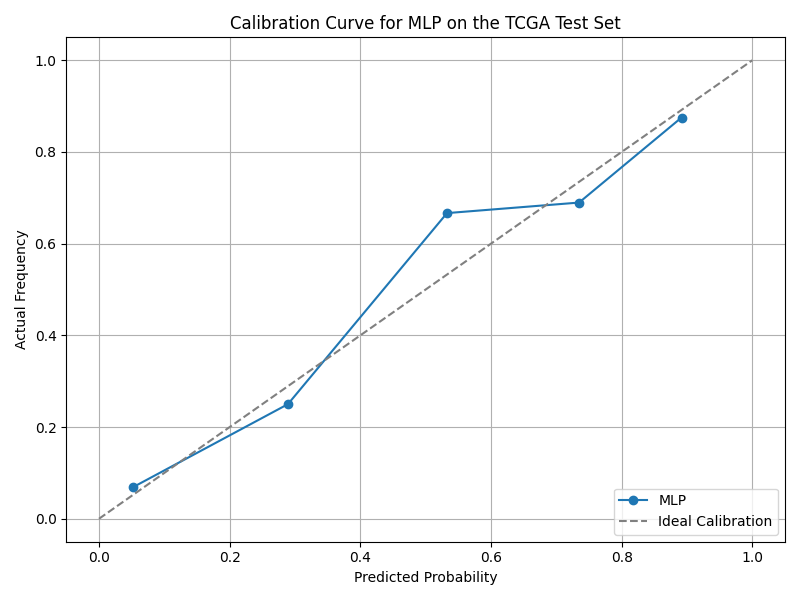

Supplement: S3 File — (ZIP) [file pone.0314831.s013.zip › S3 File/MLP_calibration_curve.png]

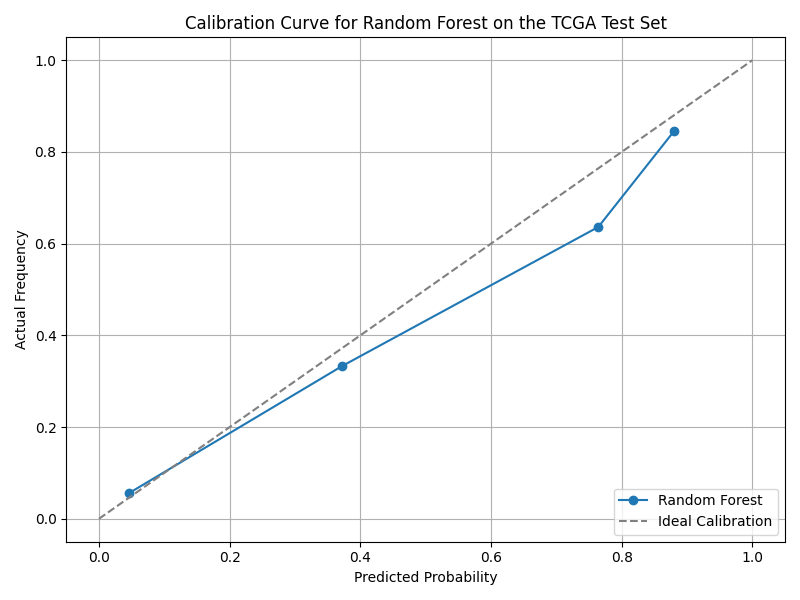

Supplement: S3 File — (ZIP) [file pone.0314831.s013.zip › S3 File/Random Forest_calibration_curve.png]

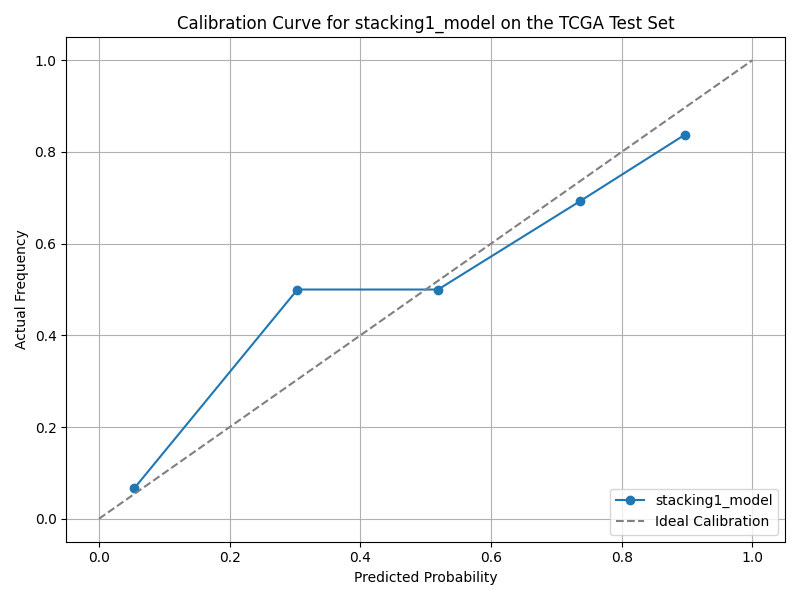

Supplement: S3 File — (ZIP) [file pone.0314831.s013.zip › S3 File/stacking1_model_calibration_curve.png]

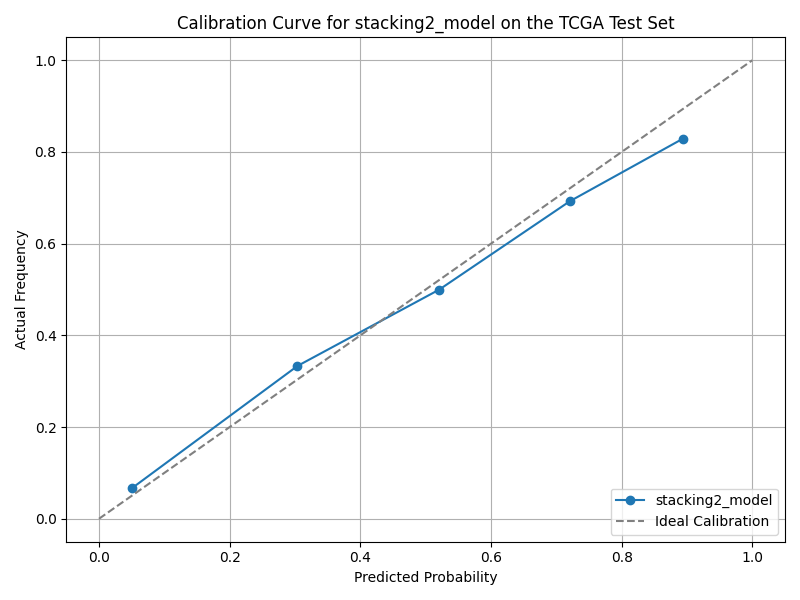

Supplement: S3 File — (ZIP) [file pone.0314831.s013.zip › S3 File/stacking2_model_calibration_curve.png]

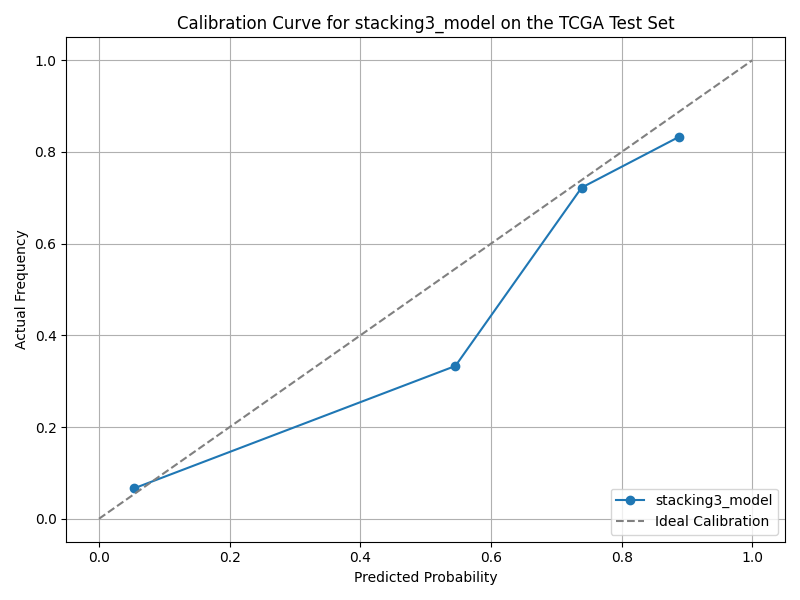

Supplement: S3 File — (ZIP) [file pone.0314831.s013.zip › S3 File/stacking3_model_calibration_curve.png]

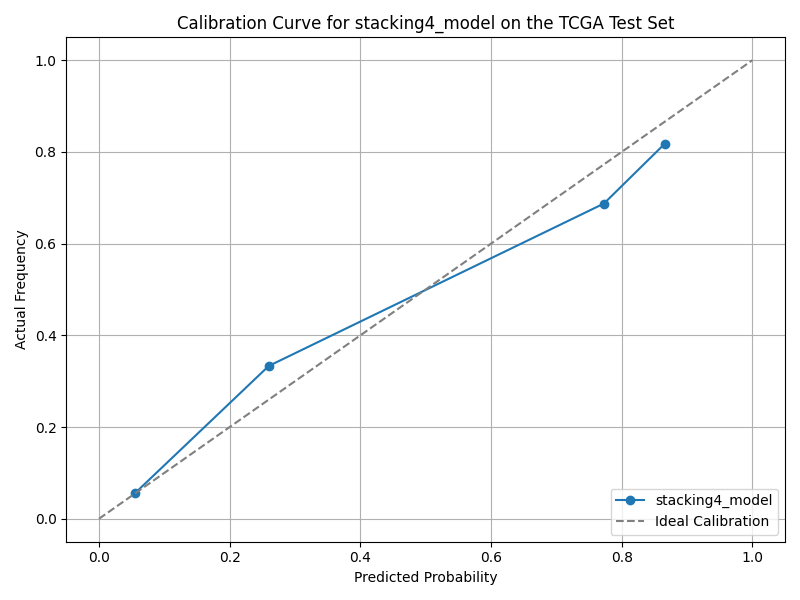

Supplement: S3 File — (ZIP) [file pone.0314831.s013.zip › S3 File/stacking4_model_calibration_curve.png]

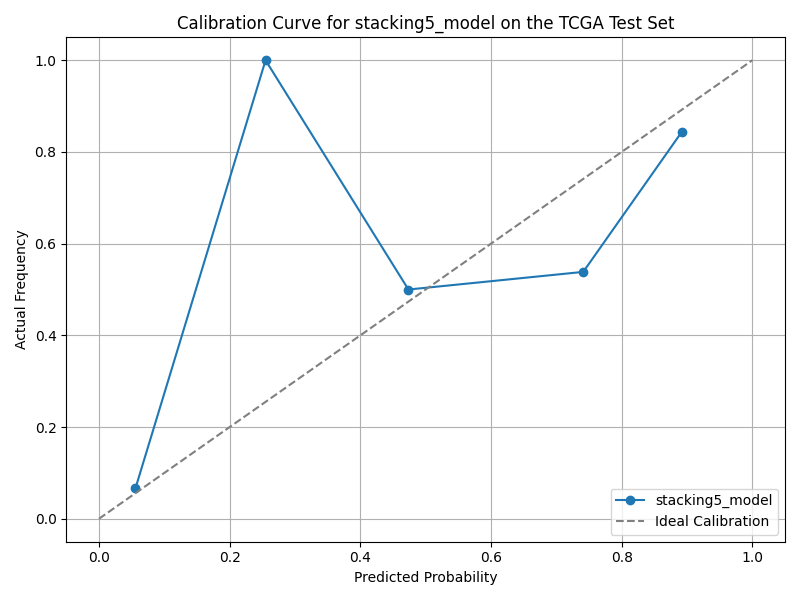

Supplement: S3 File — (ZIP) [file pone.0314831.s013.zip › S3 File/stacking5_model_calibration_curve.png]

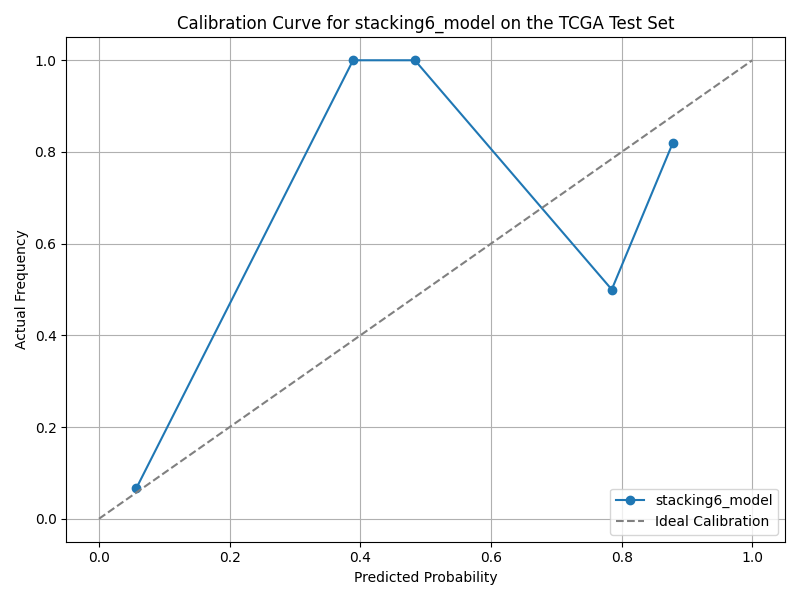

Supplement: S3 File — (ZIP) [file pone.0314831.s013.zip › S3 File/stacking6_model_calibration_curve.png]

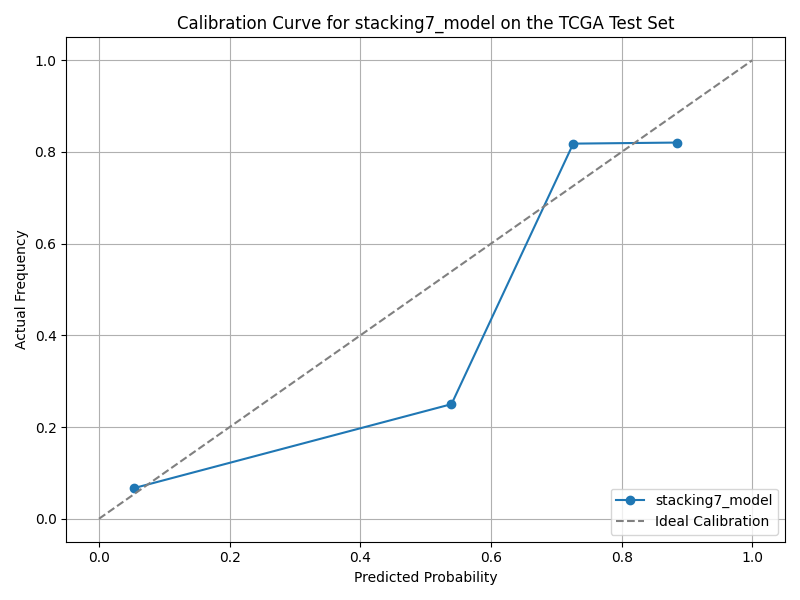

Supplement: S3 File — (ZIP) [file pone.0314831.s013.zip › S3 File/stacking7_model_calibration_curve.png]

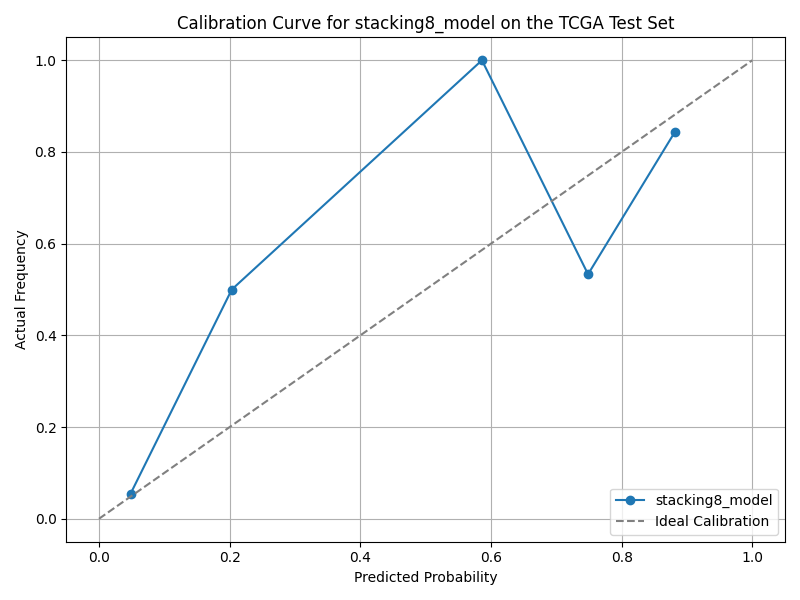

Supplement: S3 File — (ZIP) [file pone.0314831.s013.zip › S3 File/stacking8_model_calibration_curve.png]

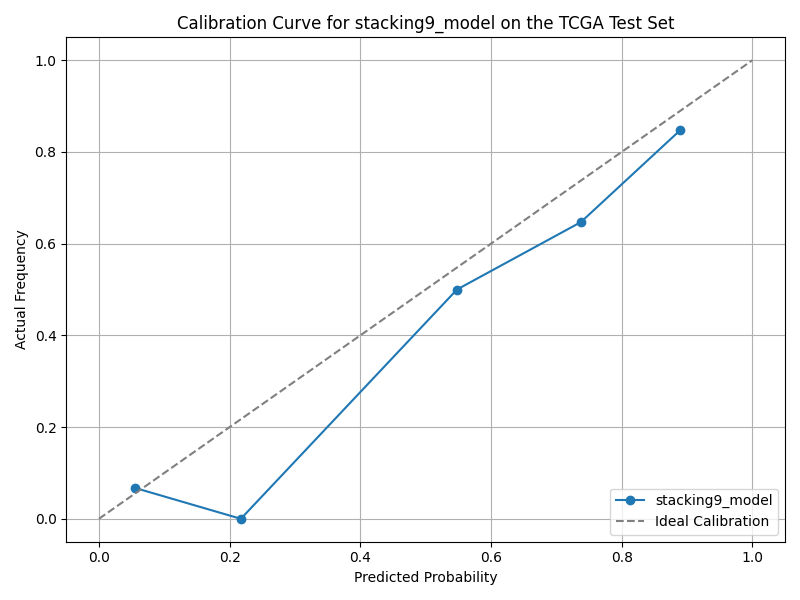

Supplement: S3 File — (ZIP) [file pone.0314831.s013.zip › S3 File/stacking9_model_calibration_curve.png]

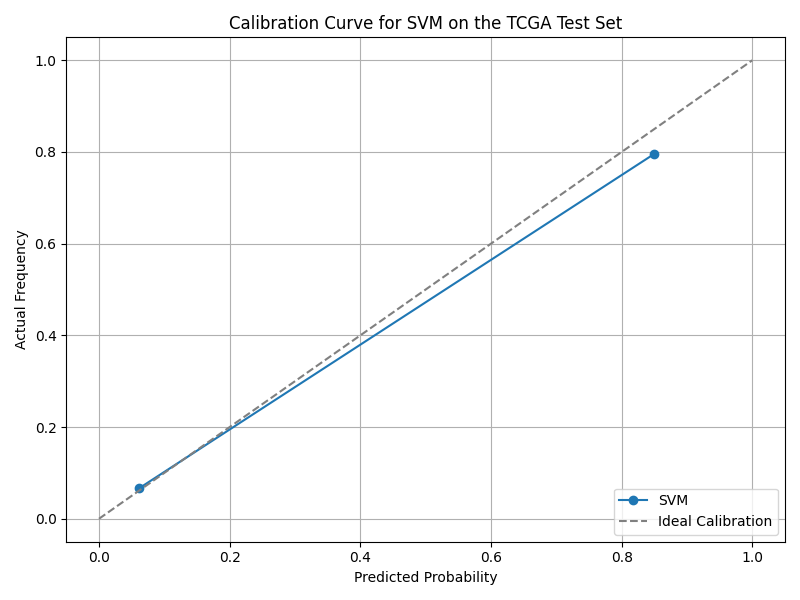

Supplement: S3 File — (ZIP) [file pone.0314831.s013.zip › S3 File/SVM_calibration_curve.png]

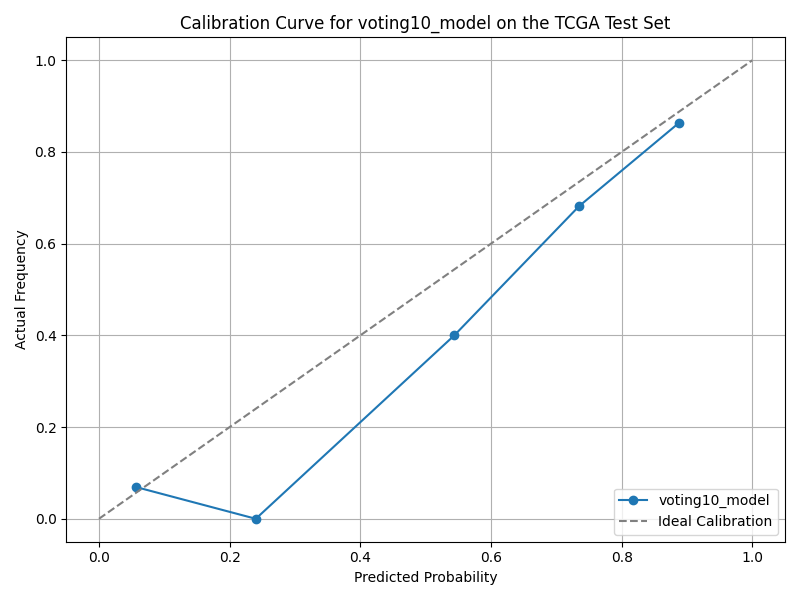

Supplement: S3 File — (ZIP) [file pone.0314831.s013.zip › S3 File/voting10_model_calibration_curve.png]

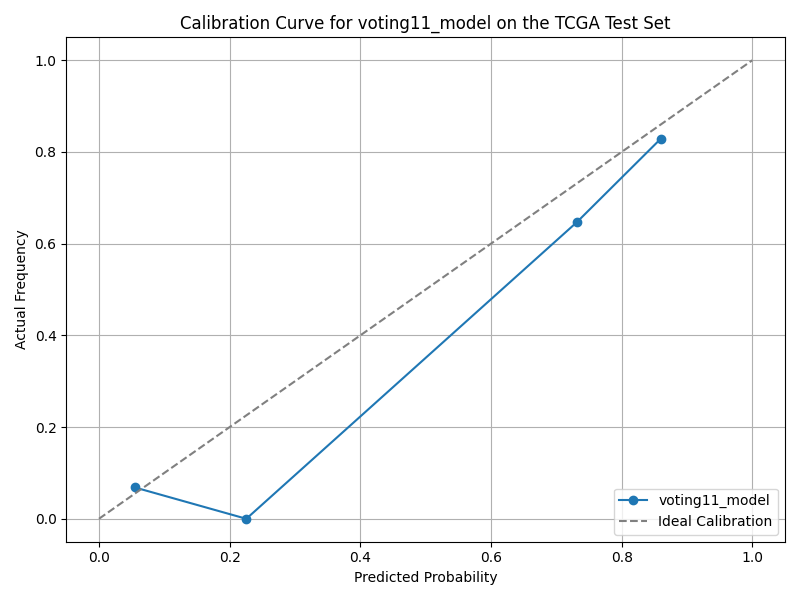

Supplement: S3 File — (ZIP) [file pone.0314831.s013.zip › S3 File/voting11_model_calibration_curve.png]

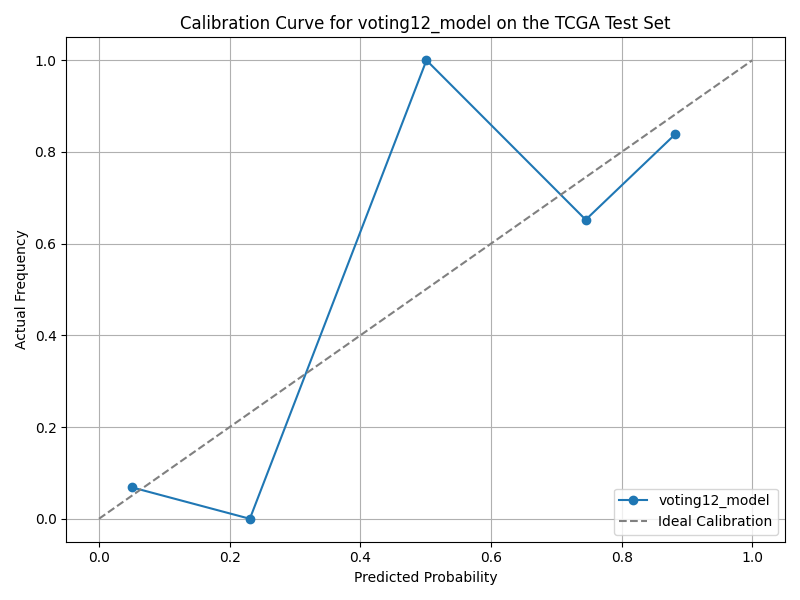

Supplement: S3 File — (ZIP) [file pone.0314831.s013.zip › S3 File/voting12_model_calibration_curve.png]

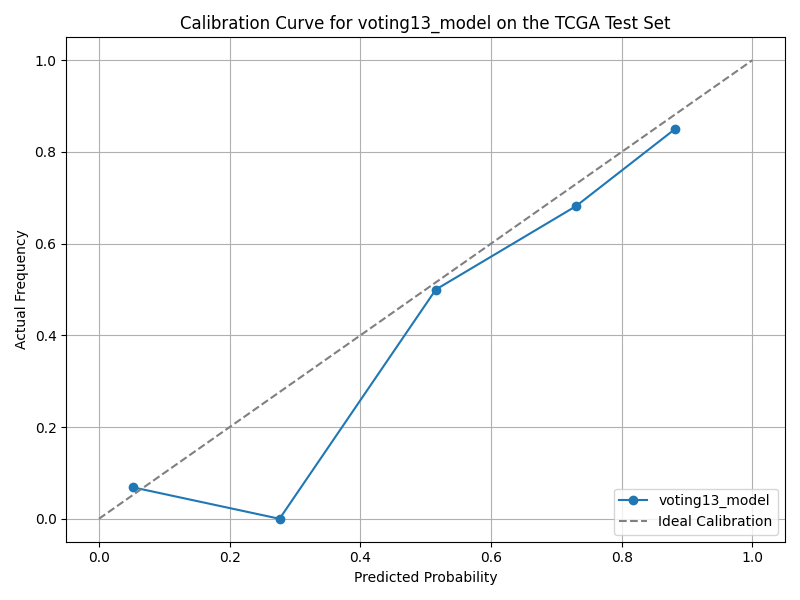

Supplement: S3 File — (ZIP) [file pone.0314831.s013.zip › S3 File/voting13_model_calibration_curve.png]

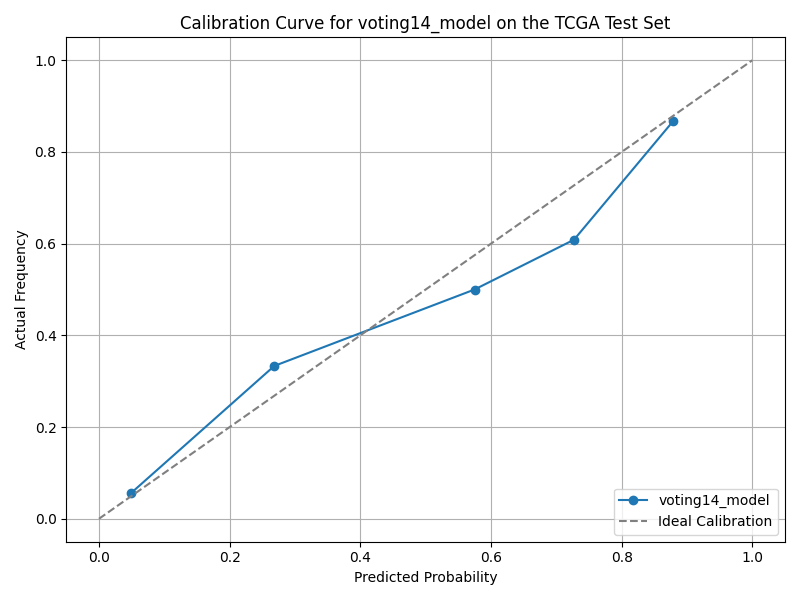

Supplement: S3 File — (ZIP) [file pone.0314831.s013.zip › S3 File/voting14_model_calibration_curve.png]

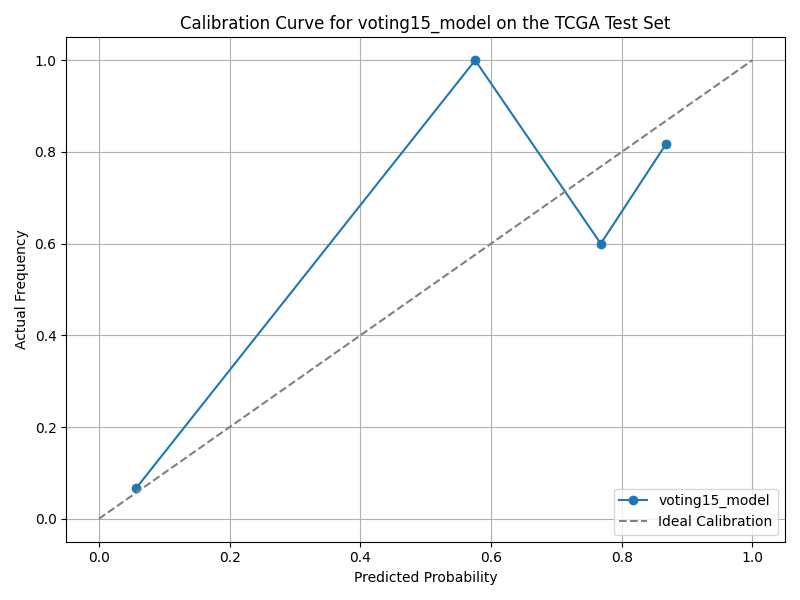

Supplement: S3 File — (ZIP) [file pone.0314831.s013.zip › S3 File/voting15_model_calibration_curve.png]

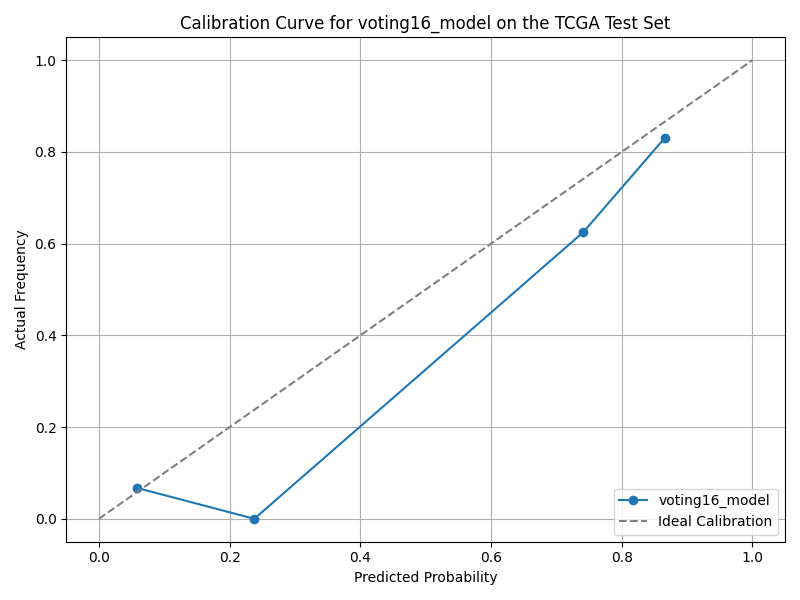

Supplement: S3 File — (ZIP) [file pone.0314831.s013.zip › S3 File/voting16_model_calibration_curve.png]

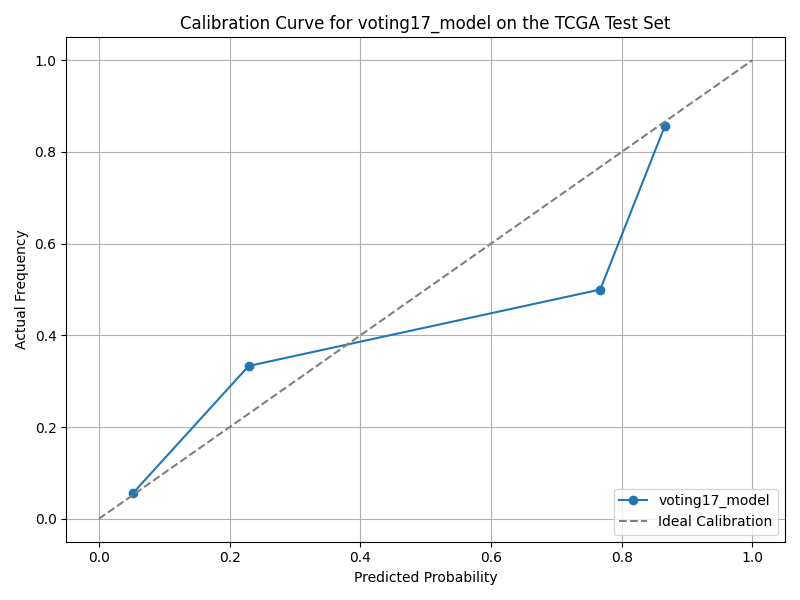

Supplement: S3 File — (ZIP) [file pone.0314831.s013.zip › S3 File/voting17_model_calibration_curve.png]

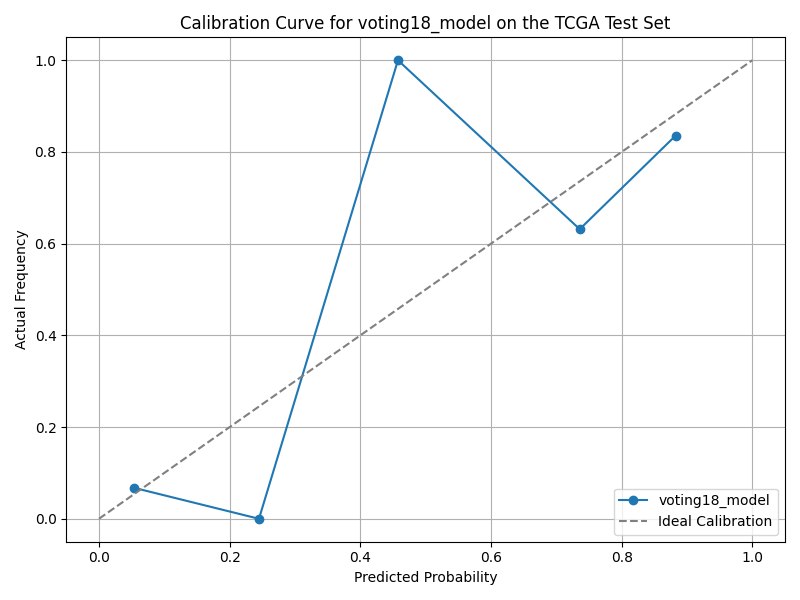

Supplement: S3 File — (ZIP) [file pone.0314831.s013.zip › S3 File/voting18_model_calibration_curve.png]

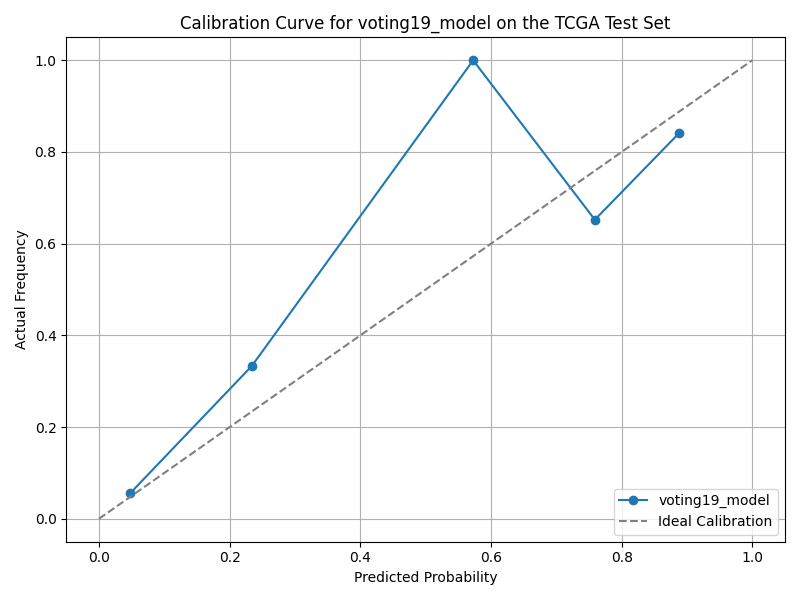

Supplement: S3 File — (ZIP) [file pone.0314831.s013.zip › S3 File/voting19_model_calibration_curve.png]

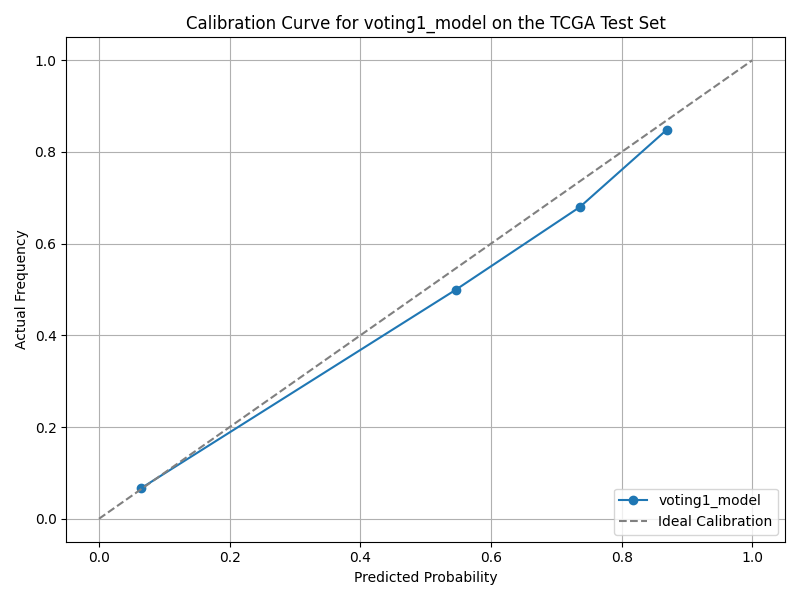

Supplement: S3 File — (ZIP) [file pone.0314831.s013.zip › S3 File/voting1_model_calibration_curve.png]

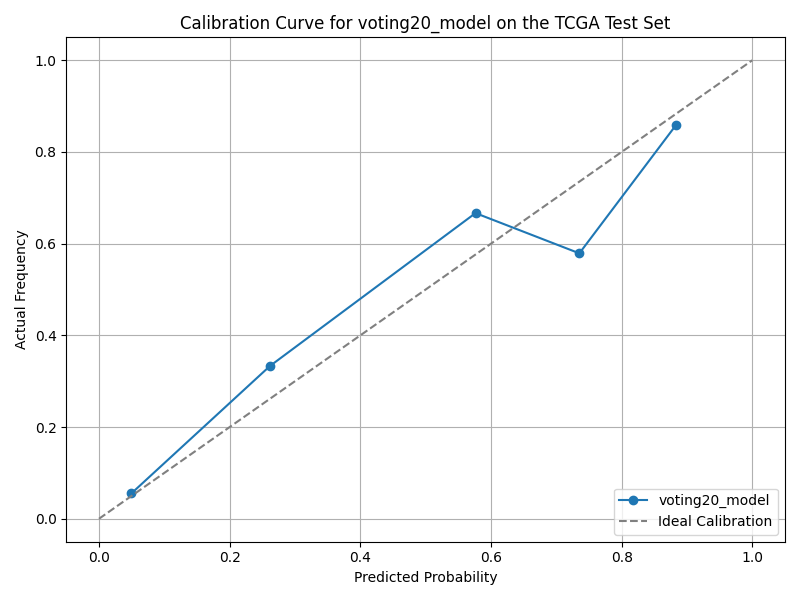

Supplement: S3 File — (ZIP) [file pone.0314831.s013.zip › S3 File/voting20_model_calibration_curve.png]

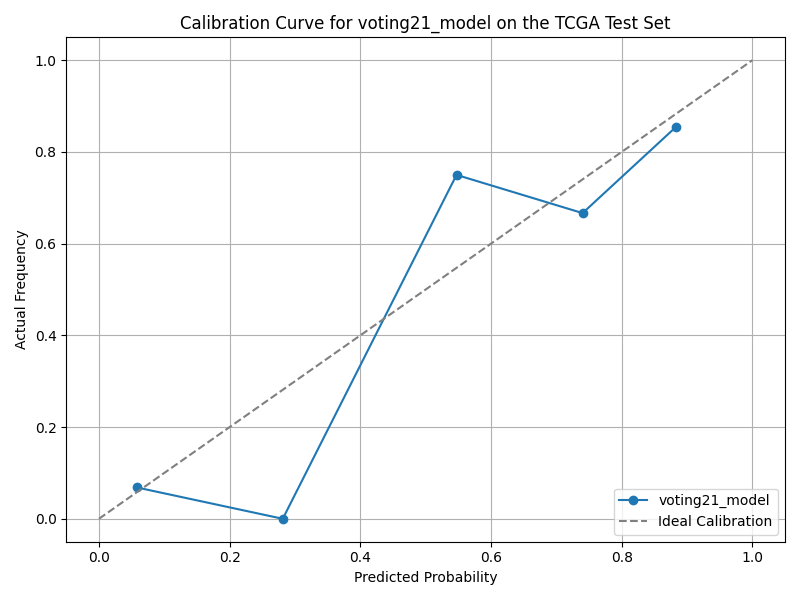

Supplement: S3 File — (ZIP) [file pone.0314831.s013.zip › S3 File/voting21_model_calibration_curve.png]

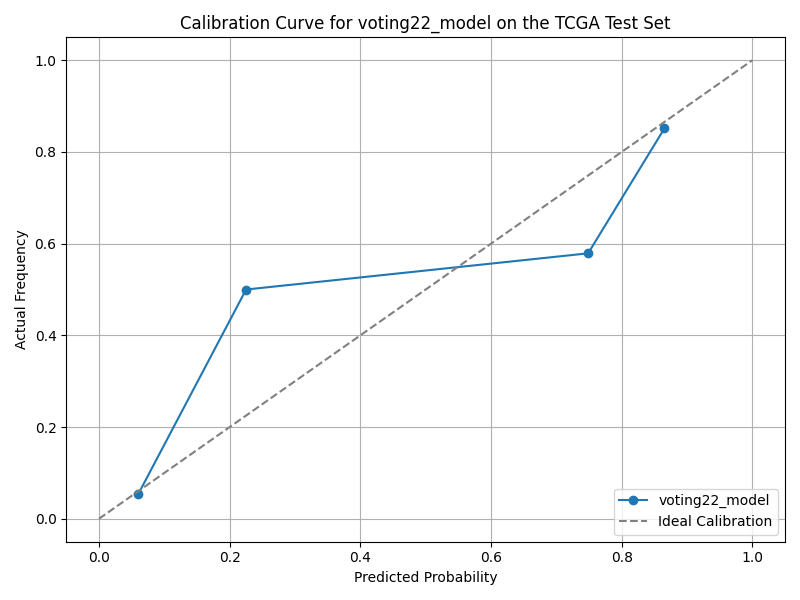

Supplement: S3 File — (ZIP) [file pone.0314831.s013.zip › S3 File/voting22_model_calibration_curve.png]

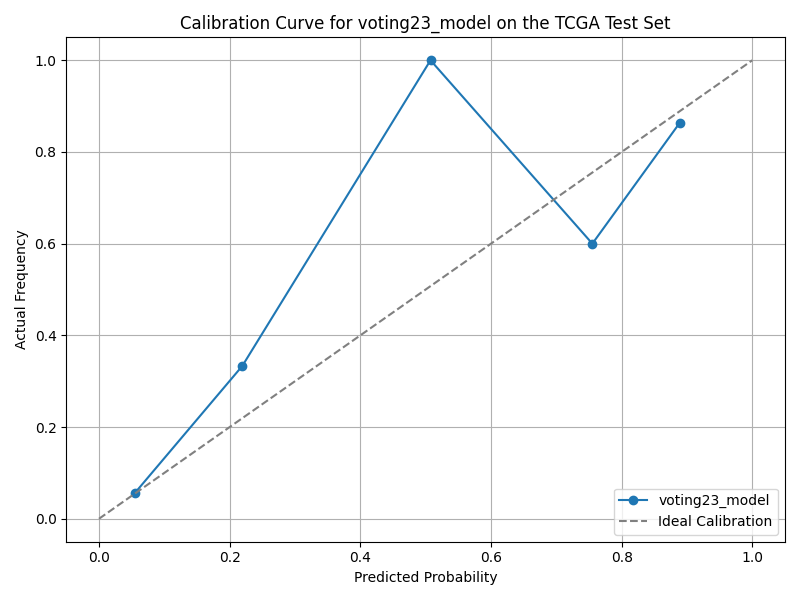

Supplement: S3 File — (ZIP) [file pone.0314831.s013.zip › S3 File/voting23_model_calibration_curve.png]

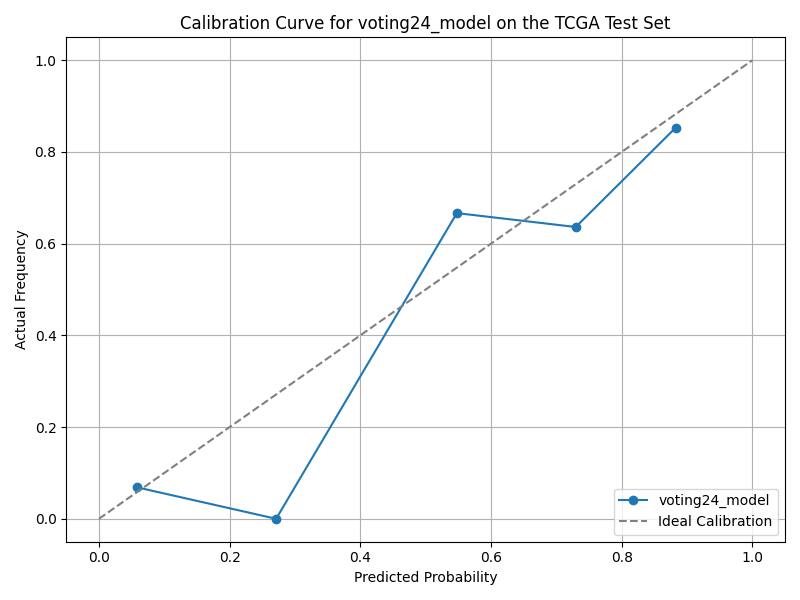

Supplement: S3 File — (ZIP) [file pone.0314831.s013.zip › S3 File/voting24_model_calibration_curve.png]

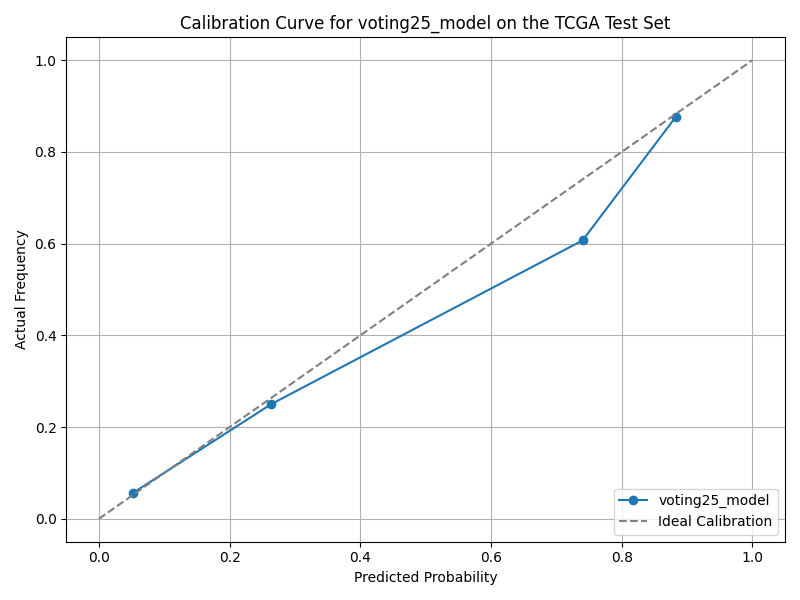

Supplement: S3 File — (ZIP) [file pone.0314831.s013.zip › S3 File/voting25_model_calibration_curve.png]

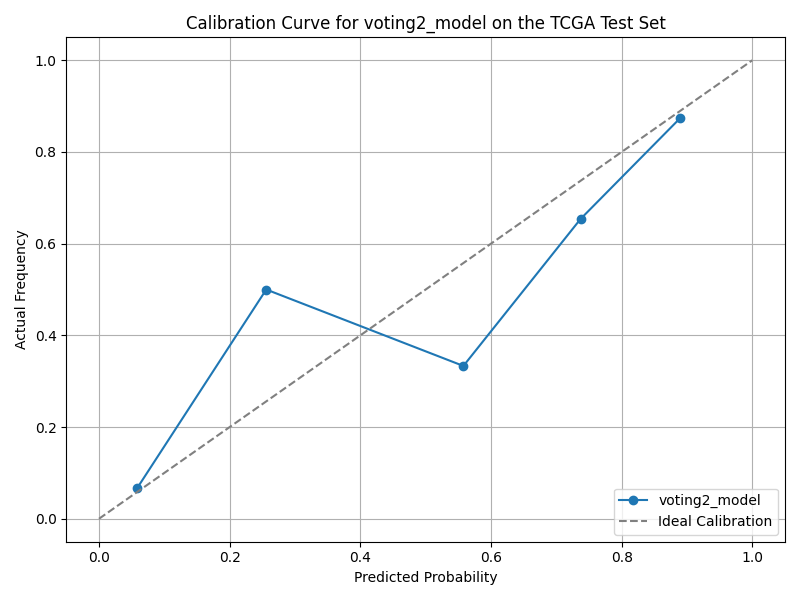

Supplement: S3 File — (ZIP) [file pone.0314831.s013.zip › S3 File/voting2_model_calibration_curve.png]

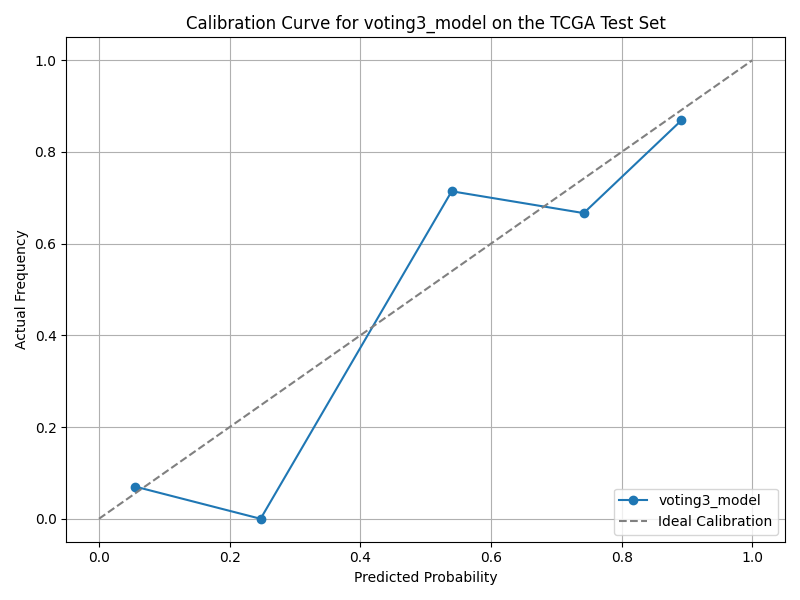

Supplement: S3 File — (ZIP) [file pone.0314831.s013.zip › S3 File/voting3_model_calibration_curve.png]

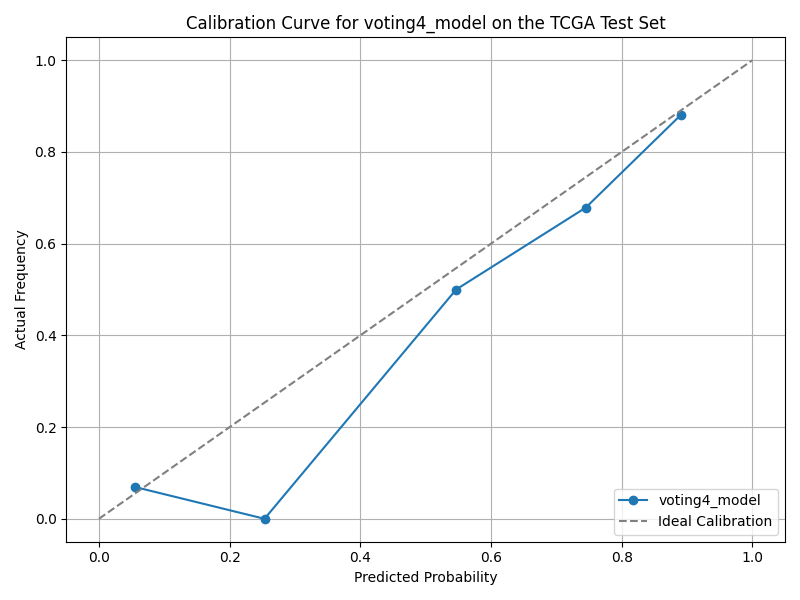

Supplement: S3 File — (ZIP) [file pone.0314831.s013.zip › S3 File/voting4_model_calibration_curve.png]

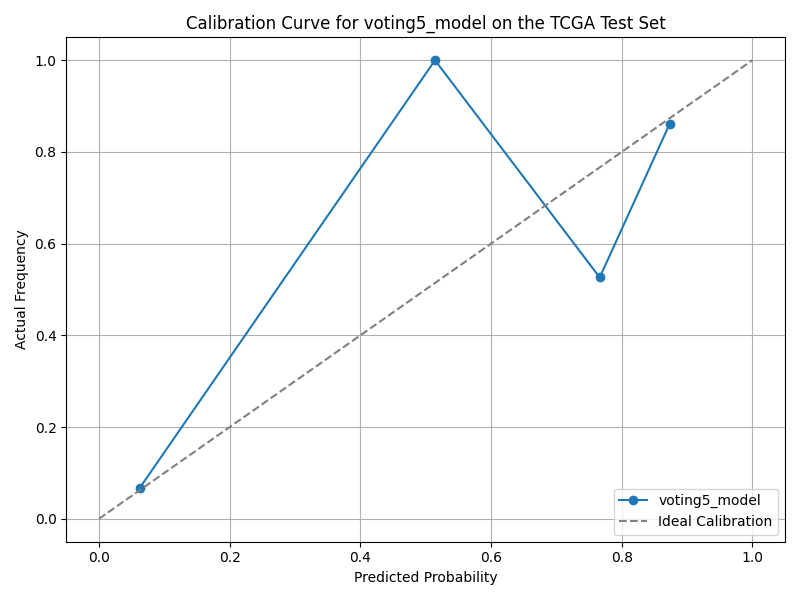

Supplement: S3 File — (ZIP) [file pone.0314831.s013.zip › S3 File/voting5_model_calibration_curve.png]

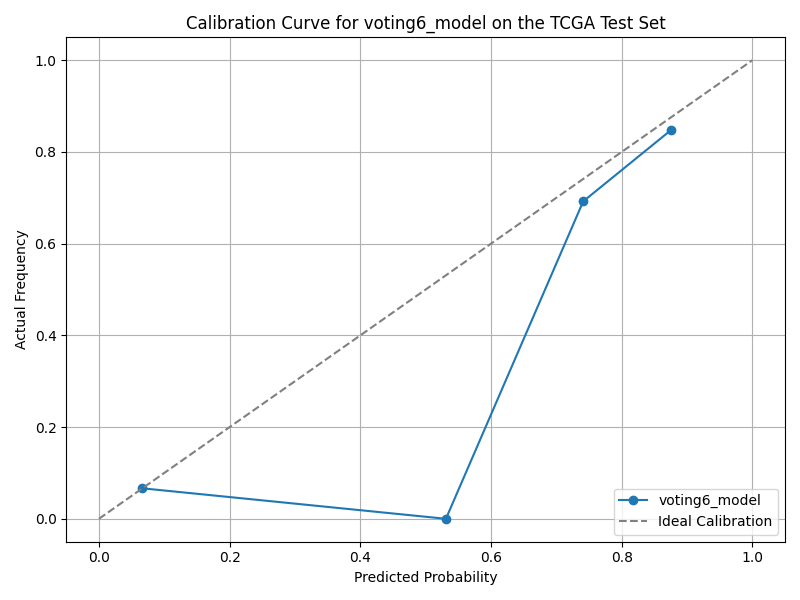

Supplement: S3 File — (ZIP) [file pone.0314831.s013.zip › S3 File/voting6_model_calibration_curve.png]

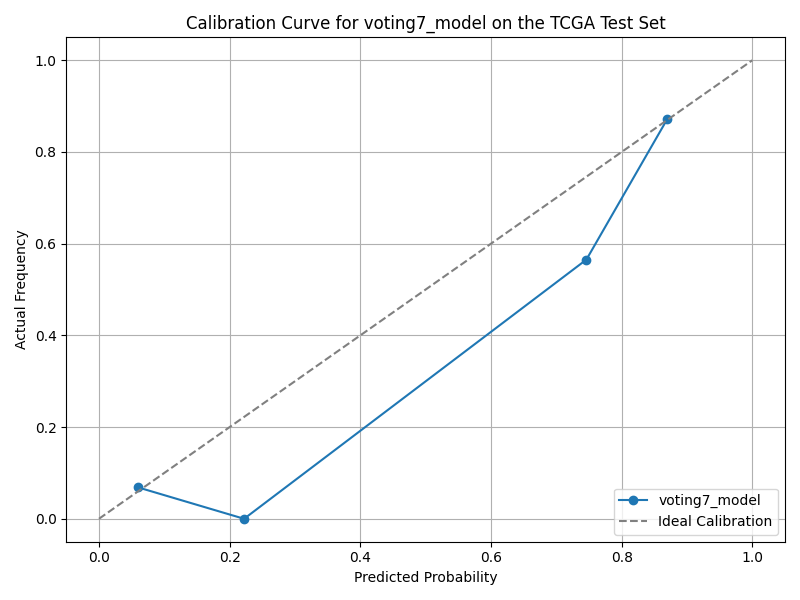

Supplement: S3 File — (ZIP) [file pone.0314831.s013.zip › S3 File/voting7_model_calibration_curve.png]

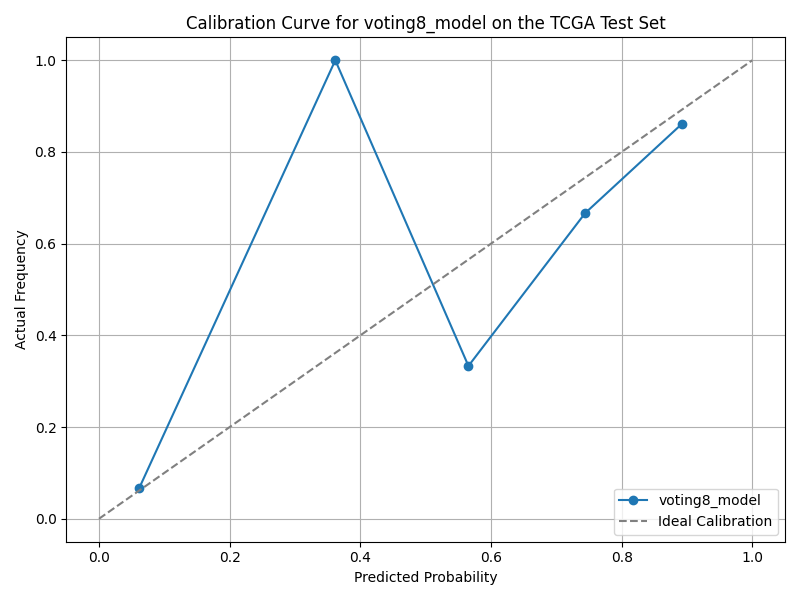

Supplement: S3 File — (ZIP) [file pone.0314831.s013.zip › S3 File/voting8_model_calibration_curve.png]

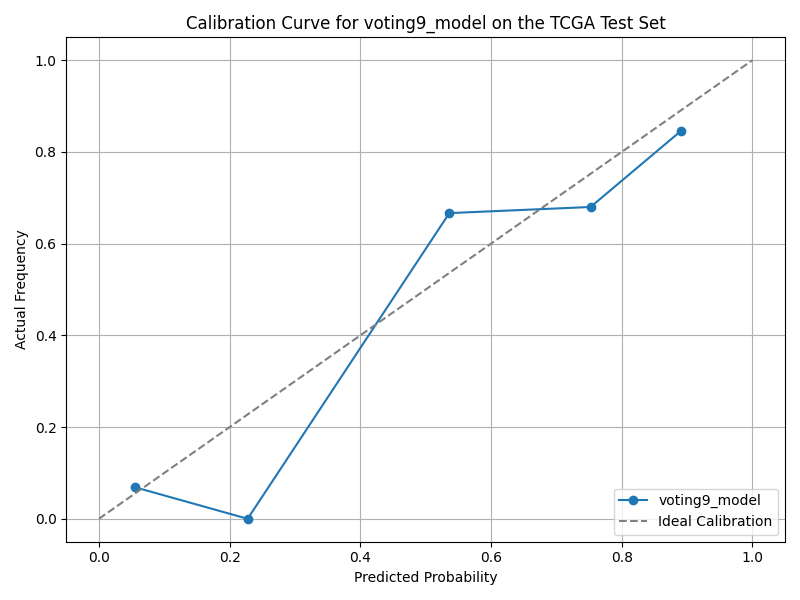

Supplement: S3 File — (ZIP) [file pone.0314831.s013.zip › S3 File/voting9_model_calibration_curve.png]

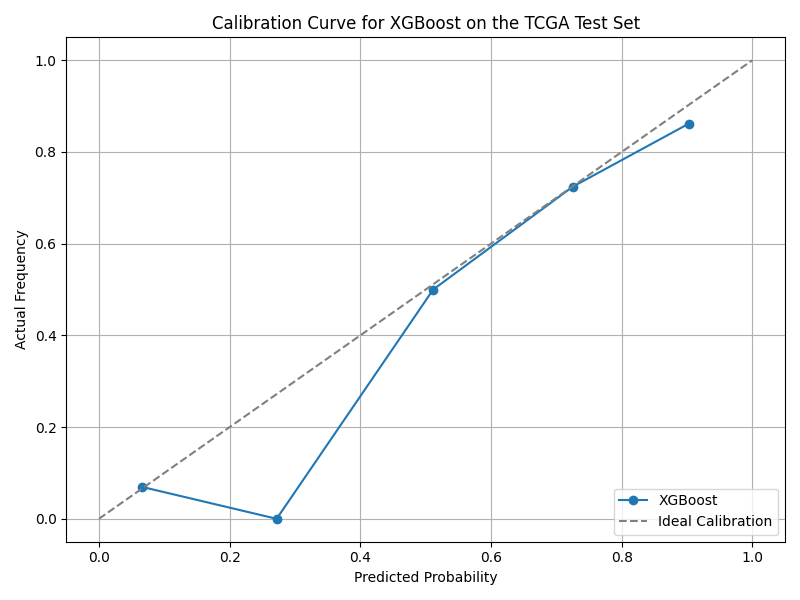

Supplement: S3 File — (ZIP) [file pone.0314831.s013.zip › S3 File/XGBoost_calibration_curve.png]

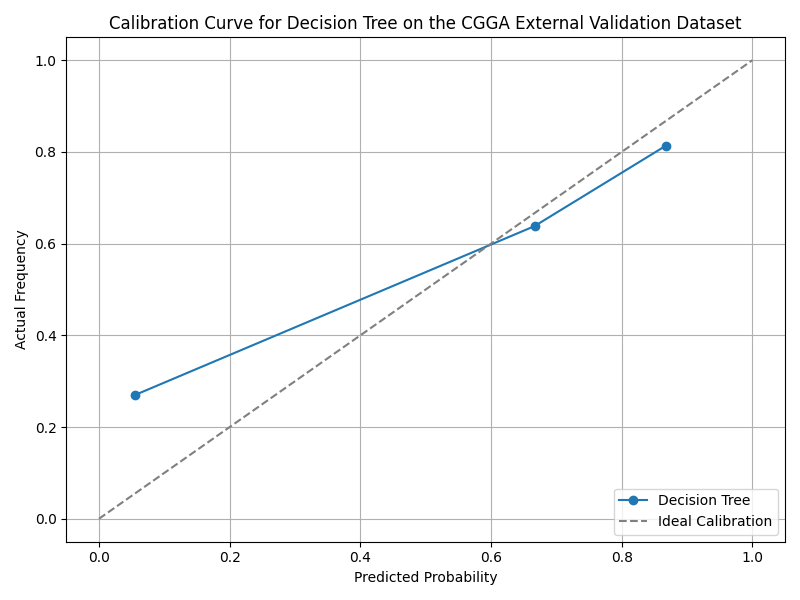

Supplement: S4 File — (ZIP) [file pone.0314831.s014.zip › S4 File/Decision Tree_calibration_curve.png]

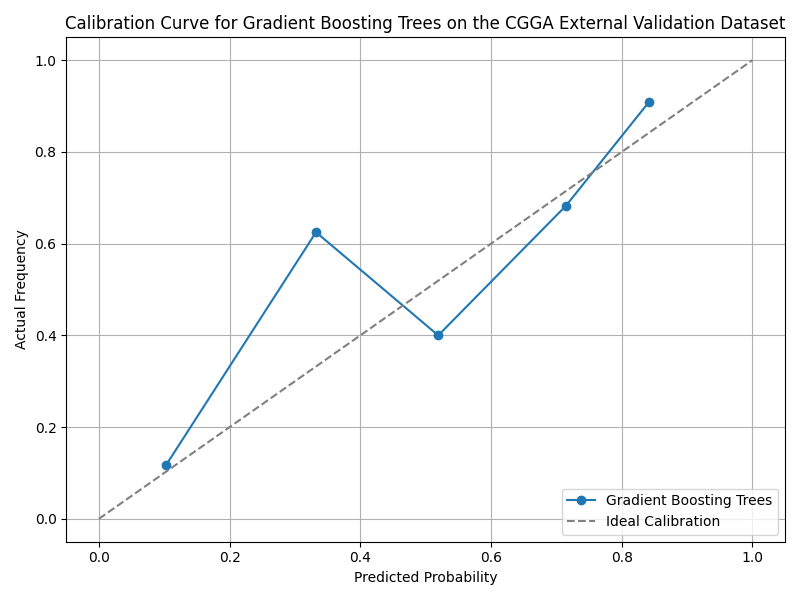

Supplement: S4 File — (ZIP) [file pone.0314831.s014.zip › S4 File/Gradient Boosting Trees_calibration_curve.png]

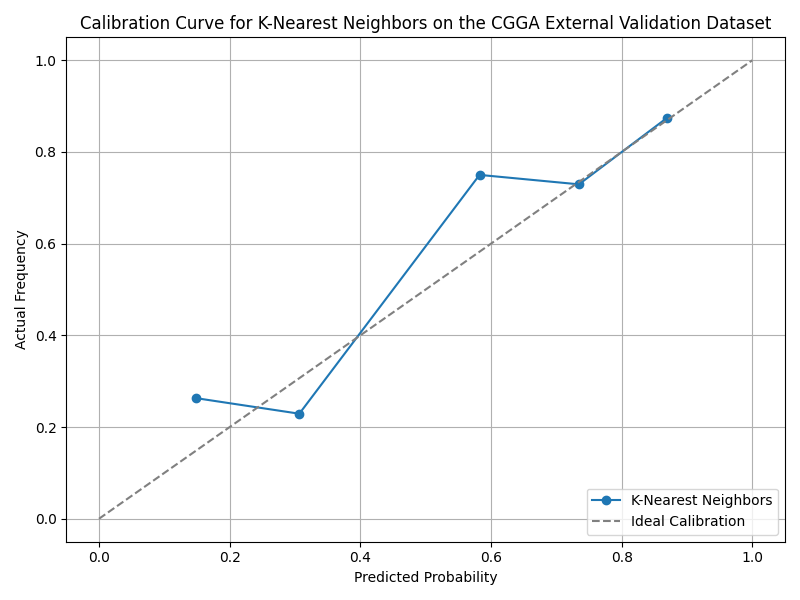

Supplement: S4 File — (ZIP) [file pone.0314831.s014.zip › S4 File/K-Nearest Neighbors_calibration_curve.png]

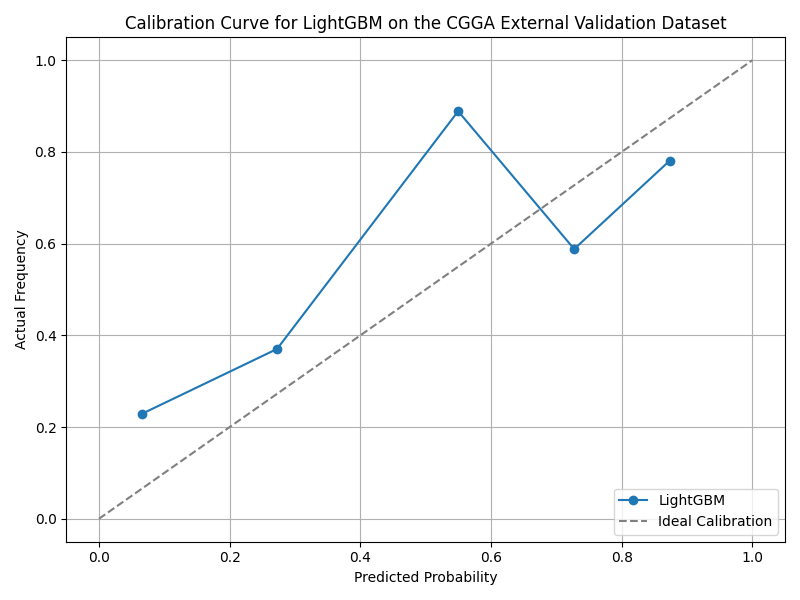

Supplement: S4 File — (ZIP) [file pone.0314831.s014.zip › S4 File/LightGBM_calibration_curve.png]

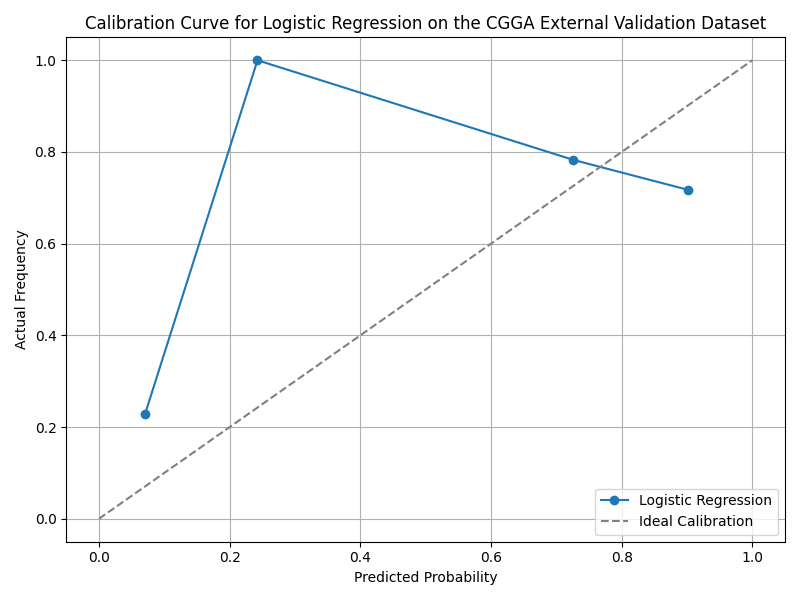

Supplement: S4 File — (ZIP) [file pone.0314831.s014.zip › S4 File/Logistic Regression_calibration_curve.png]

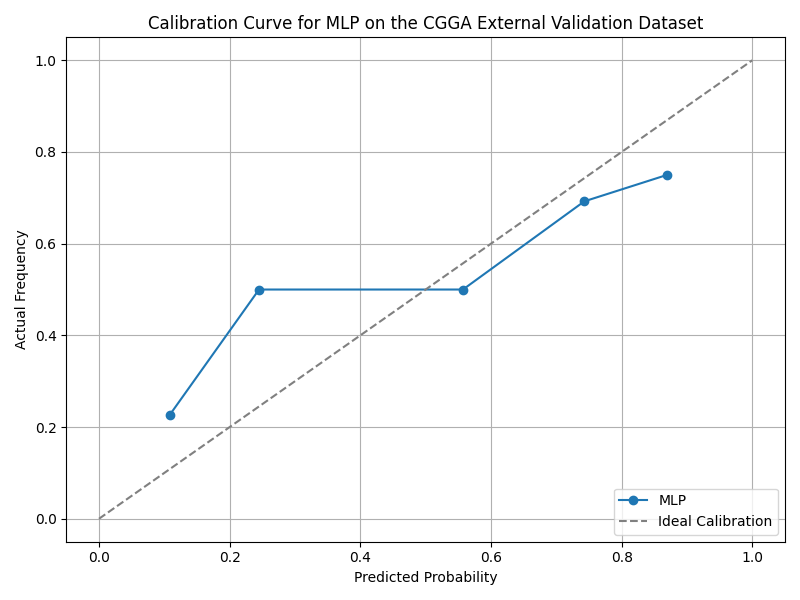

Supplement: S4 File — (ZIP) [file pone.0314831.s014.zip › S4 File/MLP_calibration_curve.png]

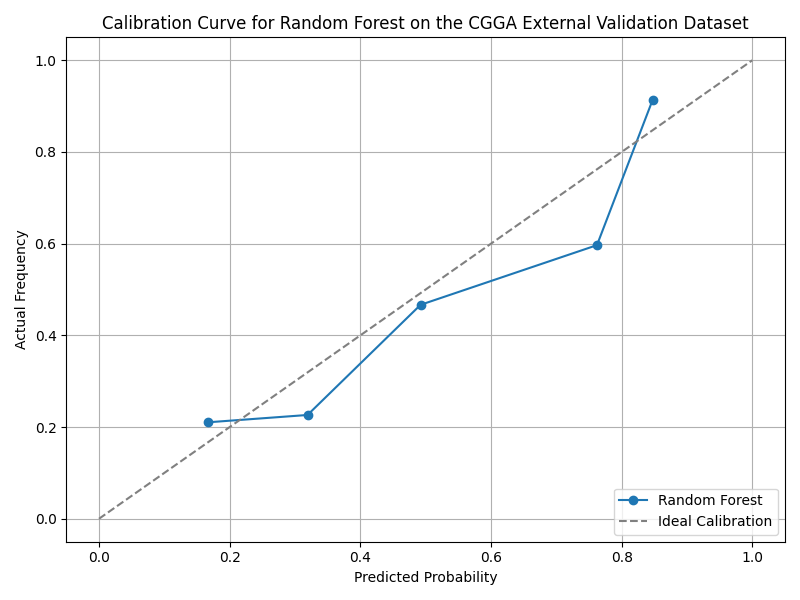

Supplement: S4 File — (ZIP) [file pone.0314831.s014.zip › S4 File/Random Forest_calibration_curve.png]

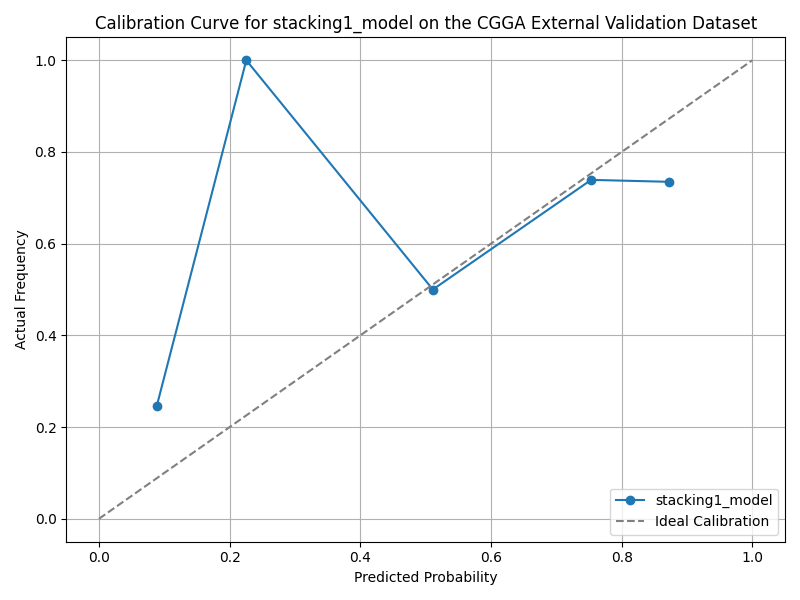

Supplement: S4 File — (ZIP) [file pone.0314831.s014.zip › S4 File/stacking1_model_calibration_curve.png]

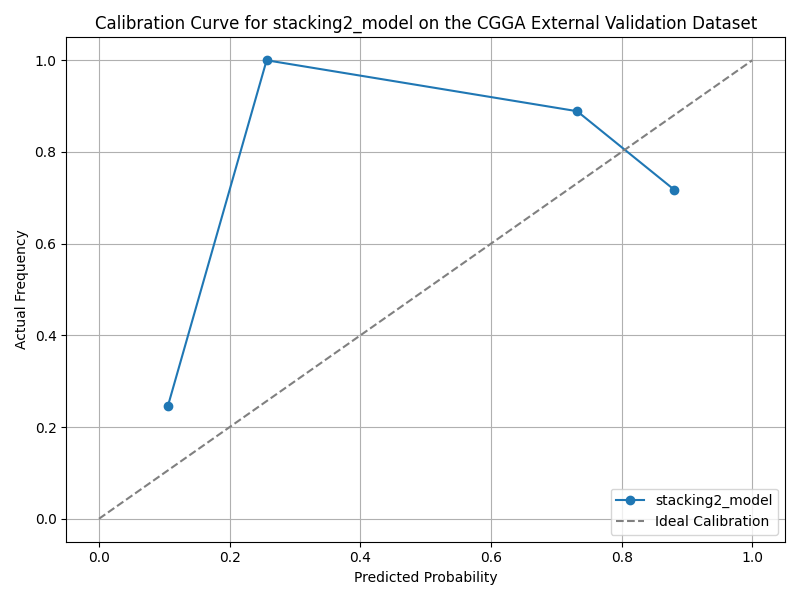

Supplement: S4 File — (ZIP) [file pone.0314831.s014.zip › S4 File/stacking2_model_calibration_curve.png]

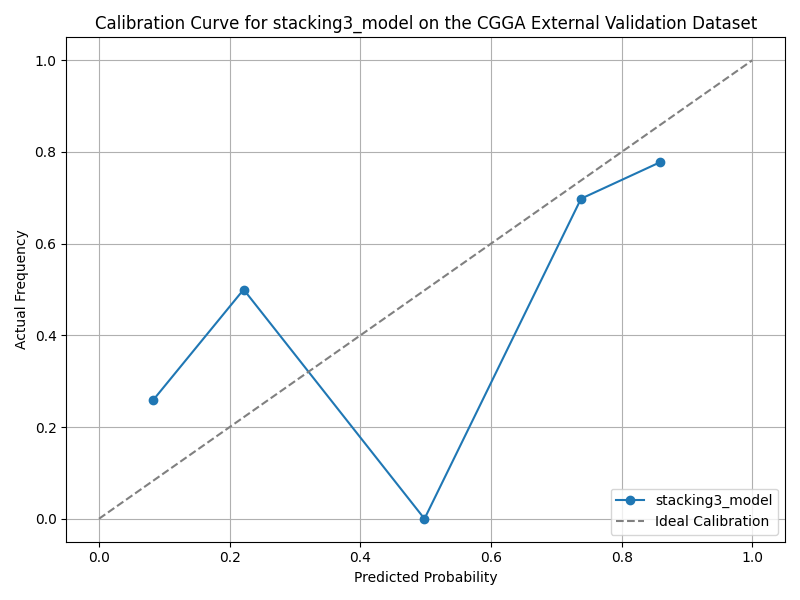

Supplement: S4 File — (ZIP) [file pone.0314831.s014.zip › S4 File/stacking3_model_calibration_curve.png]

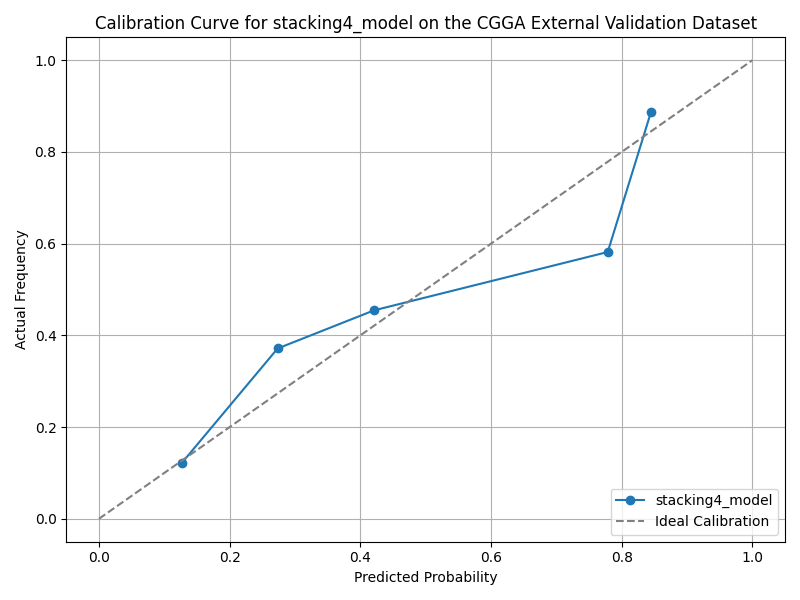

Supplement: S4 File — (ZIP) [file pone.0314831.s014.zip › S4 File/stacking4_model_calibration_curve.png]

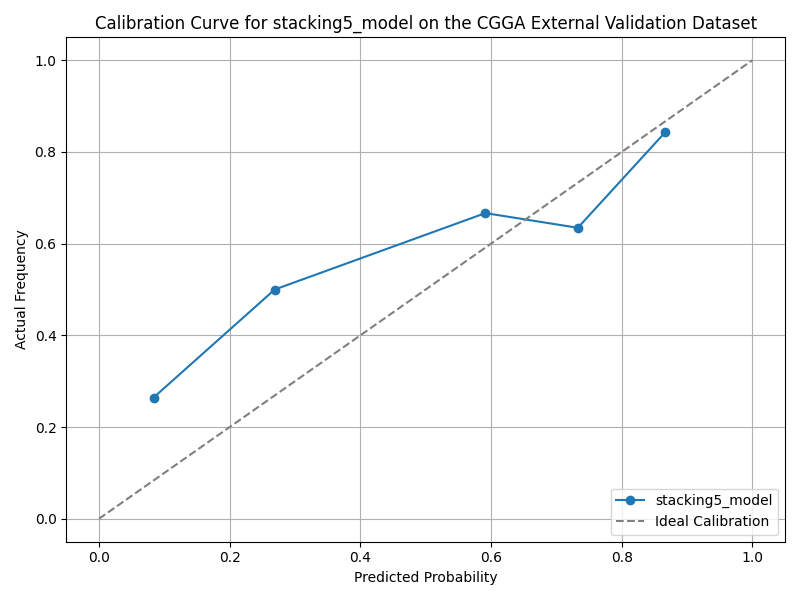

Supplement: S4 File — (ZIP) [file pone.0314831.s014.zip › S4 File/stacking5_model_calibration_curve.png]

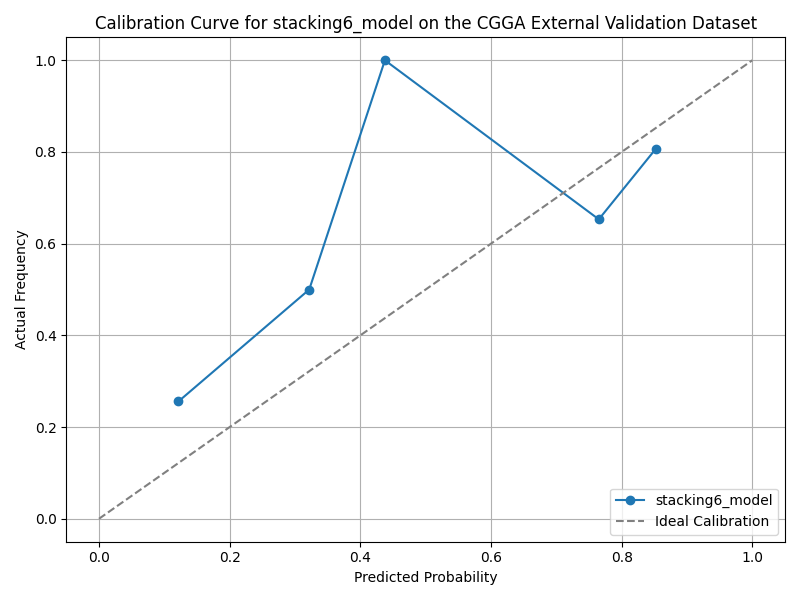

Supplement: S4 File — (ZIP) [file pone.0314831.s014.zip › S4 File/stacking6_model_calibration_curve.png]

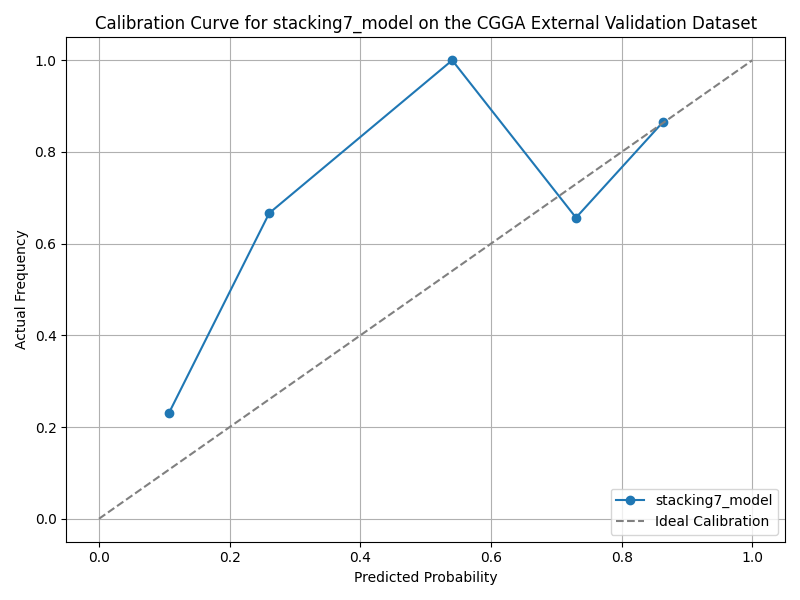

Supplement: S4 File — (ZIP) [file pone.0314831.s014.zip › S4 File/stacking7_model_calibration_curve.png]

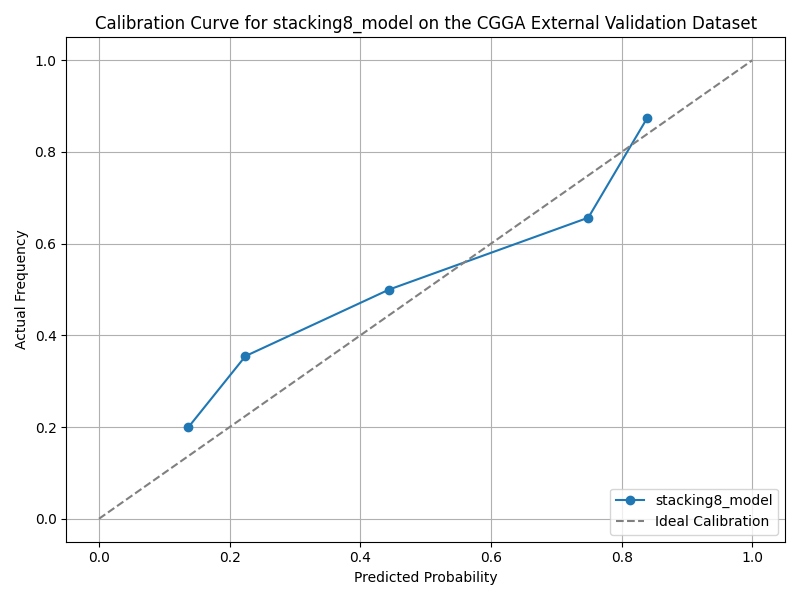

Supplement: S4 File — (ZIP) [file pone.0314831.s014.zip › S4 File/stacking8_model_calibration_curve.png]

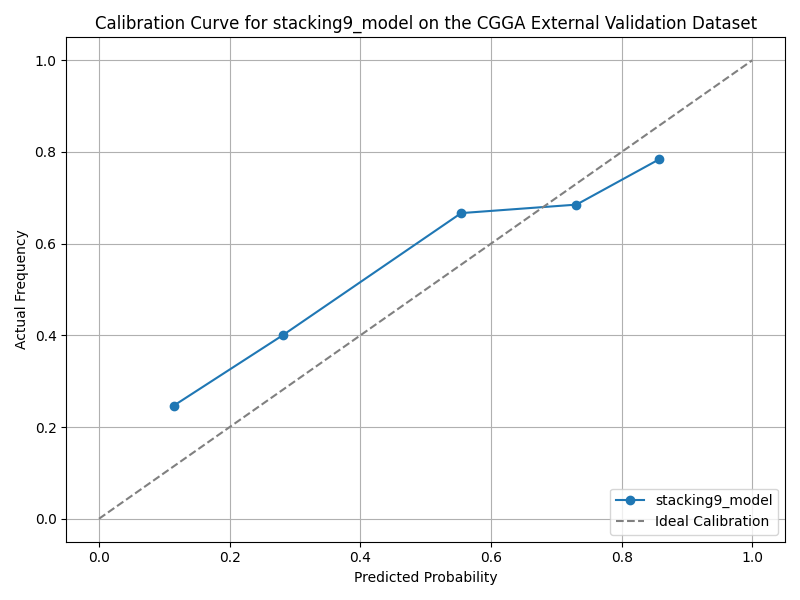

Supplement: S4 File — (ZIP) [file pone.0314831.s014.zip › S4 File/stacking9_model_calibration_curve.png]

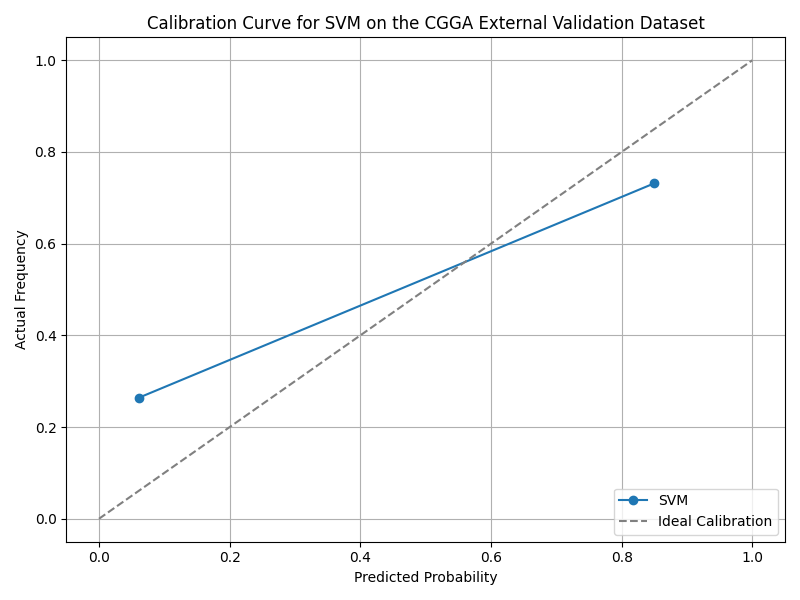

Supplement: S4 File — (ZIP) [file pone.0314831.s014.zip › S4 File/SVM_calibration_curve.png]

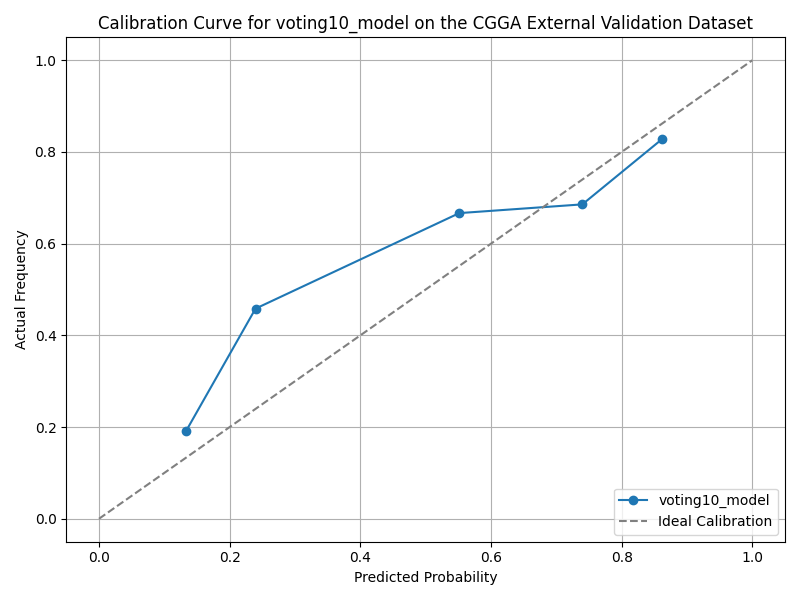

Supplement: S4 File — (ZIP) [file pone.0314831.s014.zip › S4 File/voting10_model_calibration_curve.png]

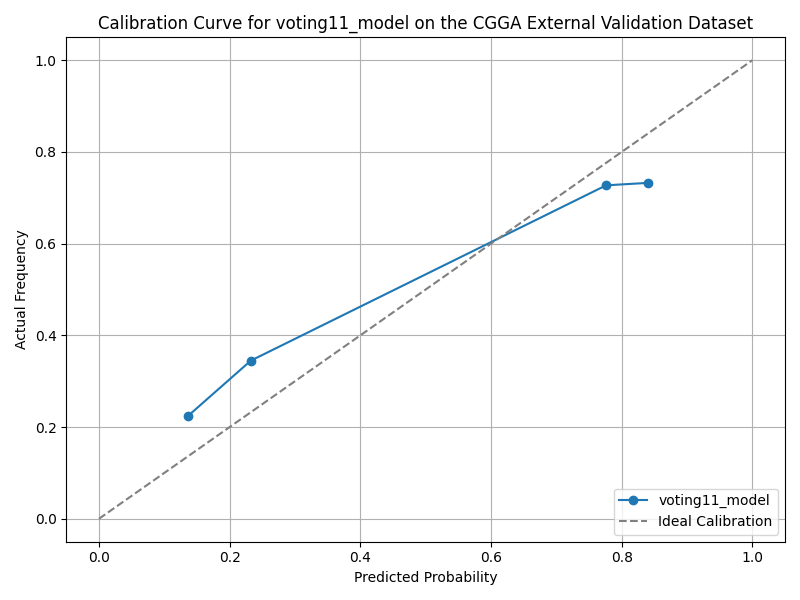

Supplement: S4 File — (ZIP) [file pone.0314831.s014.zip › S4 File/voting11_model_calibration_curve.png]

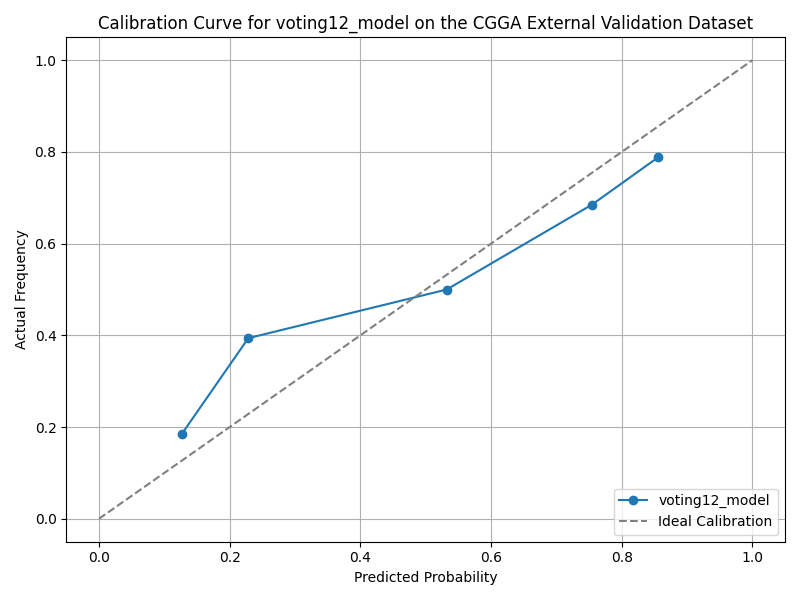

Supplement: S4 File — (ZIP) [file pone.0314831.s014.zip › S4 File/voting12_model_calibration_curve.png]

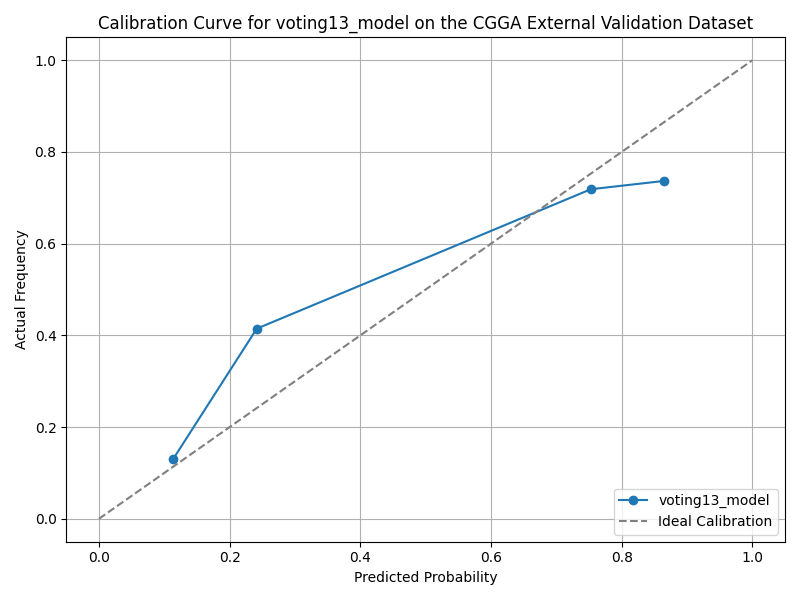

Supplement: S4 File — (ZIP) [file pone.0314831.s014.zip › S4 File/voting13_model_calibration_curve.png]

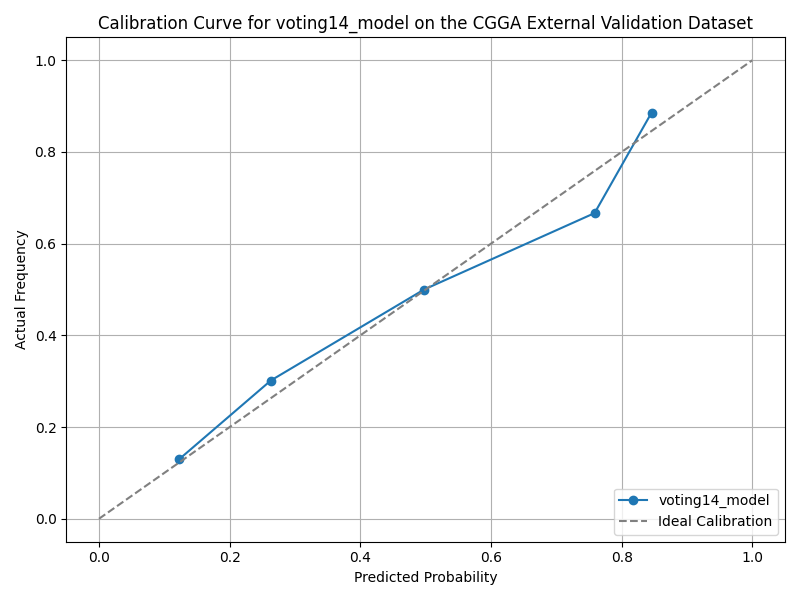

Supplement: S4 File — (ZIP) [file pone.0314831.s014.zip › S4 File/voting14_model_calibration_curve.png]

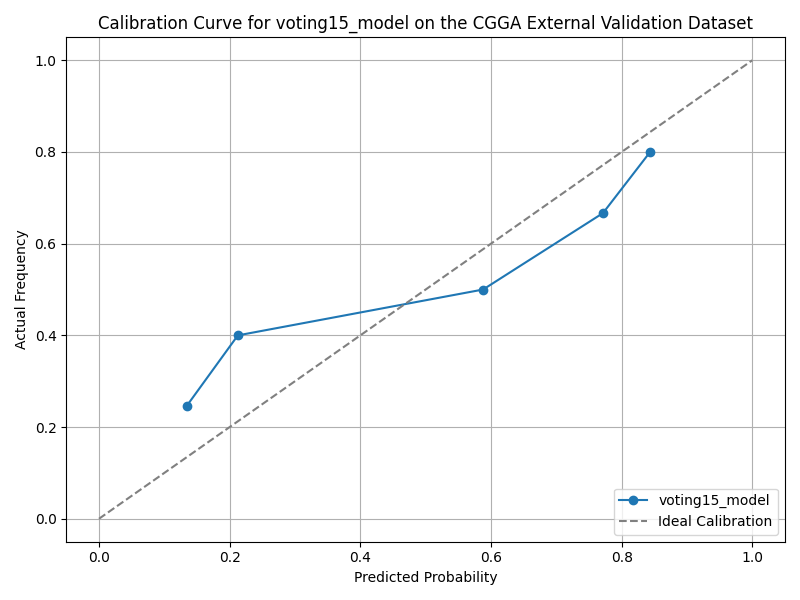

Supplement: S4 File — (ZIP) [file pone.0314831.s014.zip › S4 File/voting15_model_calibration_curve.png]

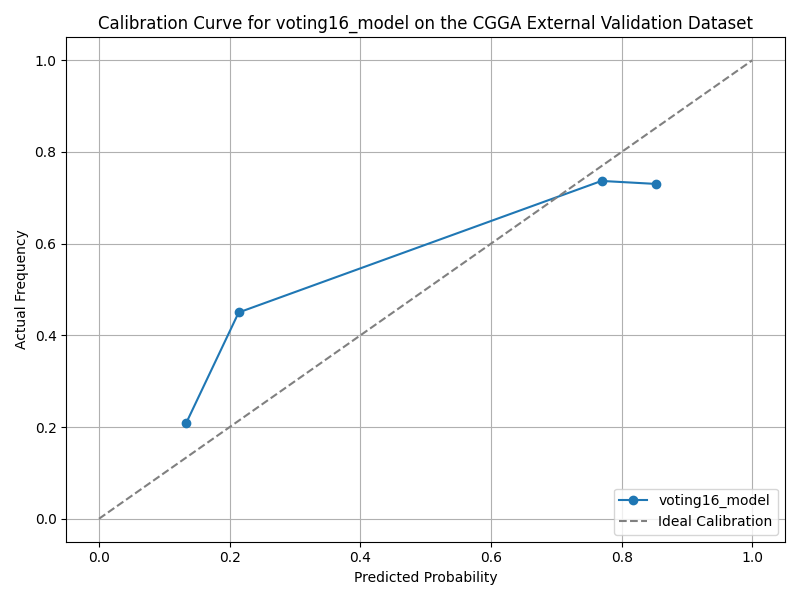

Supplement: S4 File — (ZIP) [file pone.0314831.s014.zip › S4 File/voting16_model_calibration_curve.png]

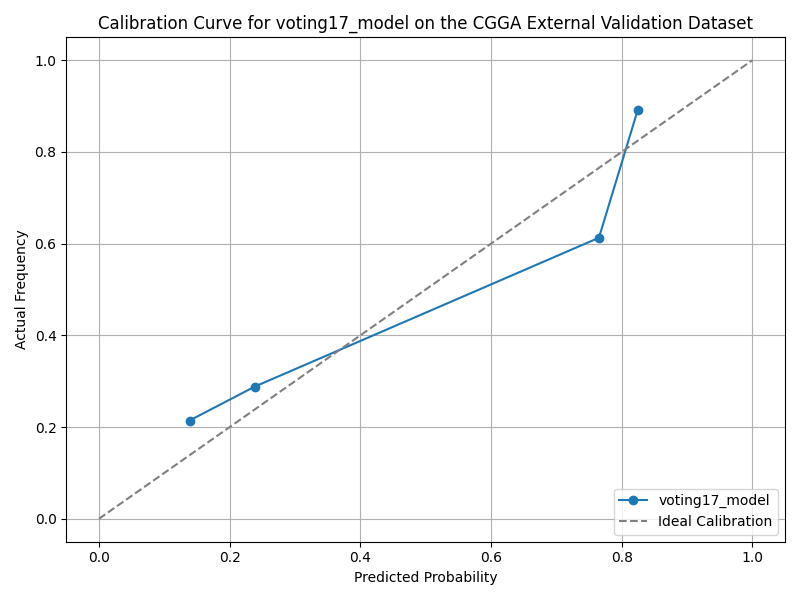

Supplement: S4 File — (ZIP) [file pone.0314831.s014.zip › S4 File/voting17_model_calibration_curve.png]

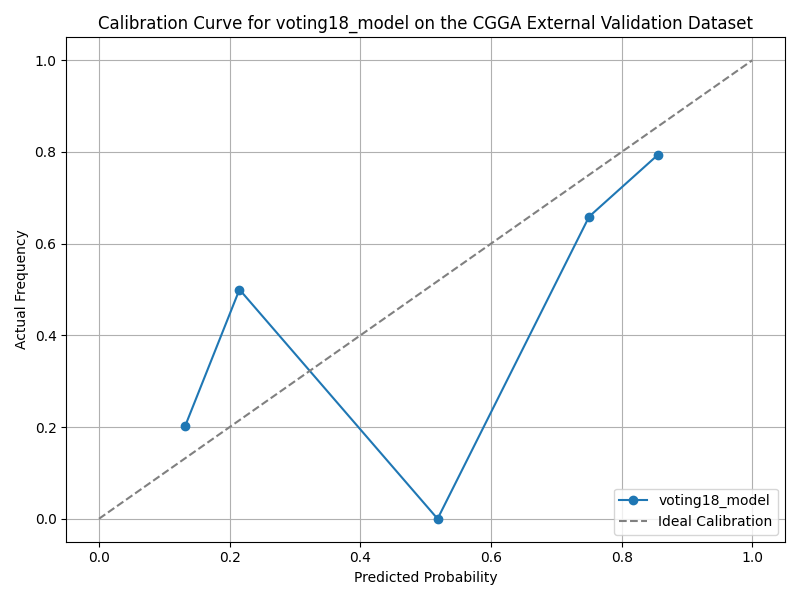

Supplement: S4 File — (ZIP) [file pone.0314831.s014.zip › S4 File/voting18_model_calibration_curve.png]

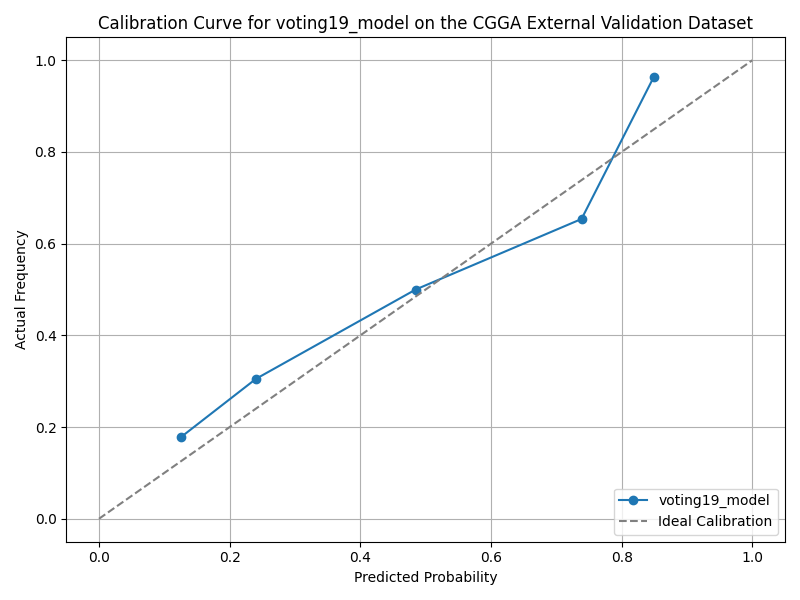

Supplement: S4 File — (ZIP) [file pone.0314831.s014.zip › S4 File/voting19_model_calibration_curve.png]

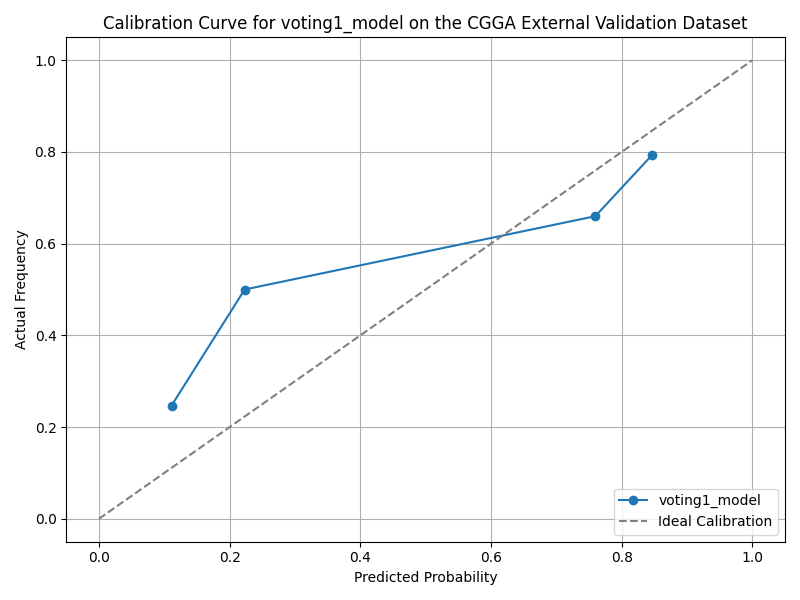

Supplement: S4 File — (ZIP) [file pone.0314831.s014.zip › S4 File/voting1_model_calibration_curve.png]

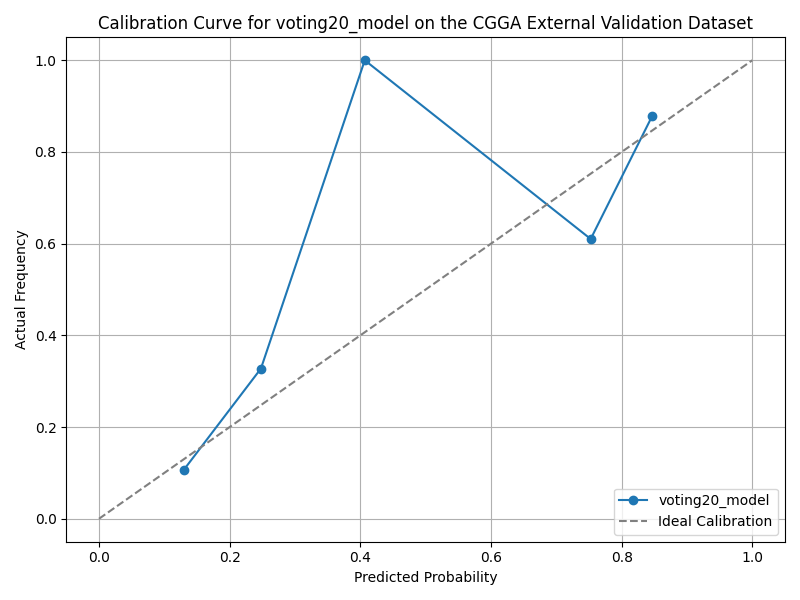

Supplement: S4 File — (ZIP) [file pone.0314831.s014.zip › S4 File/voting20_model_calibration_curve.png]

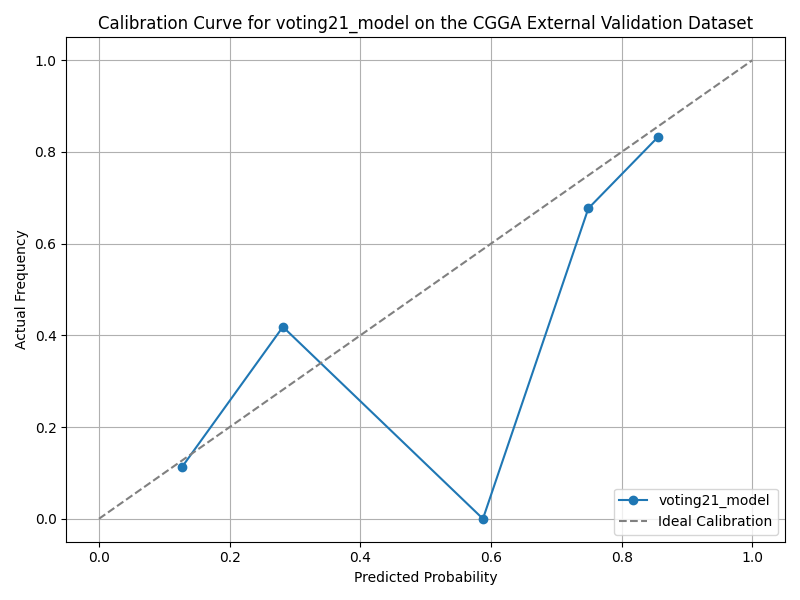

Supplement: S4 File — (ZIP) [file pone.0314831.s014.zip › S4 File/voting21_model_calibration_curve.png]

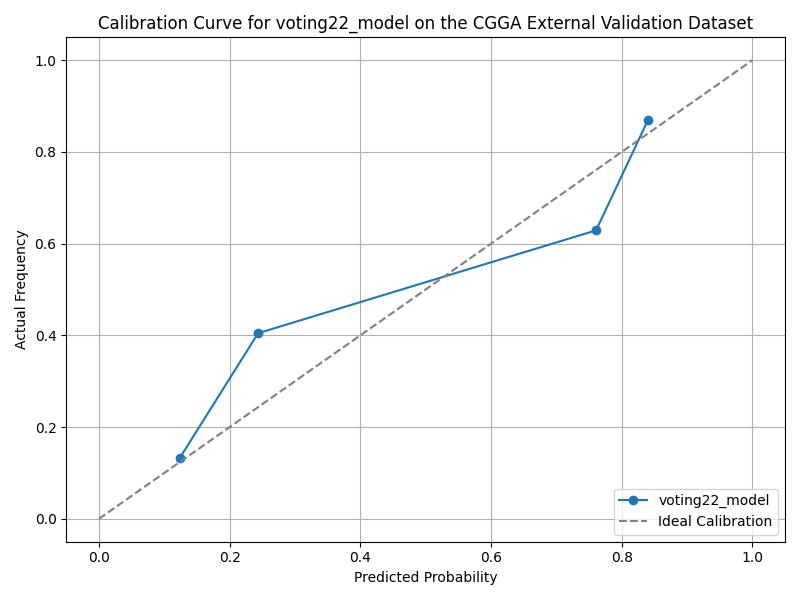

Supplement: S4 File — (ZIP) [file pone.0314831.s014.zip › S4 File/voting22_model_calibration_curve.png]

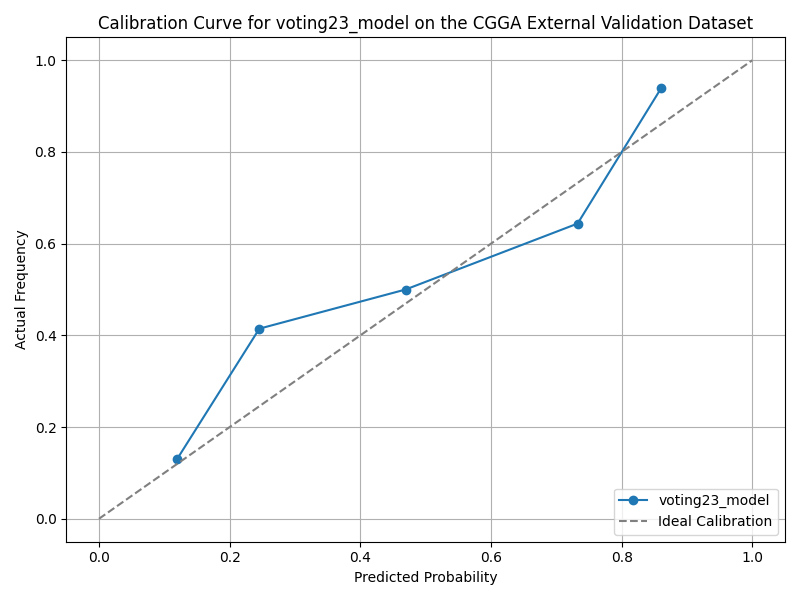

Supplement: S4 File — (ZIP) [file pone.0314831.s014.zip › S4 File/voting23_model_calibration_curve.png]

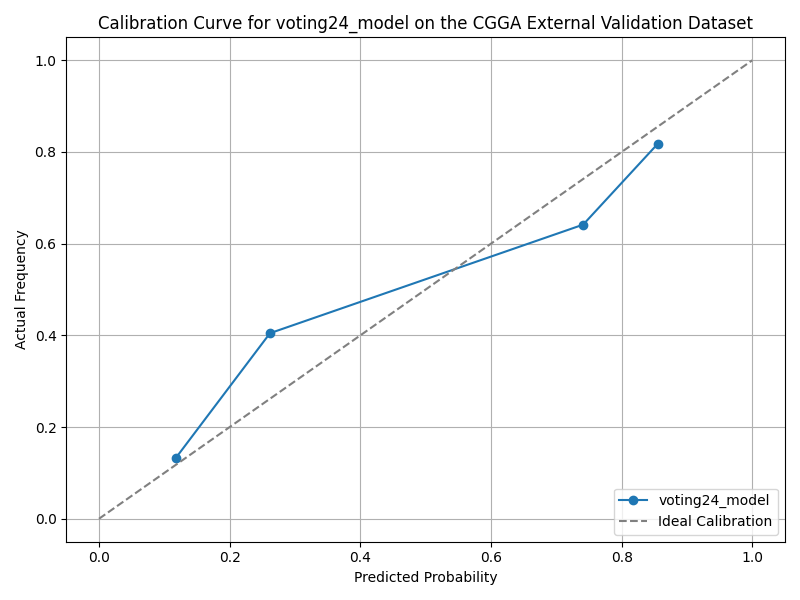

Supplement: S4 File — (ZIP) [file pone.0314831.s014.zip › S4 File/voting24_model_calibration_curve.png]

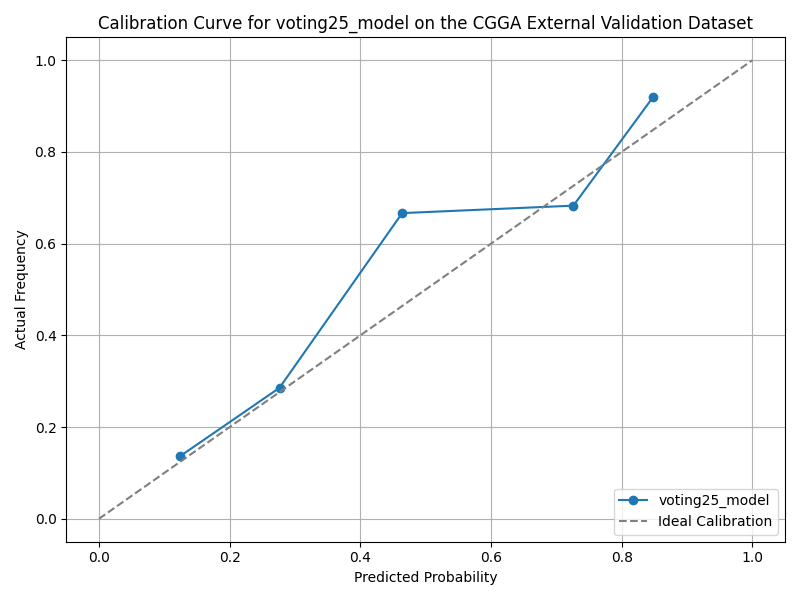

Supplement: S4 File — (ZIP) [file pone.0314831.s014.zip › S4 File/voting25_model_calibration_curve.png]

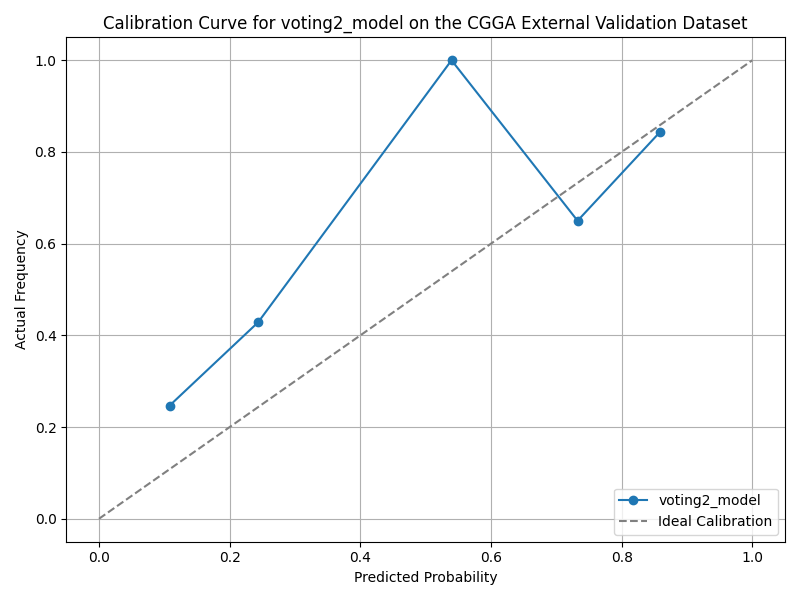

Supplement: S4 File — (ZIP) [file pone.0314831.s014.zip › S4 File/voting2_model_calibration_curve.png]

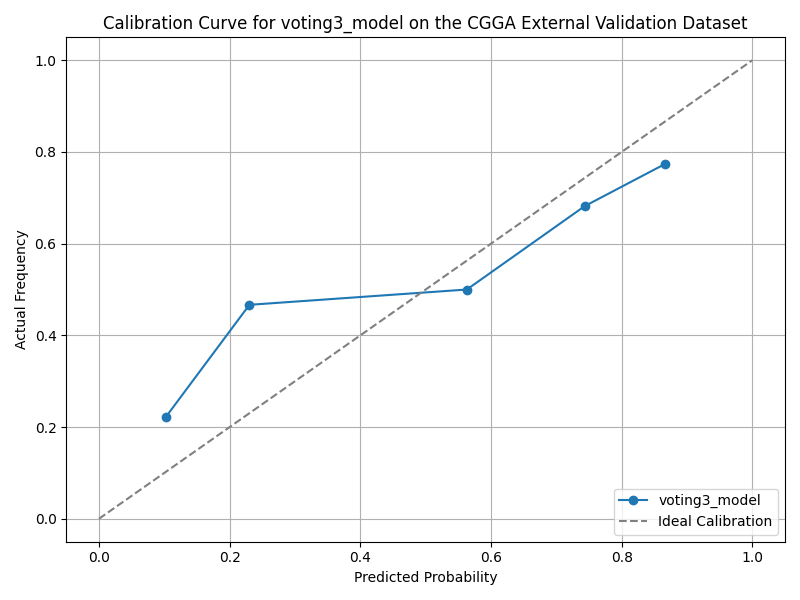

Supplement: S4 File — (ZIP) [file pone.0314831.s014.zip › S4 File/voting3_model_calibration_curve.png]

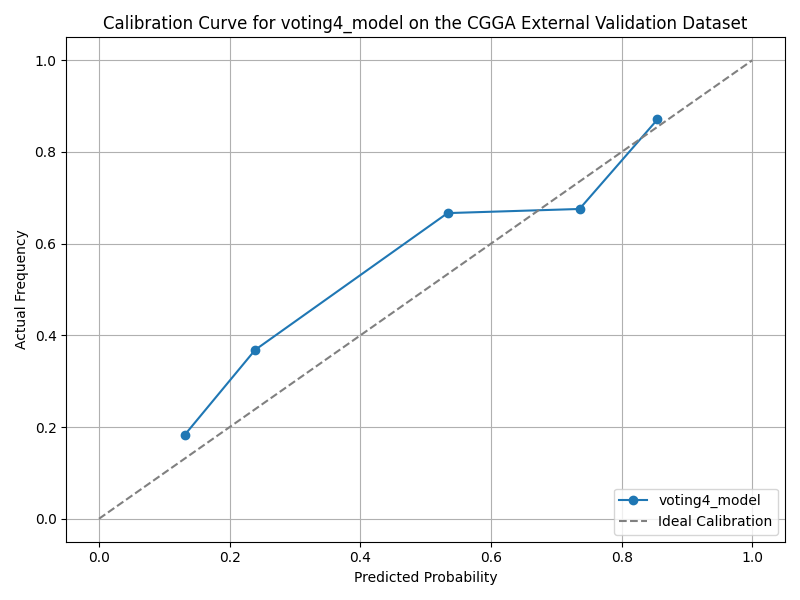

Supplement: S4 File — (ZIP) [file pone.0314831.s014.zip › S4 File/voting4_model_calibration_curve.png]

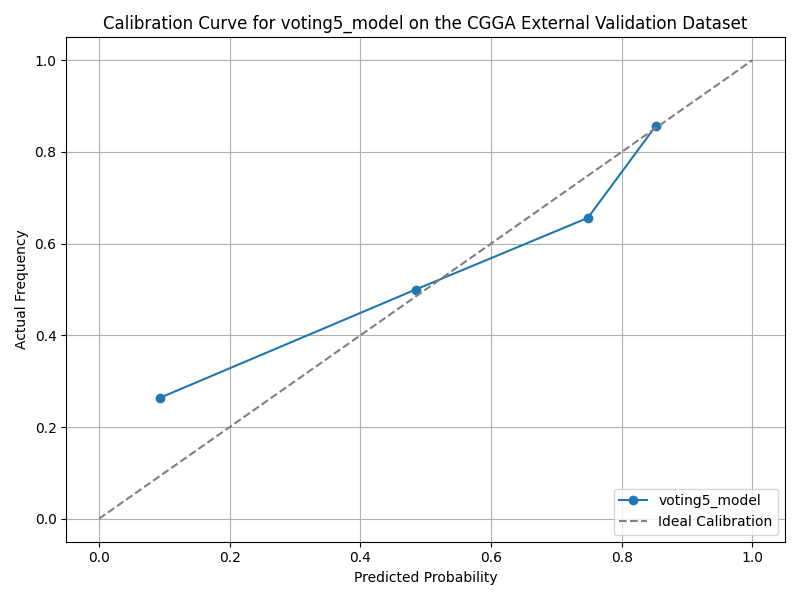

Supplement: S4 File — (ZIP) [file pone.0314831.s014.zip › S4 File/voting5_model_calibration_curve.png]

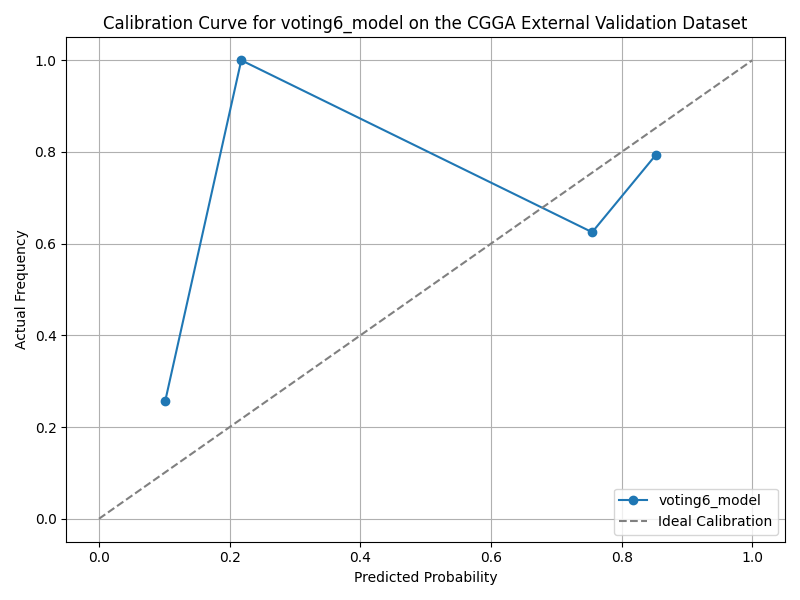

Supplement: S4 File — (ZIP) [file pone.0314831.s014.zip › S4 File/voting6_model_calibration_curve.png]

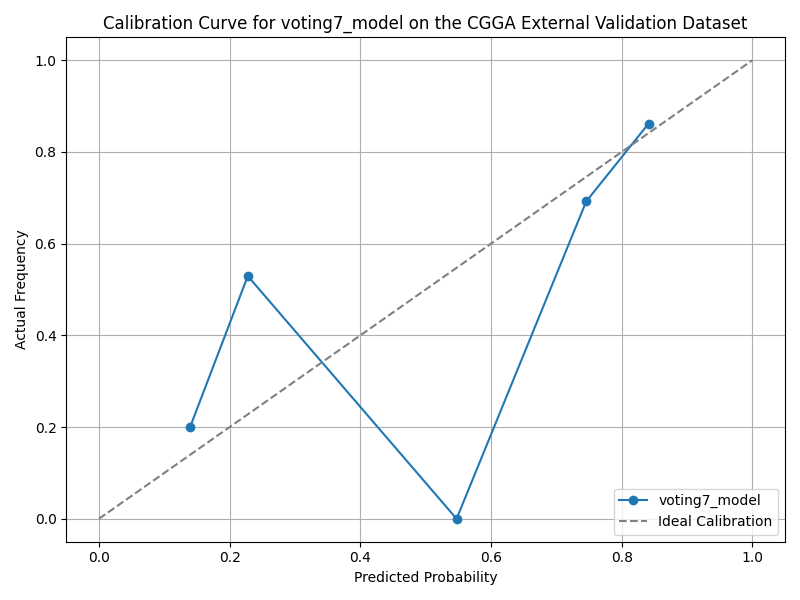

Supplement: S4 File — (ZIP) [file pone.0314831.s014.zip › S4 File/voting7_model_calibration_curve.png]

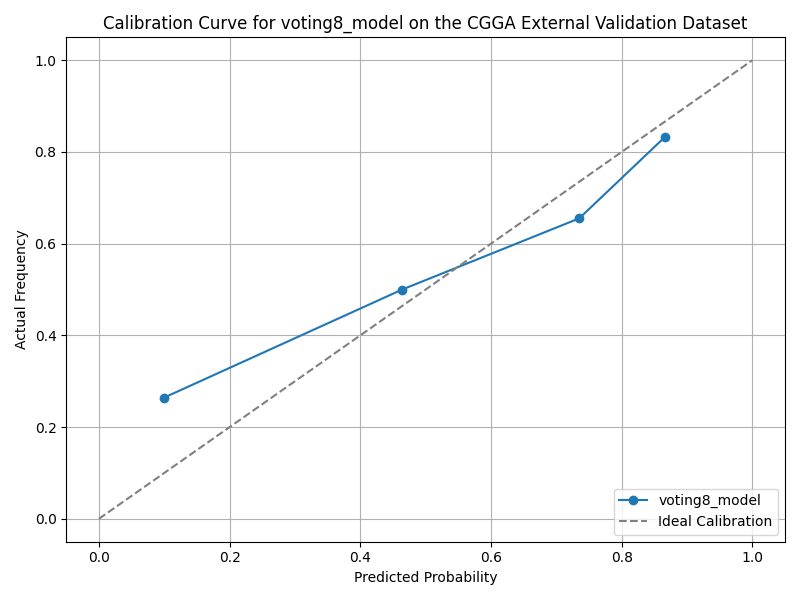

Supplement: S4 File — (ZIP) [file pone.0314831.s014.zip › S4 File/voting8_model_calibration_curve.png]

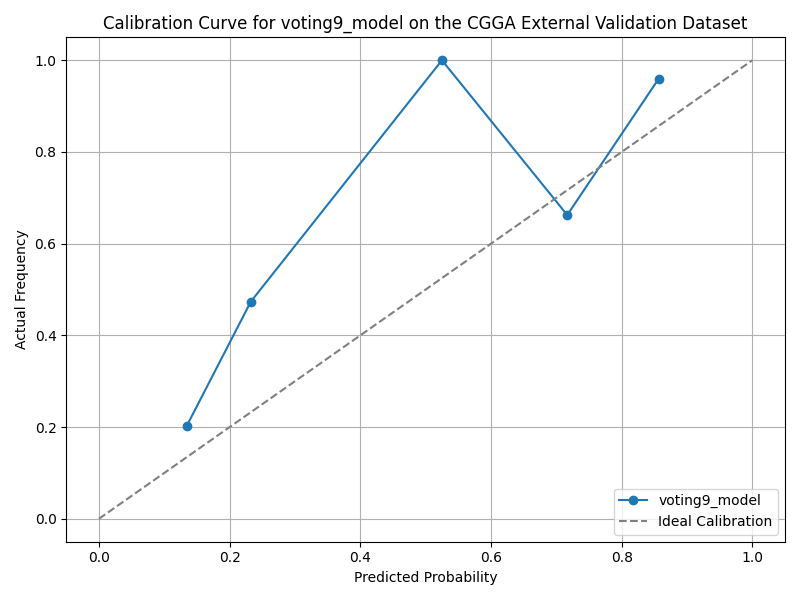

Supplement: S4 File — (ZIP) [file pone.0314831.s014.zip › S4 File/voting9_model_calibration_curve.png]

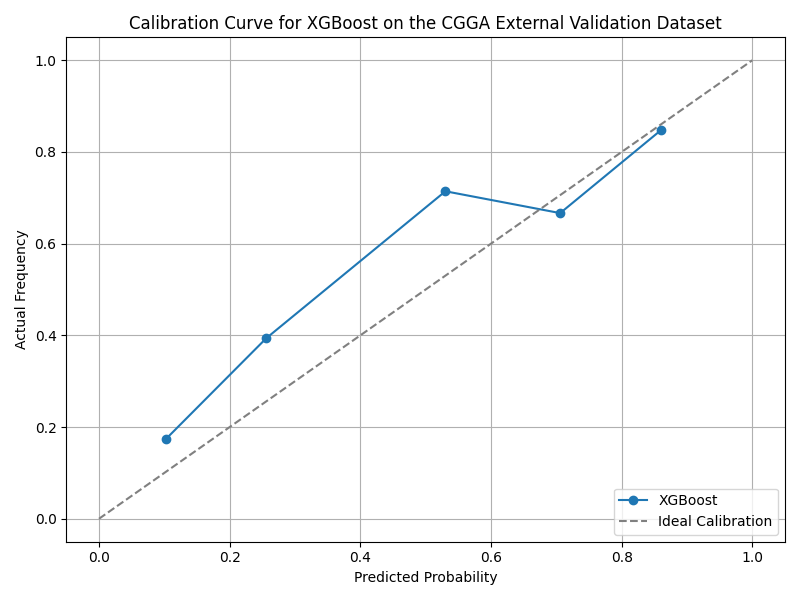

Supplement: S4 File — (ZIP) [file pone.0314831.s014.zip › S4 File/XGBoost_calibration_curve.png]

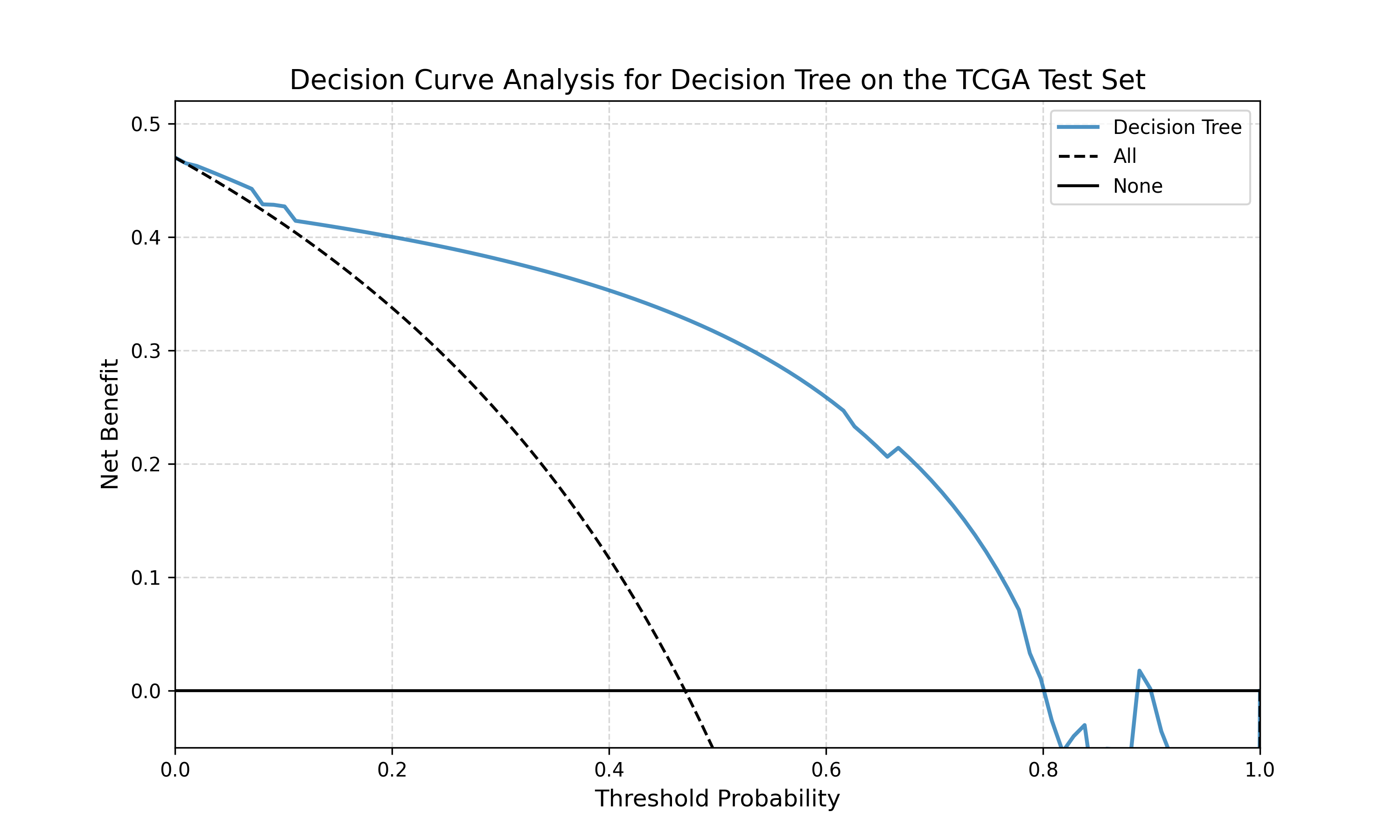

Supplement: S5 File — (ZIP) [file pone.0314831.s015.zip › S5 File/dca_curve_Decision Tree.png]

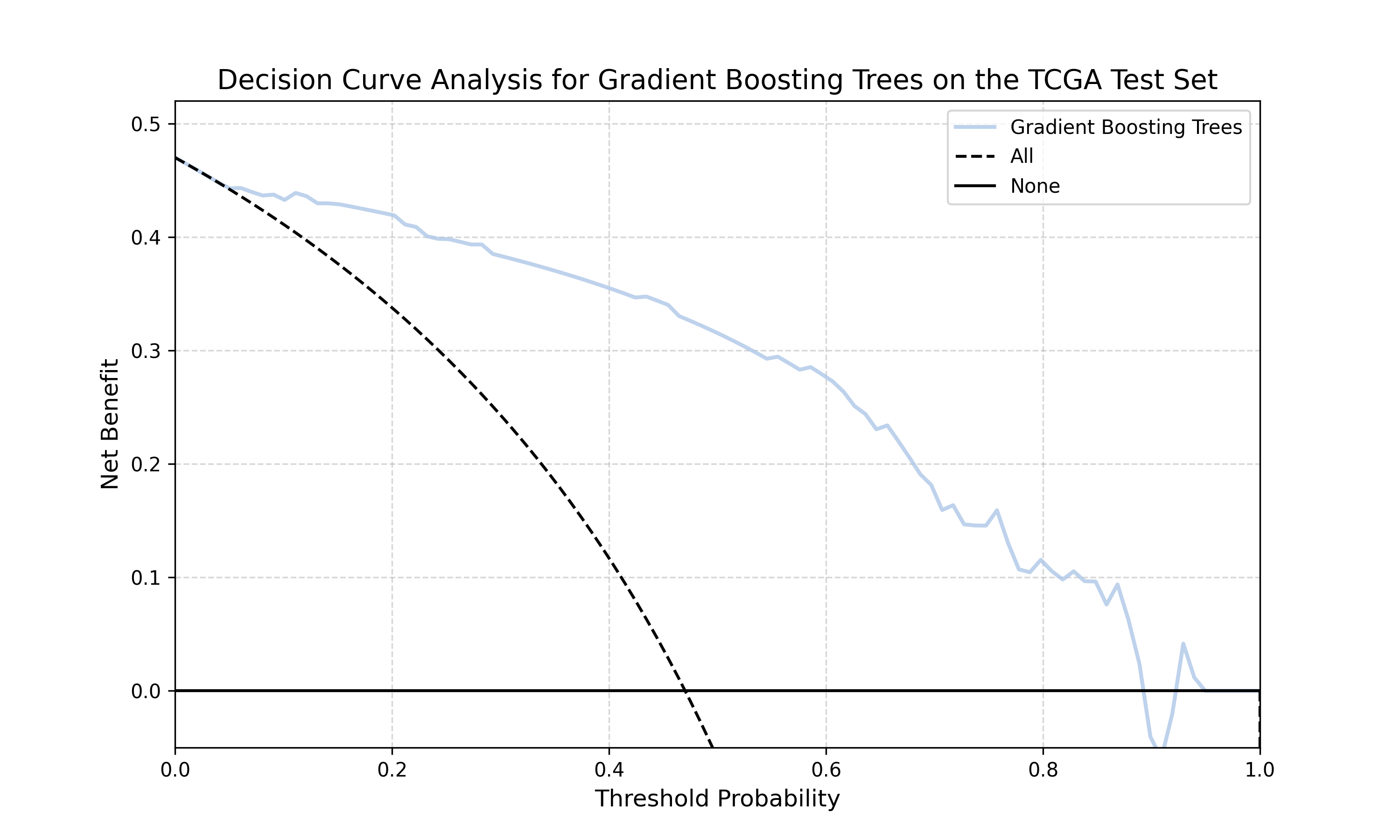

Supplement: S5 File — (ZIP) [file pone.0314831.s015.zip › S5 File/dca_curve_Gradient Boosting Trees.png]

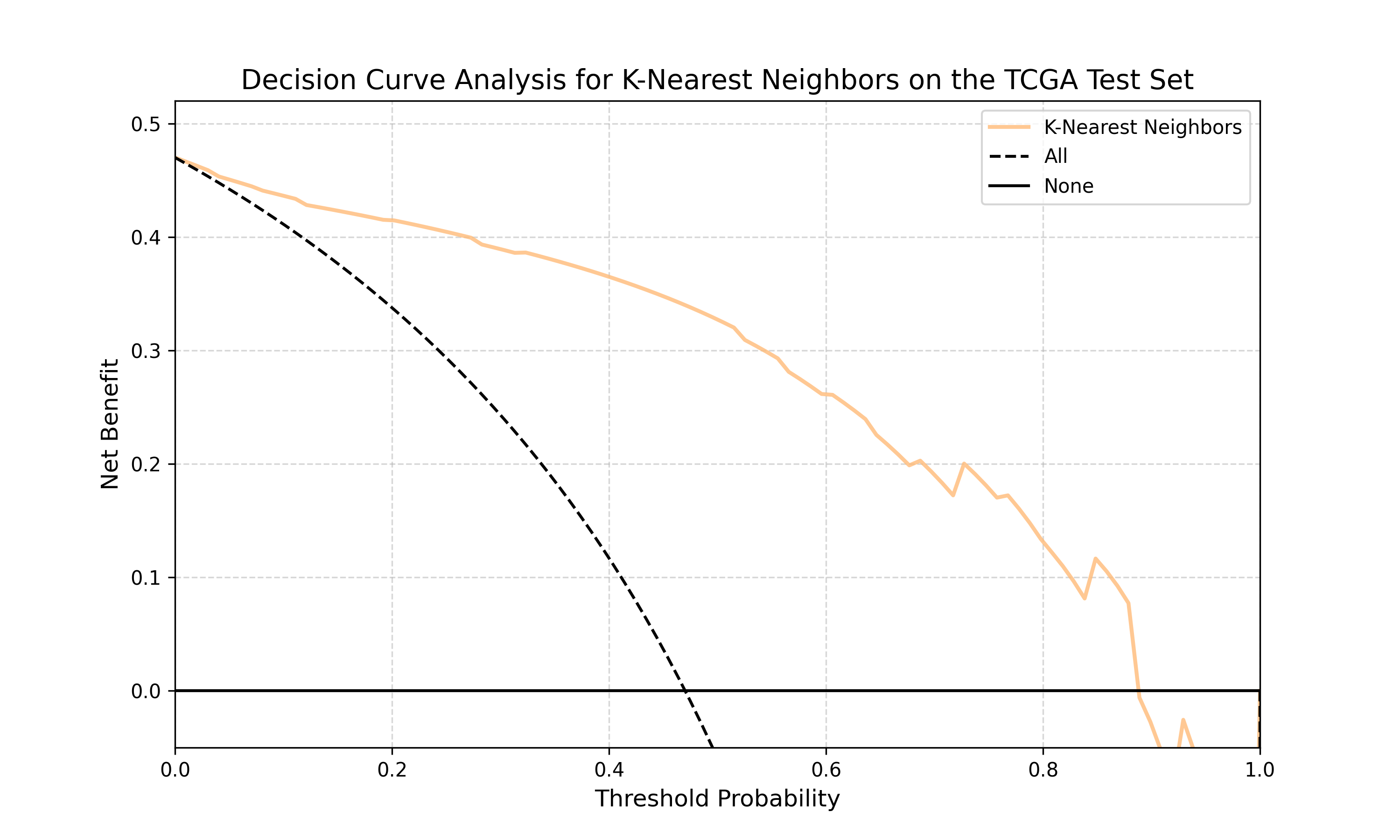

Supplement: S5 File — (ZIP) [file pone.0314831.s015.zip › S5 File/dca_curve_K-Nearest Neighbors.png]

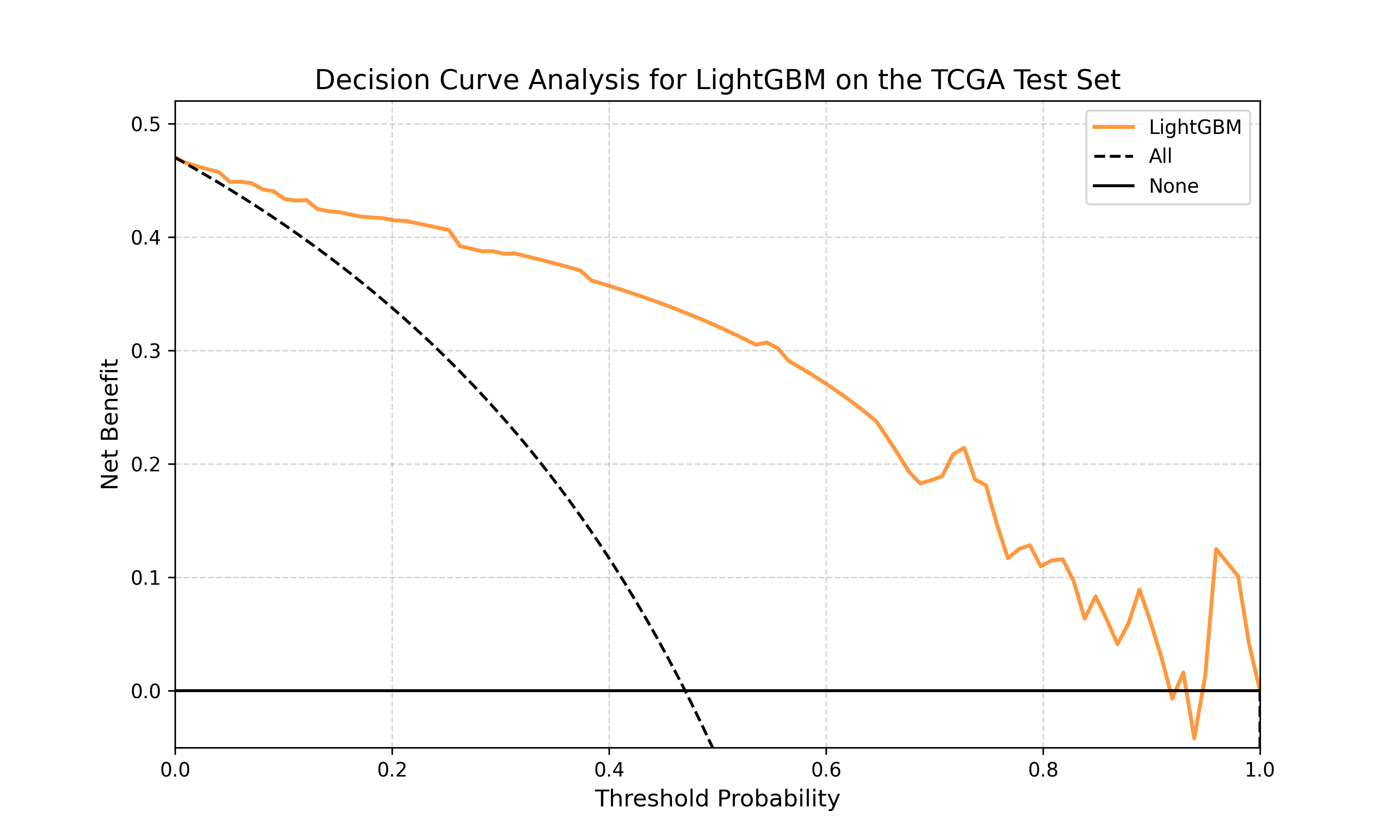

Supplement: S5 File — (ZIP) [file pone.0314831.s015.zip › S5 File/dca_curve_LightGBM.png]

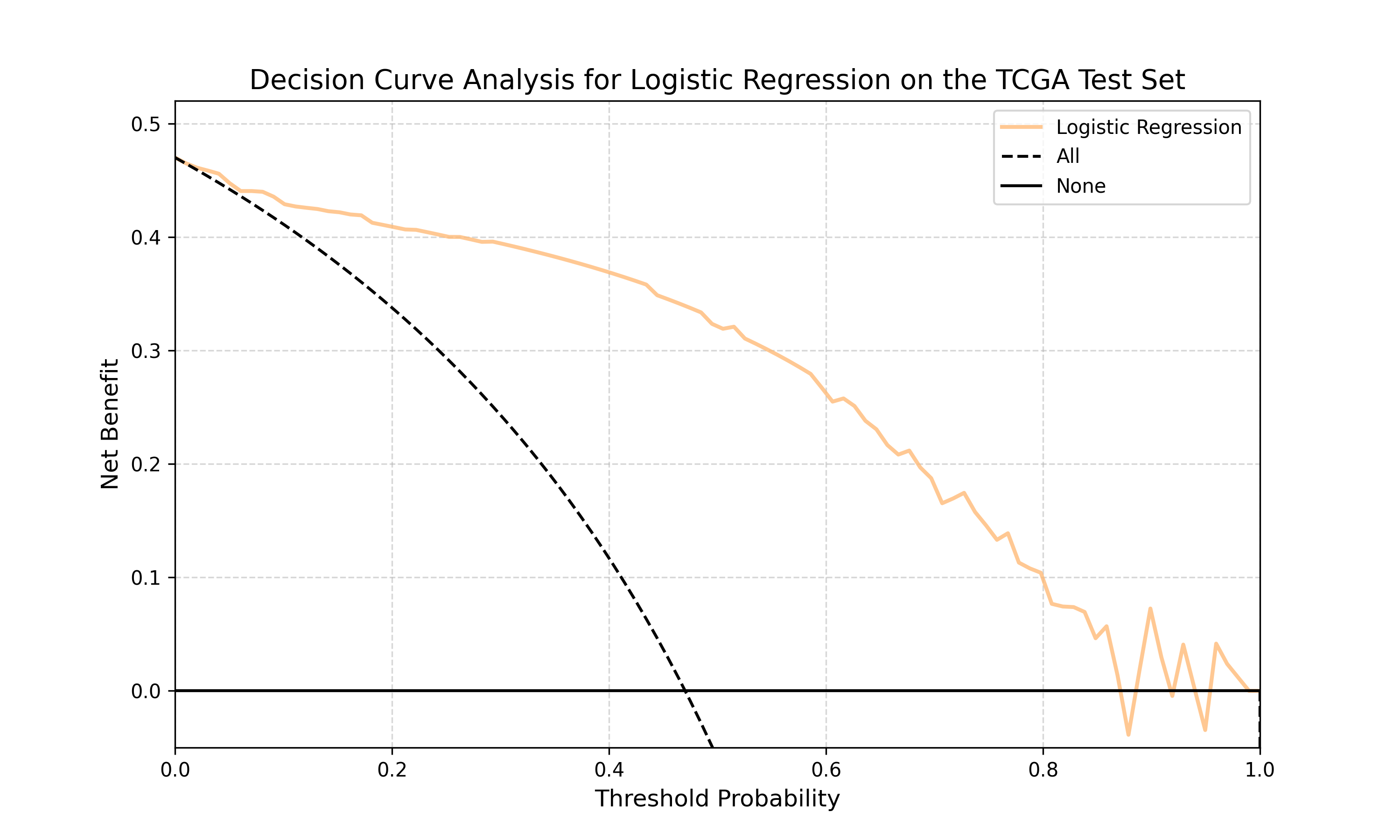

Supplement: S5 File — (ZIP) [file pone.0314831.s015.zip › S5 File/dca_curve_Logistic Regression.png]

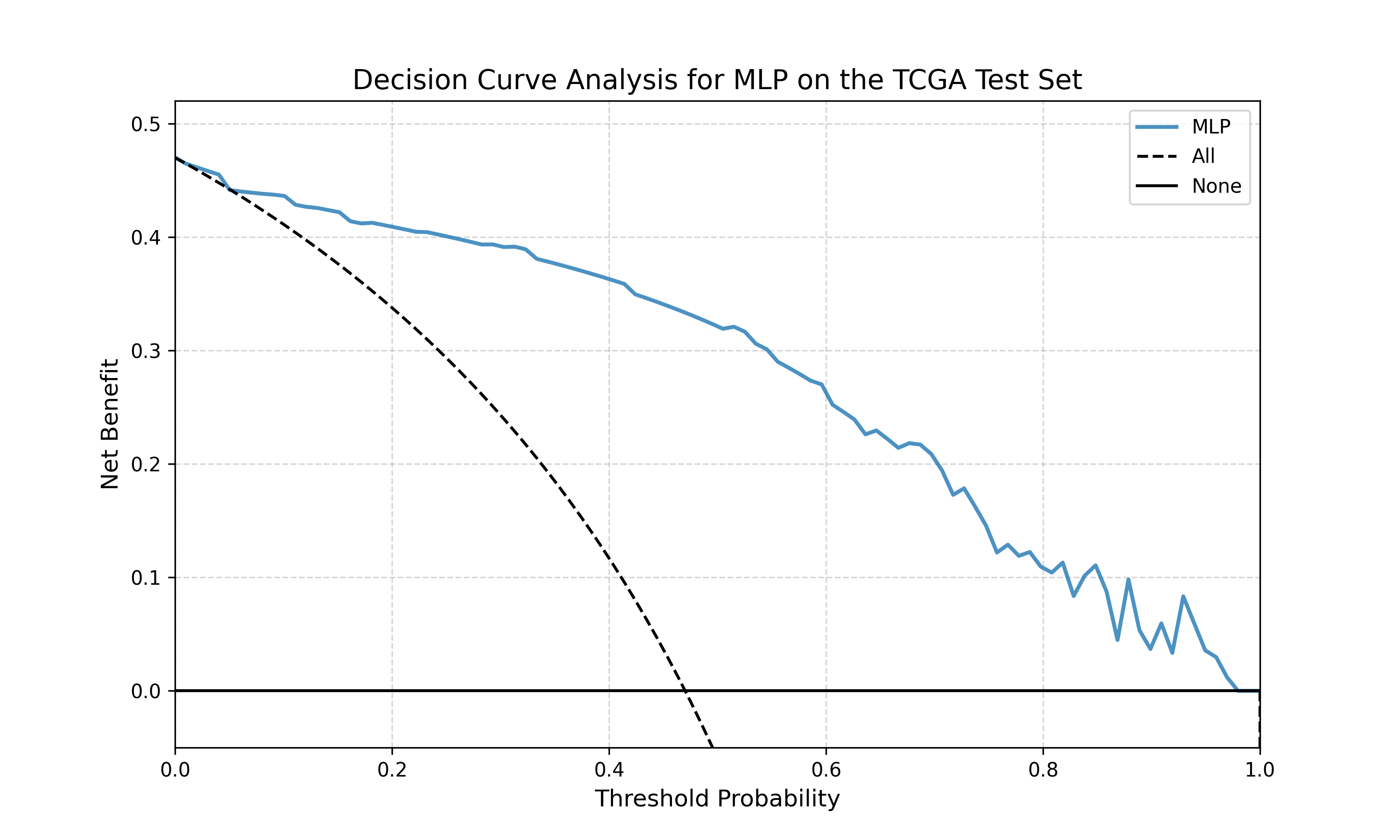

Supplement: S5 File — (ZIP) [file pone.0314831.s015.zip › S5 File/dca_curve_MLP.png]

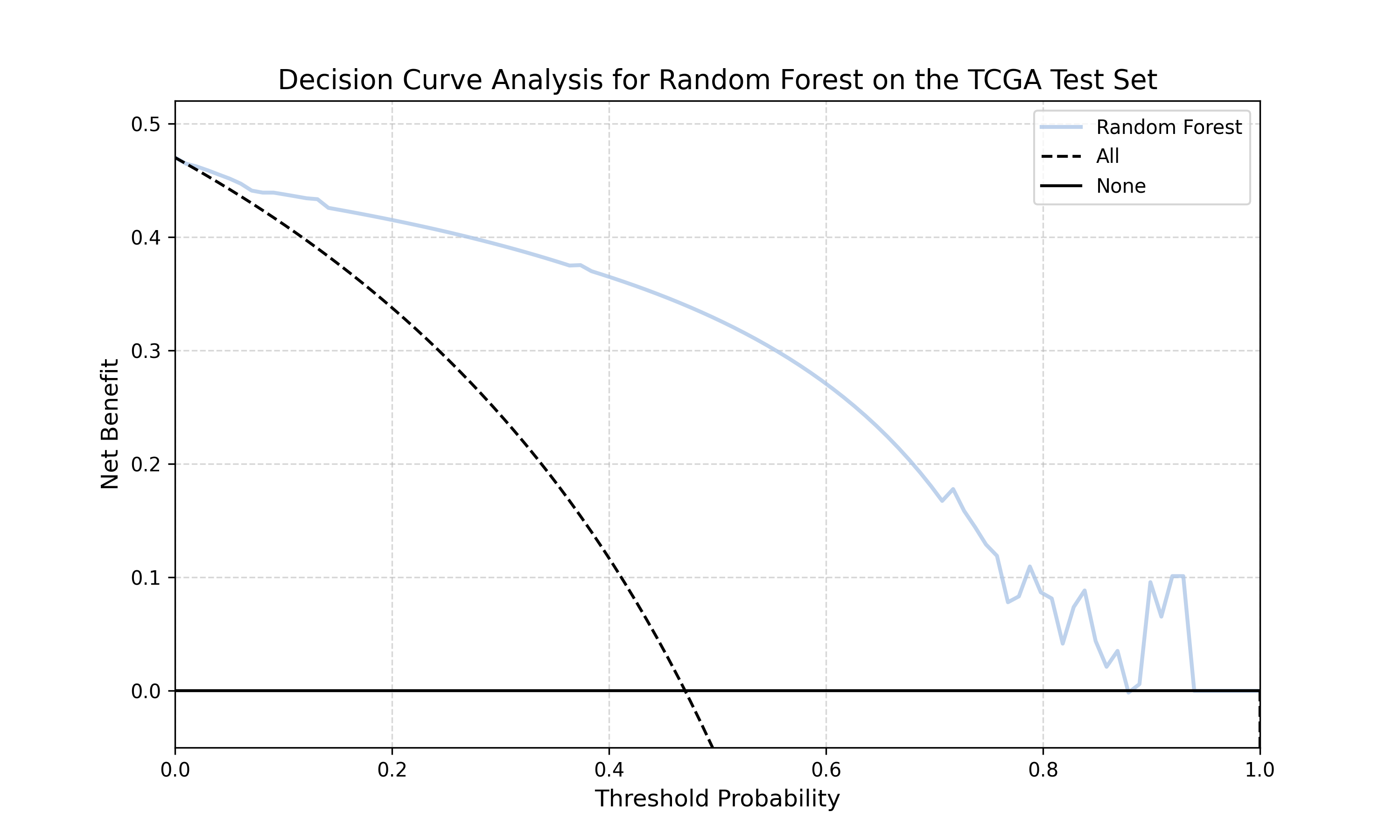

Supplement: S5 File — (ZIP) [file pone.0314831.s015.zip › S5 File/dca_curve_Random Forest.png]

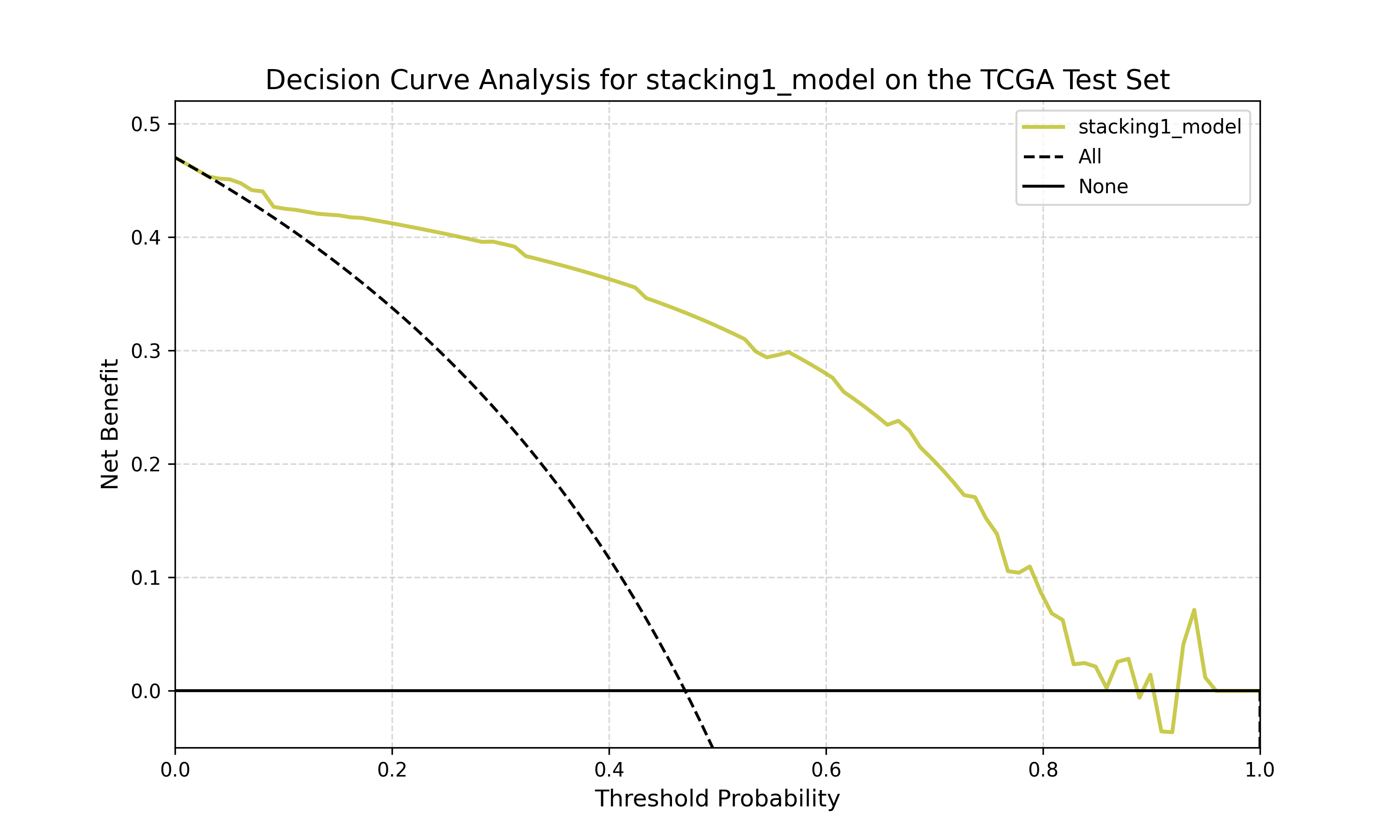

Supplement: S5 File — (ZIP) [file pone.0314831.s015.zip › S5 File/dca_curve_stacking1_model.png]

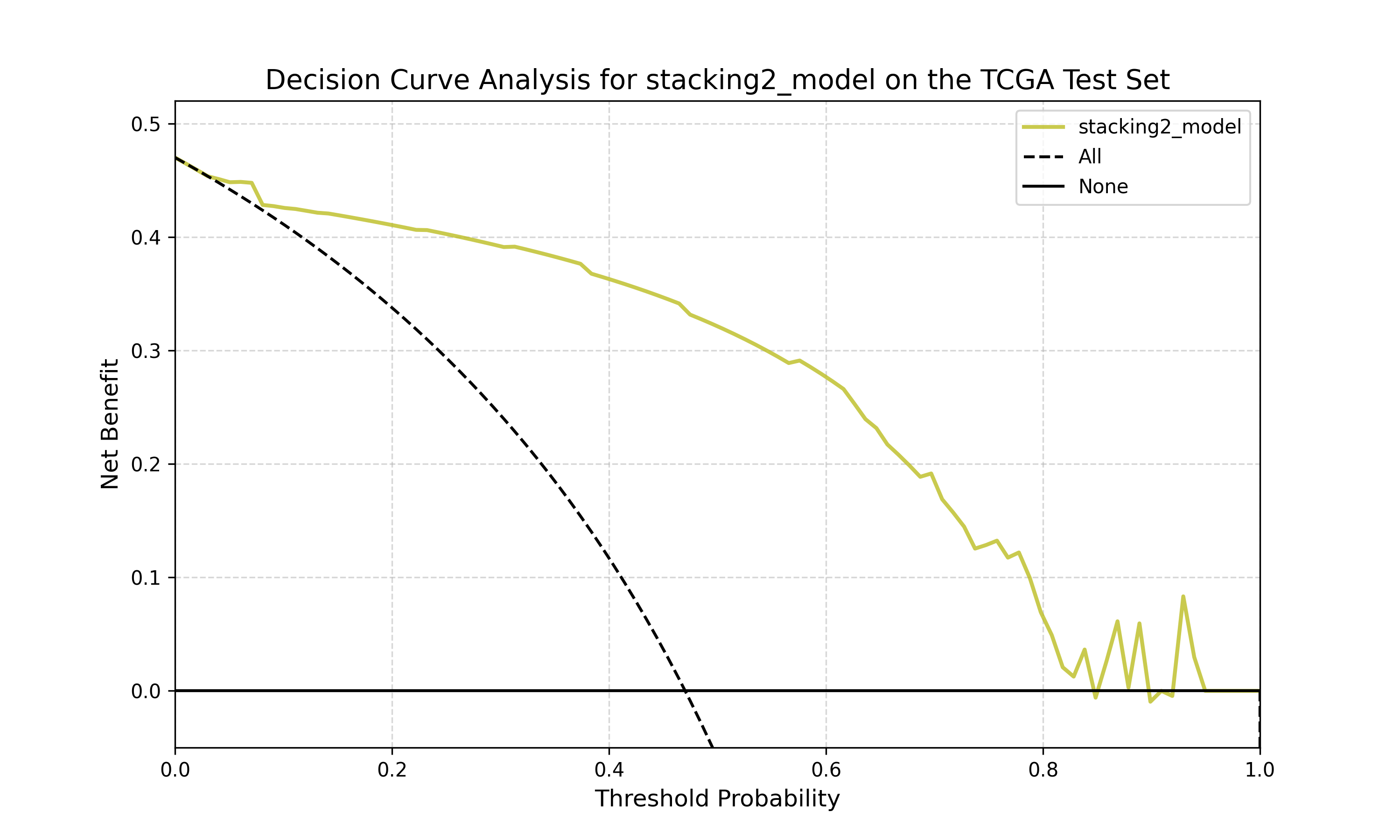

Supplement: S5 File — (ZIP) [file pone.0314831.s015.zip › S5 File/dca_curve_stacking2_model.png]

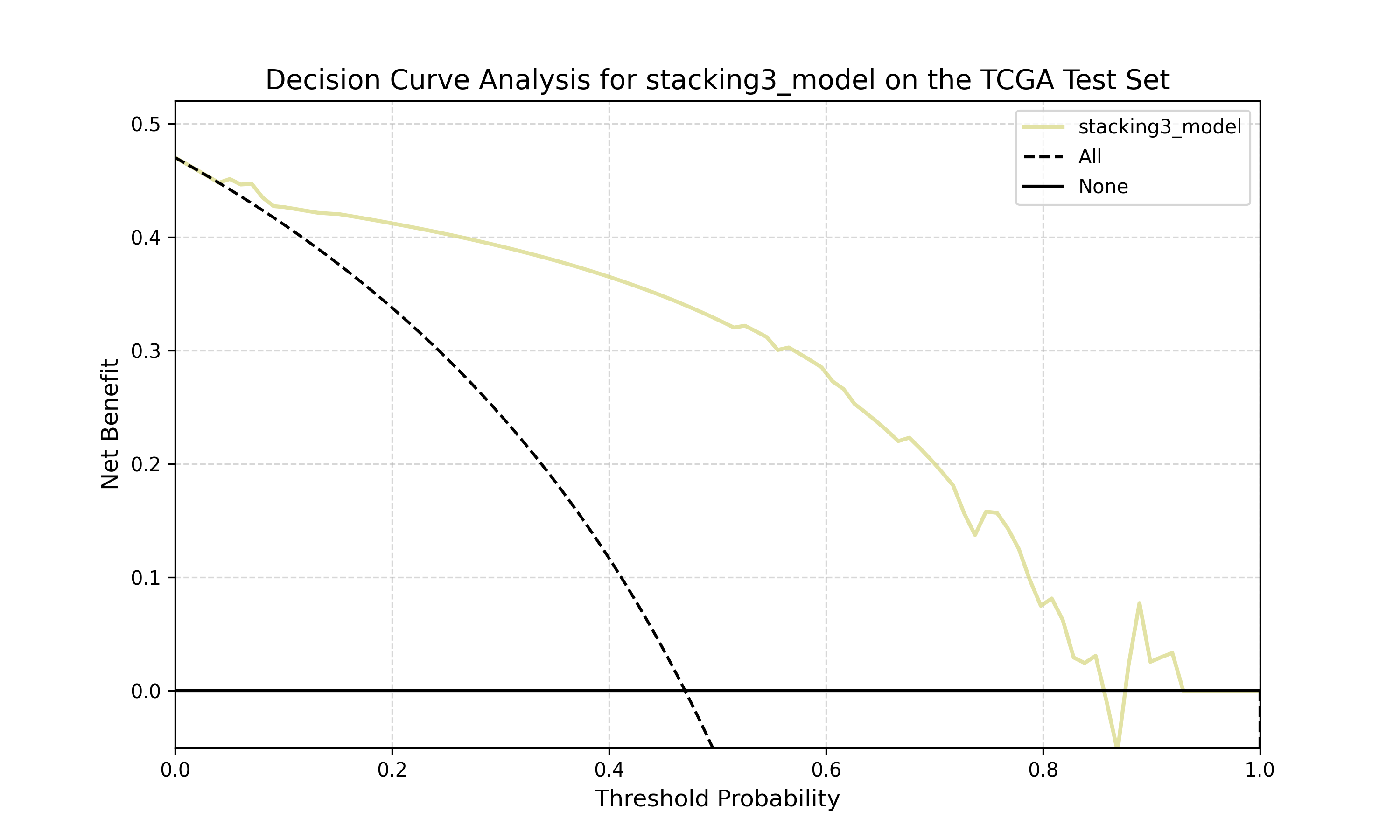

Supplement: S5 File — (ZIP) [file pone.0314831.s015.zip › S5 File/dca_curve_stacking3_model.png]

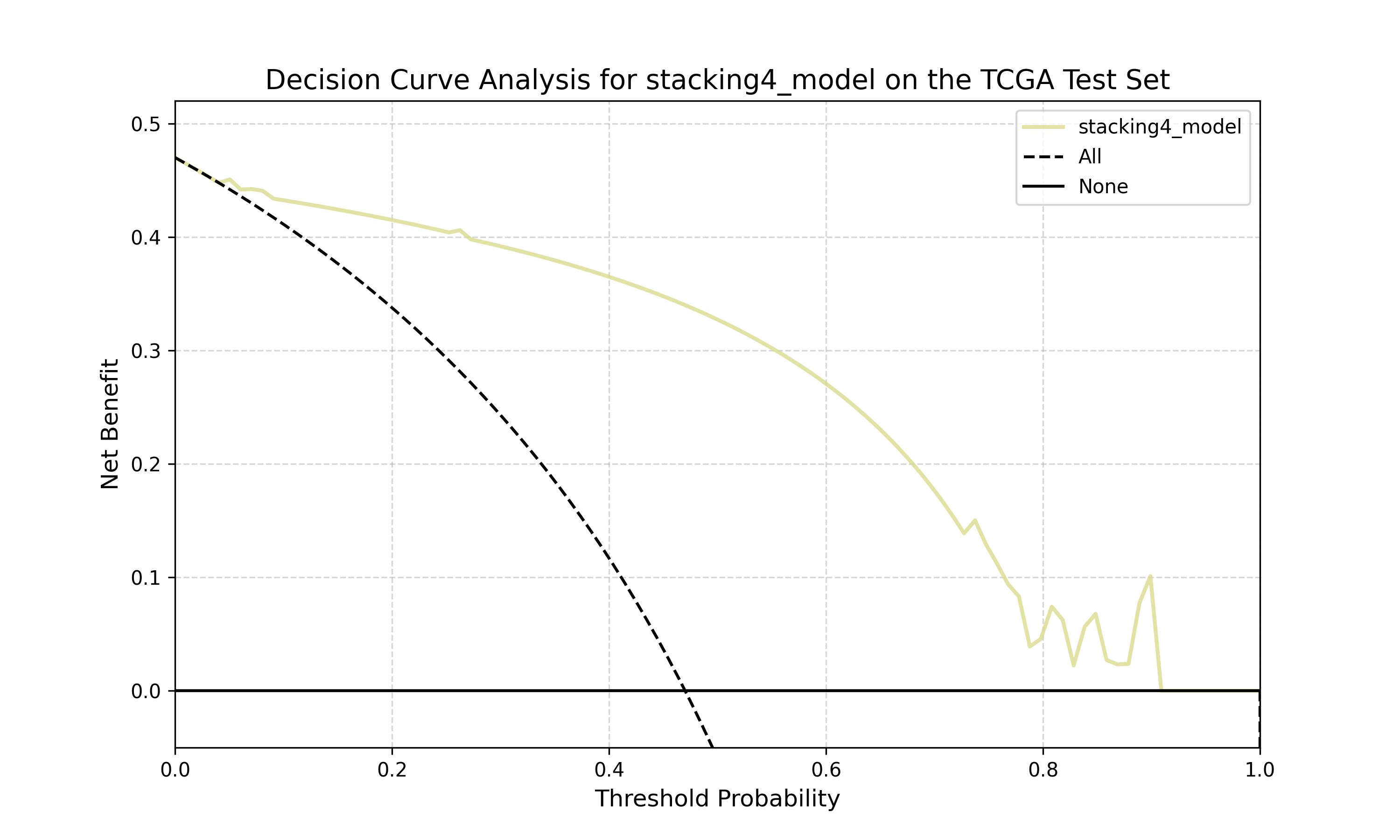

Supplement: S5 File — (ZIP) [file pone.0314831.s015.zip › S5 File/dca_curve_stacking4_model.png]

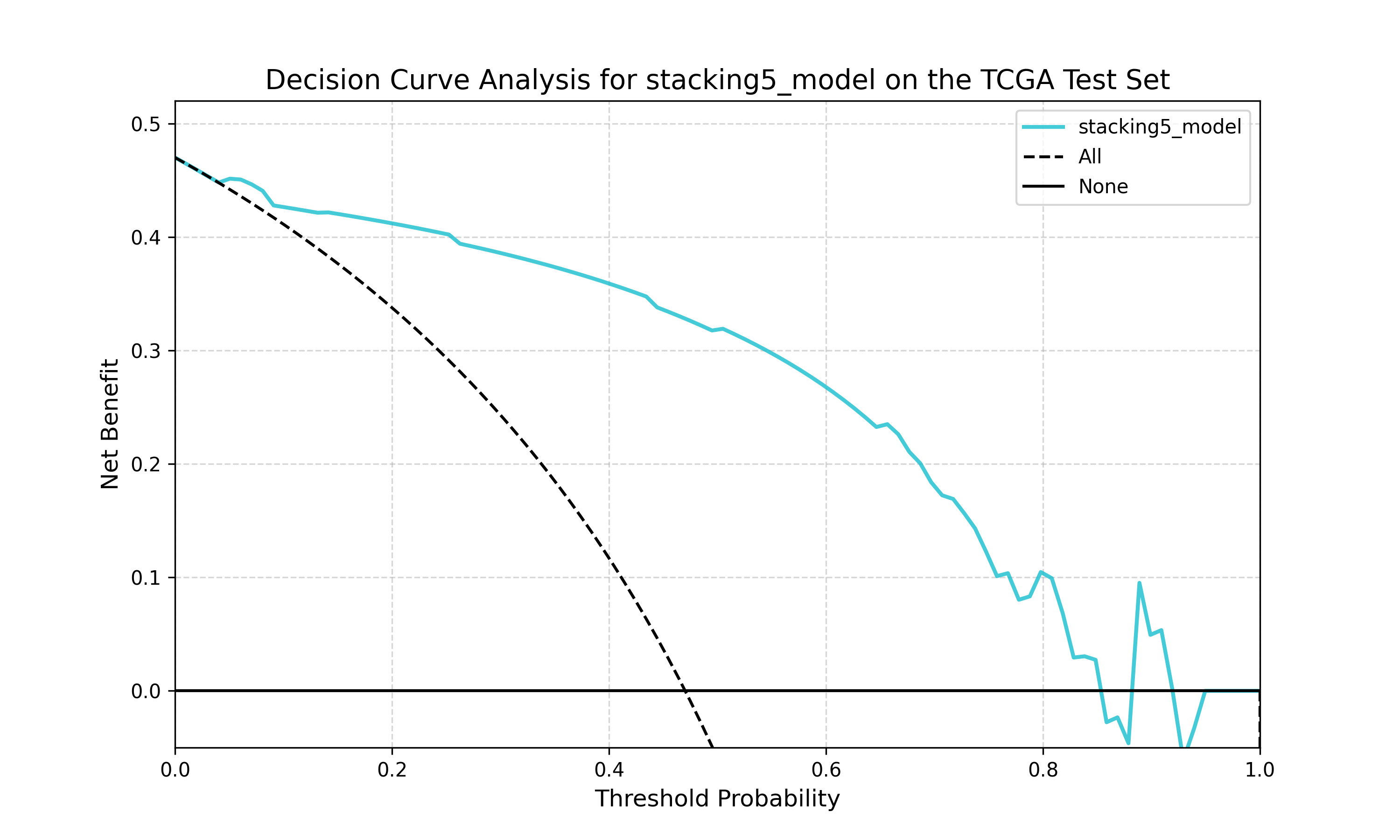

Supplement: S5 File — (ZIP) [file pone.0314831.s015.zip › S5 File/dca_curve_stacking5_model.png]

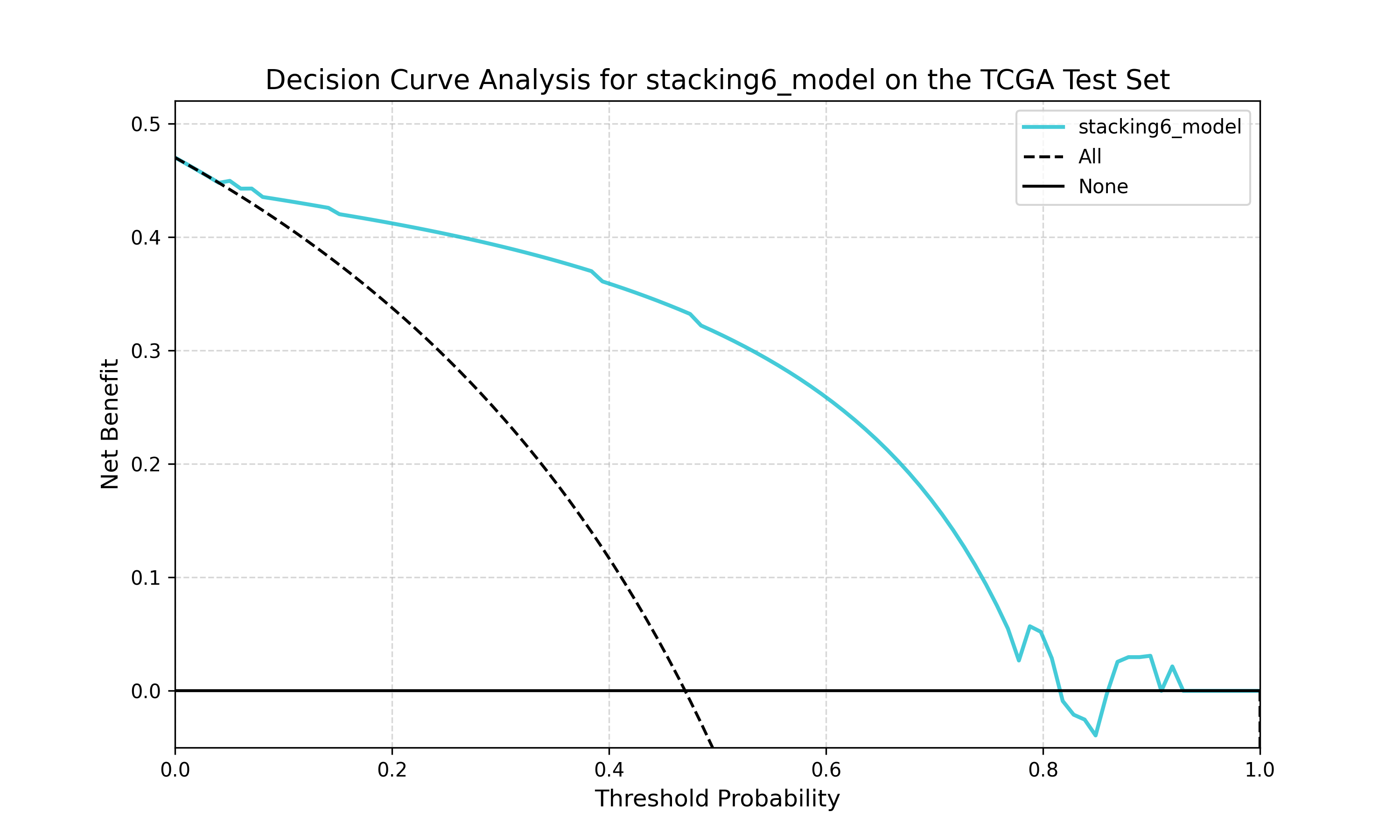

Supplement: S5 File — (ZIP) [file pone.0314831.s015.zip › S5 File/dca_curve_stacking6_model.png]
