# Supplementary figures and images for: Machine learning-based prediction of glioma grading (part 2 of 5)
Source: PLoS One. 2025 Dec 26;20(12):e0314831. doi: 10.1371/journal.pone.0314831 (PMC12742763; doi:10.1371/journal.pone.0314831)

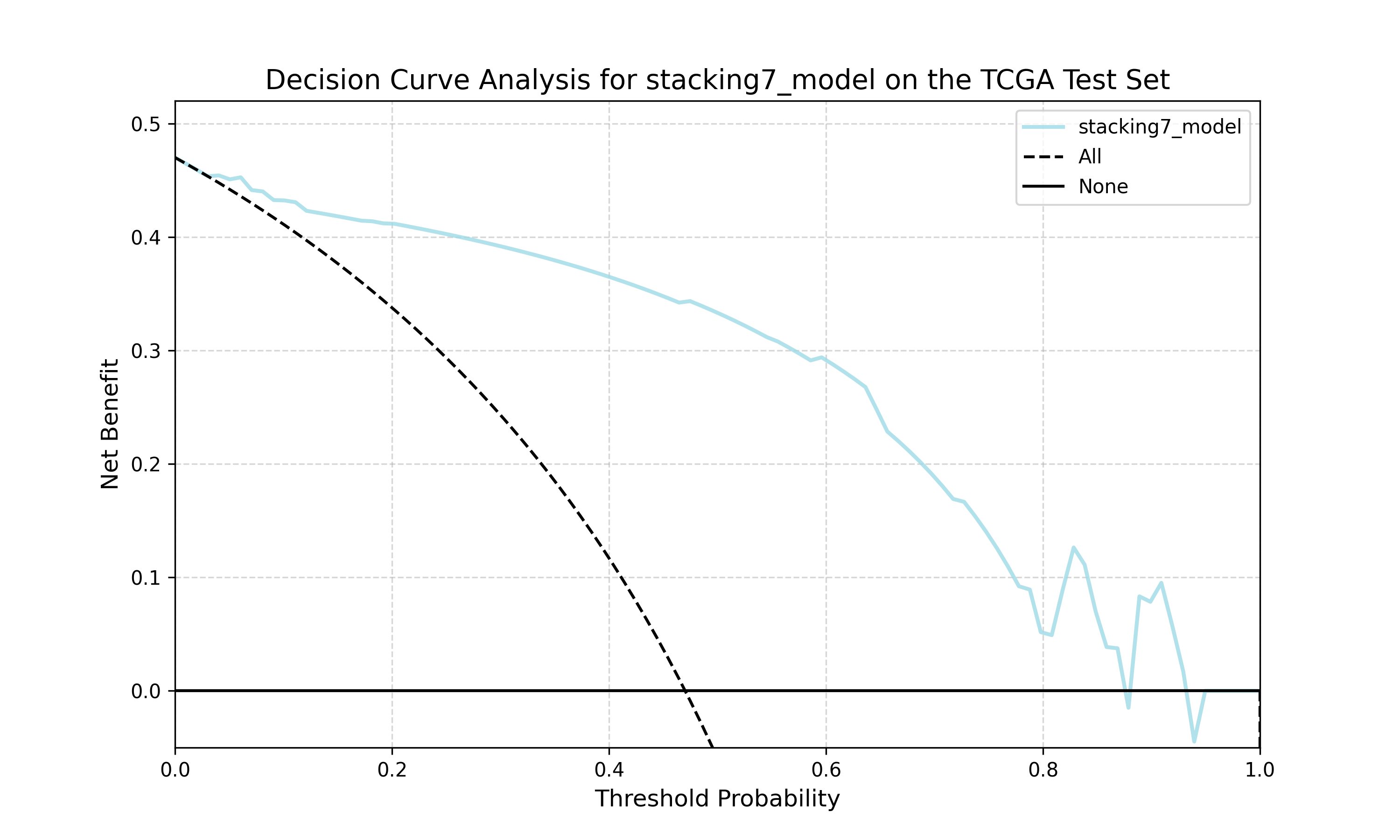

Supplement: S5 File — (ZIP) [file pone.0314831.s015.zip › S5 File/dca_curve_stacking7_model.png]

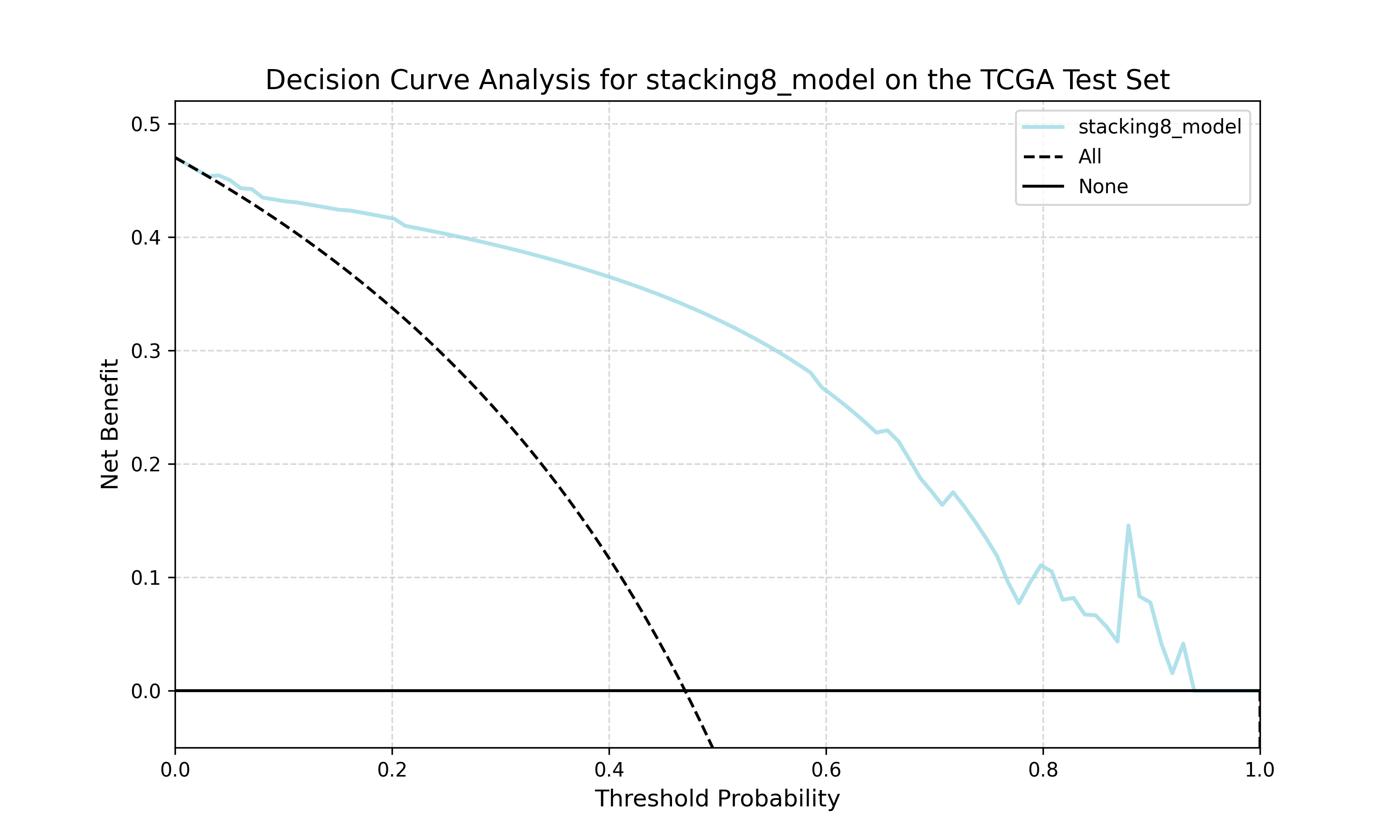

Supplement: S5 File — (ZIP) [file pone.0314831.s015.zip › S5 File/dca_curve_stacking8_model.png]

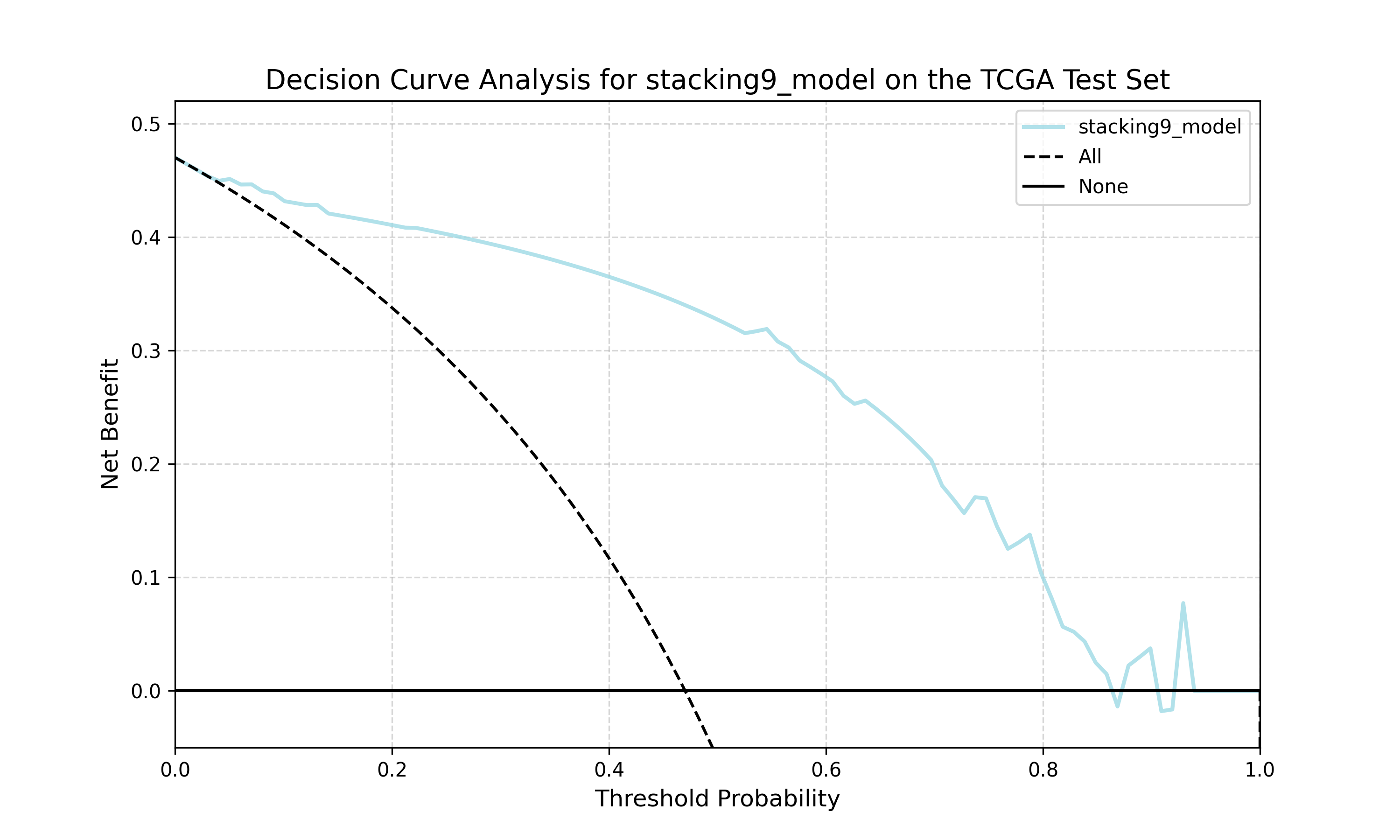

Supplement: S5 File — (ZIP) [file pone.0314831.s015.zip › S5 File/dca_curve_stacking9_model.png]

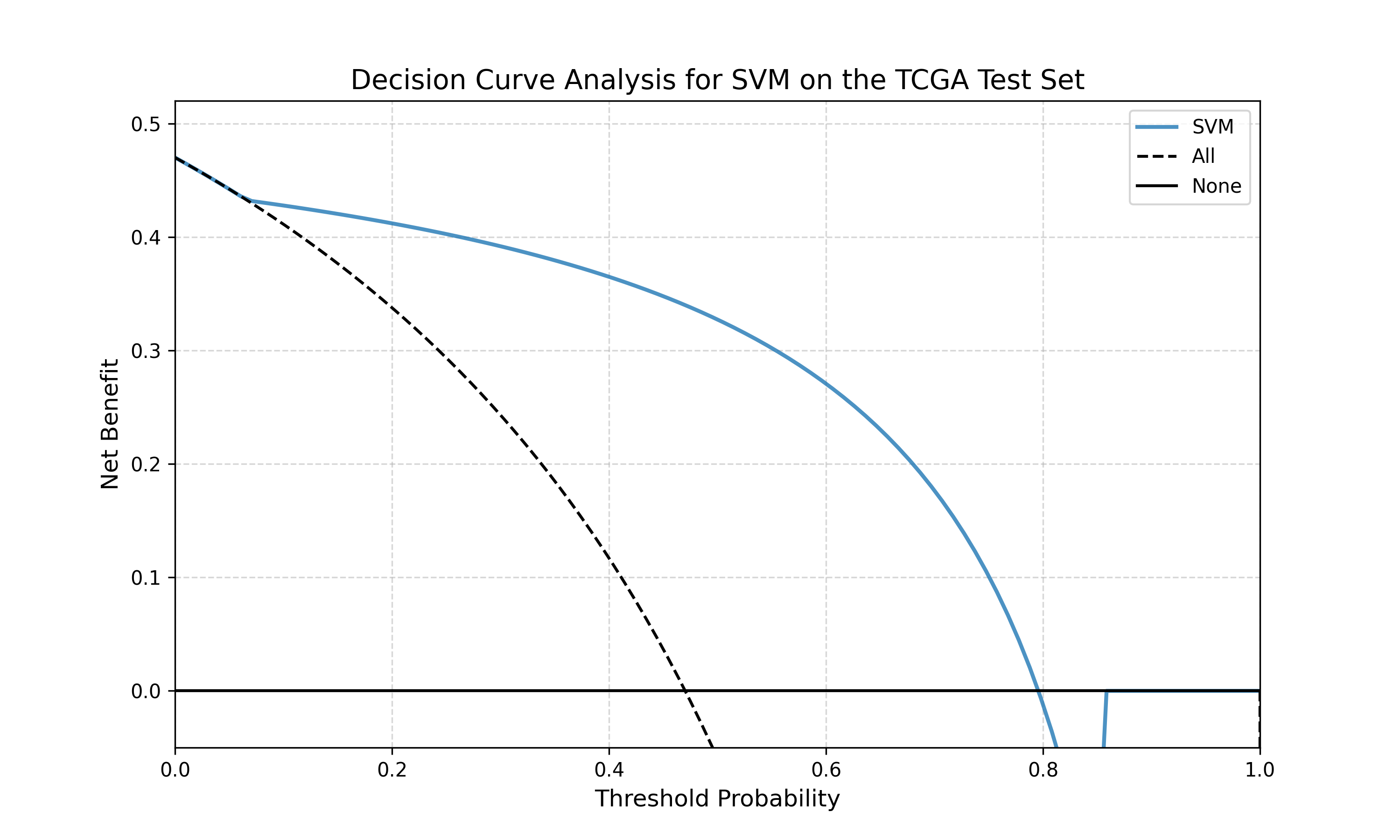

Supplement: S5 File — (ZIP) [file pone.0314831.s015.zip › S5 File/dca_curve_SVM.png]

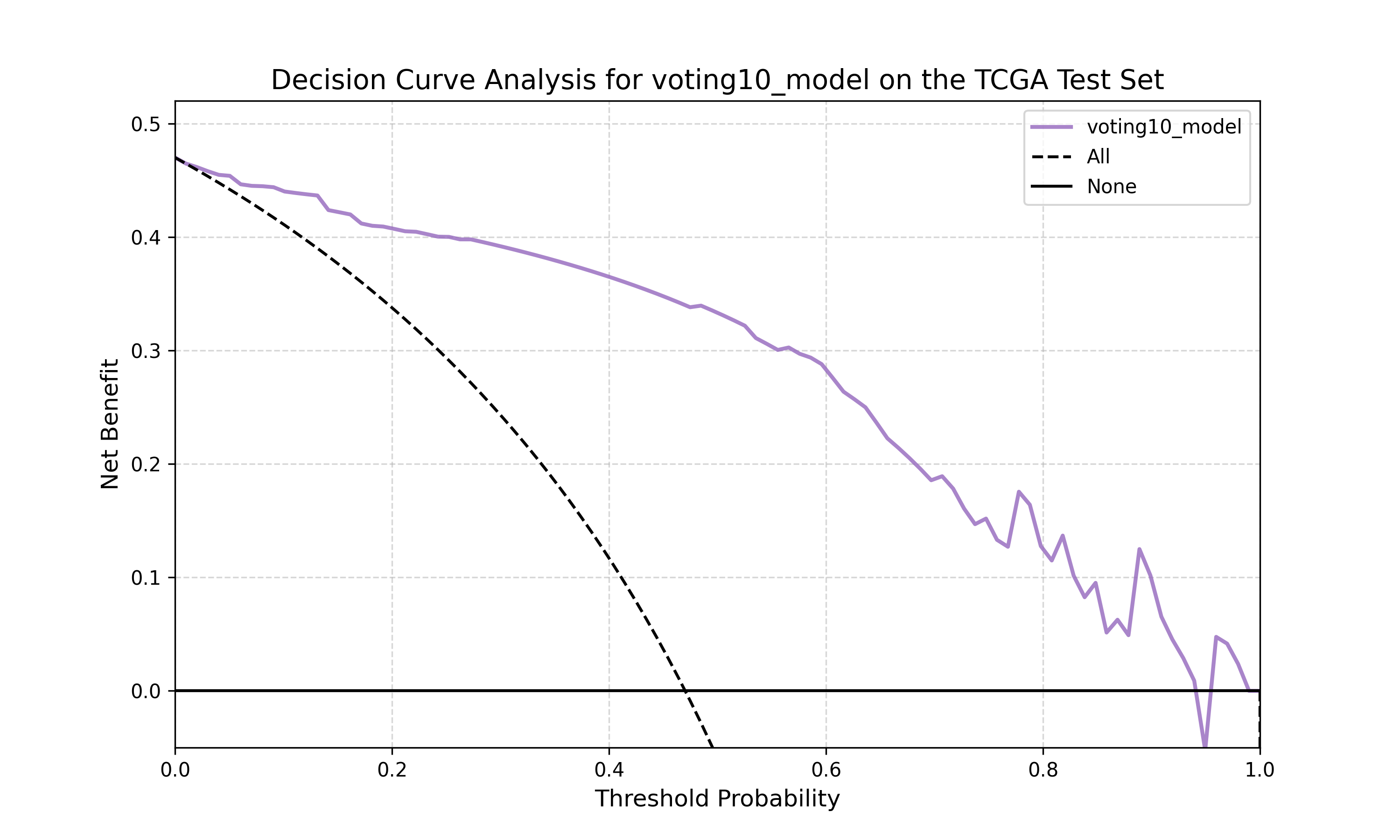

Supplement: S5 File — (ZIP) [file pone.0314831.s015.zip › S5 File/dca_curve_voting10_model.png]

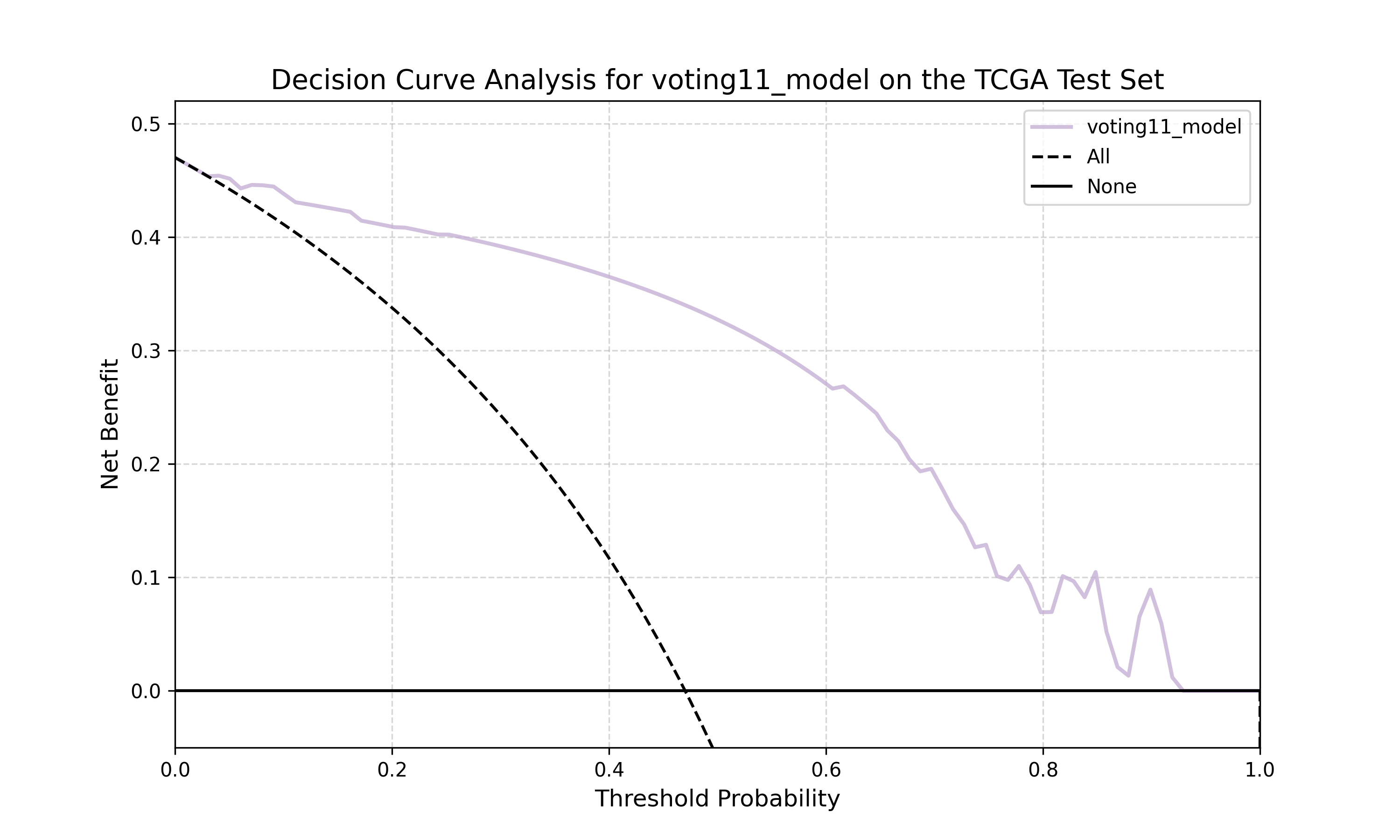

Supplement: S5 File — (ZIP) [file pone.0314831.s015.zip › S5 File/dca_curve_voting11_model.png]

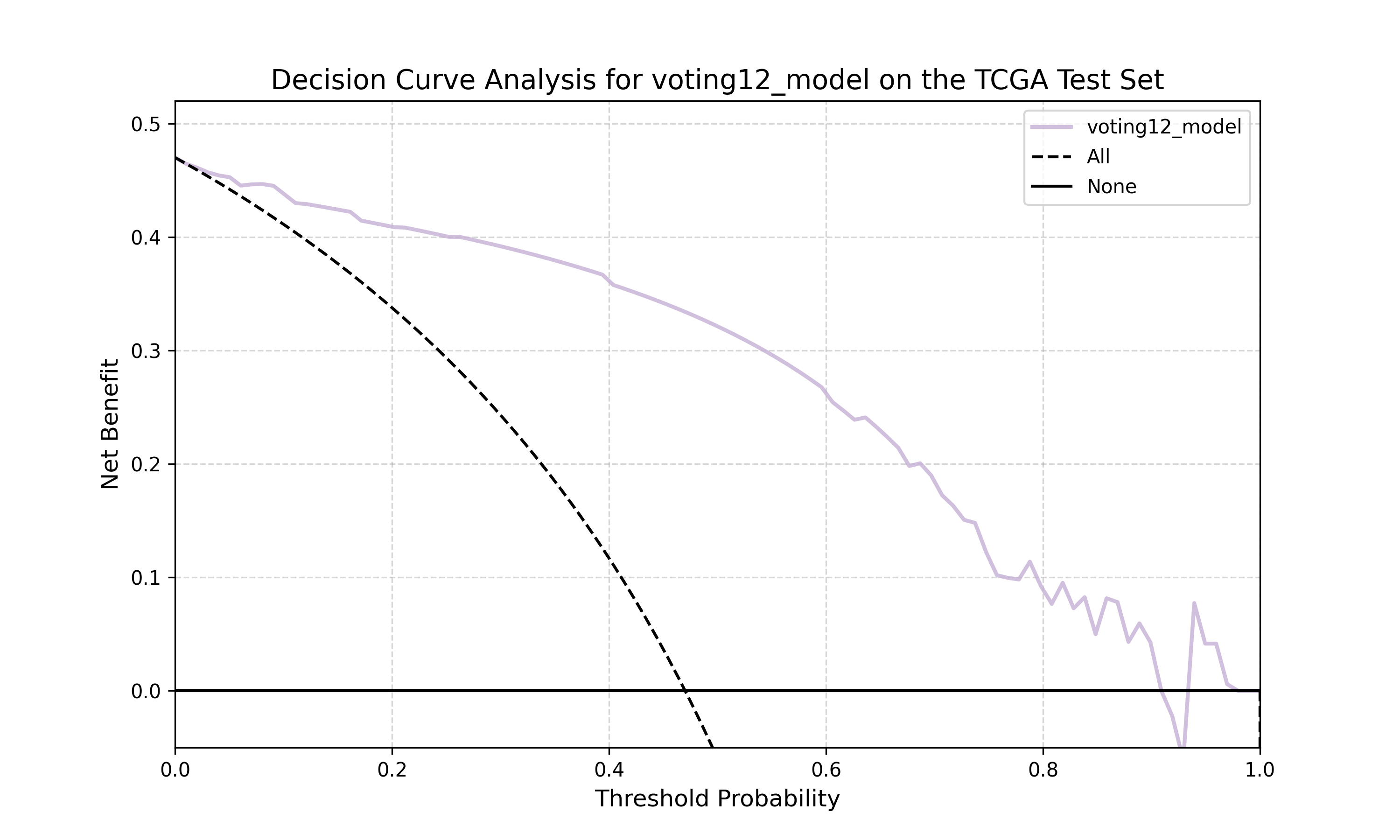

Supplement: S5 File — (ZIP) [file pone.0314831.s015.zip › S5 File/dca_curve_voting12_model.png]

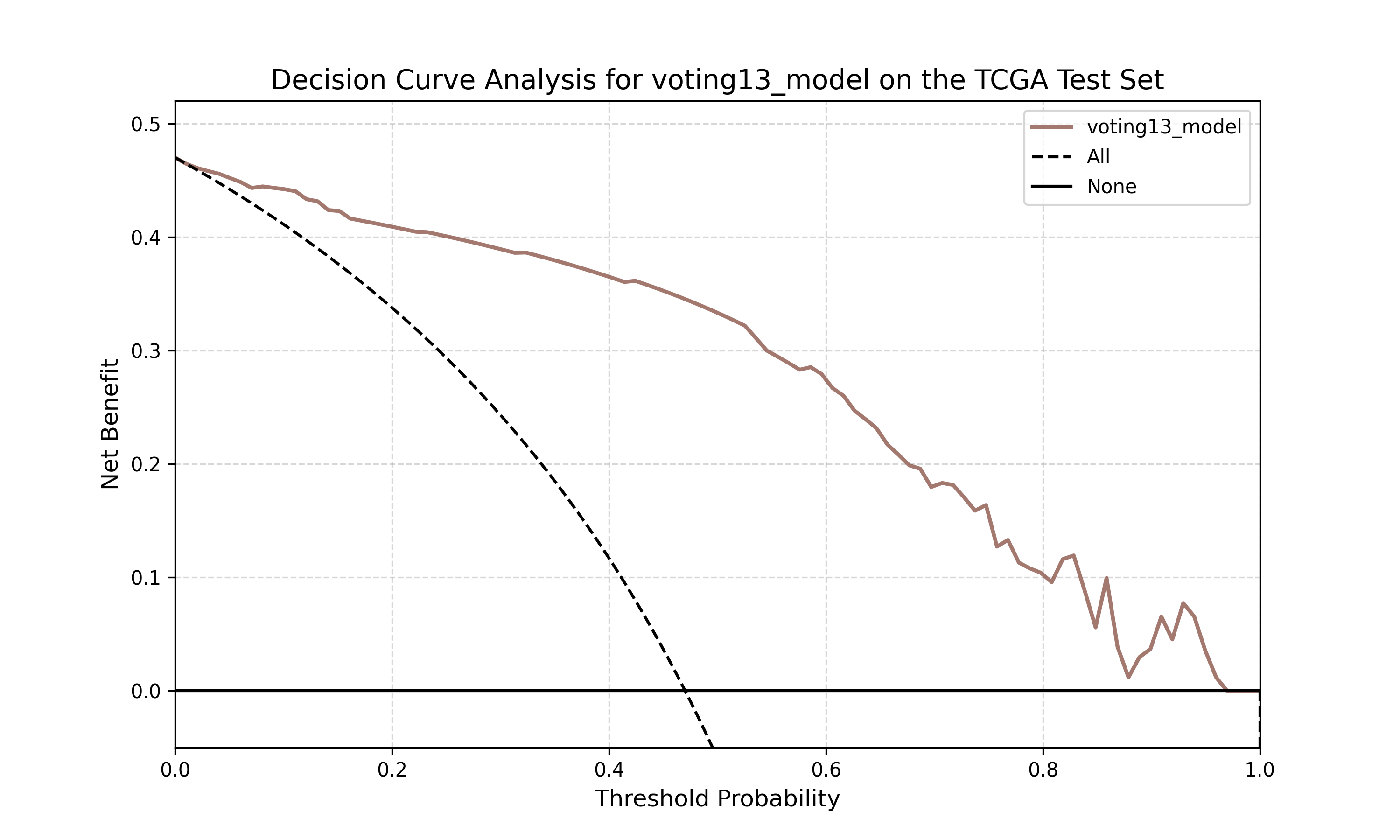

Supplement: S5 File — (ZIP) [file pone.0314831.s015.zip › S5 File/dca_curve_voting13_model.png]

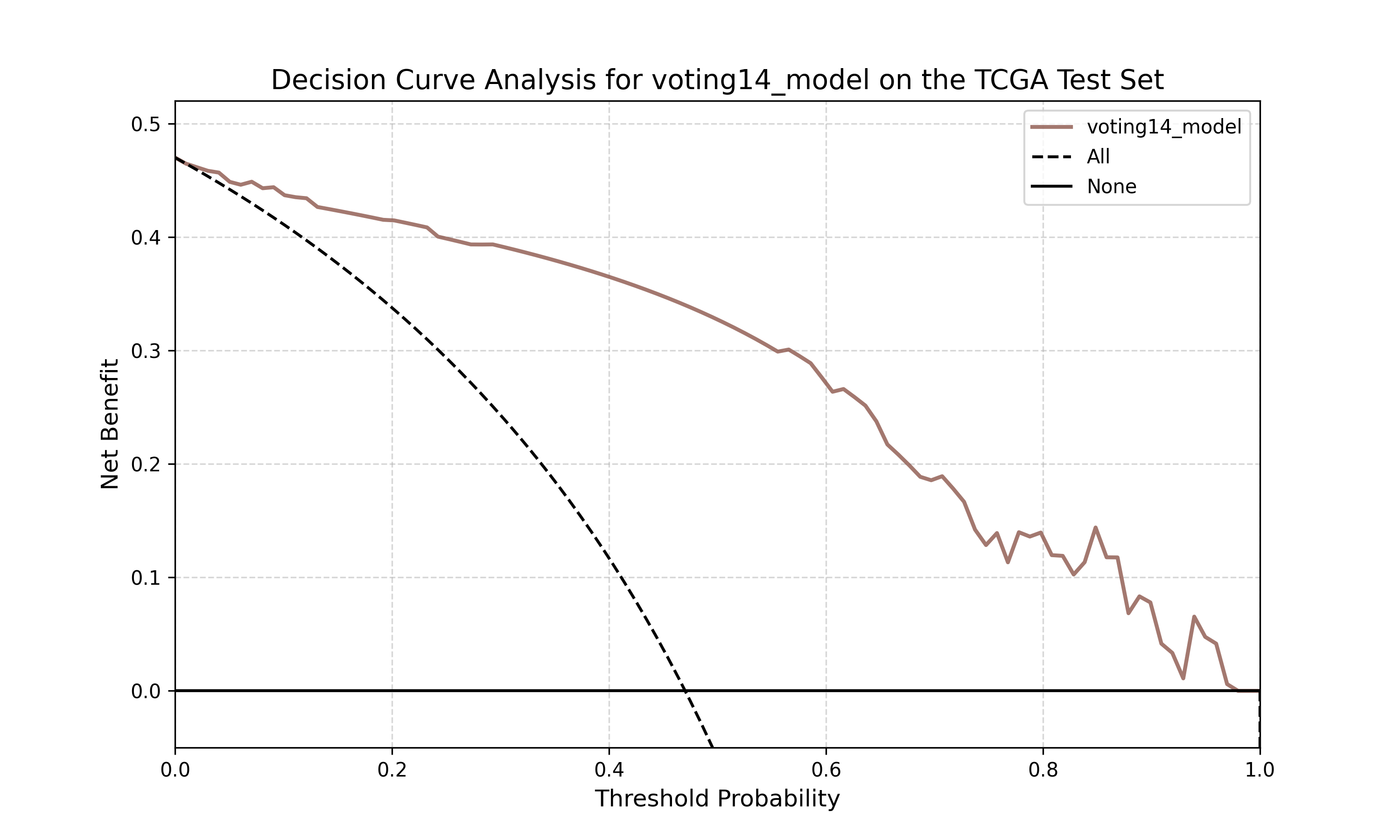

Supplement: S5 File — (ZIP) [file pone.0314831.s015.zip › S5 File/dca_curve_voting14_model.png]

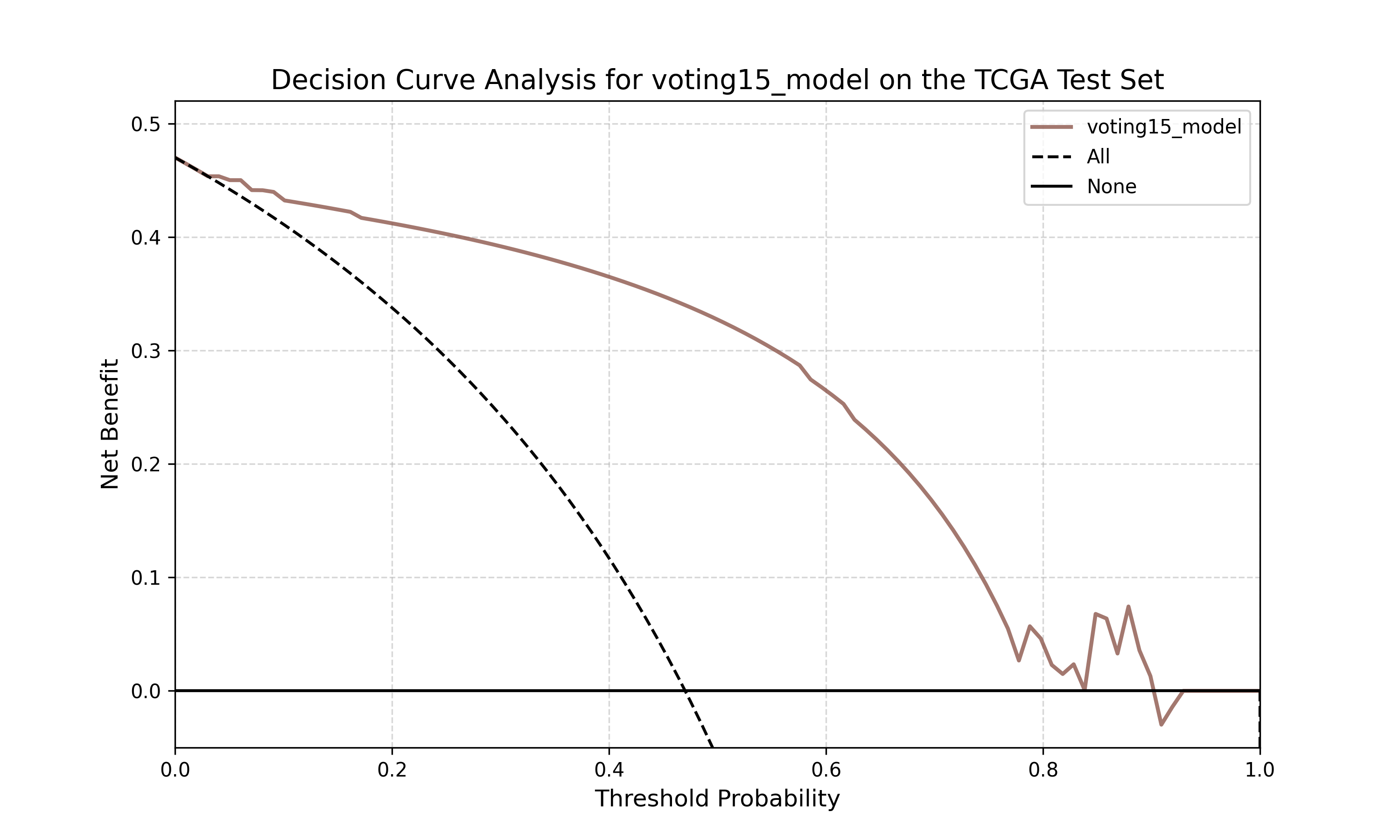

Supplement: S5 File — (ZIP) [file pone.0314831.s015.zip › S5 File/dca_curve_voting15_model.png]

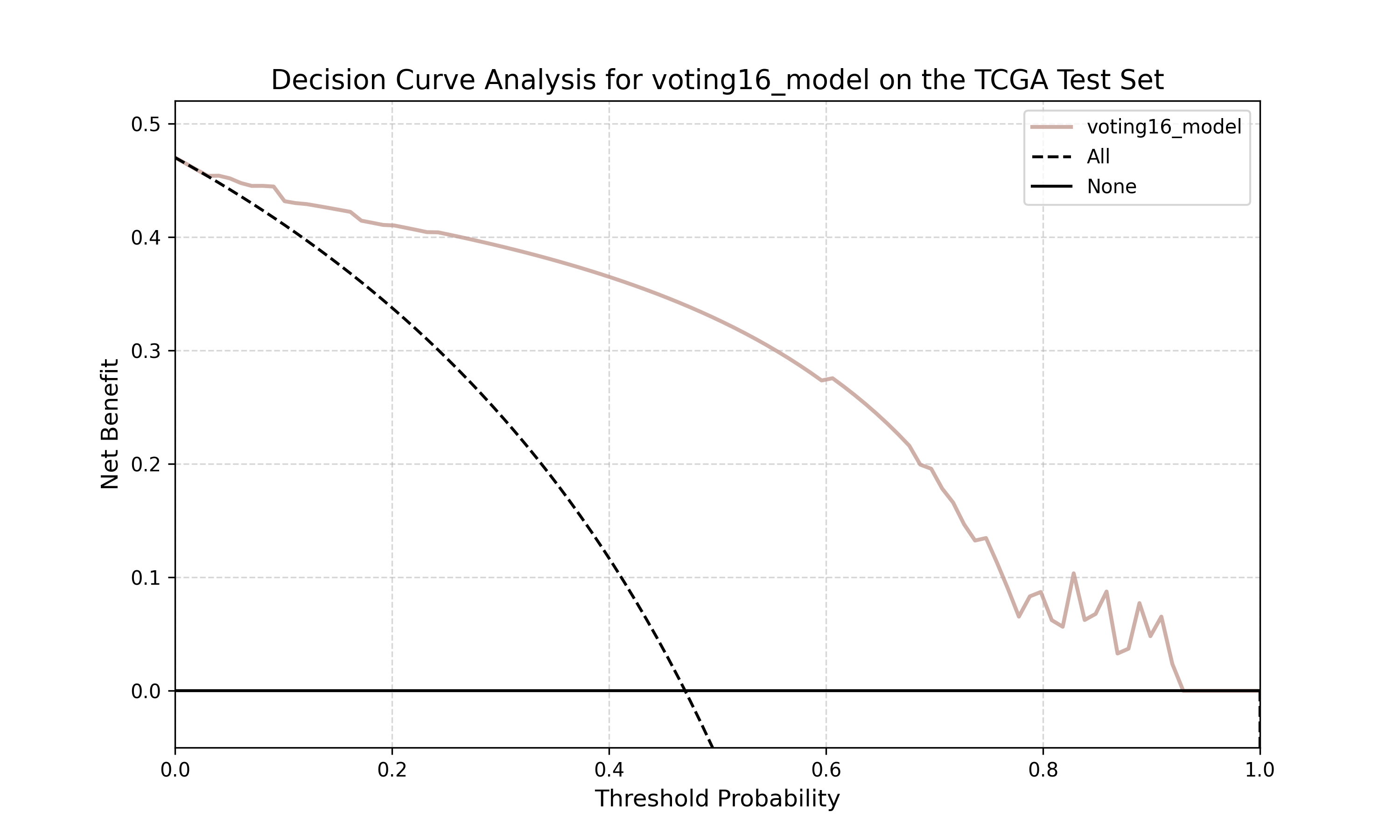

Supplement: S5 File — (ZIP) [file pone.0314831.s015.zip › S5 File/dca_curve_voting16_model.png]

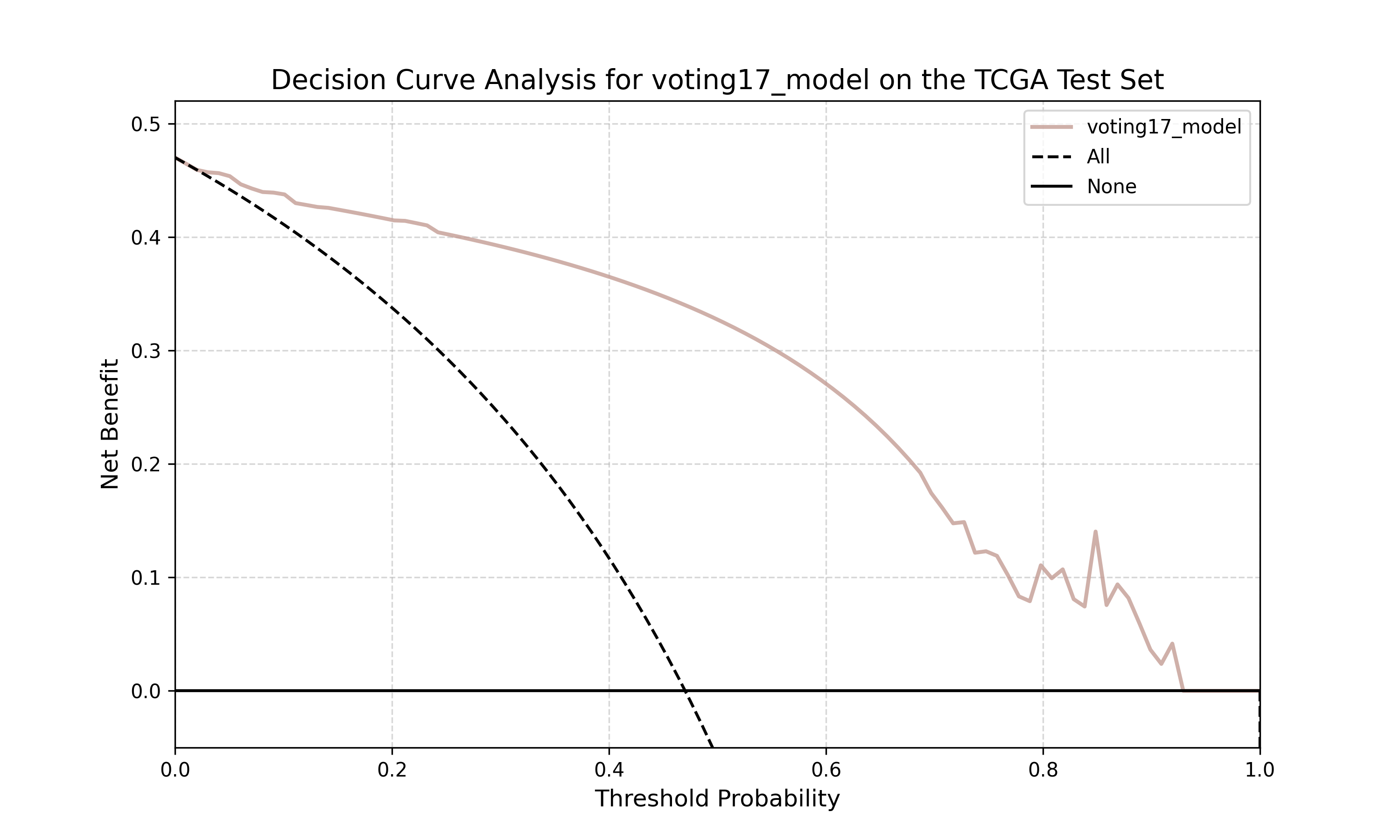

Supplement: S5 File — (ZIP) [file pone.0314831.s015.zip › S5 File/dca_curve_voting17_model.png]

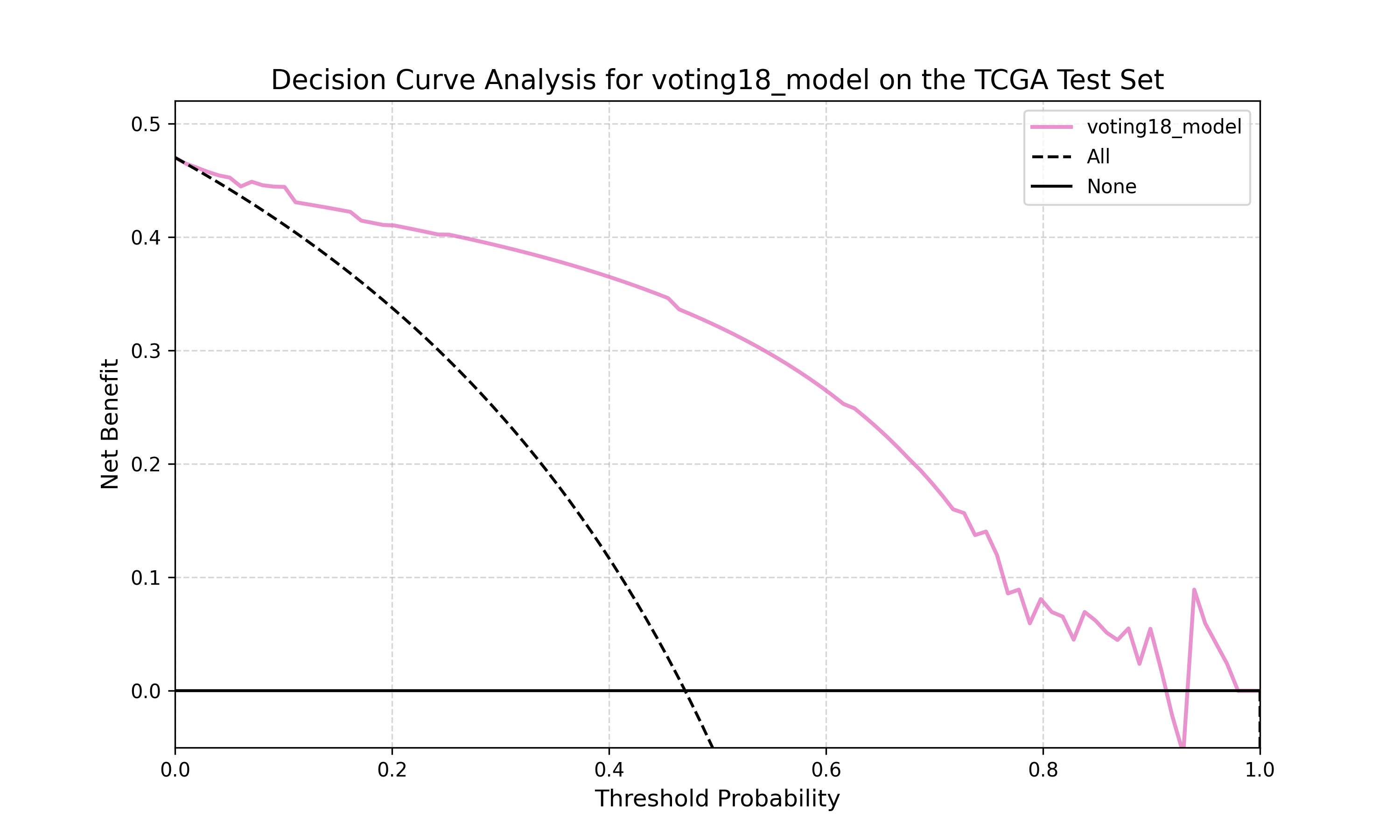

Supplement: S5 File — (ZIP) [file pone.0314831.s015.zip › S5 File/dca_curve_voting18_model.png]

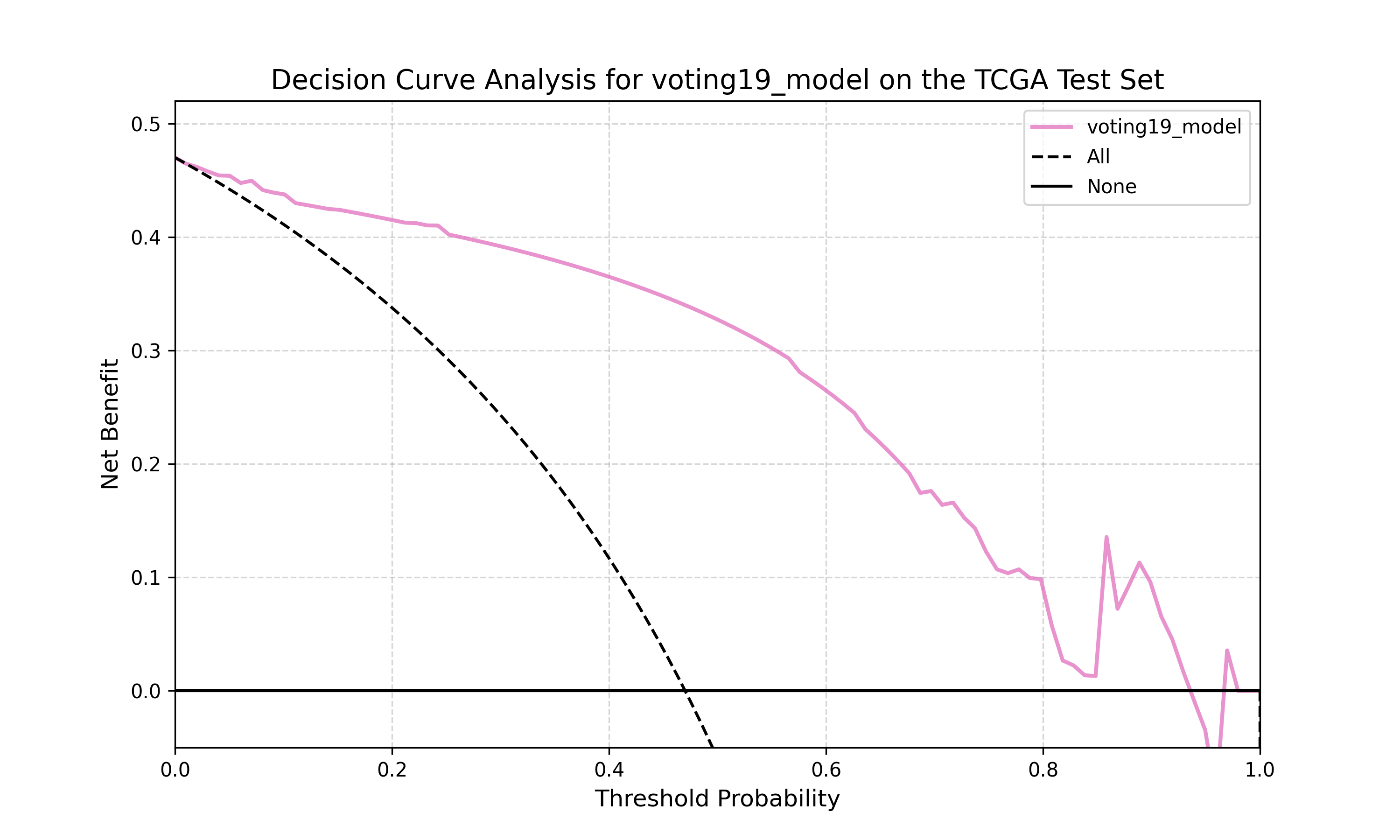

Supplement: S5 File — (ZIP) [file pone.0314831.s015.zip › S5 File/dca_curve_voting19_model.png]

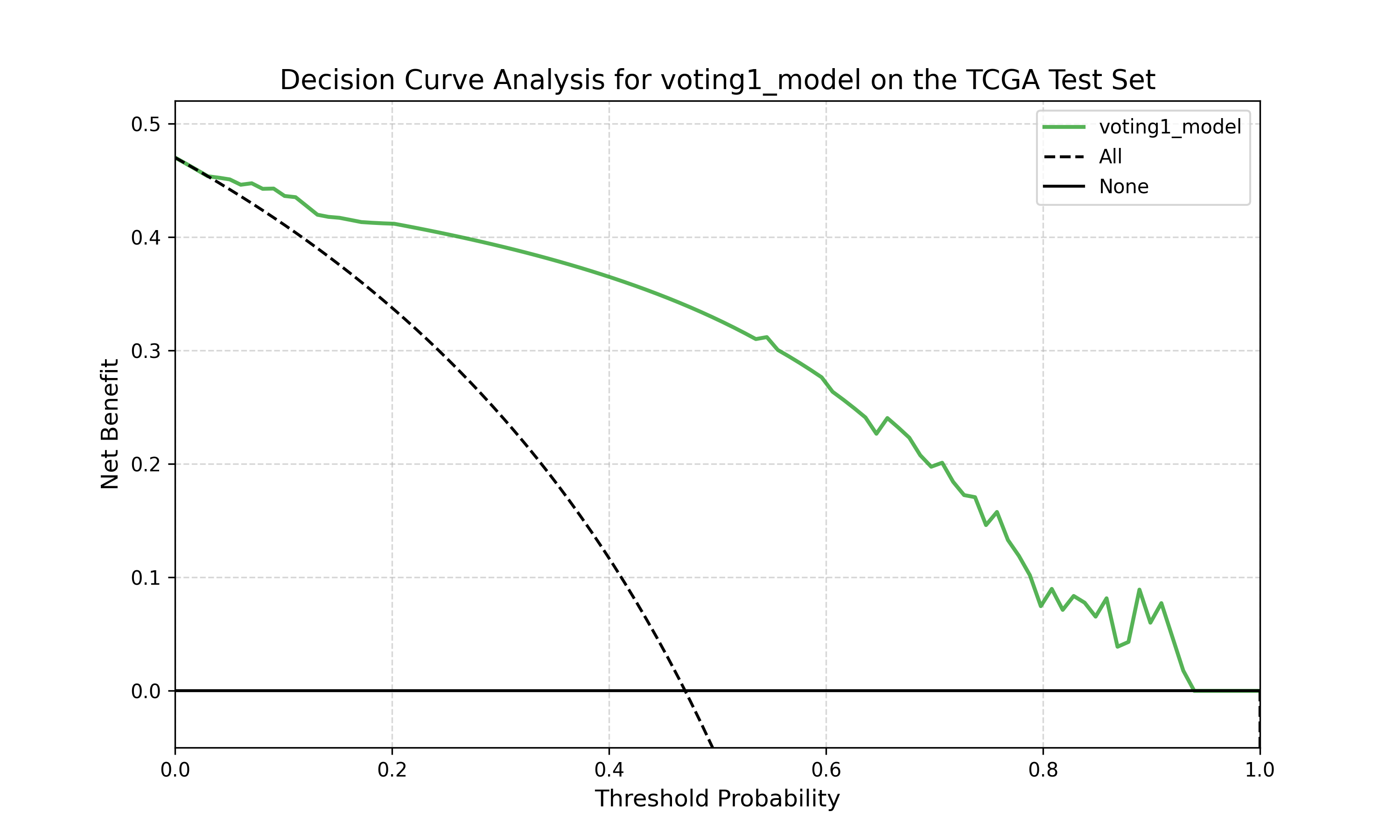

Supplement: S5 File — (ZIP) [file pone.0314831.s015.zip › S5 File/dca_curve_voting1_model.png]

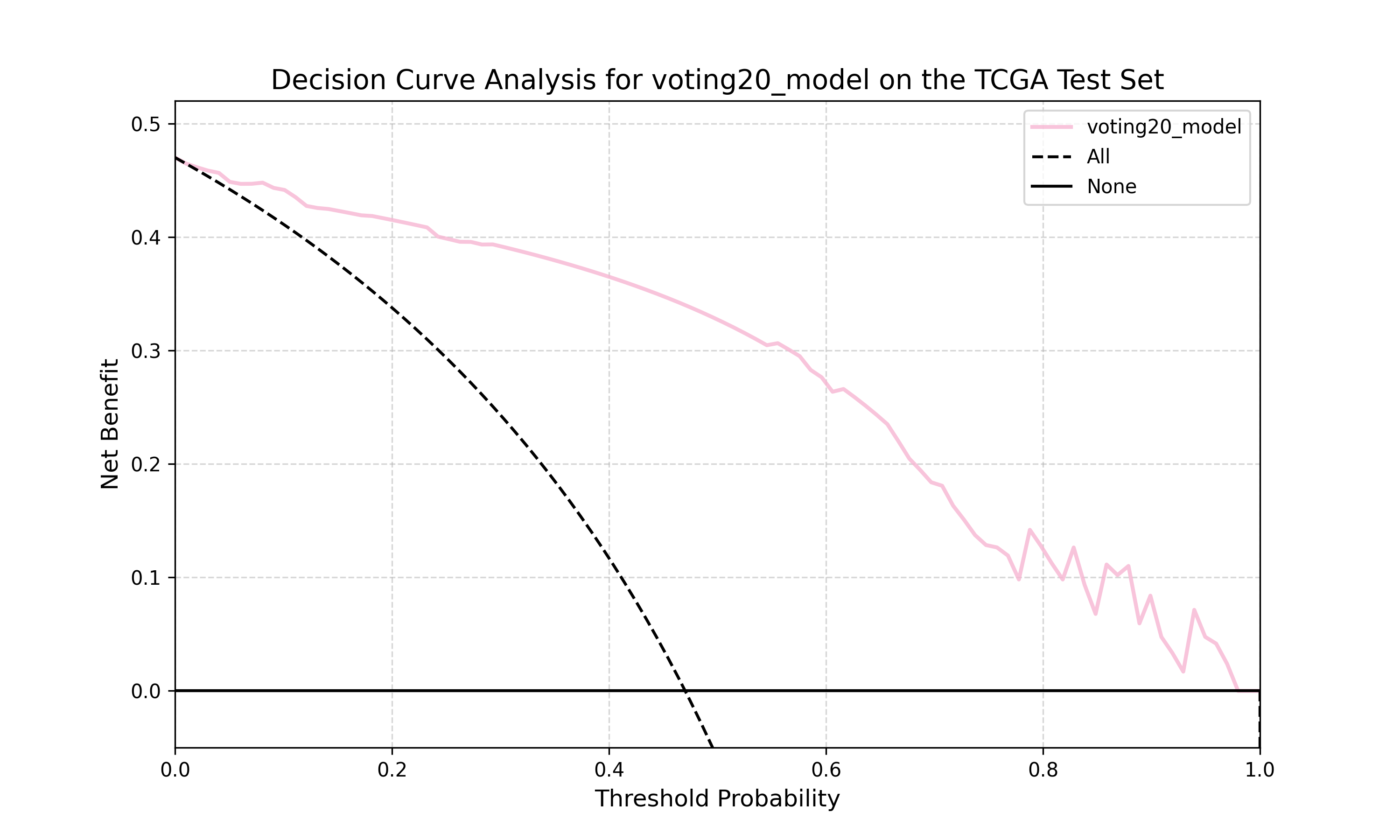

Supplement: S5 File — (ZIP) [file pone.0314831.s015.zip › S5 File/dca_curve_voting20_model.png]

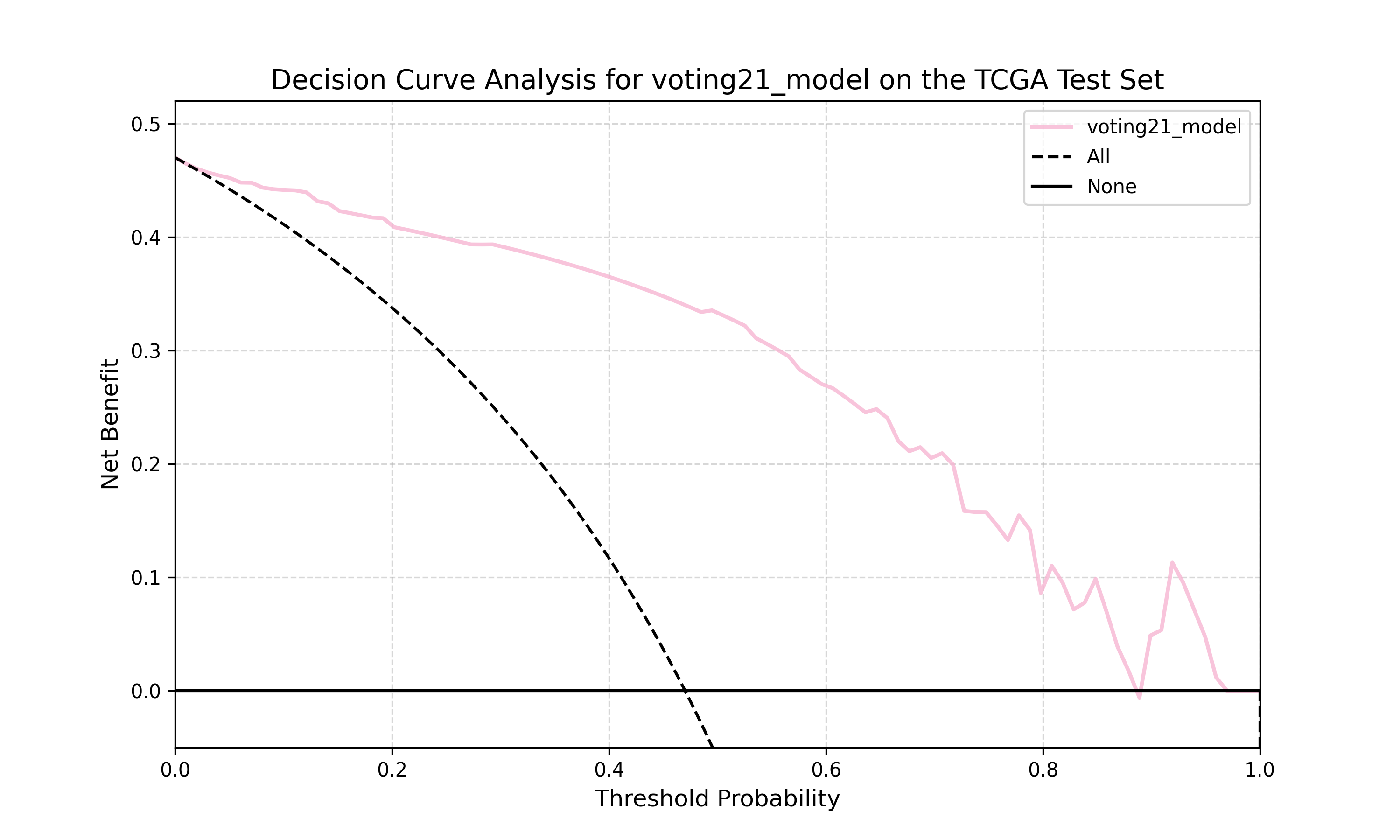

Supplement: S5 File — (ZIP) [file pone.0314831.s015.zip › S5 File/dca_curve_voting21_model.png]

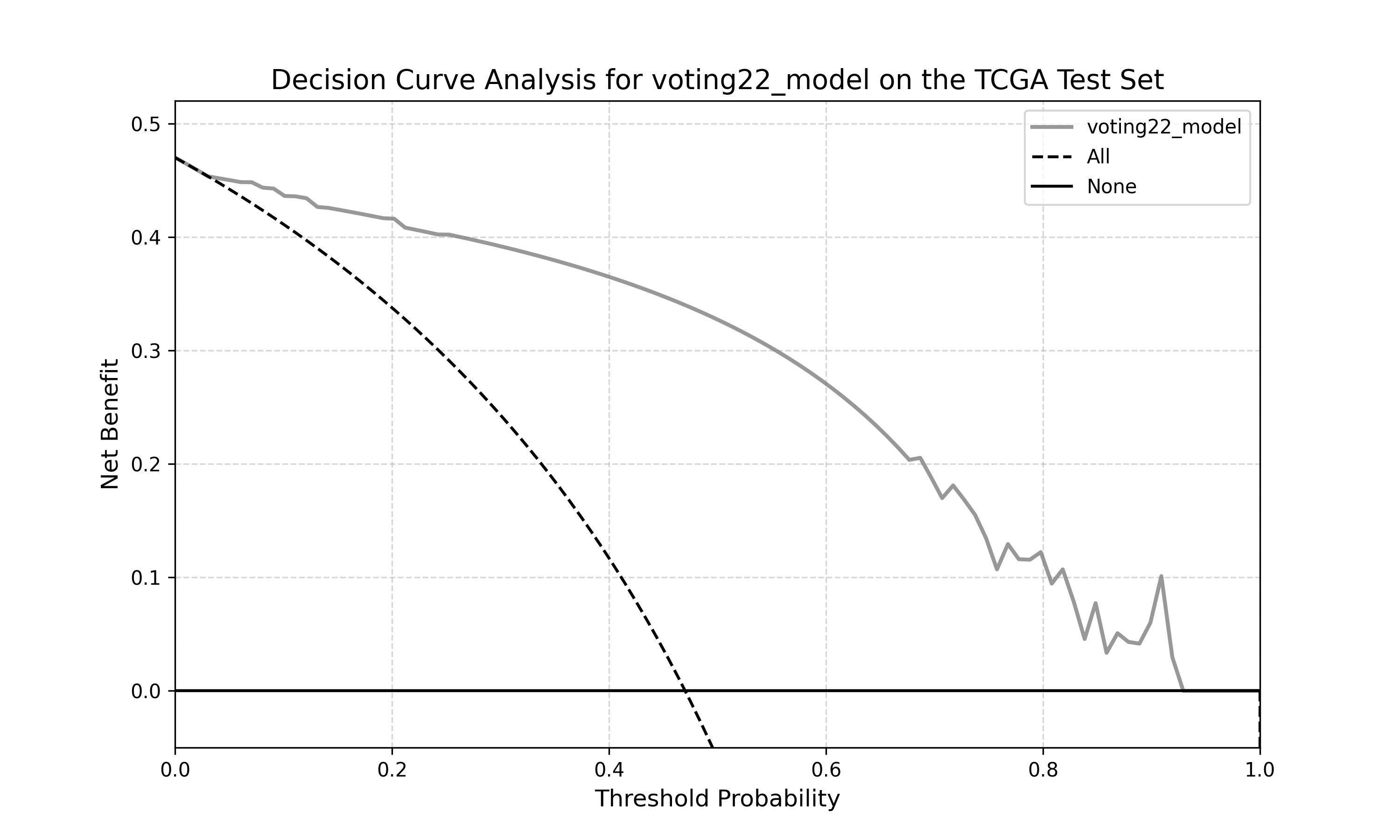

Supplement: S5 File — (ZIP) [file pone.0314831.s015.zip › S5 File/dca_curve_voting22_model.png]

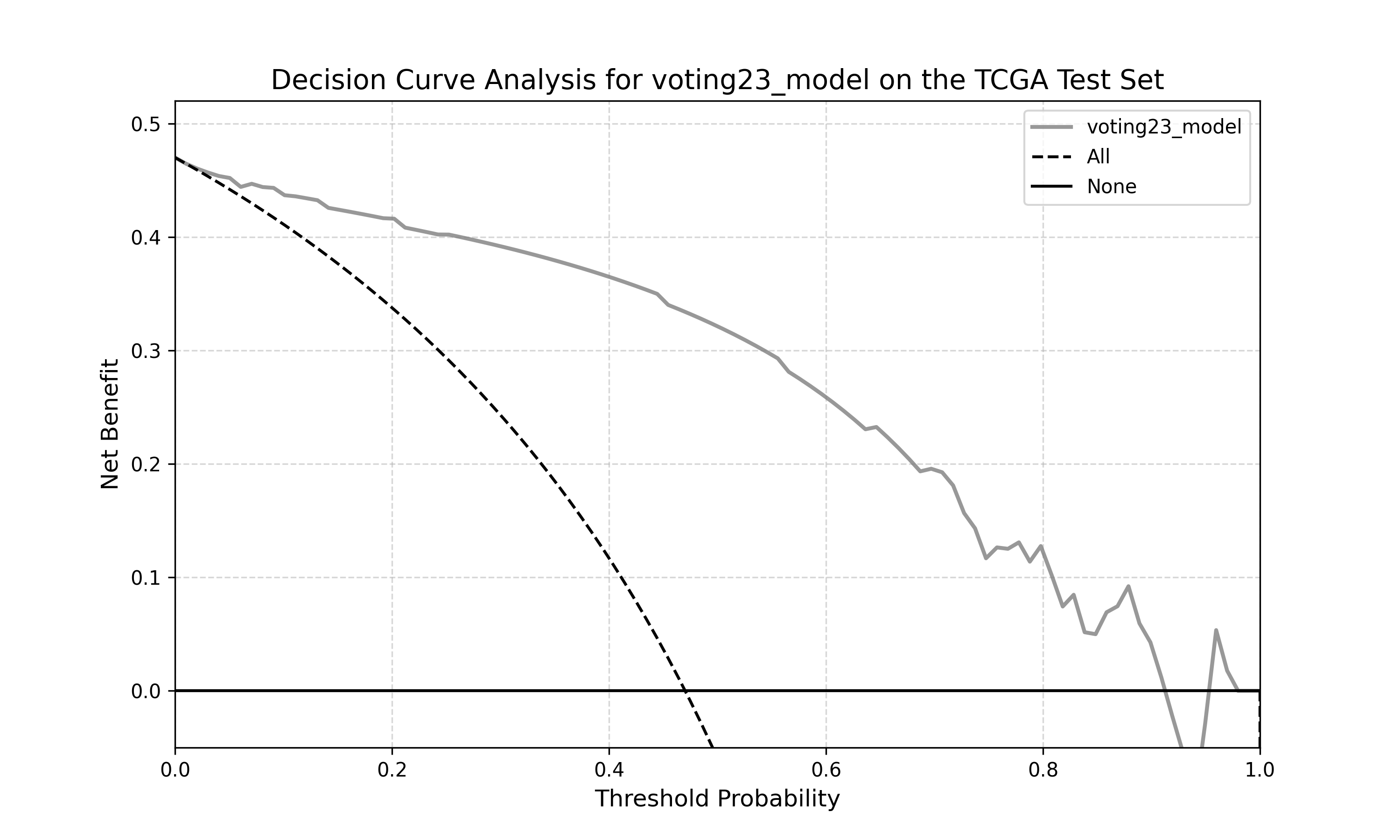

Supplement: S5 File — (ZIP) [file pone.0314831.s015.zip › S5 File/dca_curve_voting23_model.png]

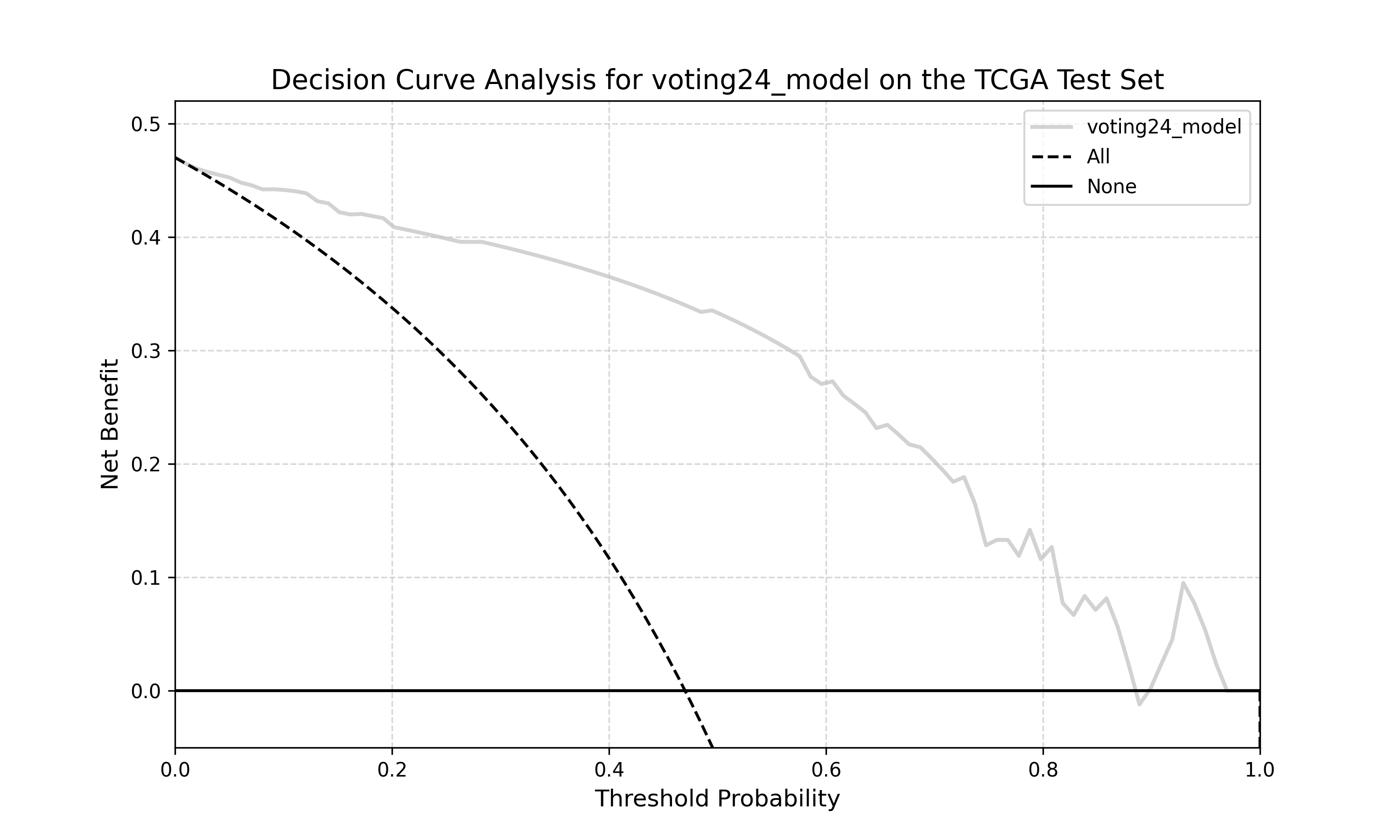

Supplement: S5 File — (ZIP) [file pone.0314831.s015.zip › S5 File/dca_curve_voting24_model.png]

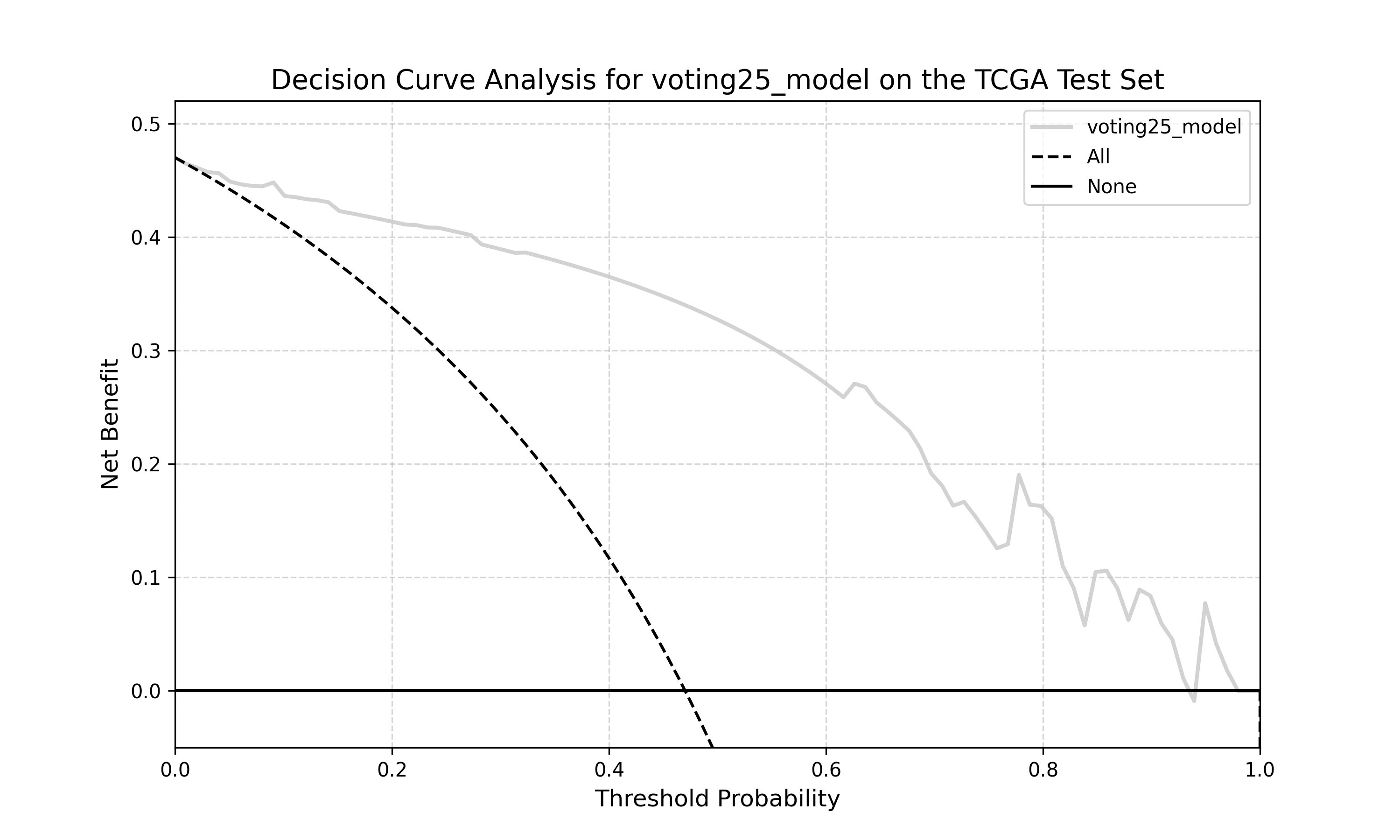

Supplement: S5 File — (ZIP) [file pone.0314831.s015.zip › S5 File/dca_curve_voting25_model.png]

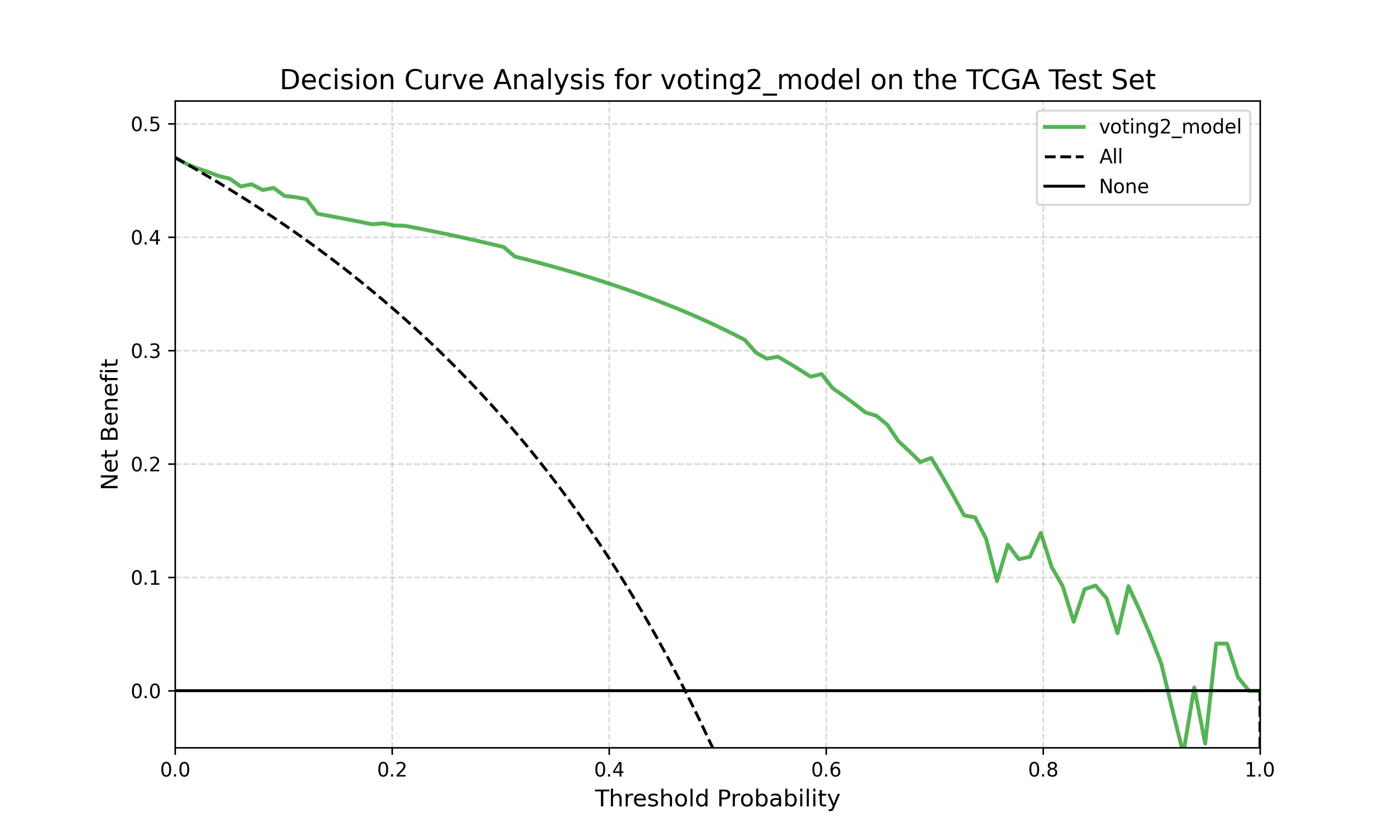

Supplement: S5 File — (ZIP) [file pone.0314831.s015.zip › S5 File/dca_curve_voting2_model.png]

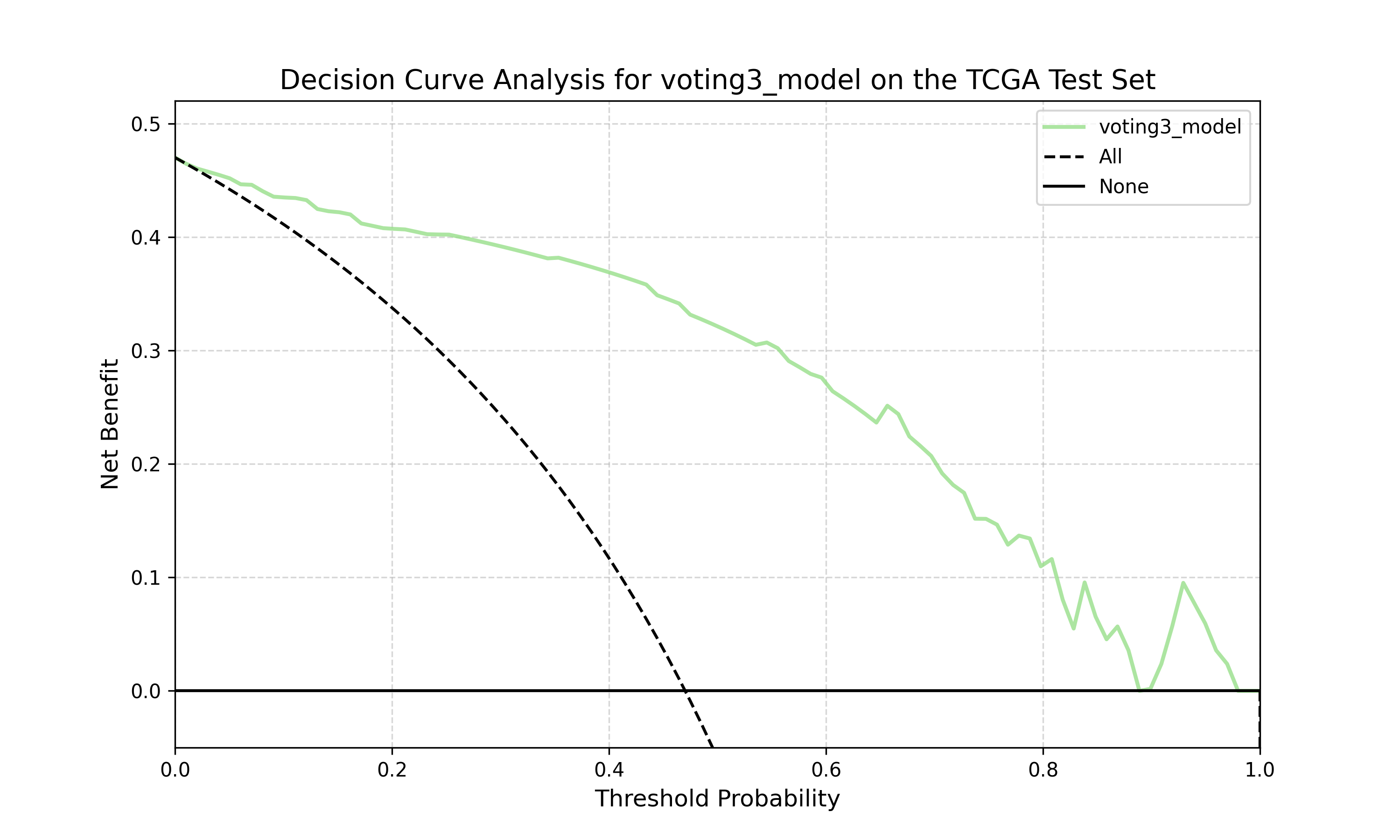

Supplement: S5 File — (ZIP) [file pone.0314831.s015.zip › S5 File/dca_curve_voting3_model.png]

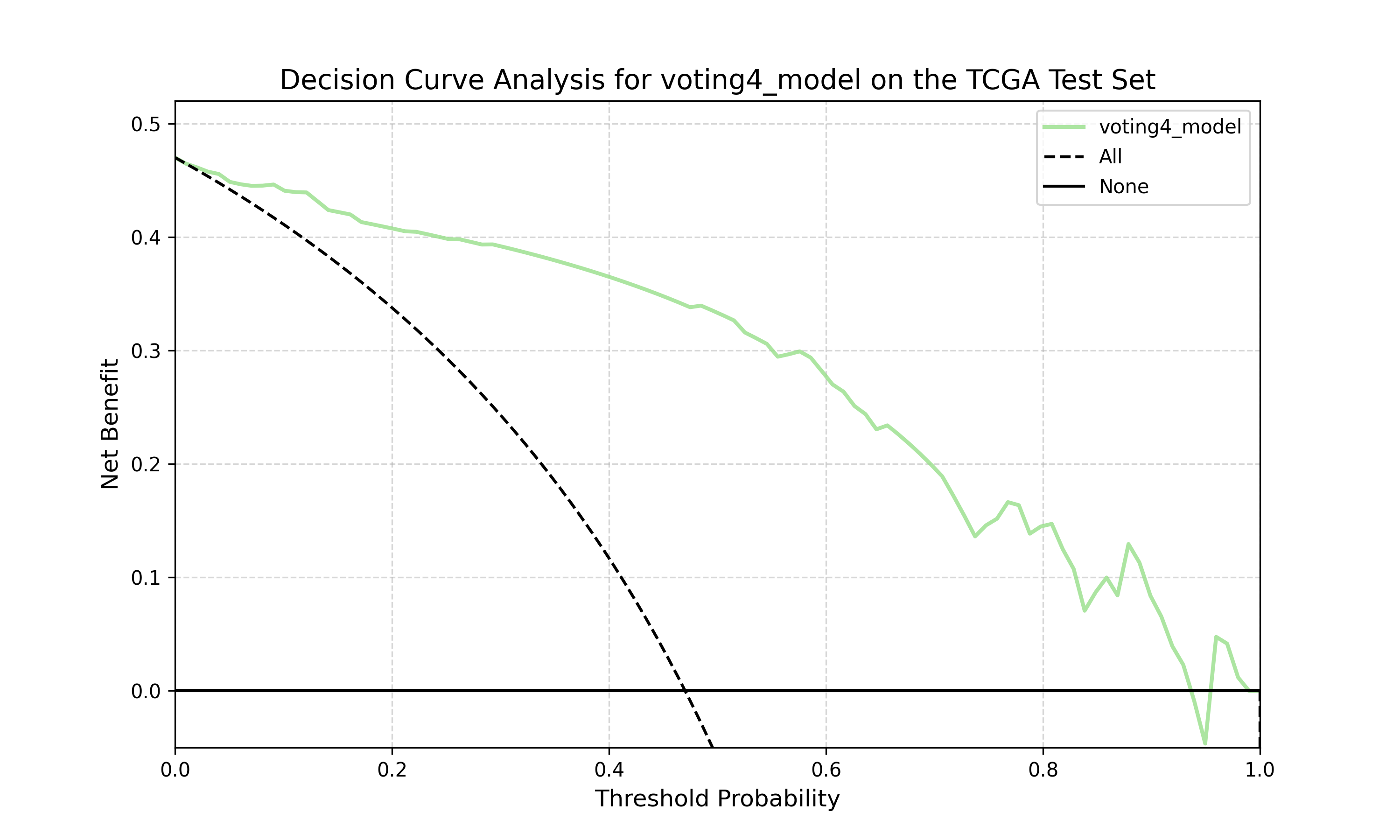

Supplement: S5 File — (ZIP) [file pone.0314831.s015.zip › S5 File/dca_curve_voting4_model.png]

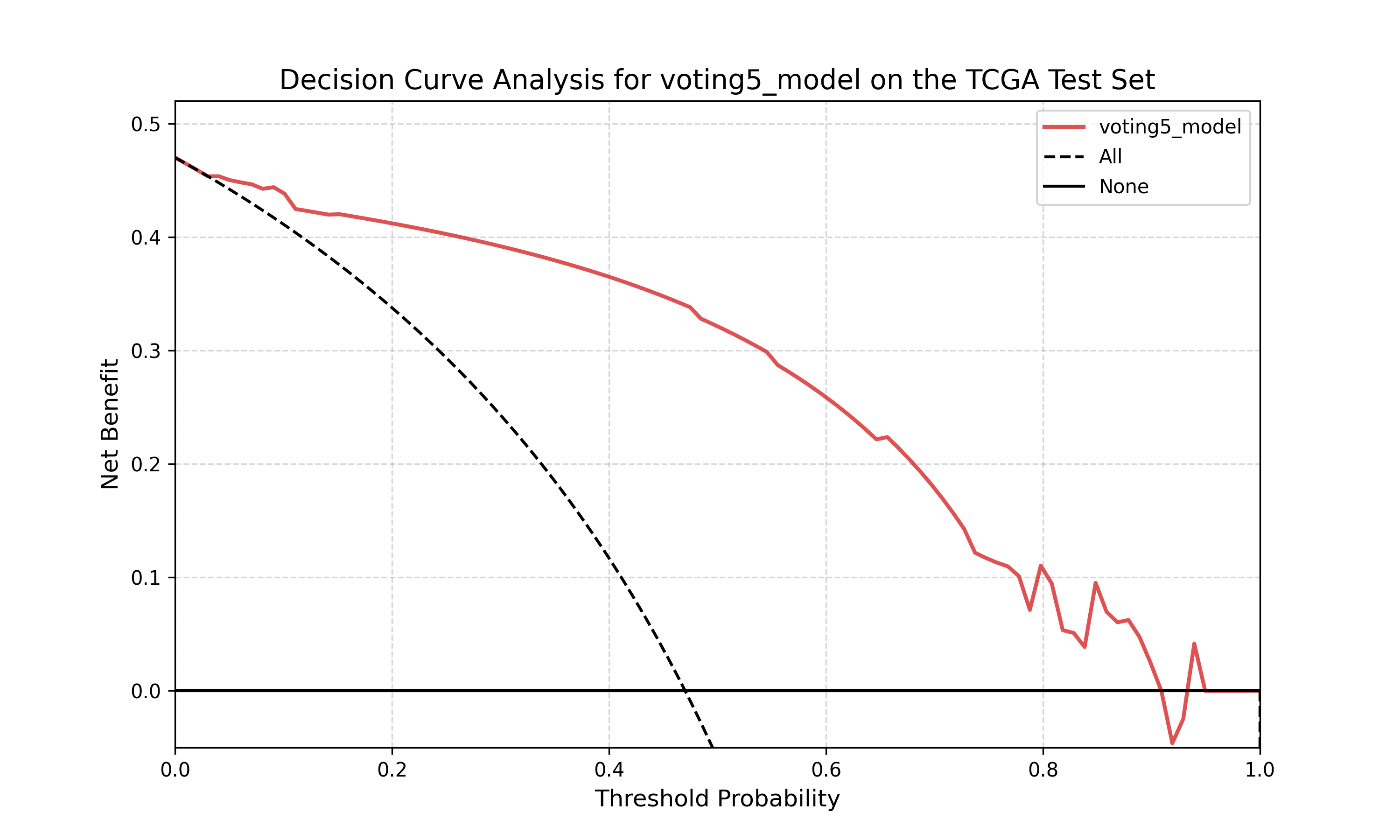

Supplement: S5 File — (ZIP) [file pone.0314831.s015.zip › S5 File/dca_curve_voting5_model.png]

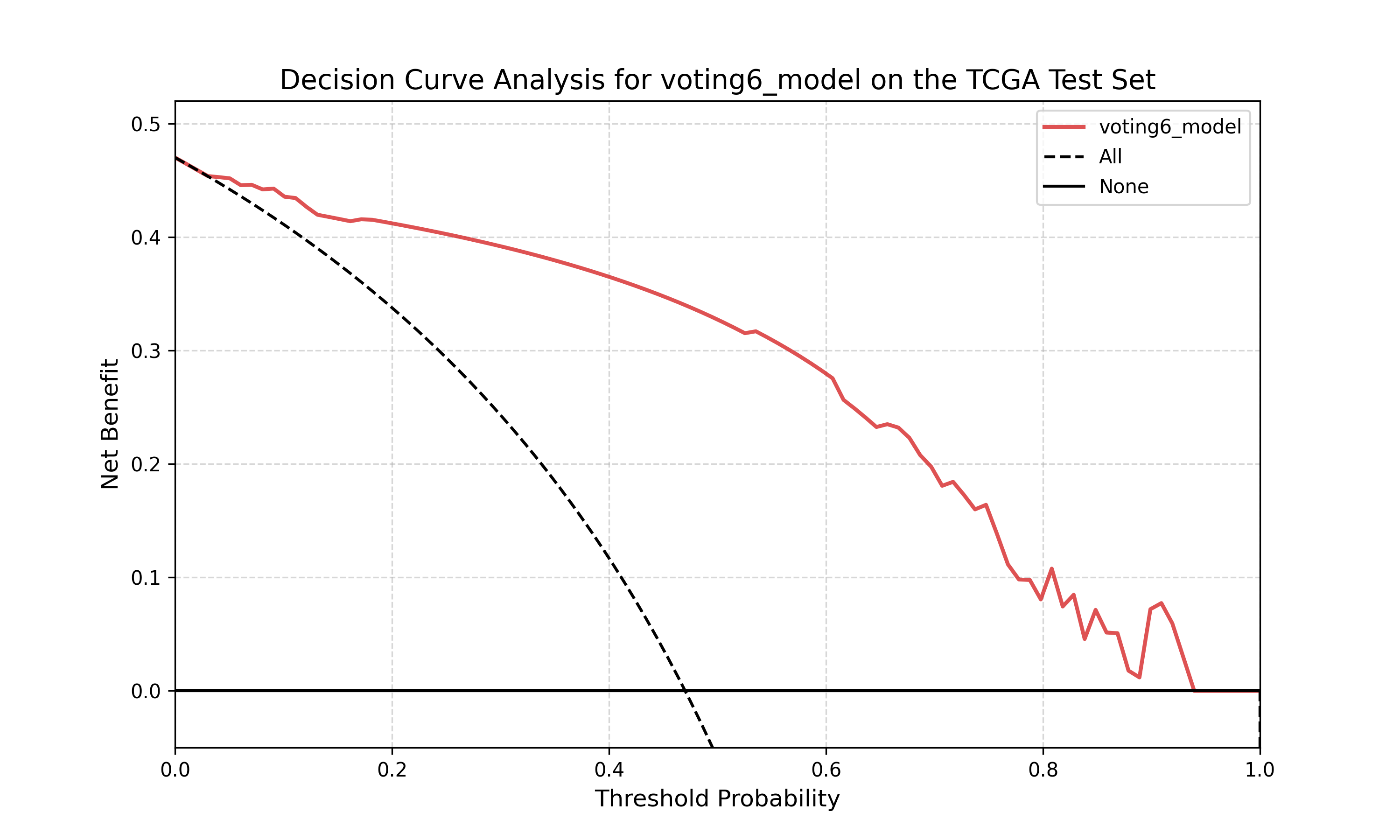

Supplement: S5 File — (ZIP) [file pone.0314831.s015.zip › S5 File/dca_curve_voting6_model.png]

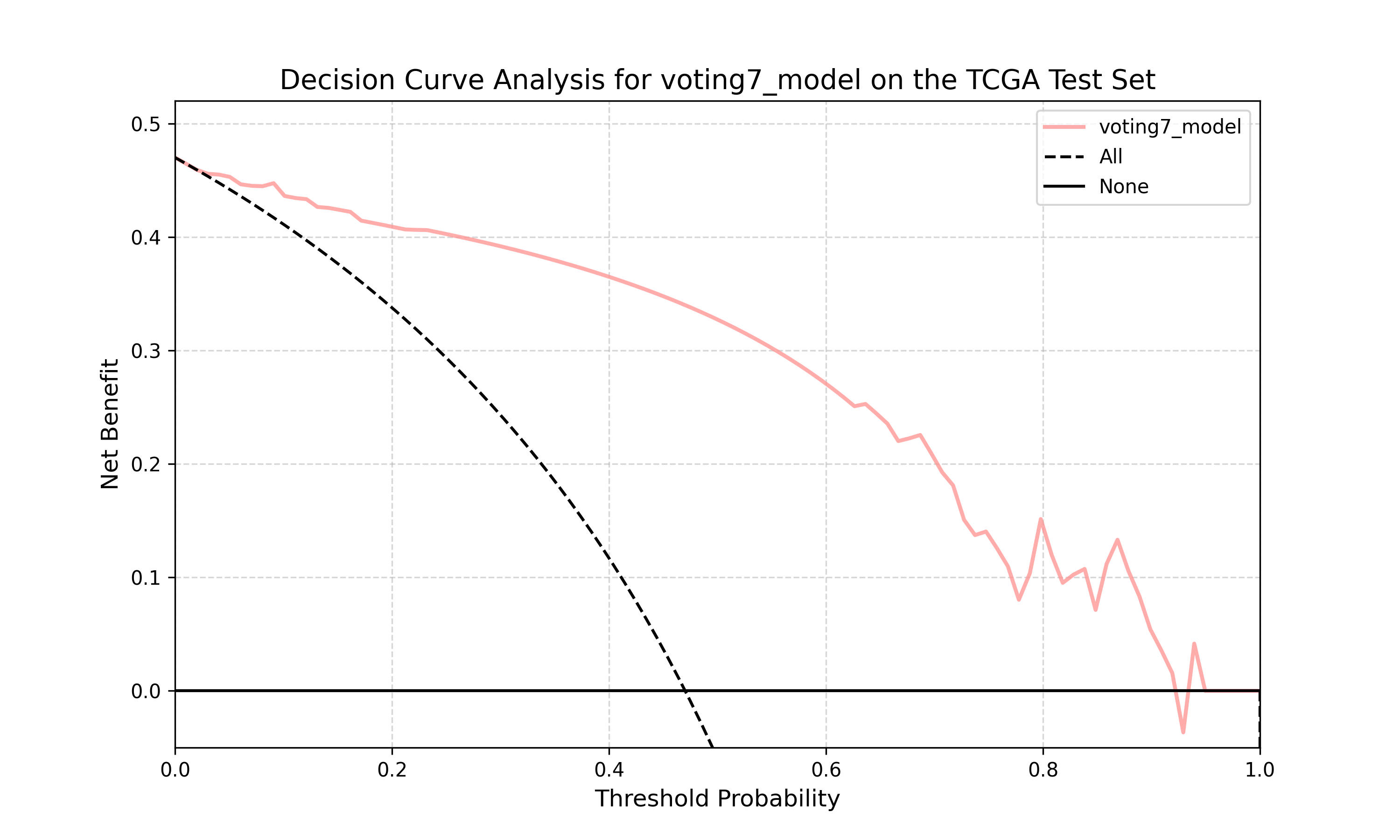

Supplement: S5 File — (ZIP) [file pone.0314831.s015.zip › S5 File/dca_curve_voting7_model.png]

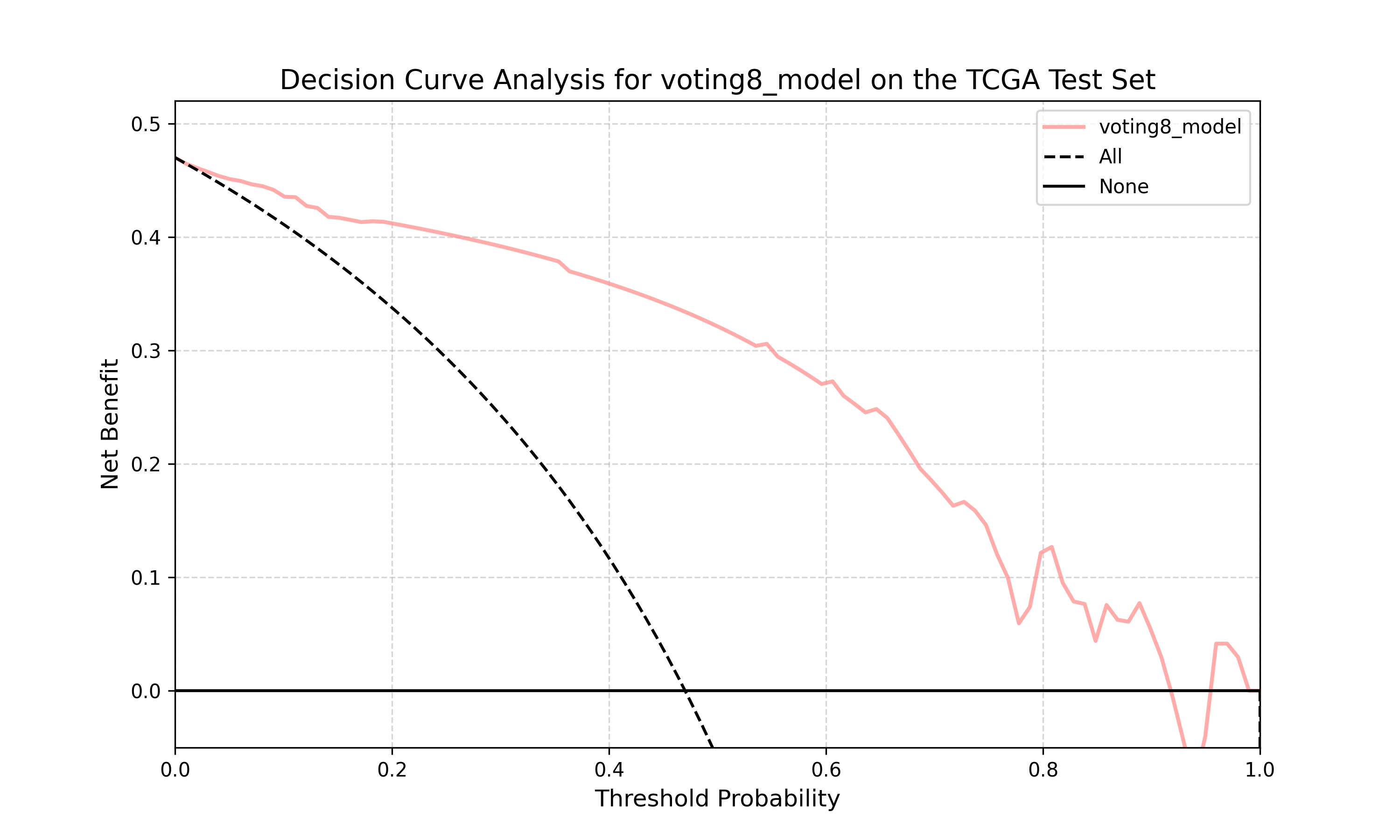

Supplement: S5 File — (ZIP) [file pone.0314831.s015.zip › S5 File/dca_curve_voting8_model.png]

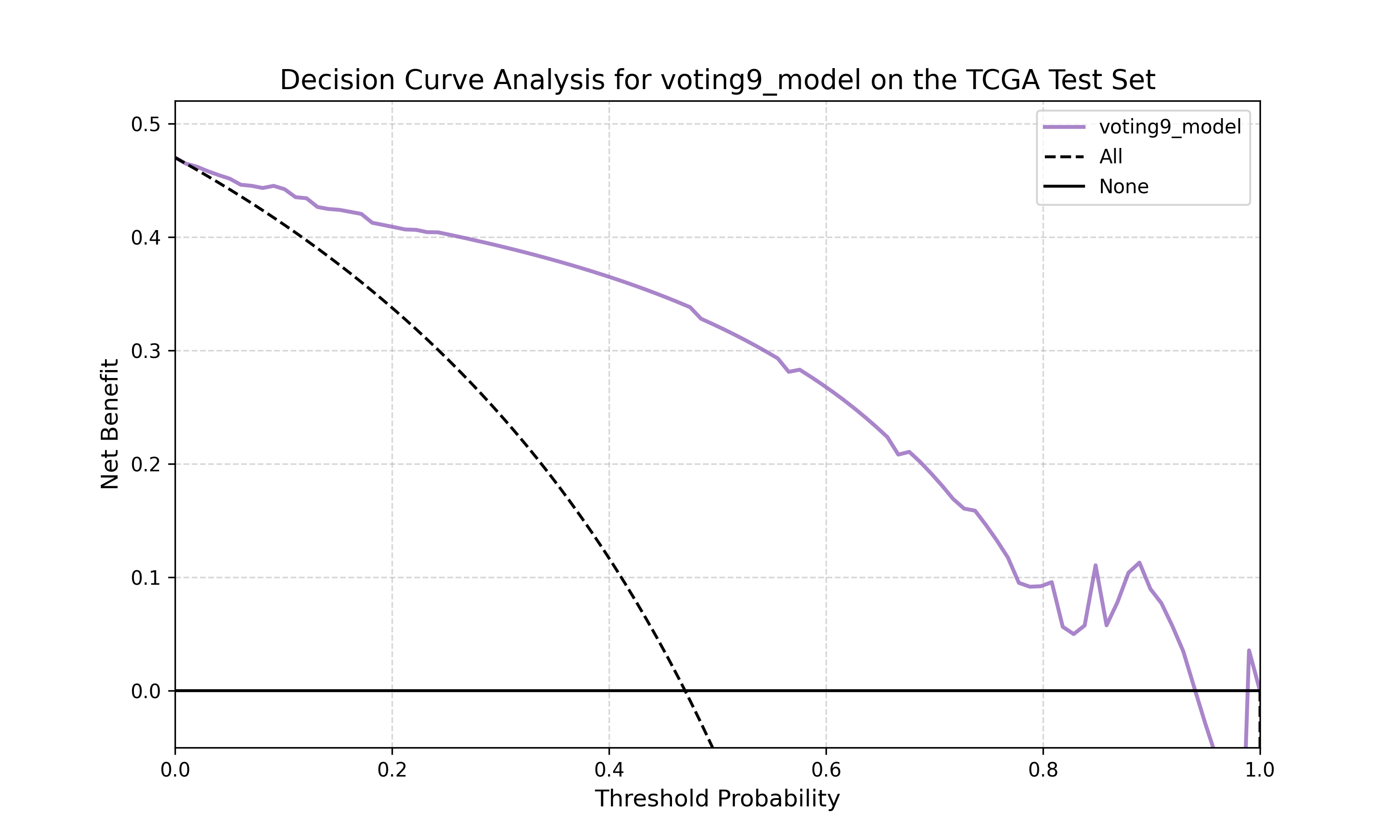

Supplement: S5 File — (ZIP) [file pone.0314831.s015.zip › S5 File/dca_curve_voting9_model.png]

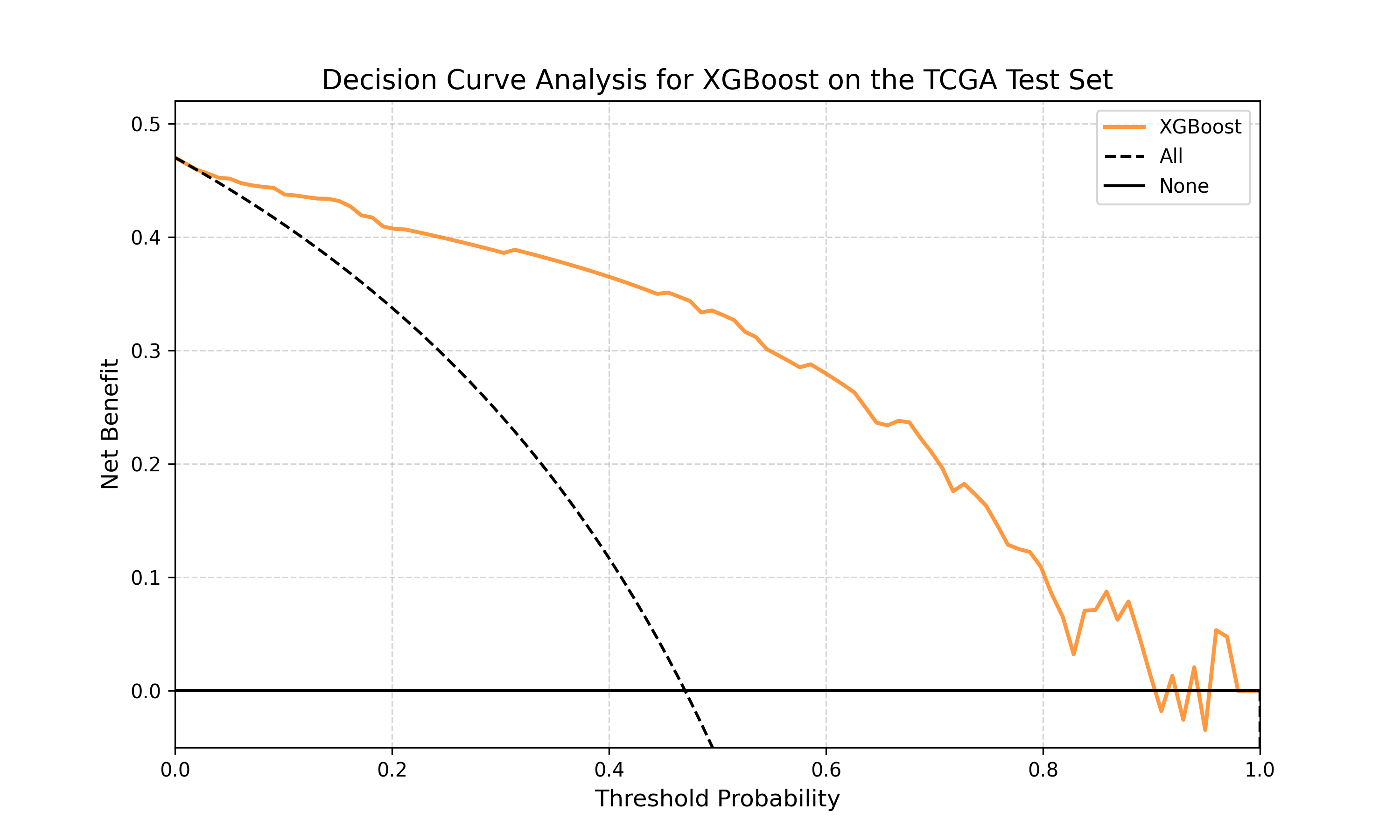

Supplement: S5 File — (ZIP) [file pone.0314831.s015.zip › S5 File/dca_curve_XGBoost.png]

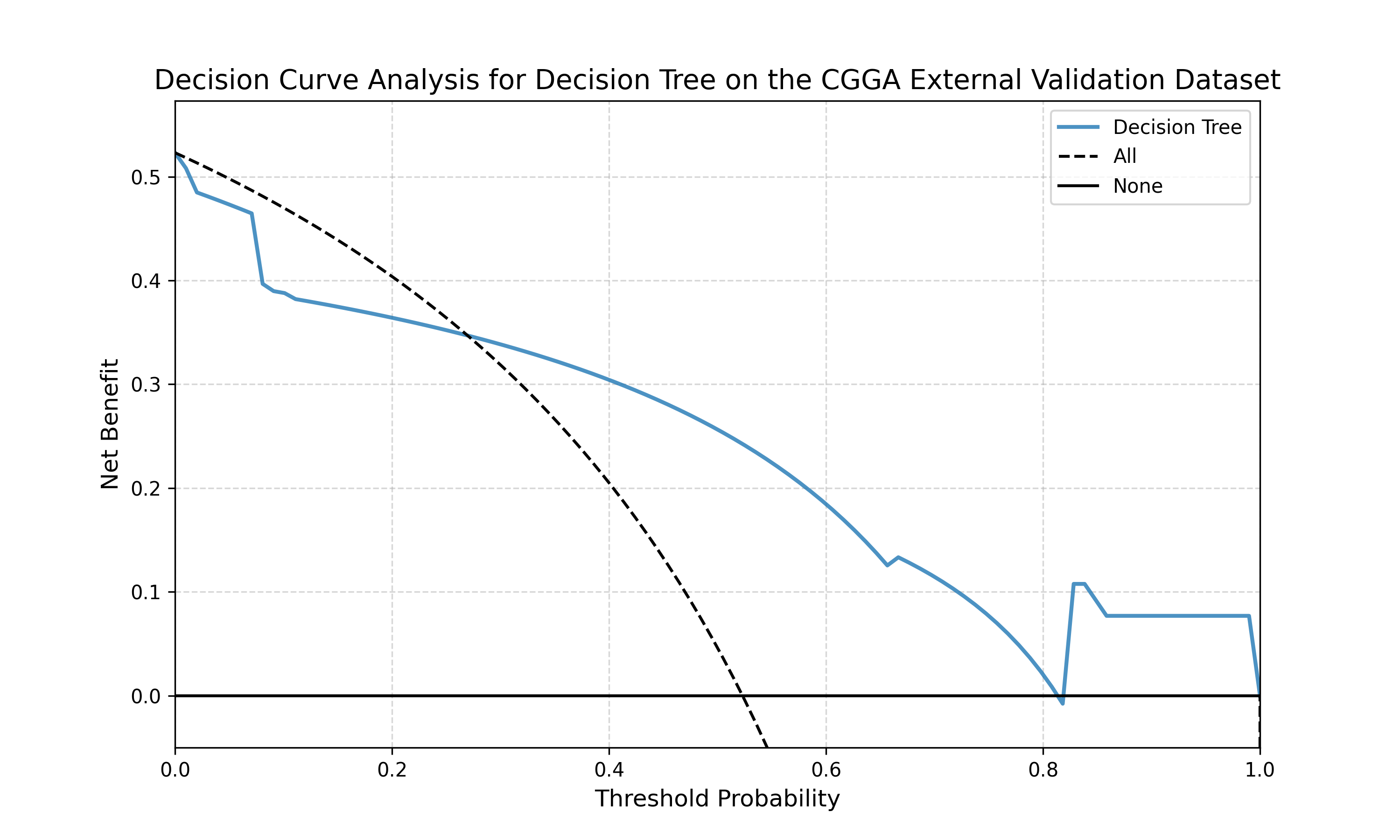

Supplement: S6 File — (ZIP) [file pone.0314831.s016.zip › S6 File/dca_curve_Decision Tree.png]

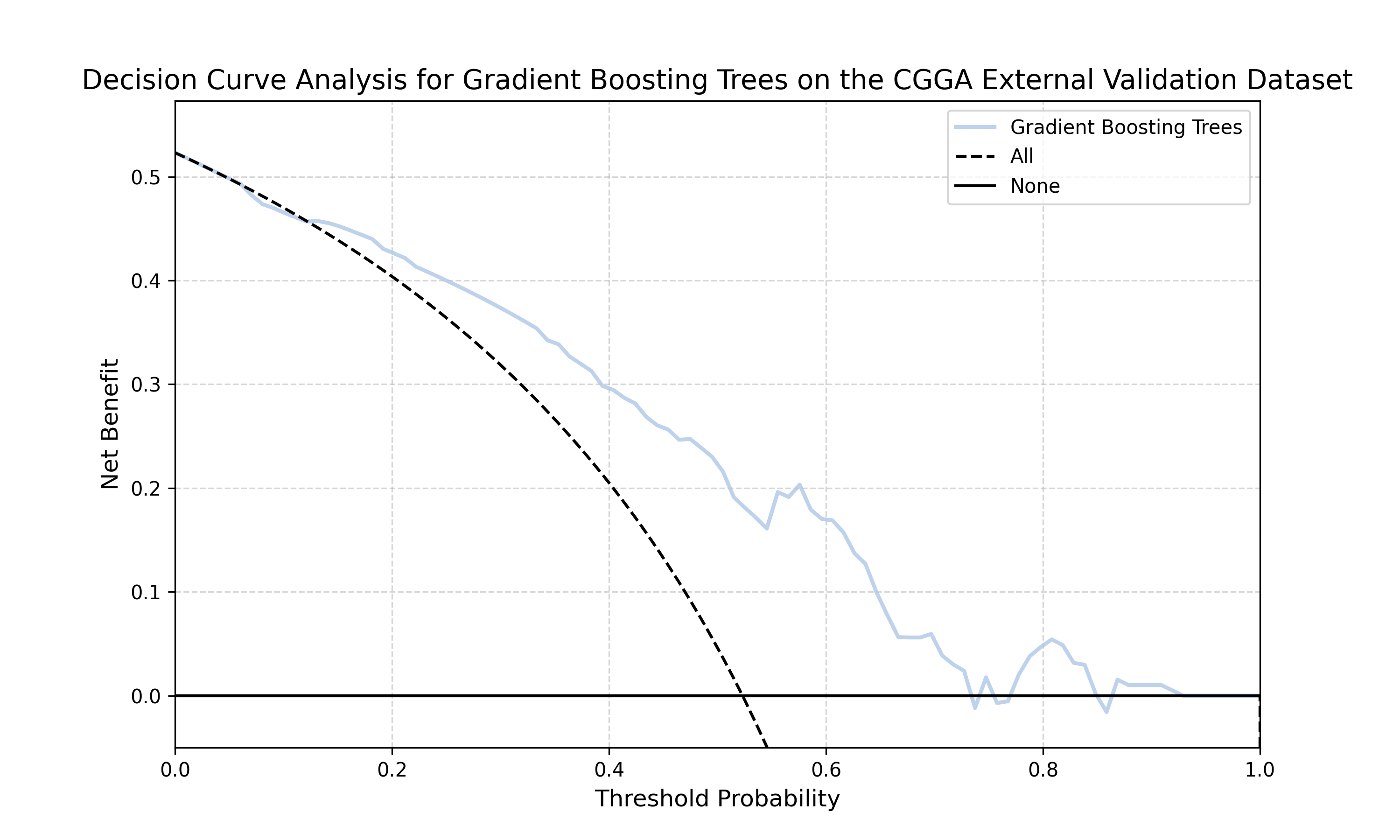

Supplement: S6 File — (ZIP) [file pone.0314831.s016.zip › S6 File/dca_curve_Gradient Boosting Trees.png]

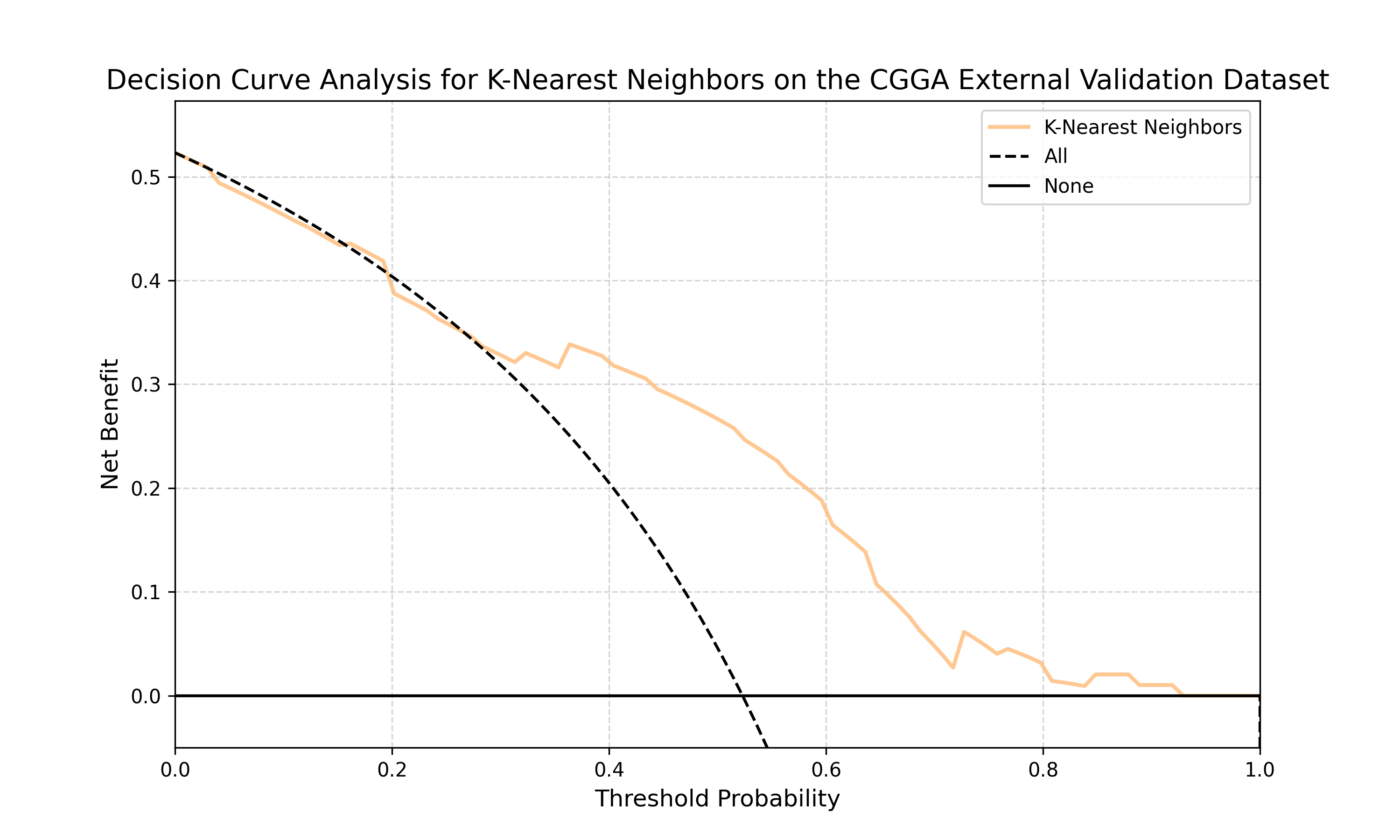

Supplement: S6 File — (ZIP) [file pone.0314831.s016.zip › S6 File/dca_curve_K-Nearest Neighbors.png]

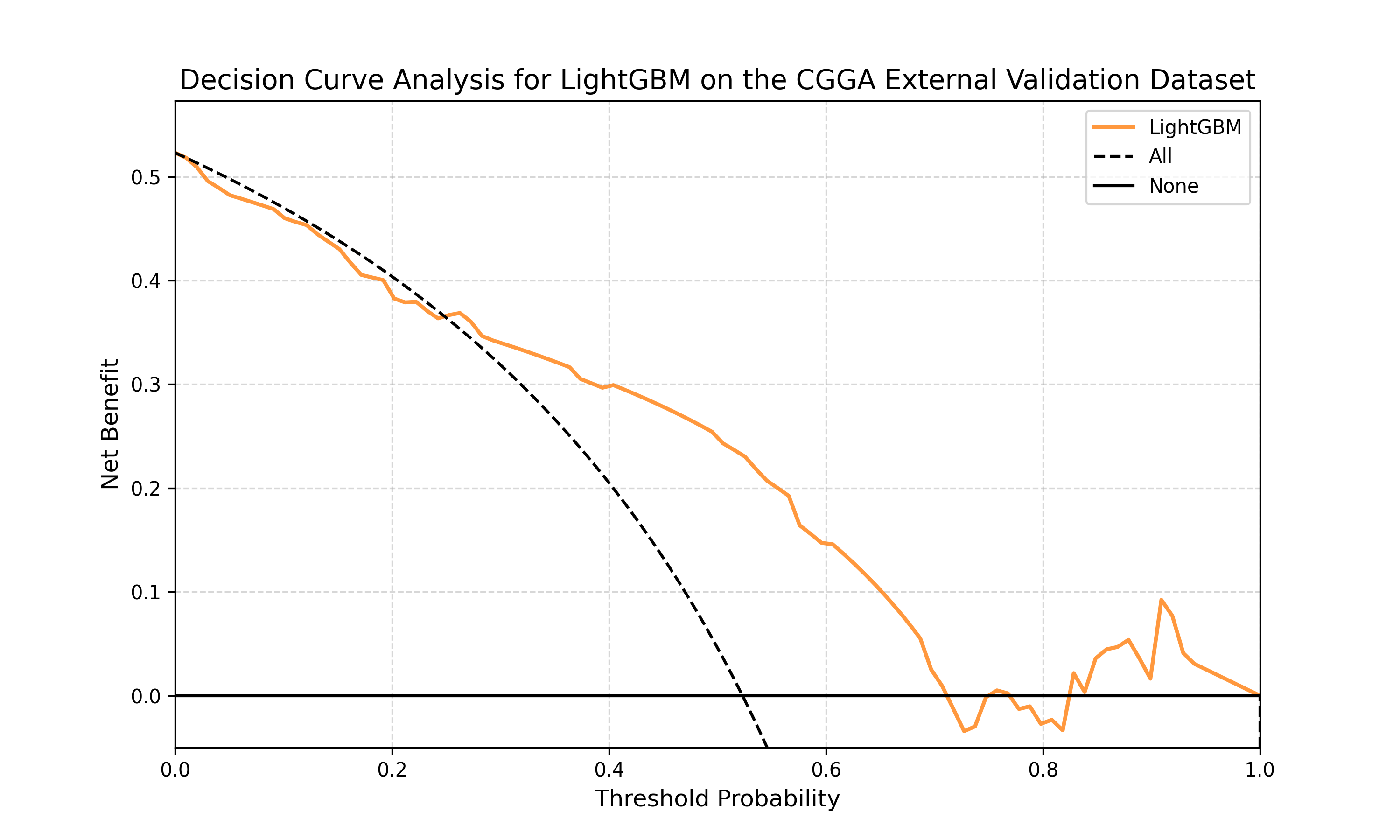

Supplement: S6 File — (ZIP) [file pone.0314831.s016.zip › S6 File/dca_curve_LightGBM.png]

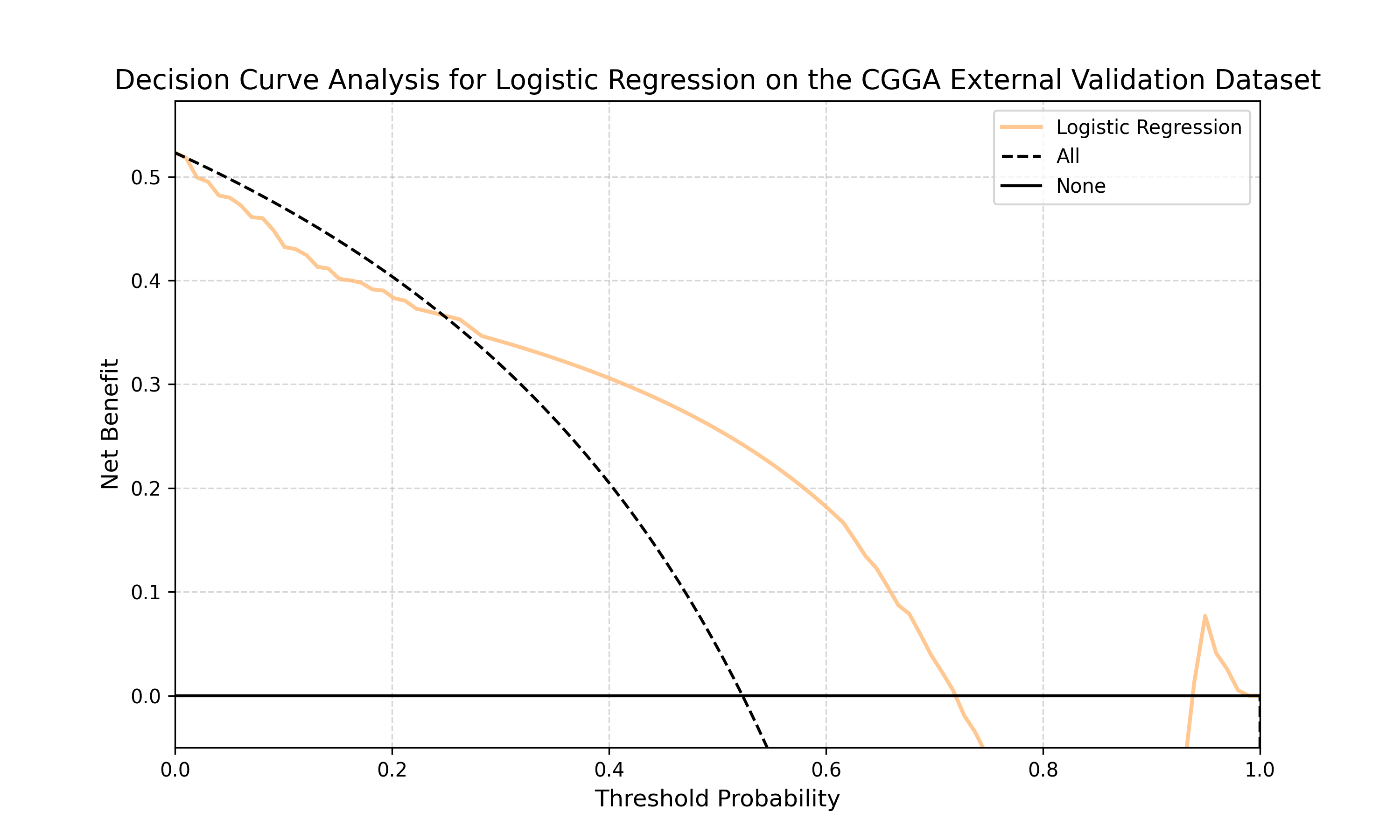

Supplement: S6 File — (ZIP) [file pone.0314831.s016.zip › S6 File/dca_curve_Logistic Regression.png]

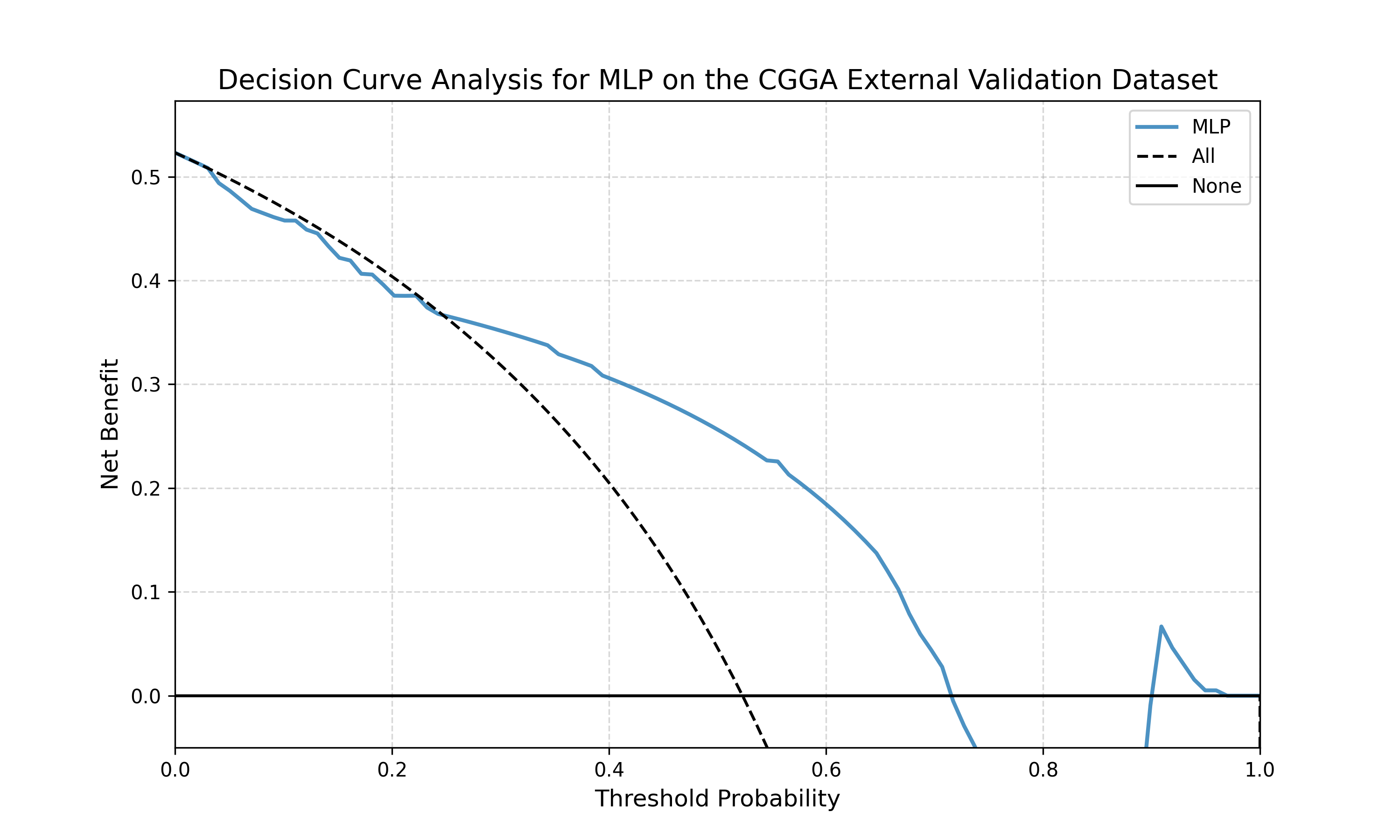

Supplement: S6 File — (ZIP) [file pone.0314831.s016.zip › S6 File/dca_curve_MLP.png]

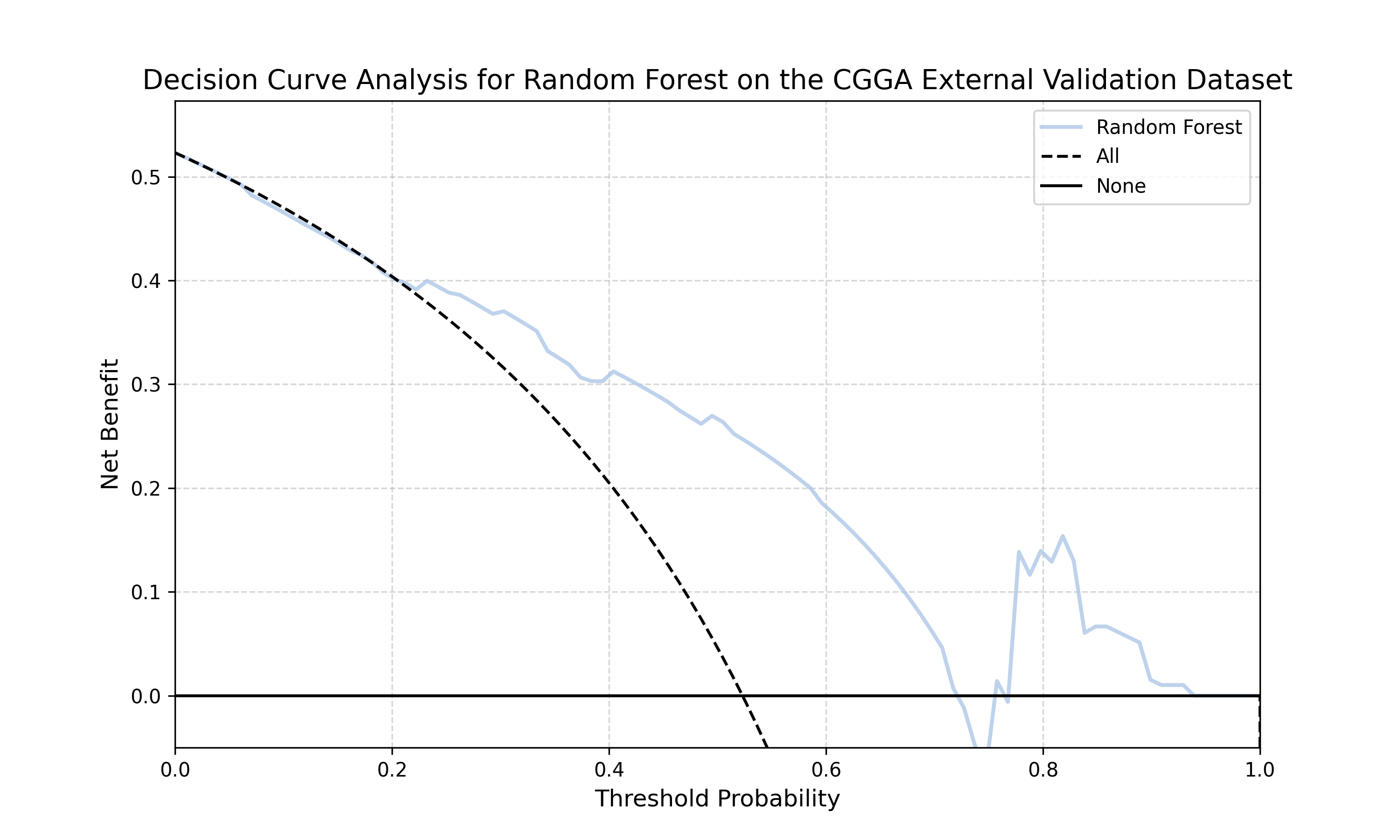

Supplement: S6 File — (ZIP) [file pone.0314831.s016.zip › S6 File/dca_curve_Random Forest.png]

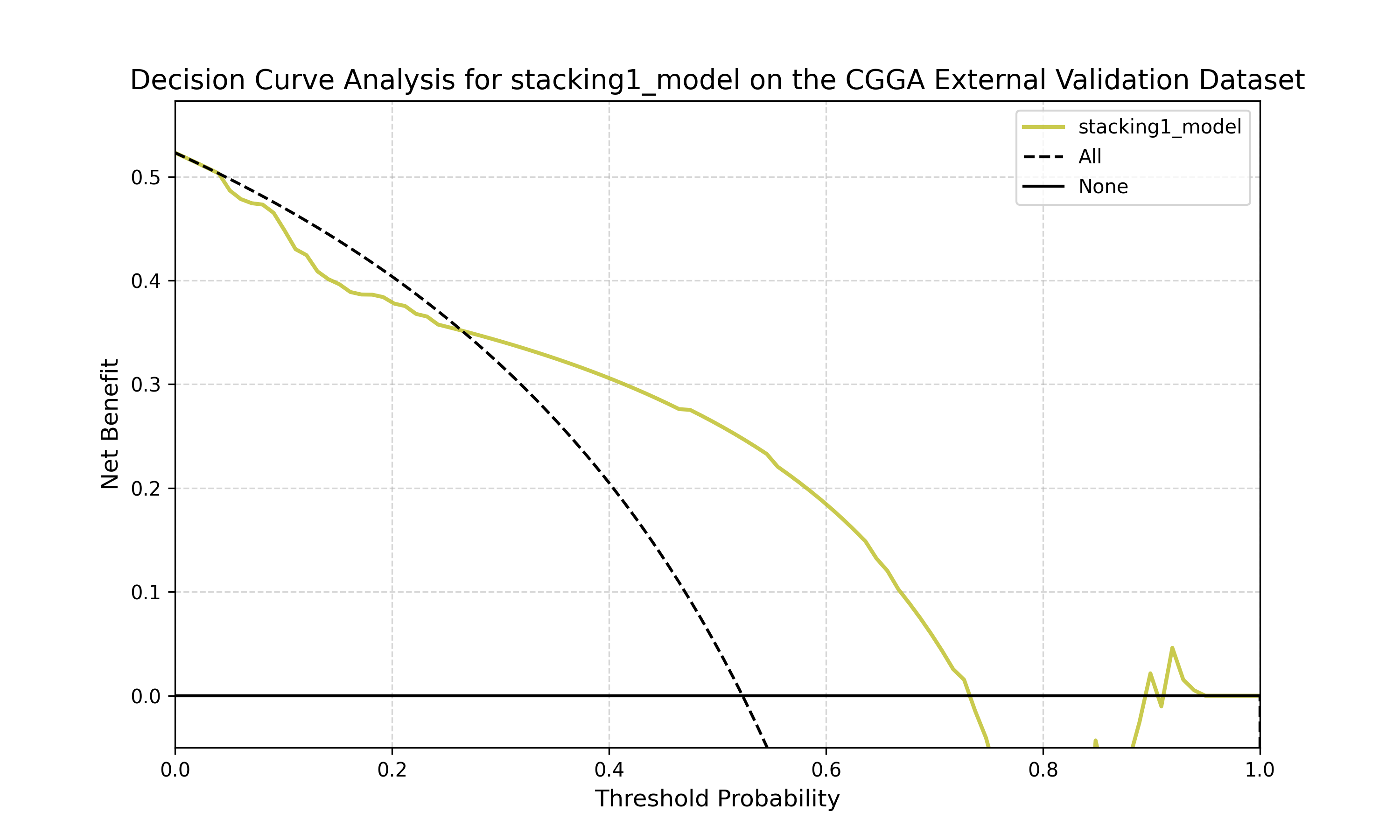

Supplement: S6 File — (ZIP) [file pone.0314831.s016.zip › S6 File/dca_curve_stacking1_model.png]

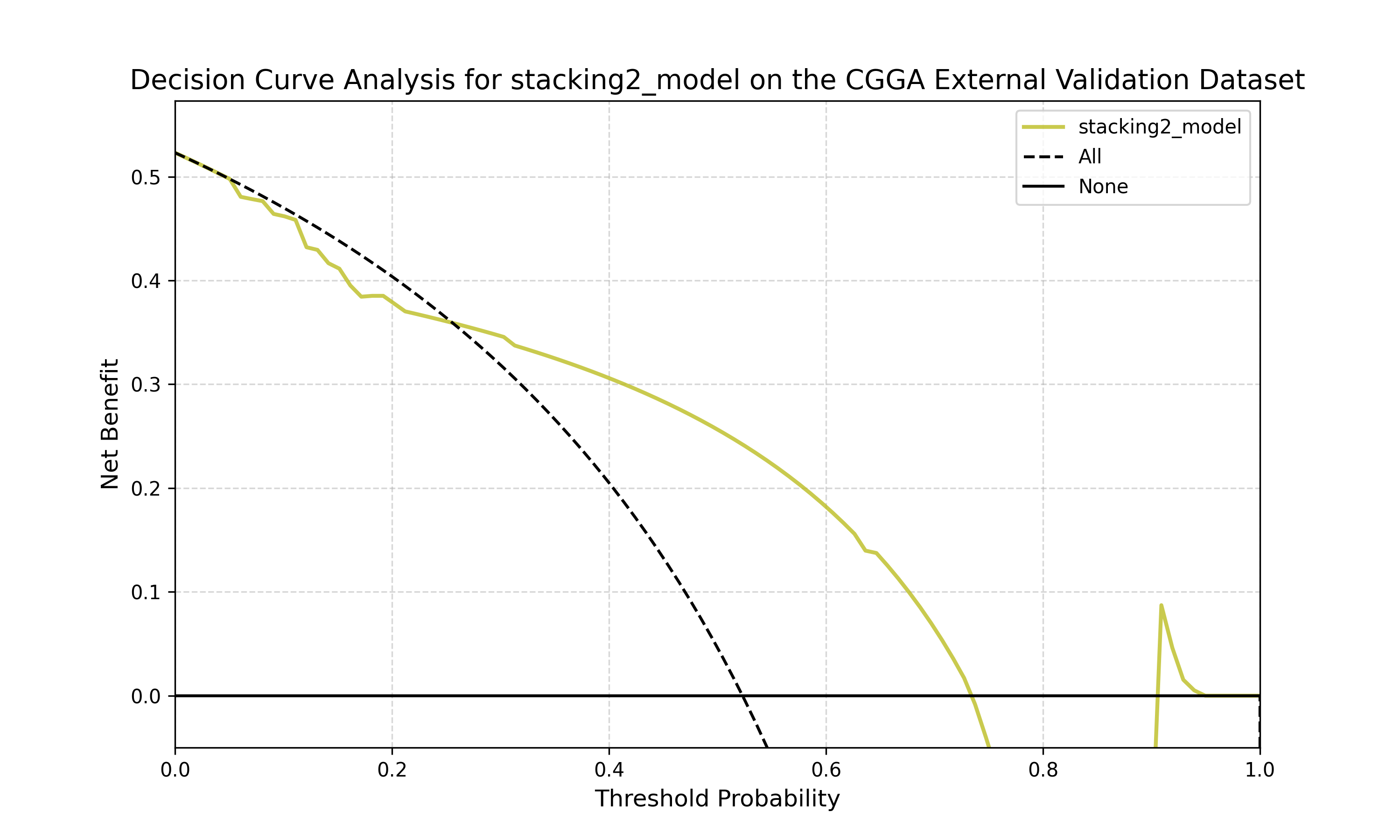

Supplement: S6 File — (ZIP) [file pone.0314831.s016.zip › S6 File/dca_curve_stacking2_model.png]

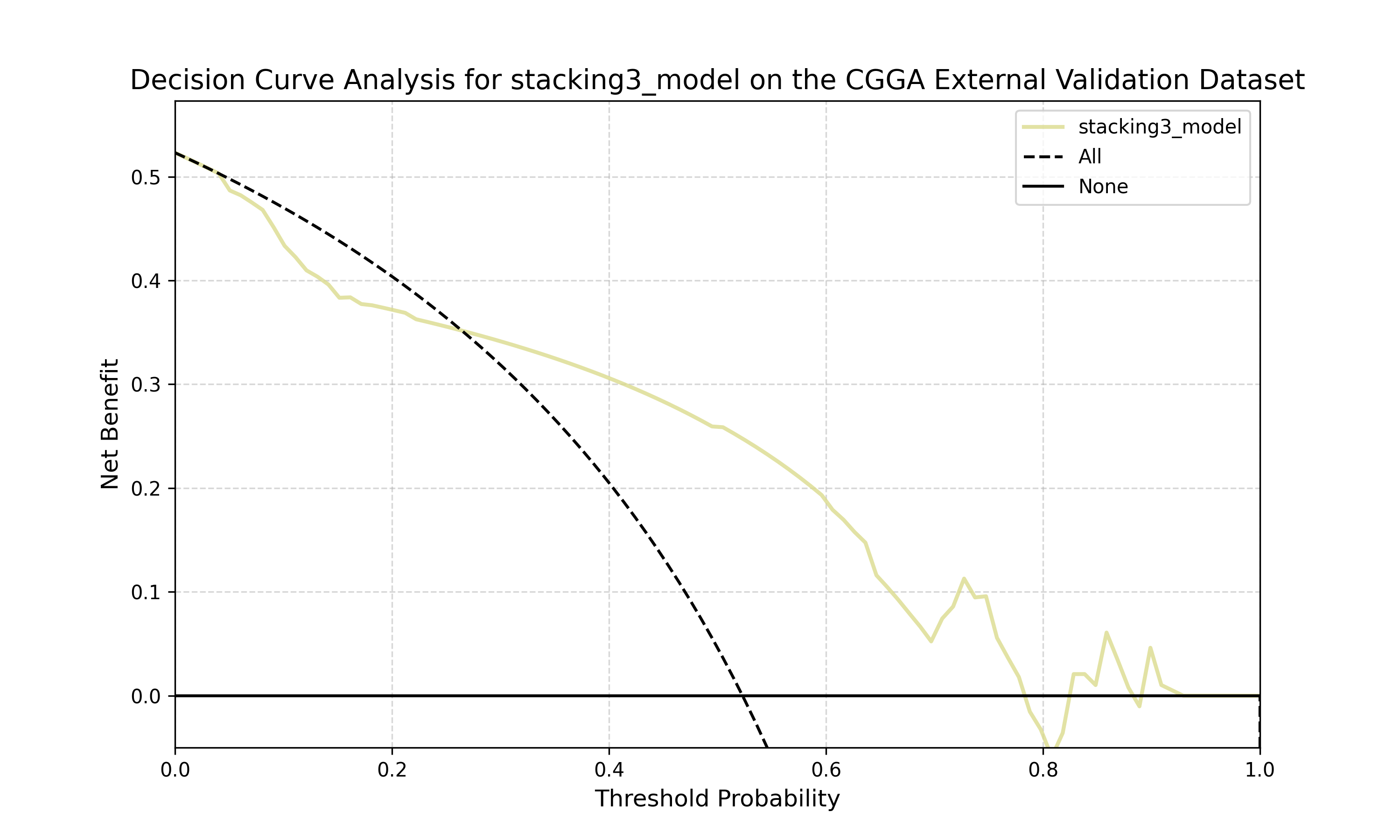

Supplement: S6 File — (ZIP) [file pone.0314831.s016.zip › S6 File/dca_curve_stacking3_model.png]

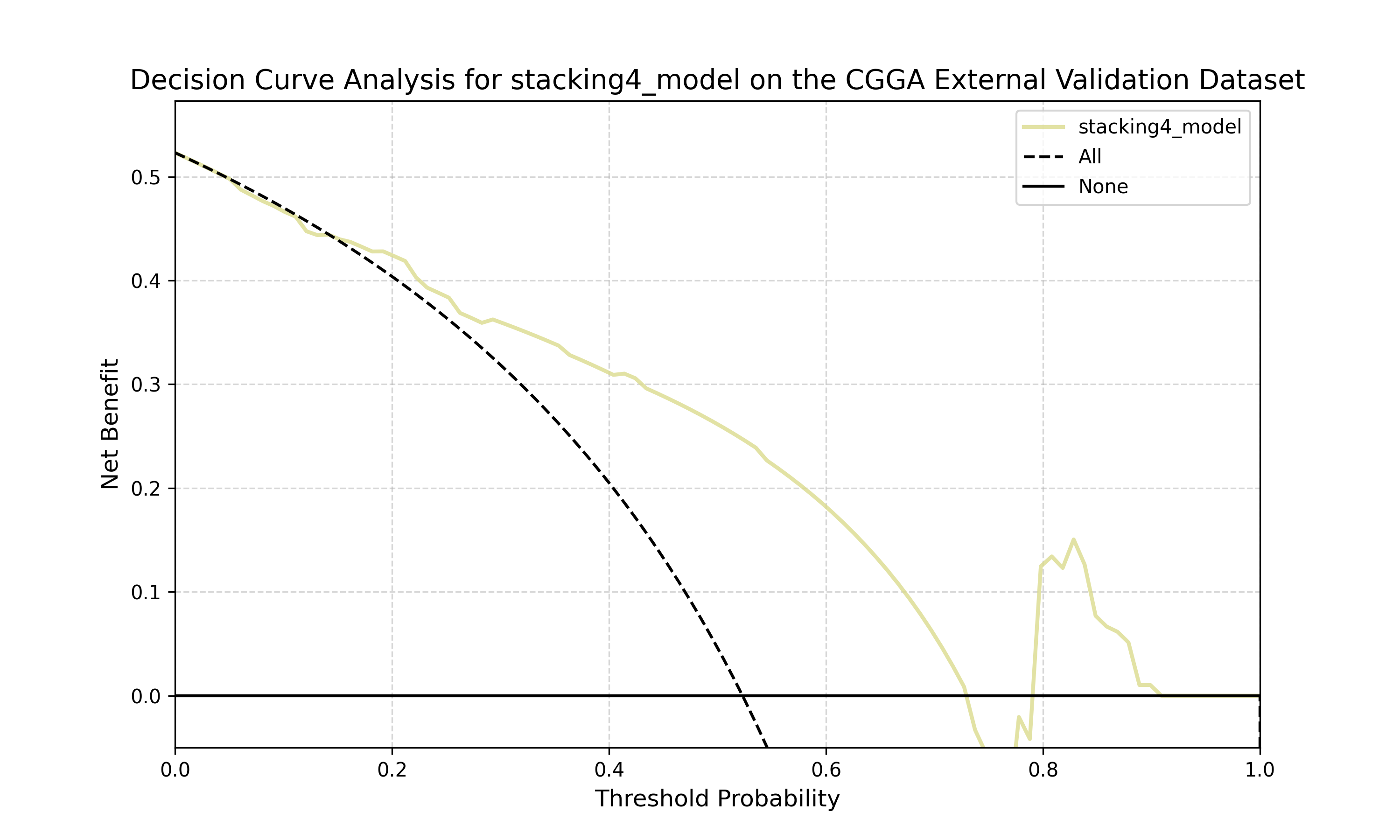

Supplement: S6 File — (ZIP) [file pone.0314831.s016.zip › S6 File/dca_curve_stacking4_model.png]

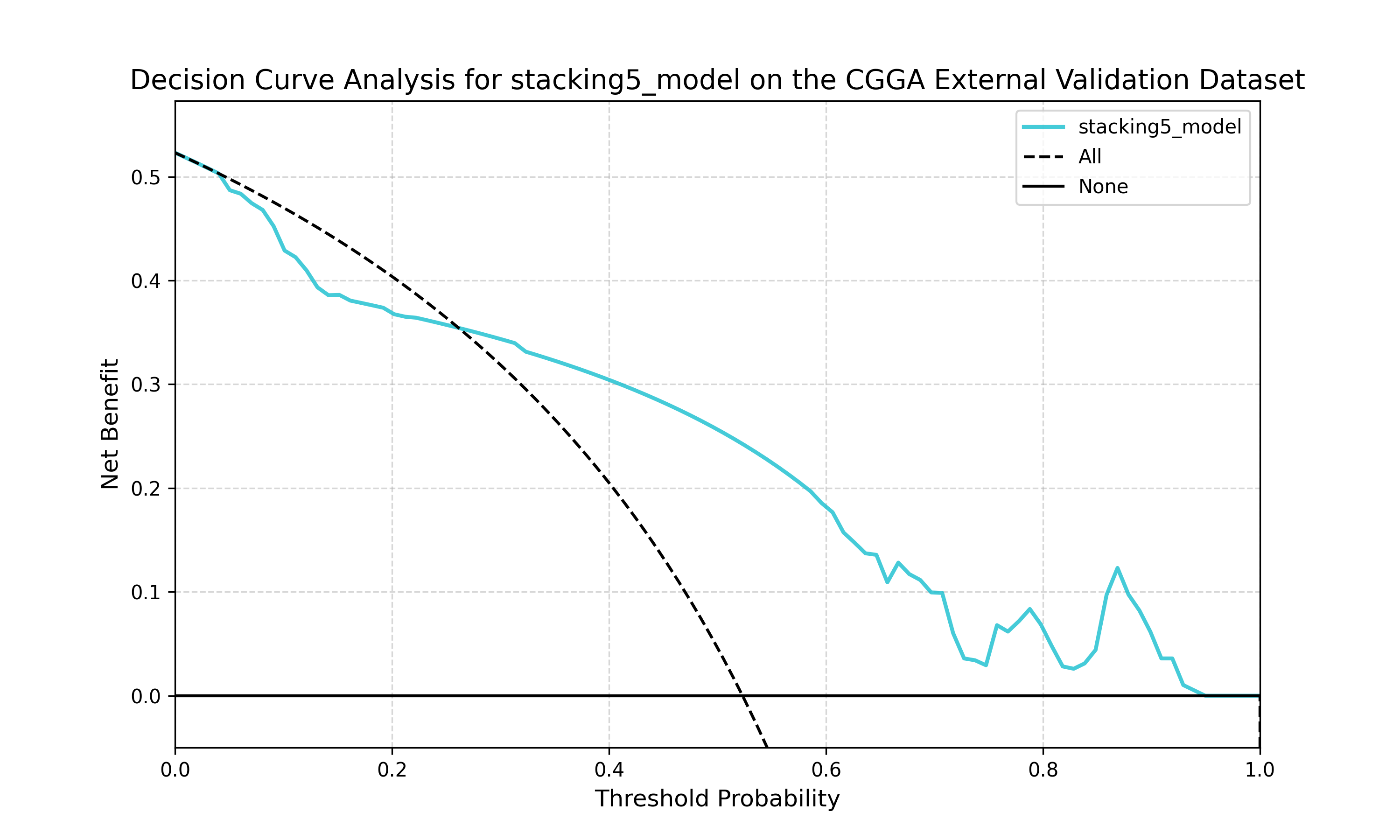

Supplement: S6 File — (ZIP) [file pone.0314831.s016.zip › S6 File/dca_curve_stacking5_model.png]

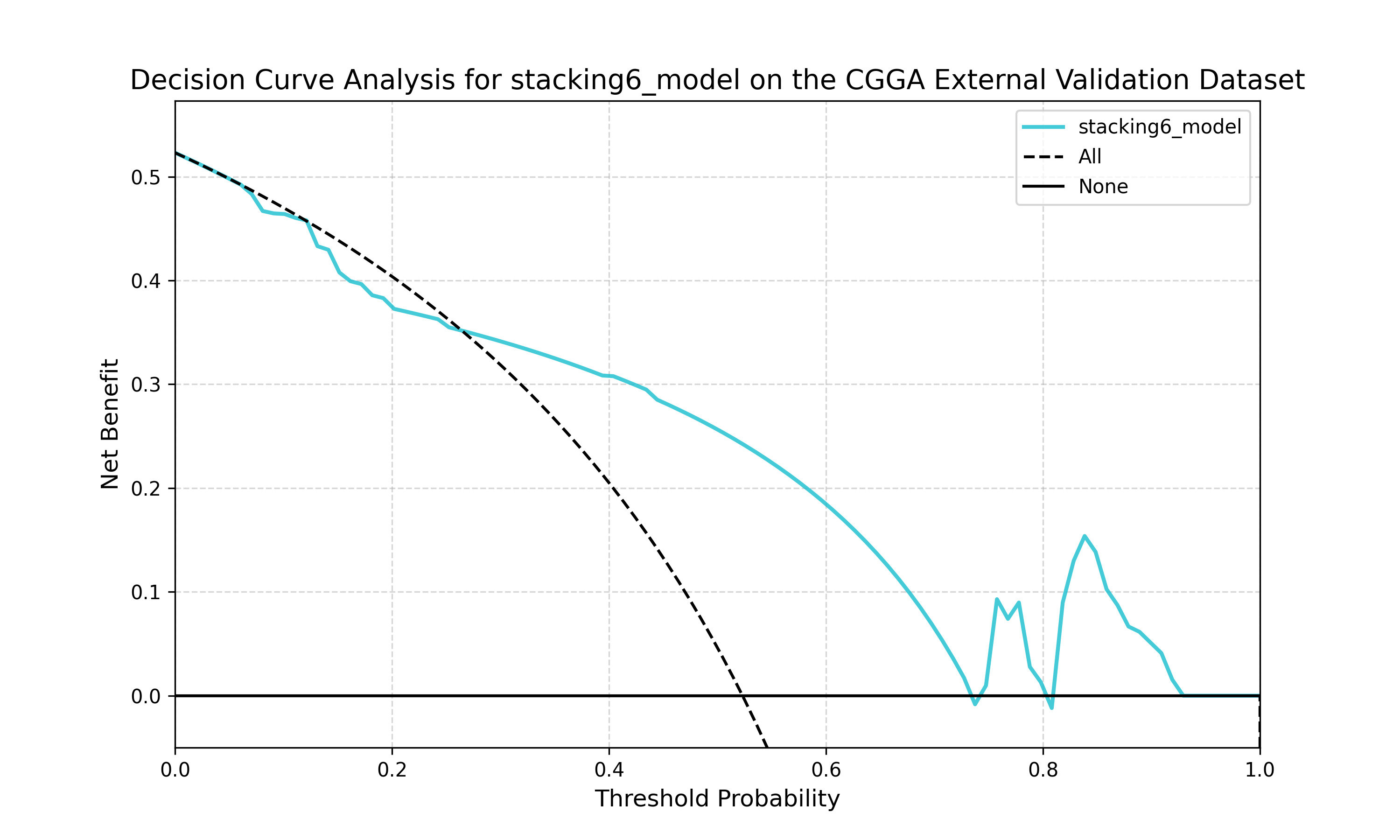

Supplement: S6 File — (ZIP) [file pone.0314831.s016.zip › S6 File/dca_curve_stacking6_model.png]

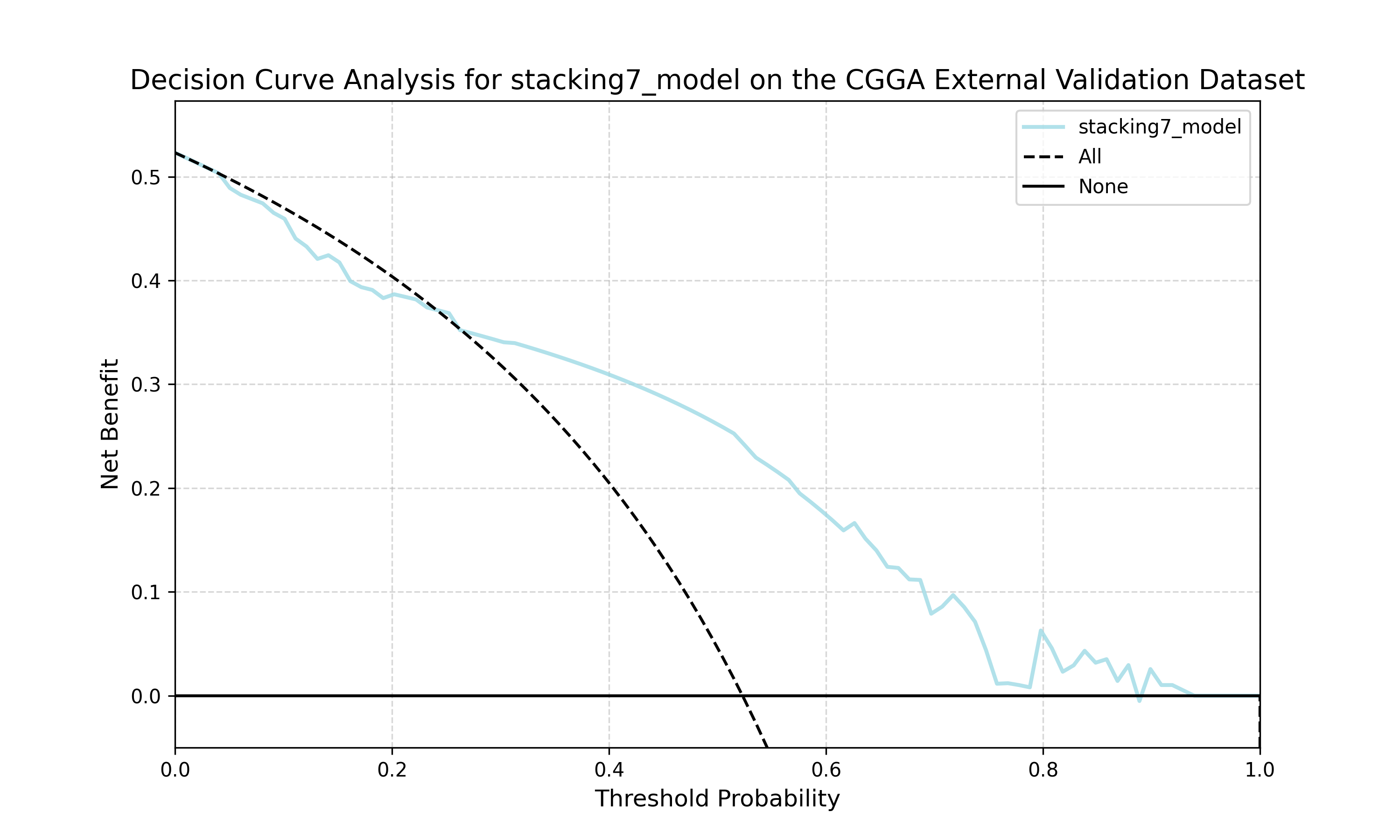

Supplement: S6 File — (ZIP) [file pone.0314831.s016.zip › S6 File/dca_curve_stacking7_model.png]

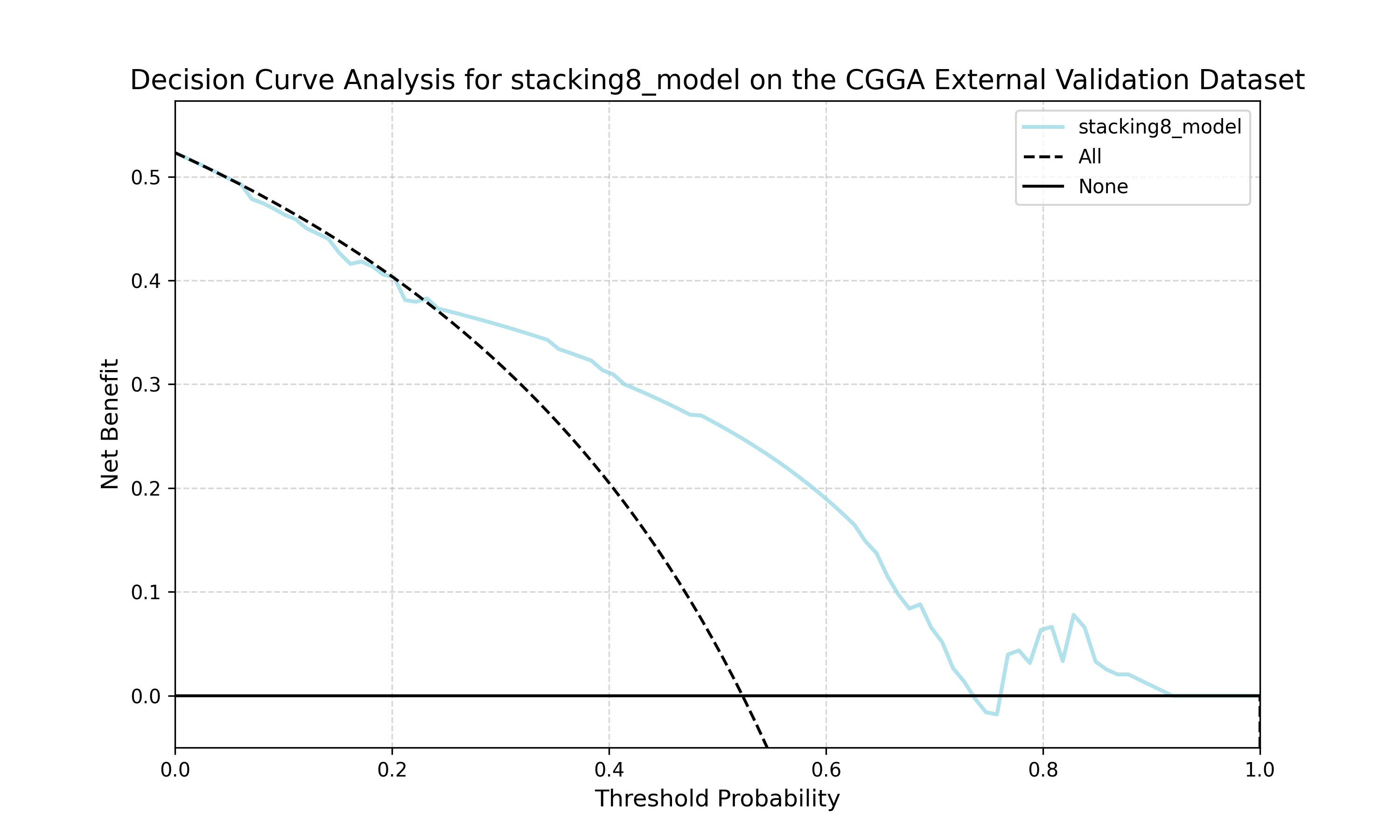

Supplement: S6 File — (ZIP) [file pone.0314831.s016.zip › S6 File/dca_curve_stacking8_model.png]

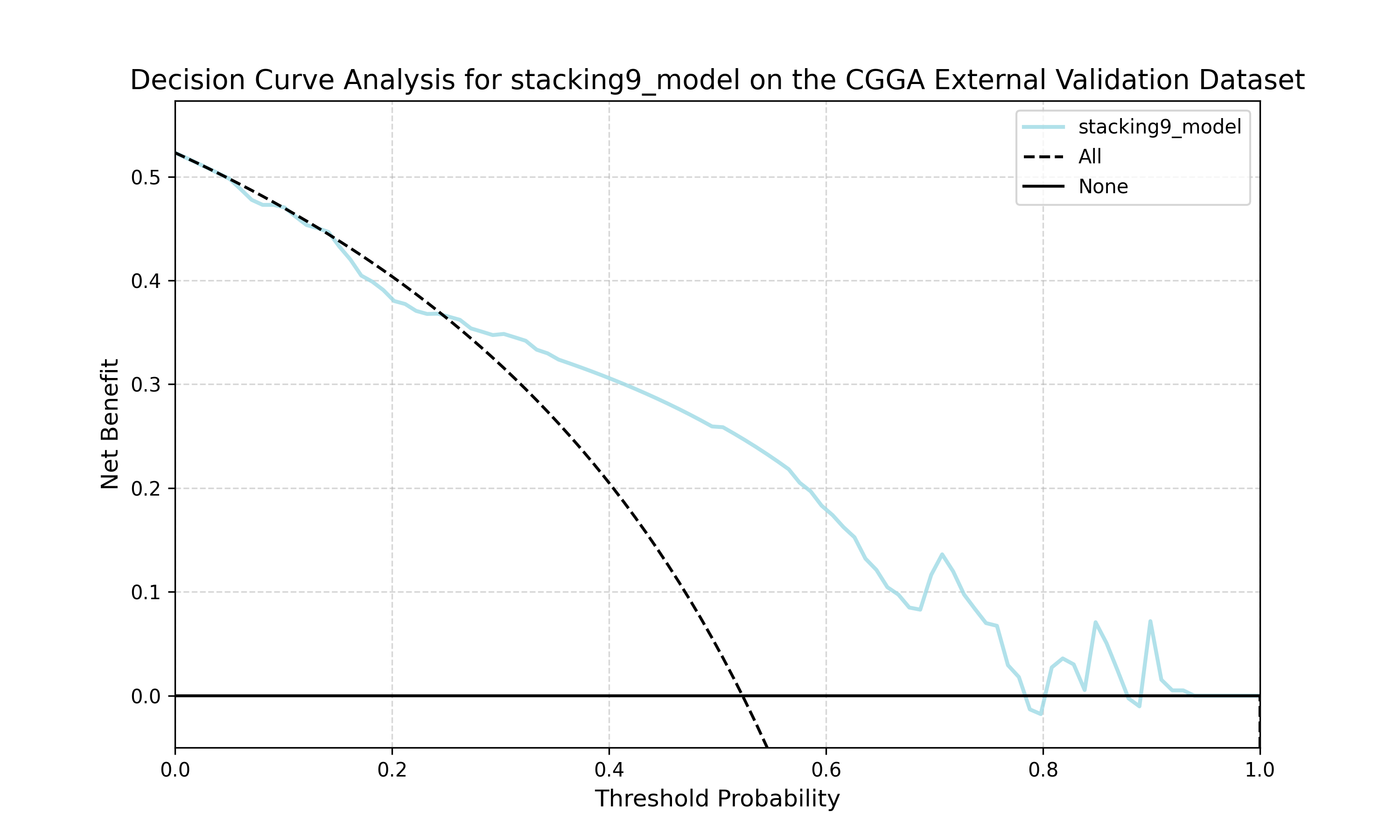

Supplement: S6 File — (ZIP) [file pone.0314831.s016.zip › S6 File/dca_curve_stacking9_model.png]

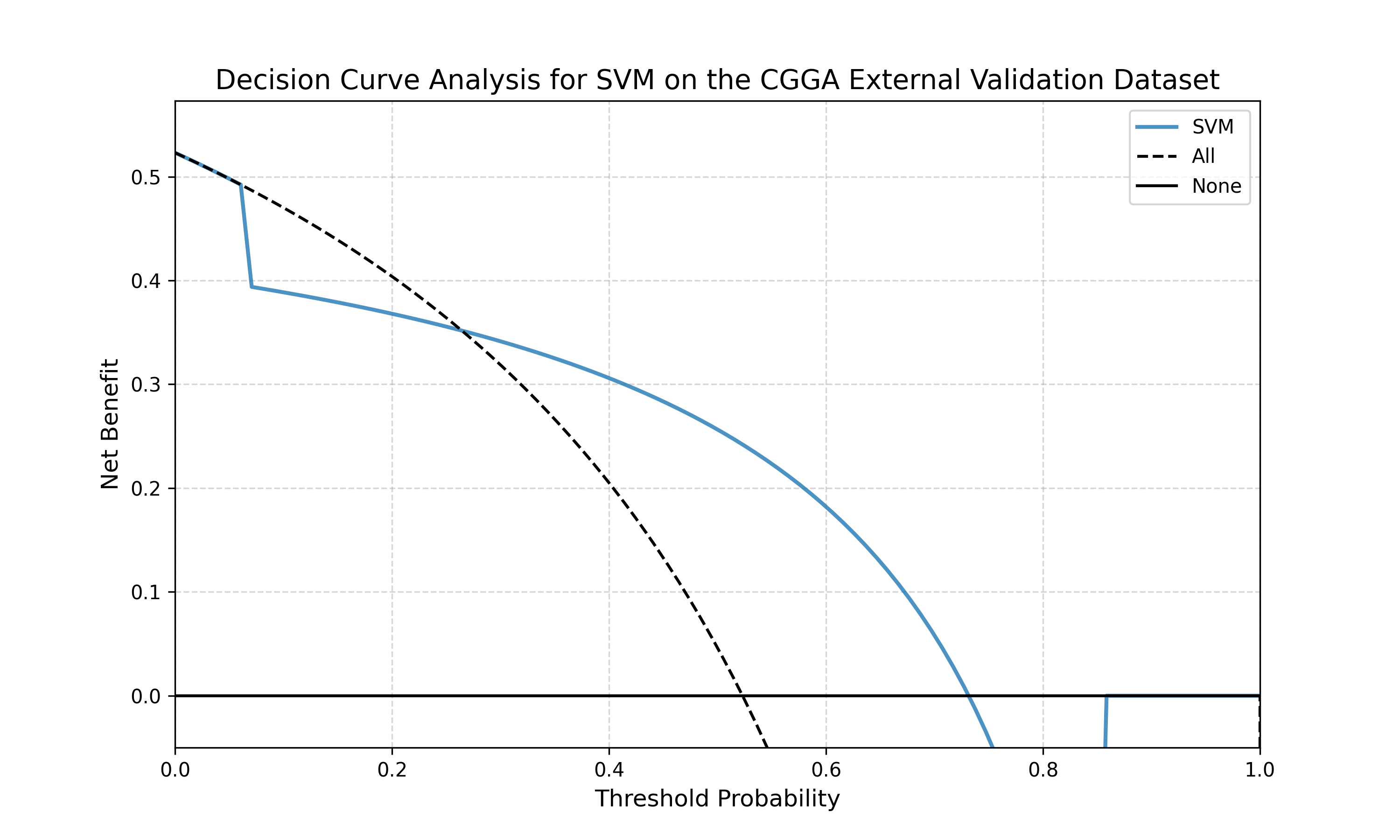

Supplement: S6 File — (ZIP) [file pone.0314831.s016.zip › S6 File/dca_curve_SVM.png]

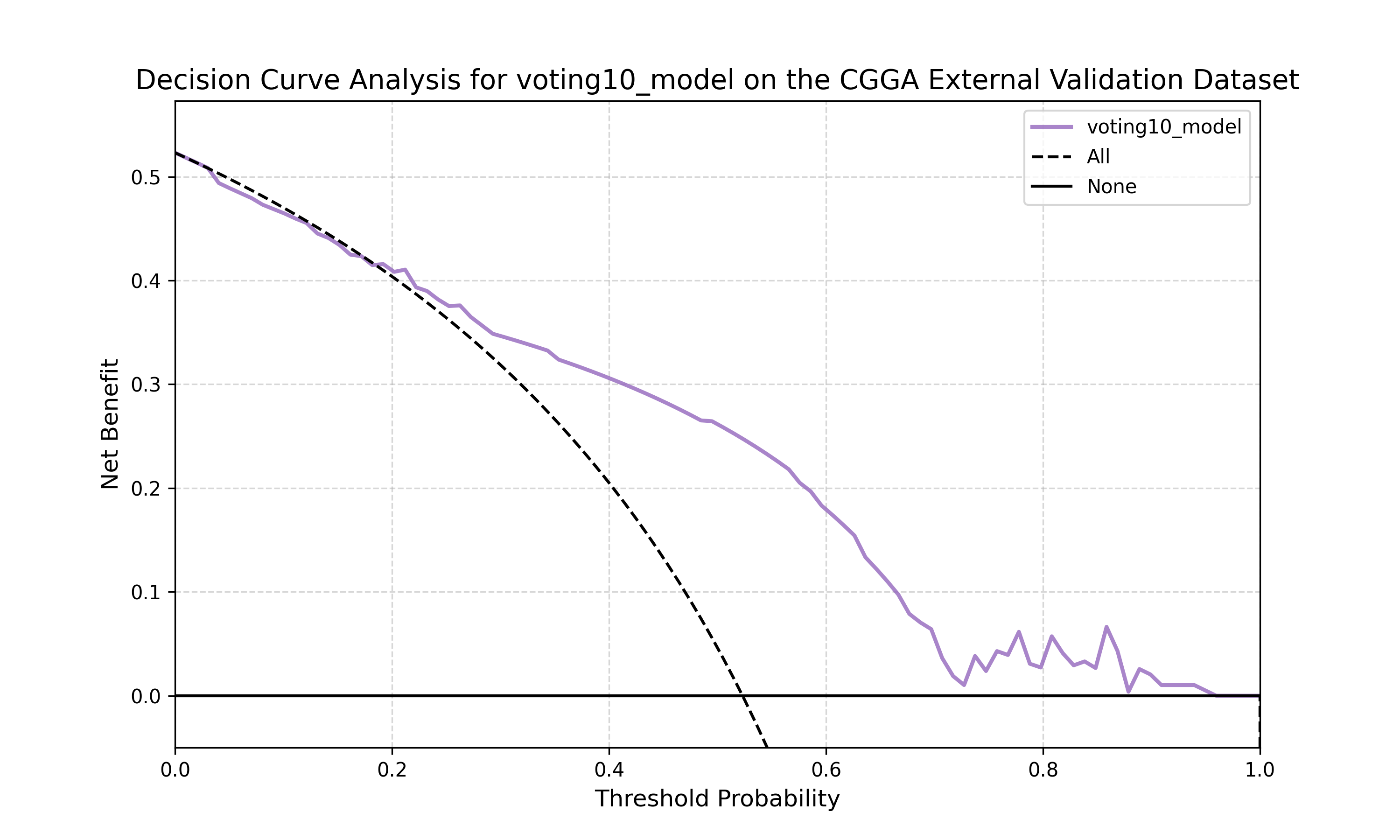

Supplement: S6 File — (ZIP) [file pone.0314831.s016.zip › S6 File/dca_curve_voting10_model.png]

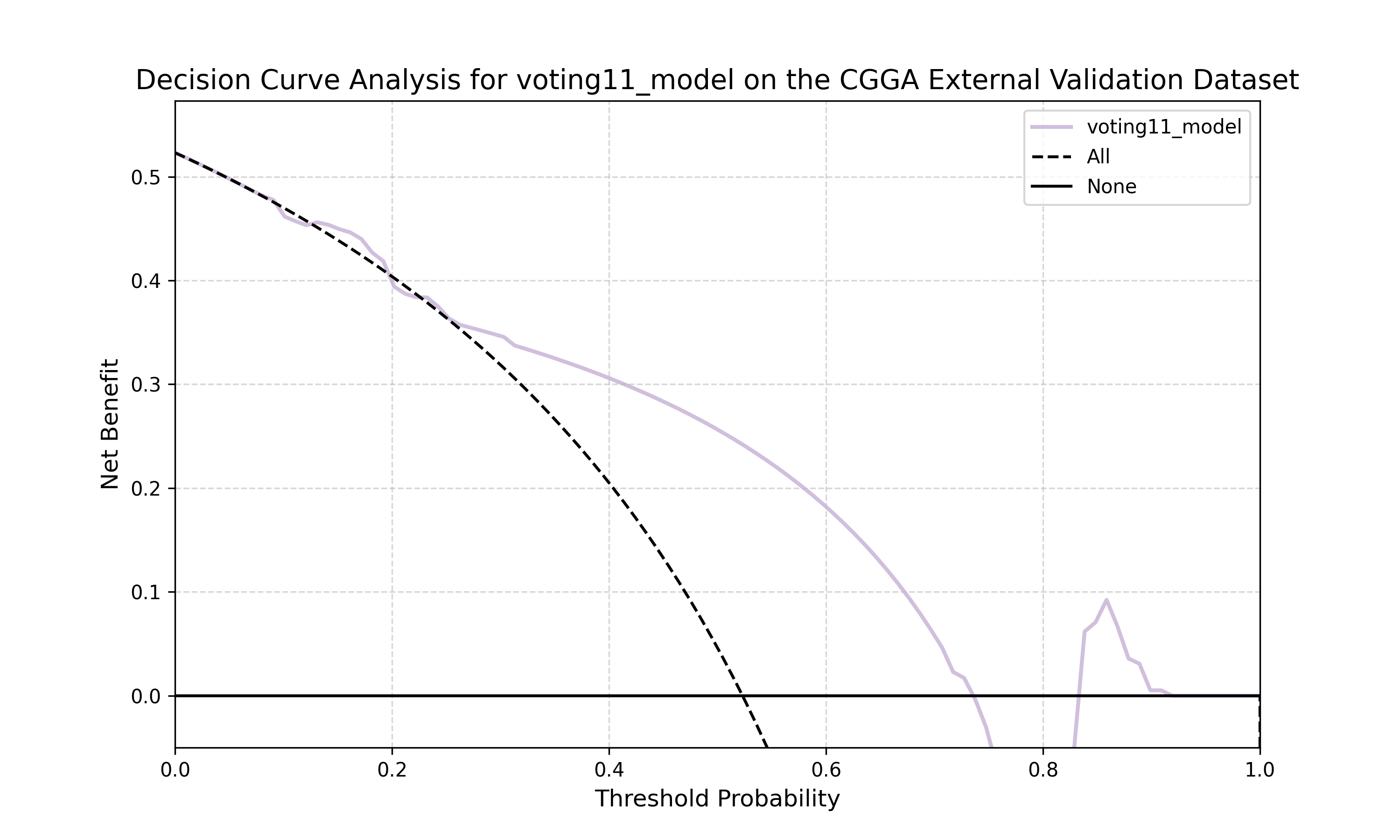

Supplement: S6 File — (ZIP) [file pone.0314831.s016.zip › S6 File/dca_curve_voting11_model.png]

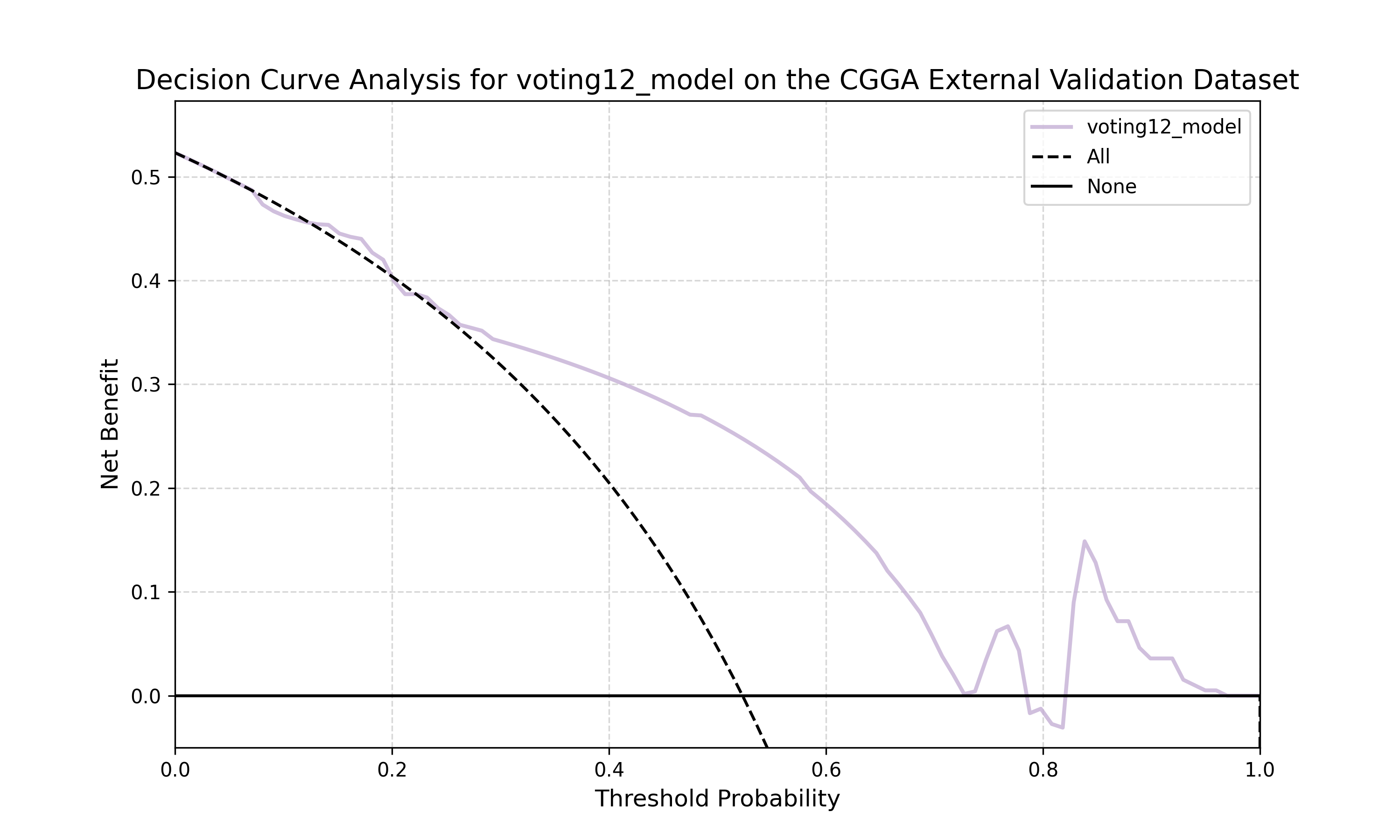

Supplement: S6 File — (ZIP) [file pone.0314831.s016.zip › S6 File/dca_curve_voting12_model.png]

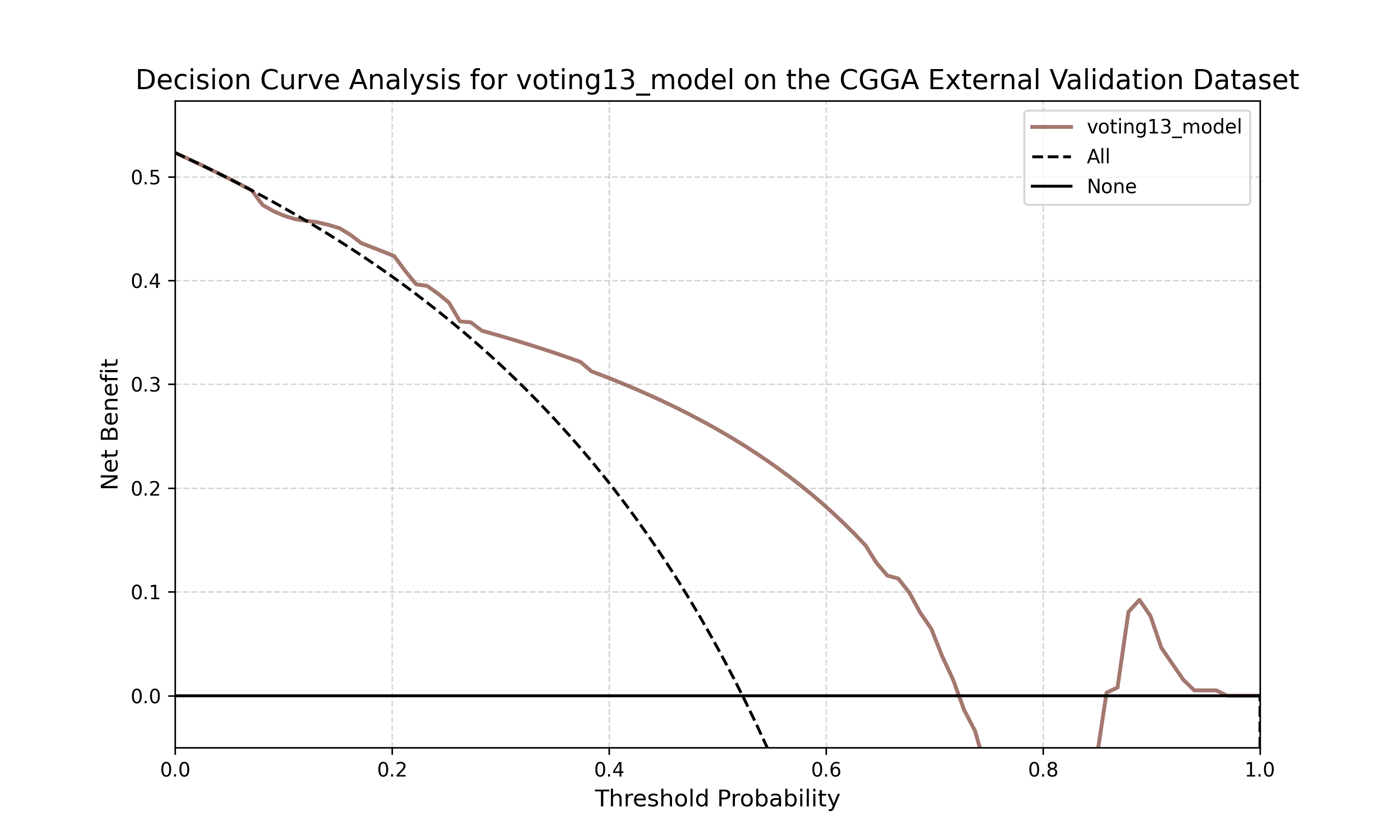

Supplement: S6 File — (ZIP) [file pone.0314831.s016.zip › S6 File/dca_curve_voting13_model.png]

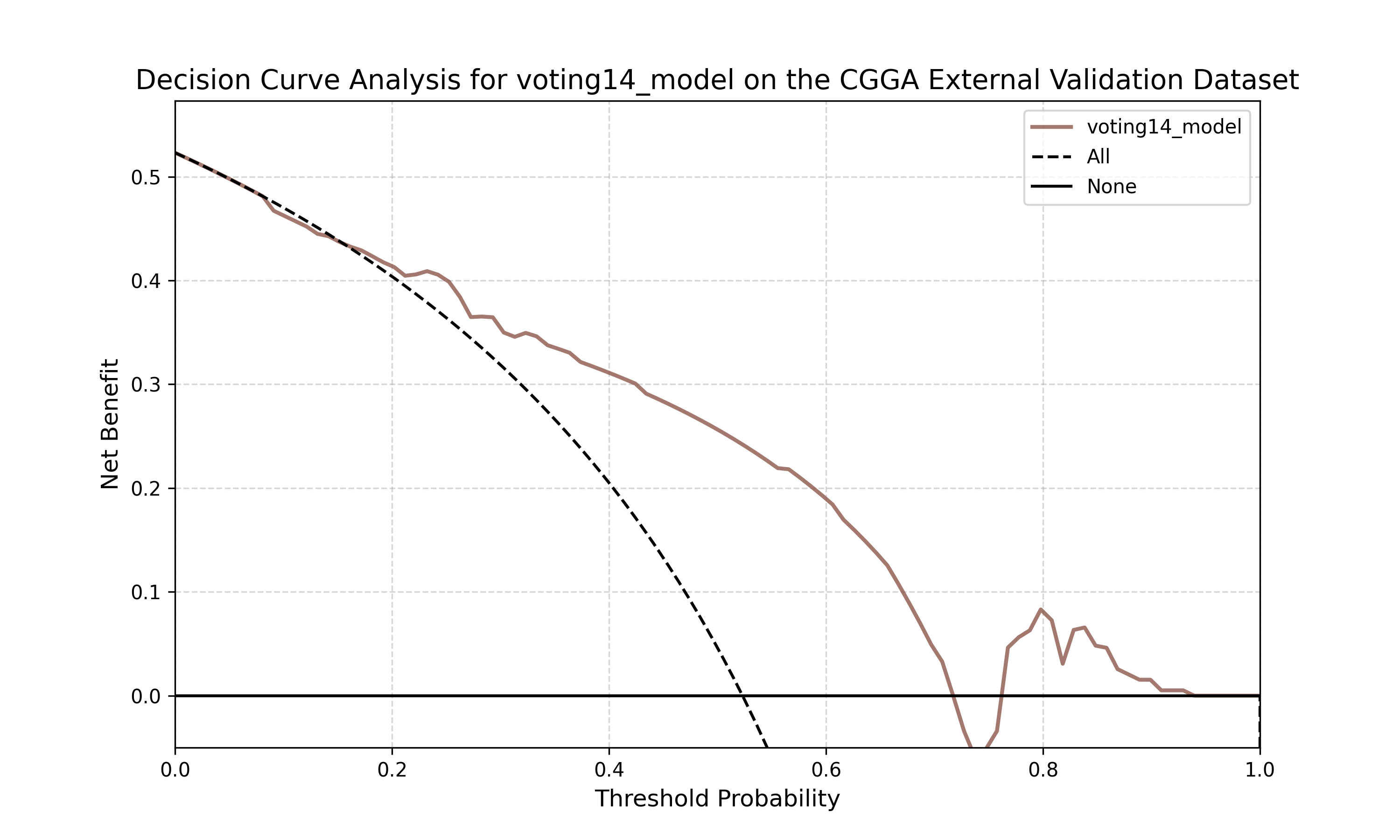

Supplement: S6 File — (ZIP) [file pone.0314831.s016.zip › S6 File/dca_curve_voting14_model.png]

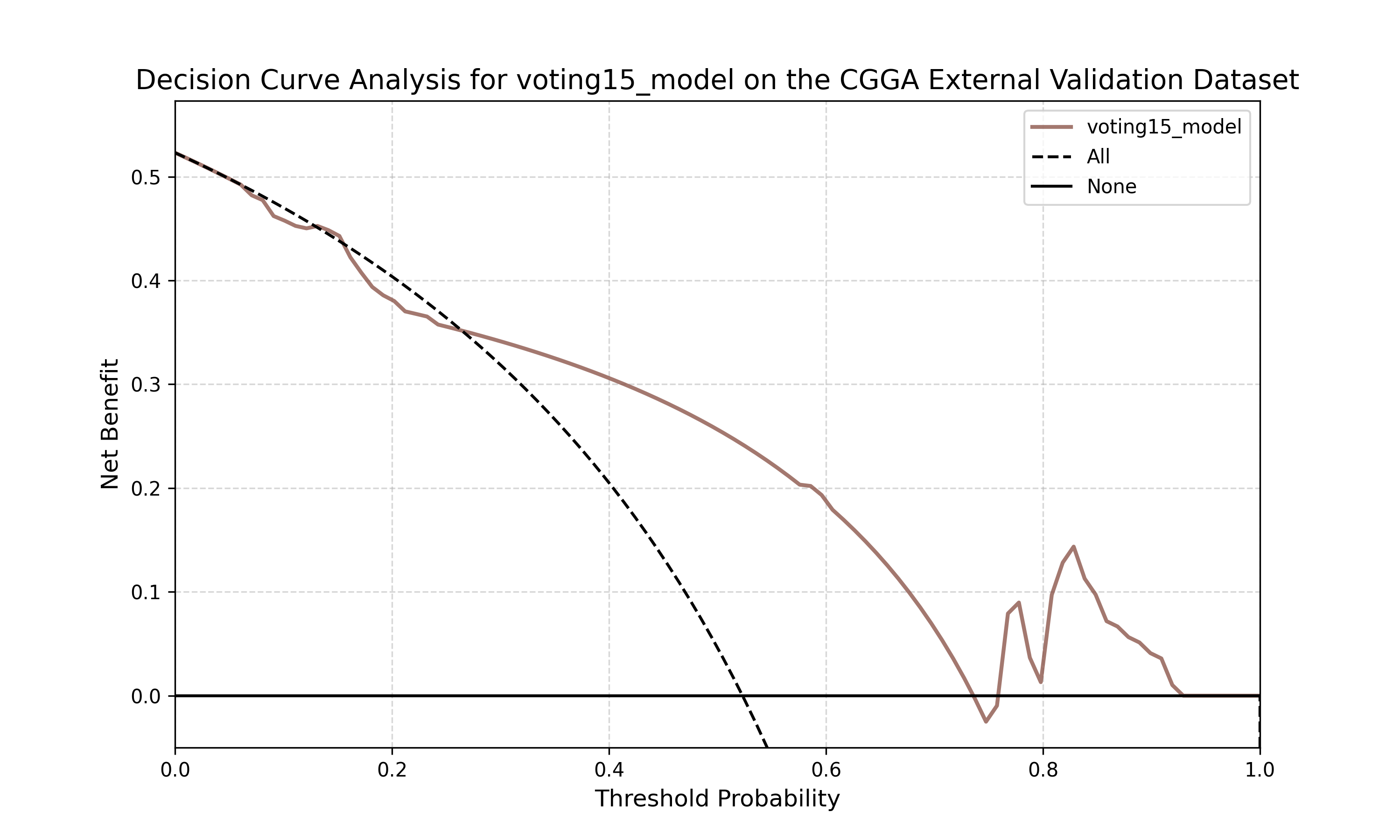

Supplement: S6 File — (ZIP) [file pone.0314831.s016.zip › S6 File/dca_curve_voting15_model.png]

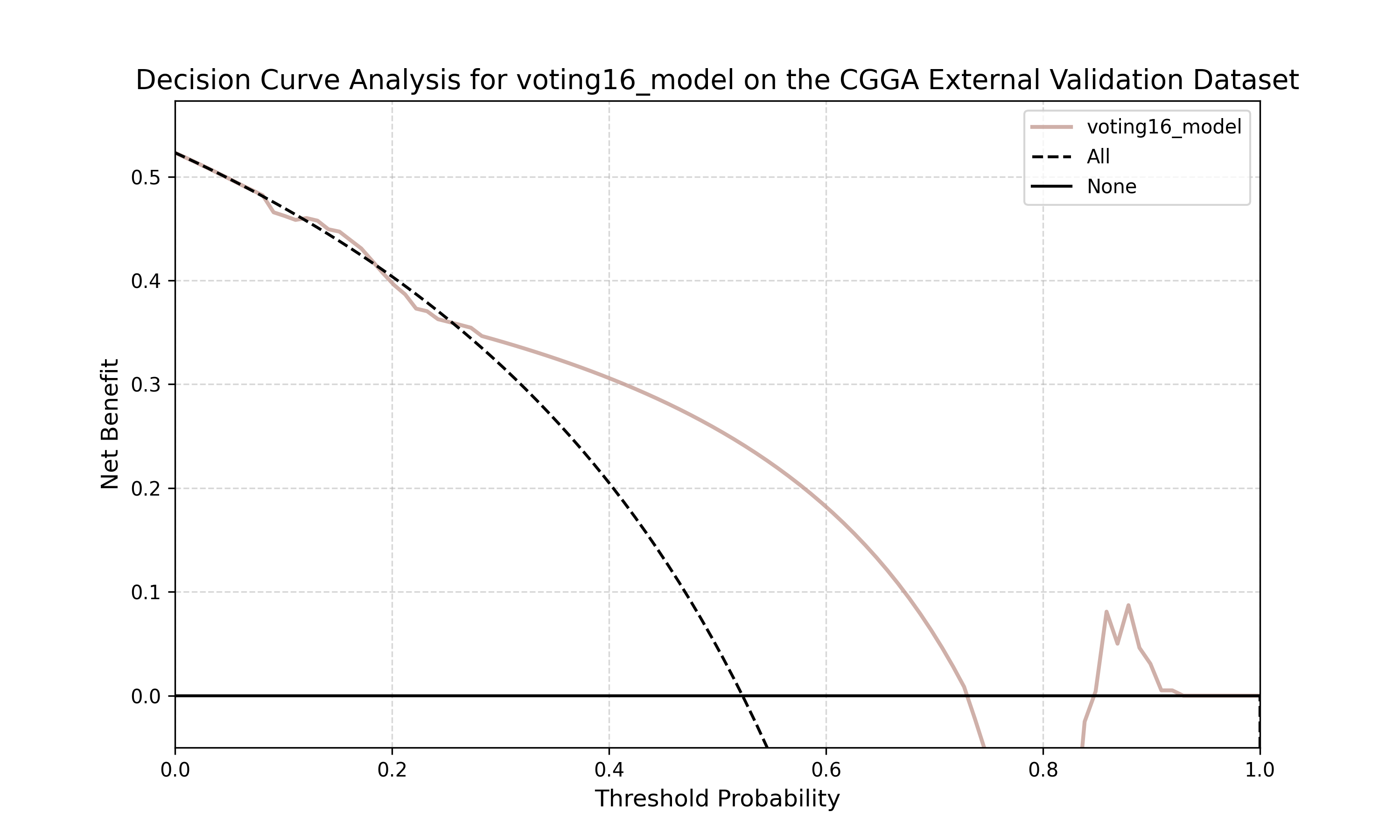

Supplement: S6 File — (ZIP) [file pone.0314831.s016.zip › S6 File/dca_curve_voting16_model.png]

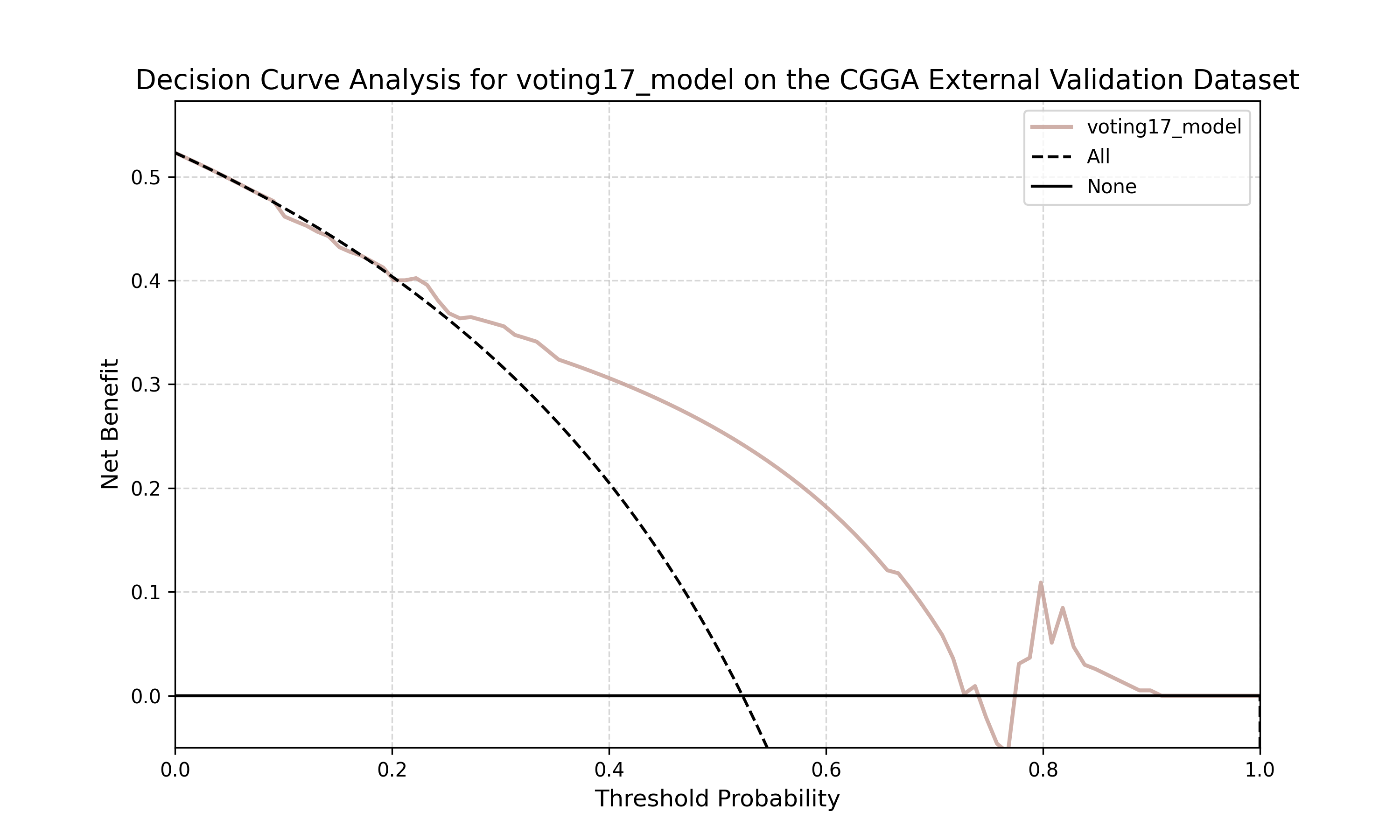

Supplement: S6 File — (ZIP) [file pone.0314831.s016.zip › S6 File/dca_curve_voting17_model.png]

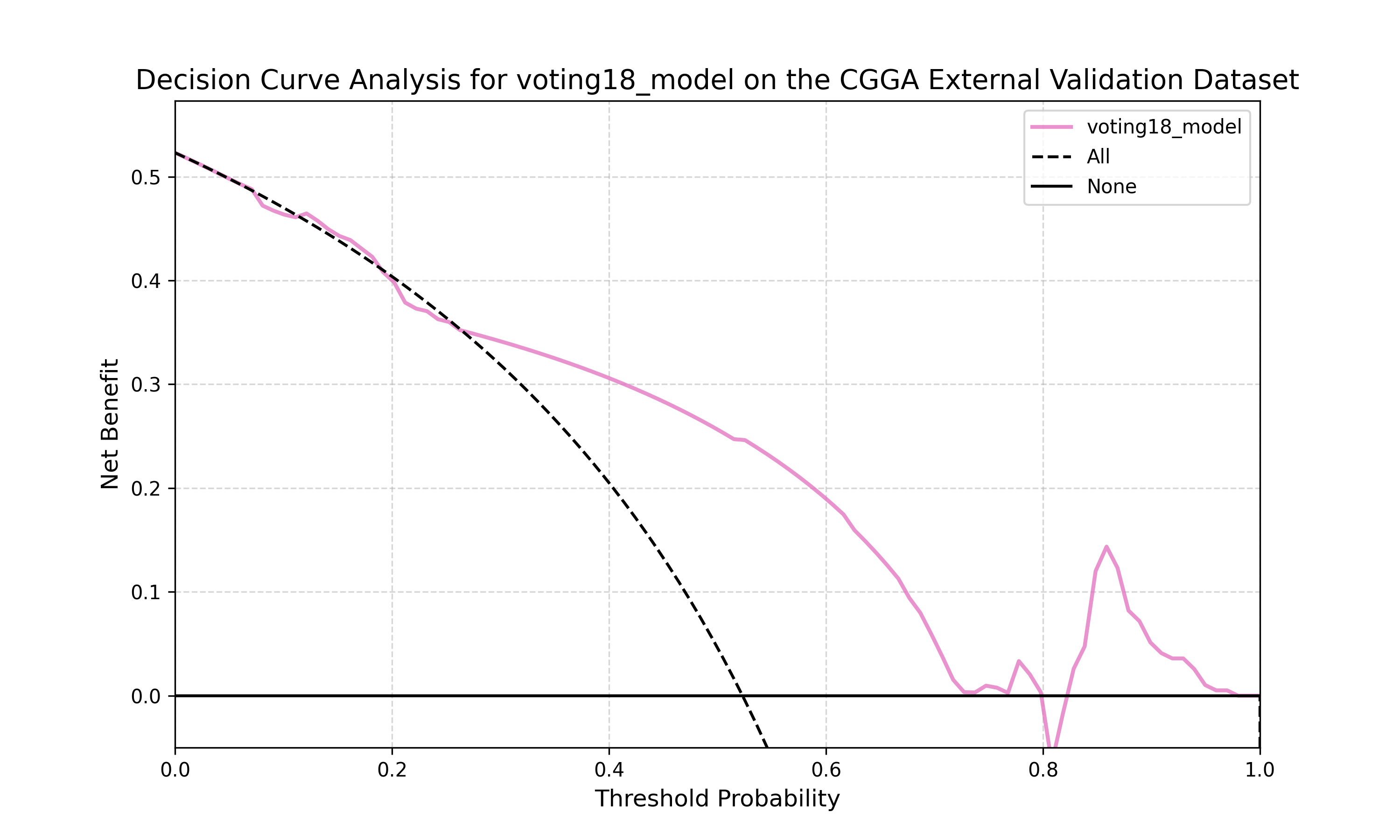

Supplement: S6 File — (ZIP) [file pone.0314831.s016.zip › S6 File/dca_curve_voting18_model.png]

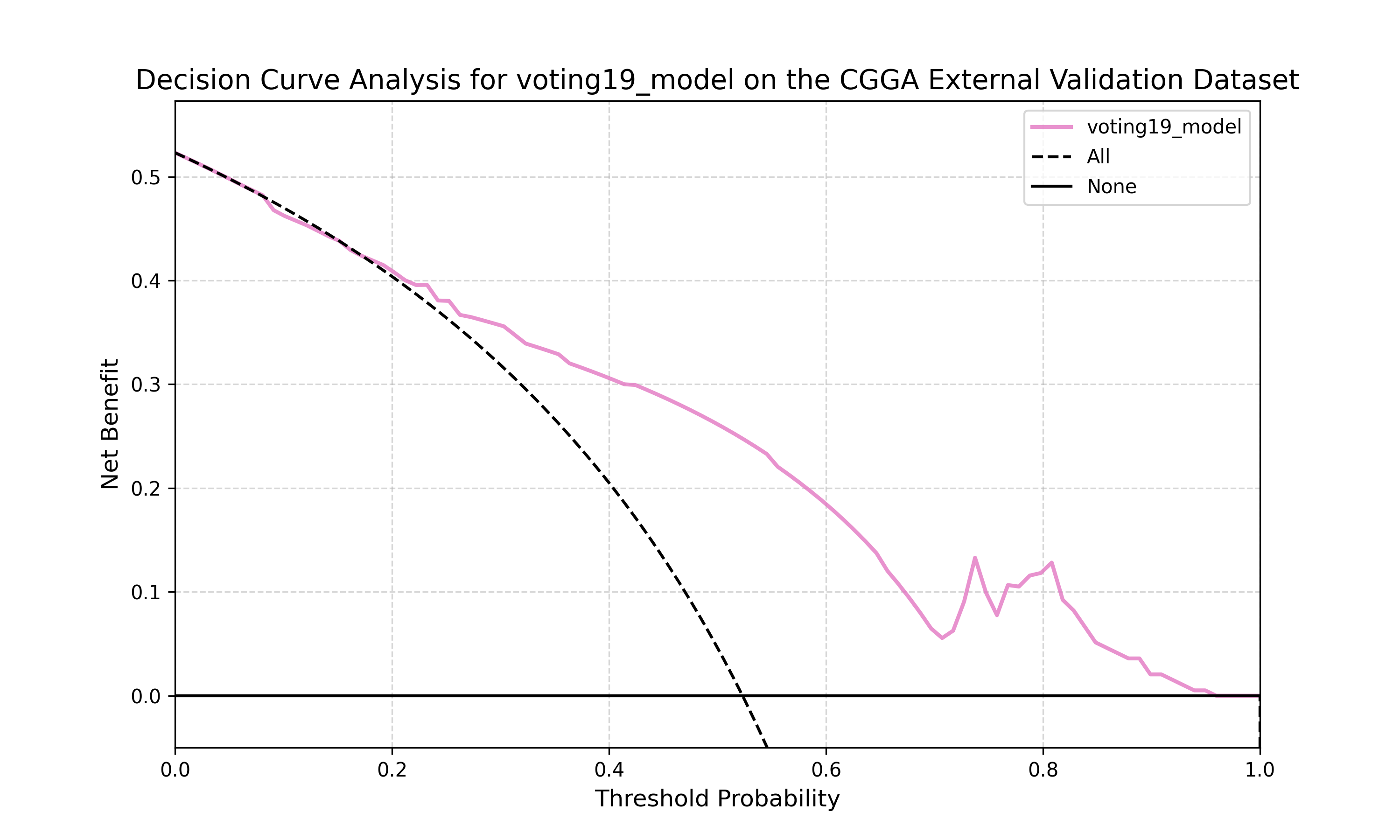

Supplement: S6 File — (ZIP) [file pone.0314831.s016.zip › S6 File/dca_curve_voting19_model.png]

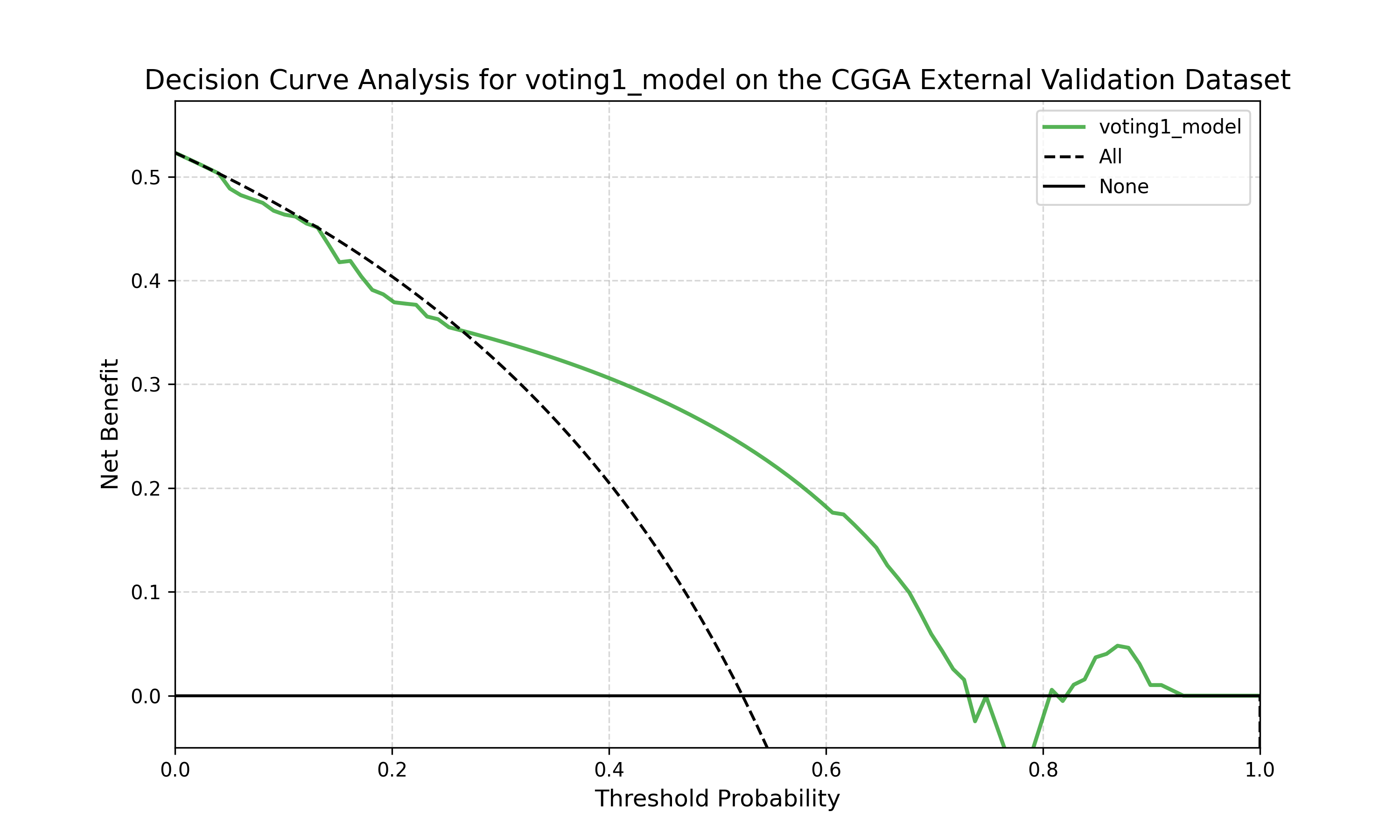

Supplement: S6 File — (ZIP) [file pone.0314831.s016.zip › S6 File/dca_curve_voting1_model.png]

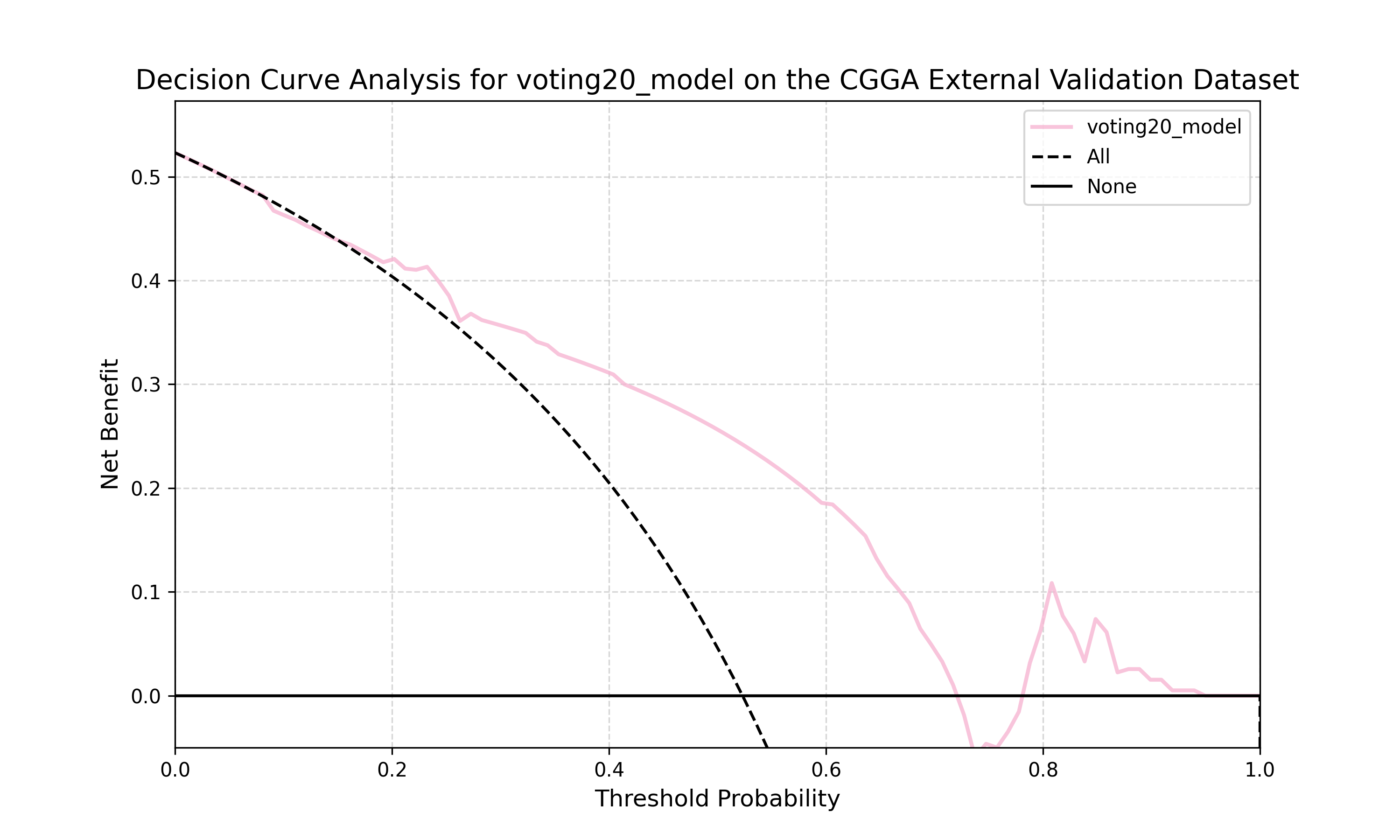

Supplement: S6 File — (ZIP) [file pone.0314831.s016.zip › S6 File/dca_curve_voting20_model.png]

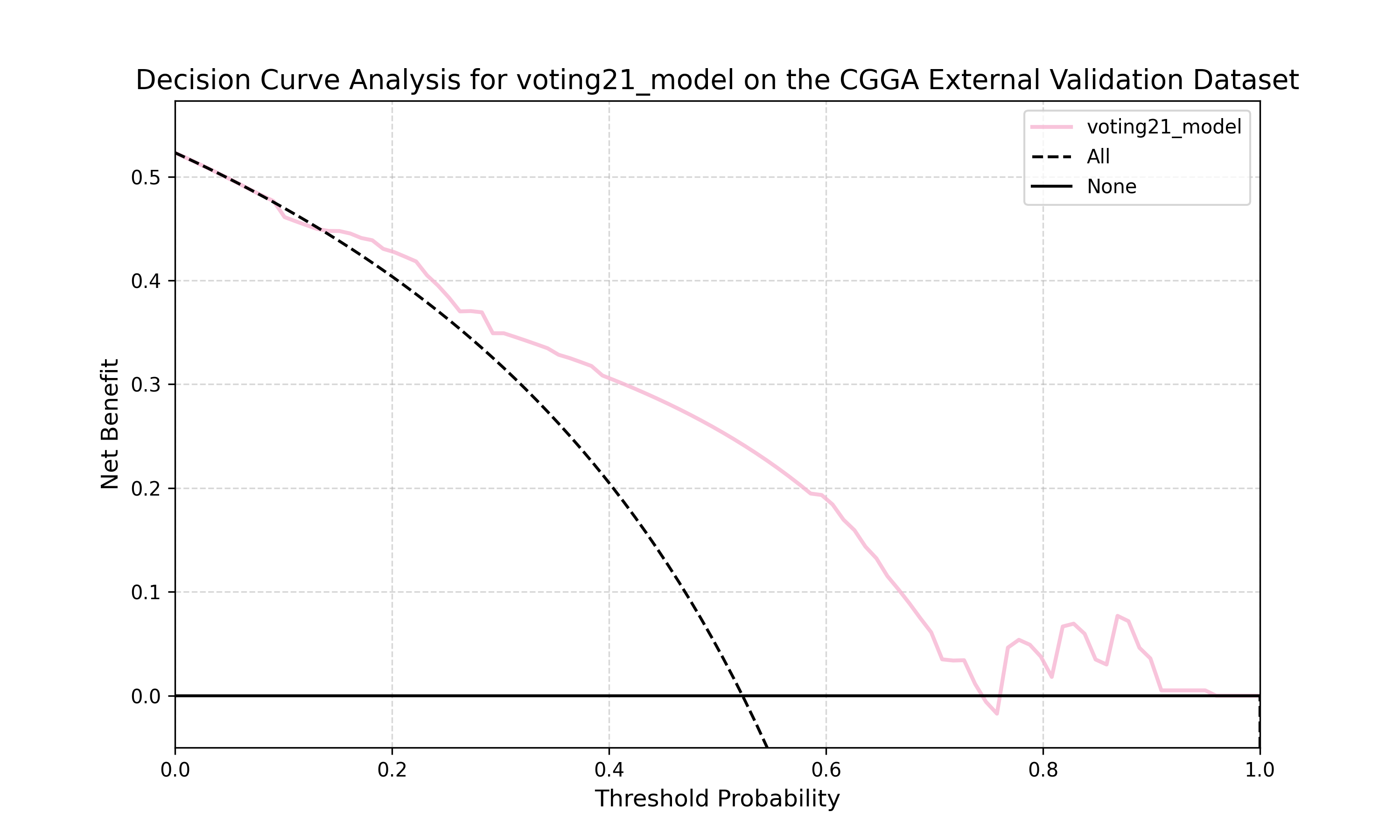

Supplement: S6 File — (ZIP) [file pone.0314831.s016.zip › S6 File/dca_curve_voting21_model.png]

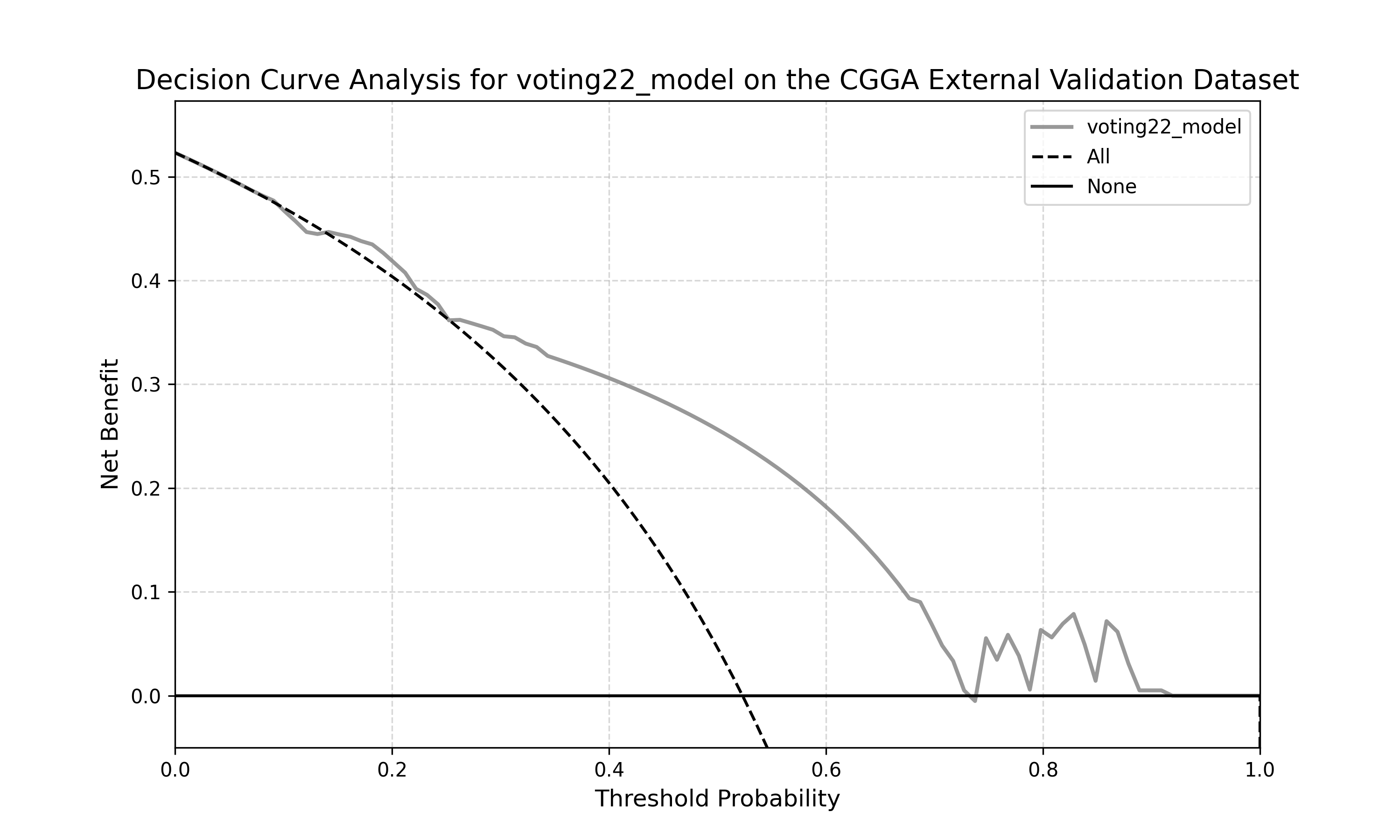

Supplement: S6 File — (ZIP) [file pone.0314831.s016.zip › S6 File/dca_curve_voting22_model.png]

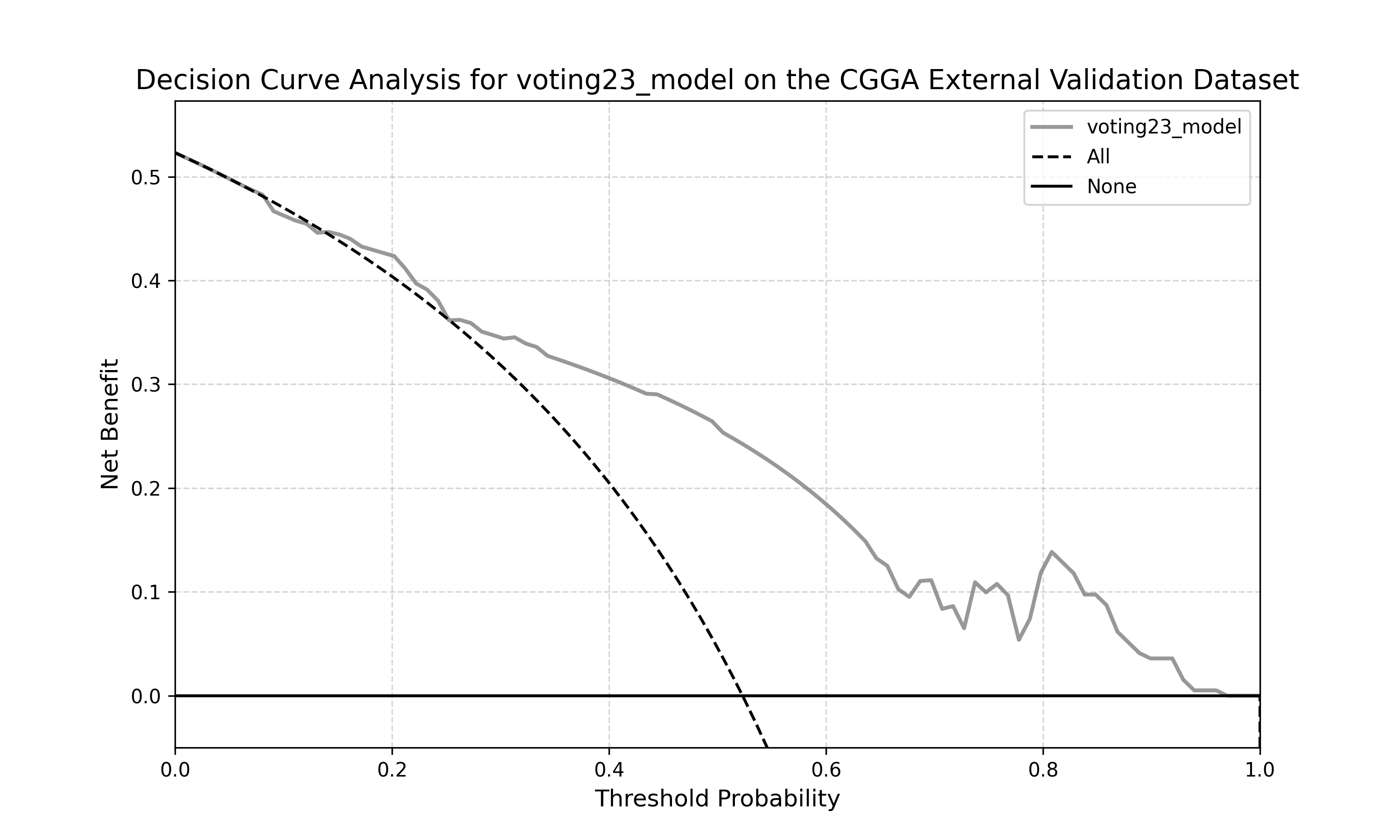

Supplement: S6 File — (ZIP) [file pone.0314831.s016.zip › S6 File/dca_curve_voting23_model.png]

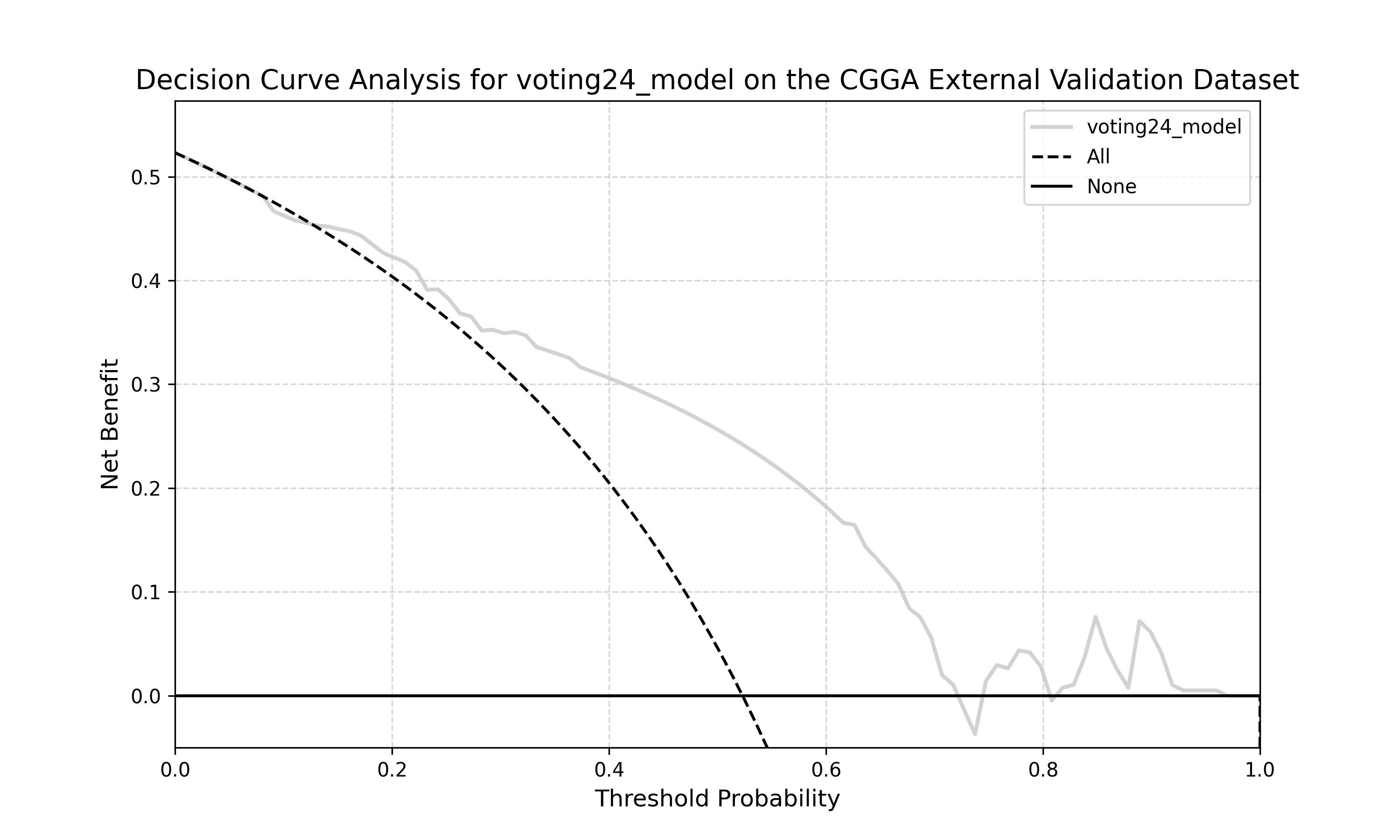

Supplement: S6 File — (ZIP) [file pone.0314831.s016.zip › S6 File/dca_curve_voting24_model.png]

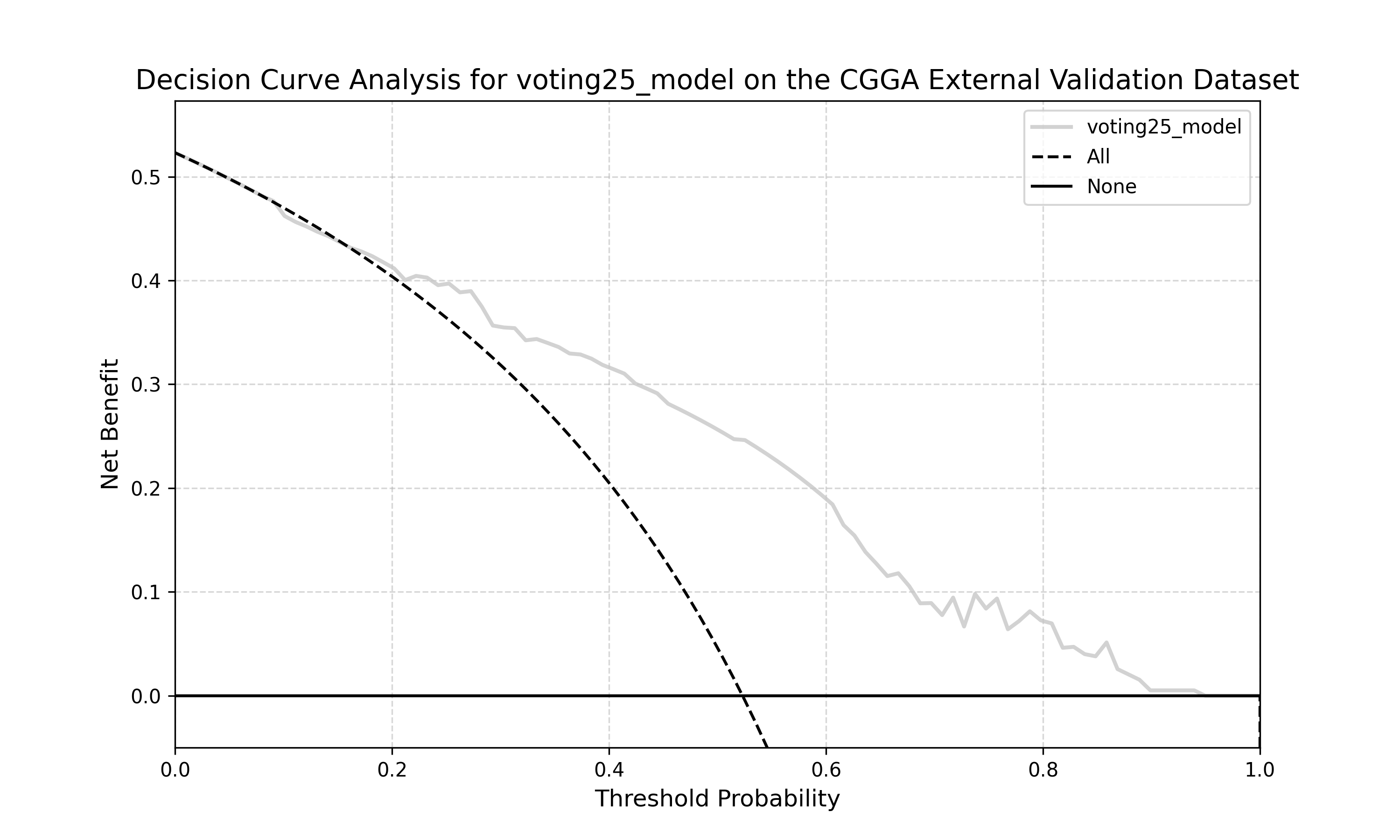

Supplement: S6 File — (ZIP) [file pone.0314831.s016.zip › S6 File/dca_curve_voting25_model.png]

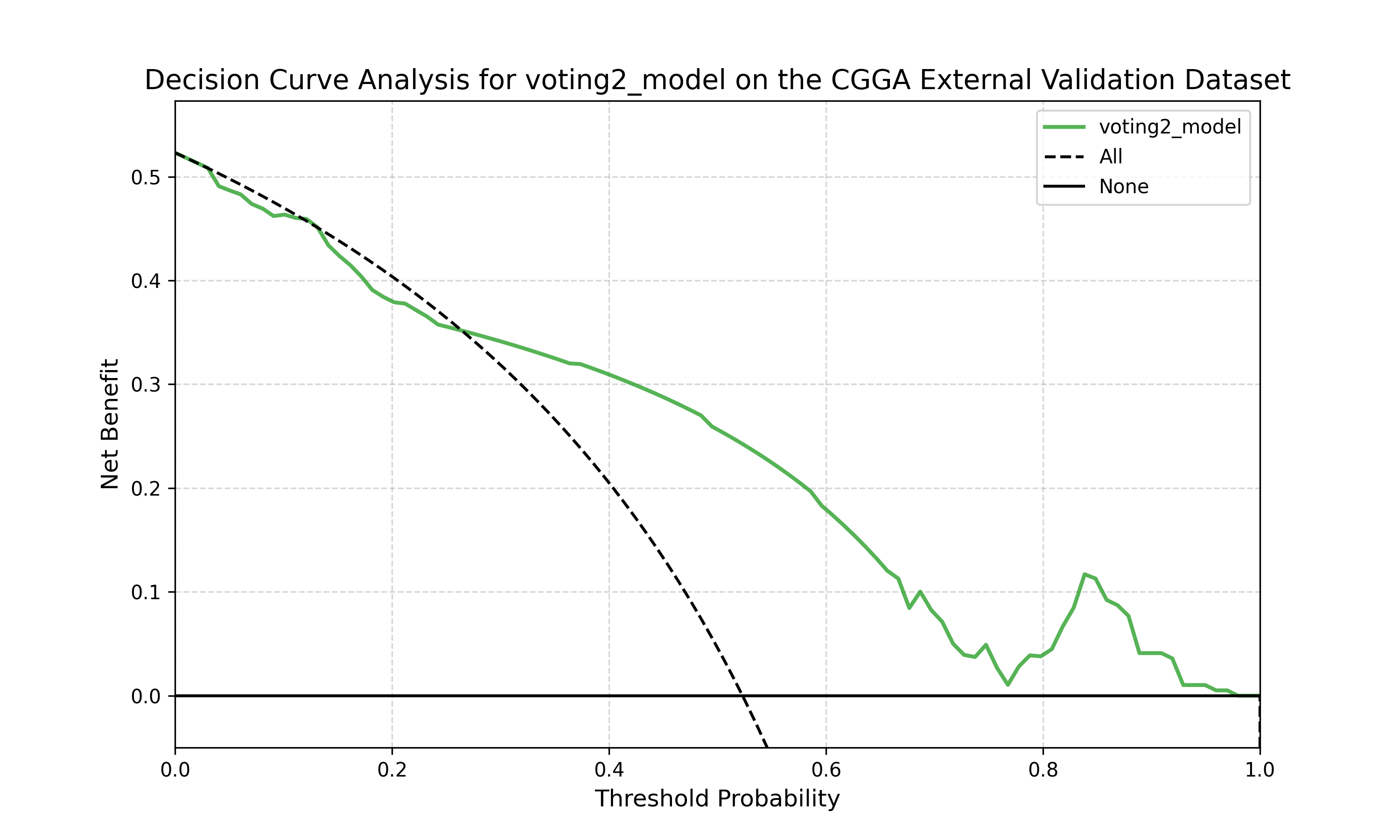

Supplement: S6 File — (ZIP) [file pone.0314831.s016.zip › S6 File/dca_curve_voting2_model.png]

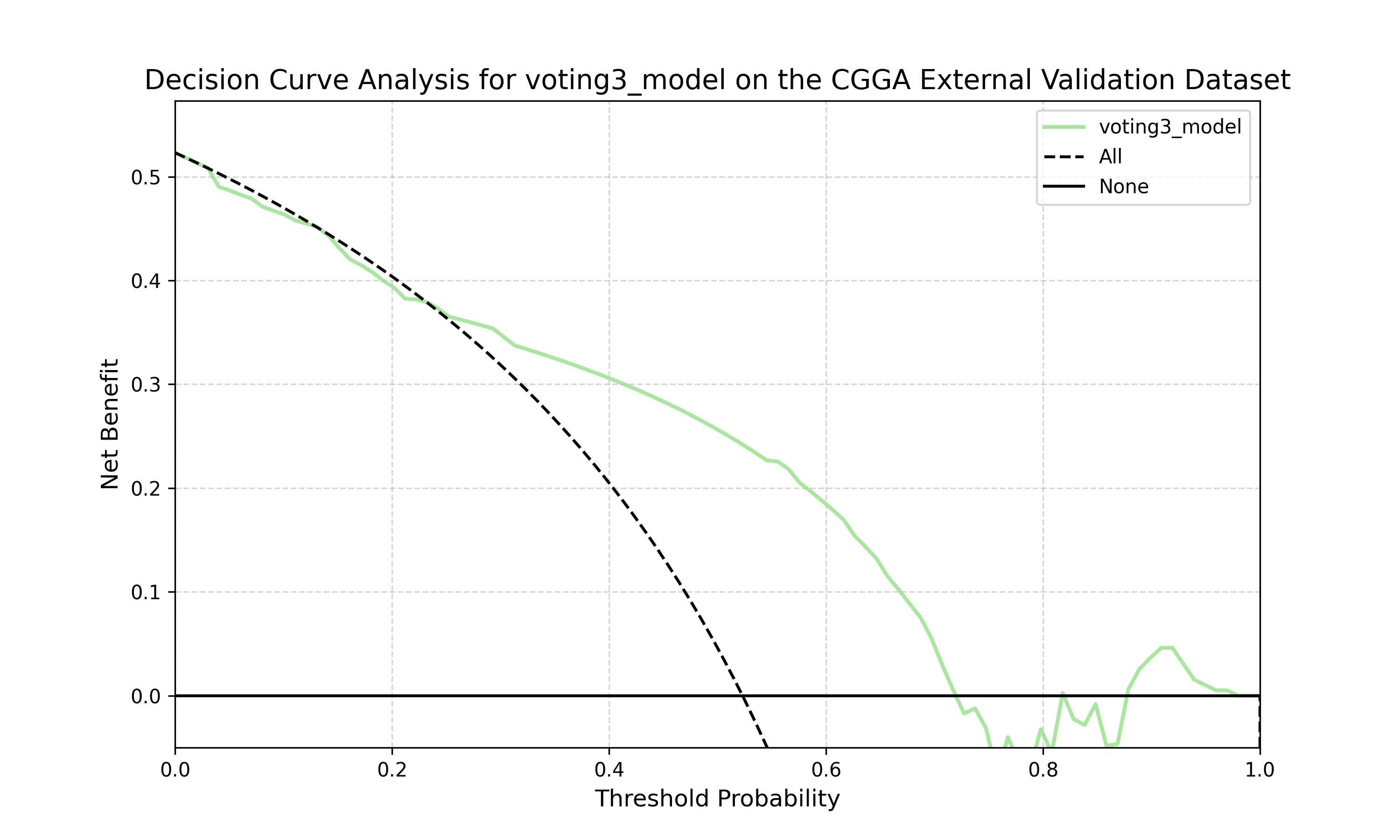

Supplement: S6 File — (ZIP) [file pone.0314831.s016.zip › S6 File/dca_curve_voting3_model.png]

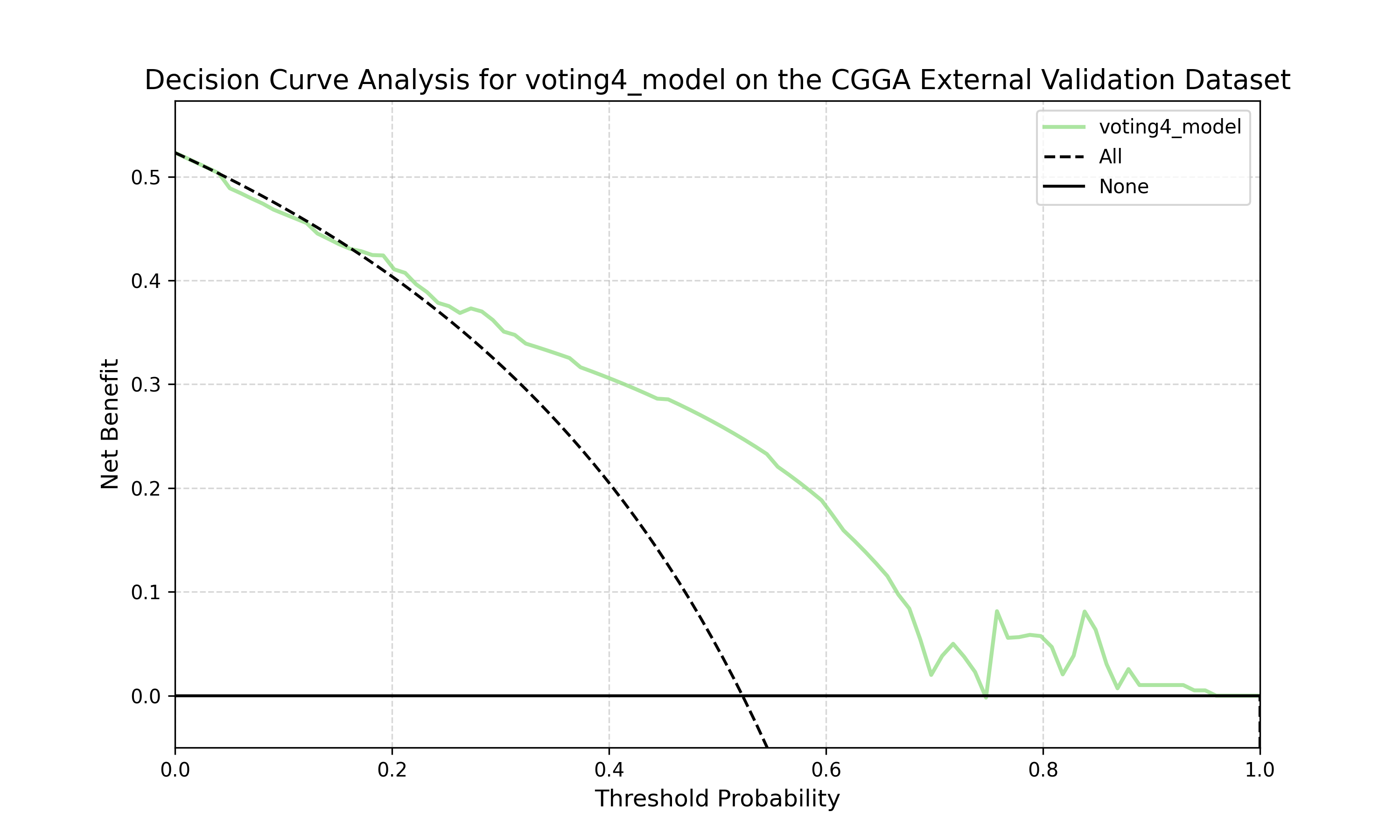

Supplement: S6 File — (ZIP) [file pone.0314831.s016.zip › S6 File/dca_curve_voting4_model.png]

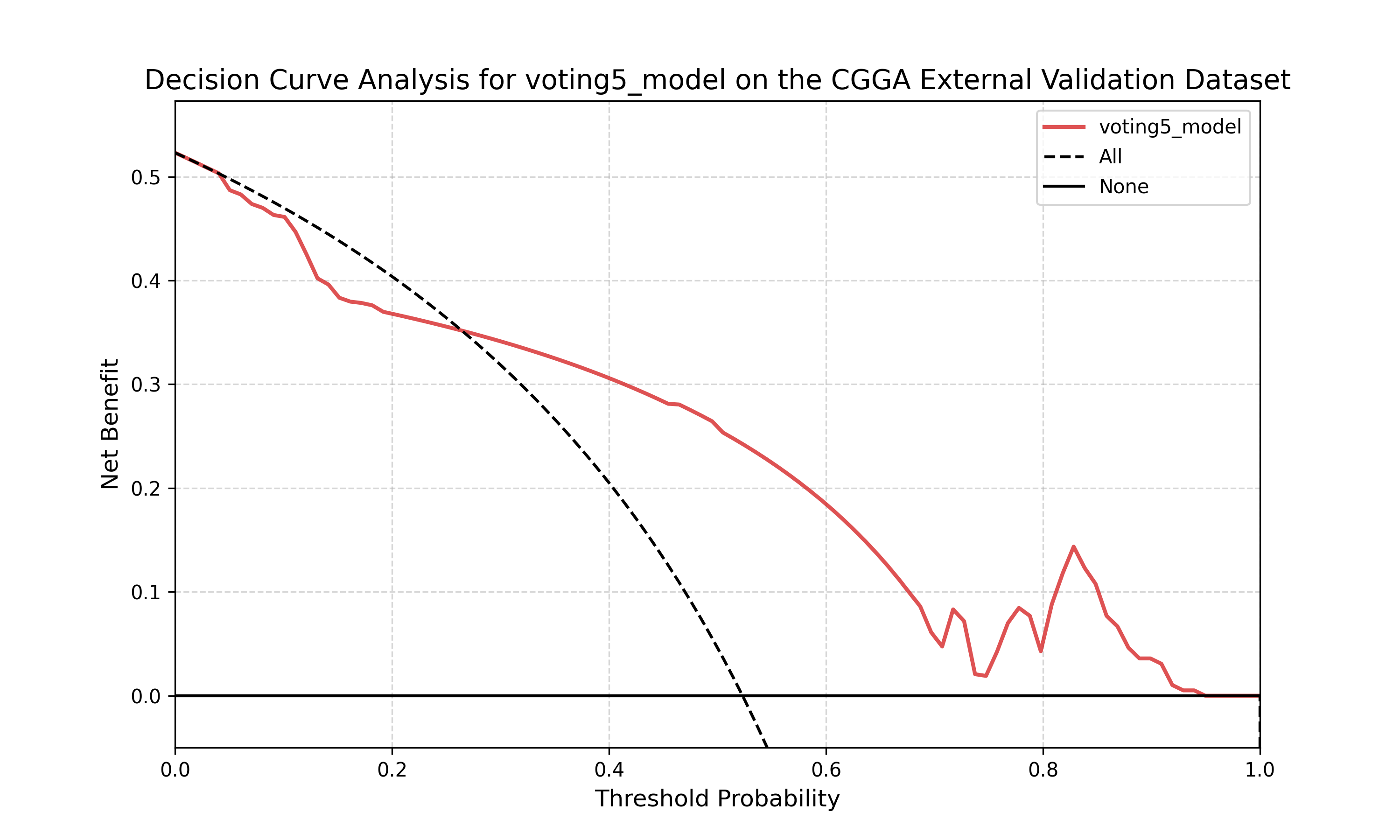

Supplement: S6 File — (ZIP) [file pone.0314831.s016.zip › S6 File/dca_curve_voting5_model.png]

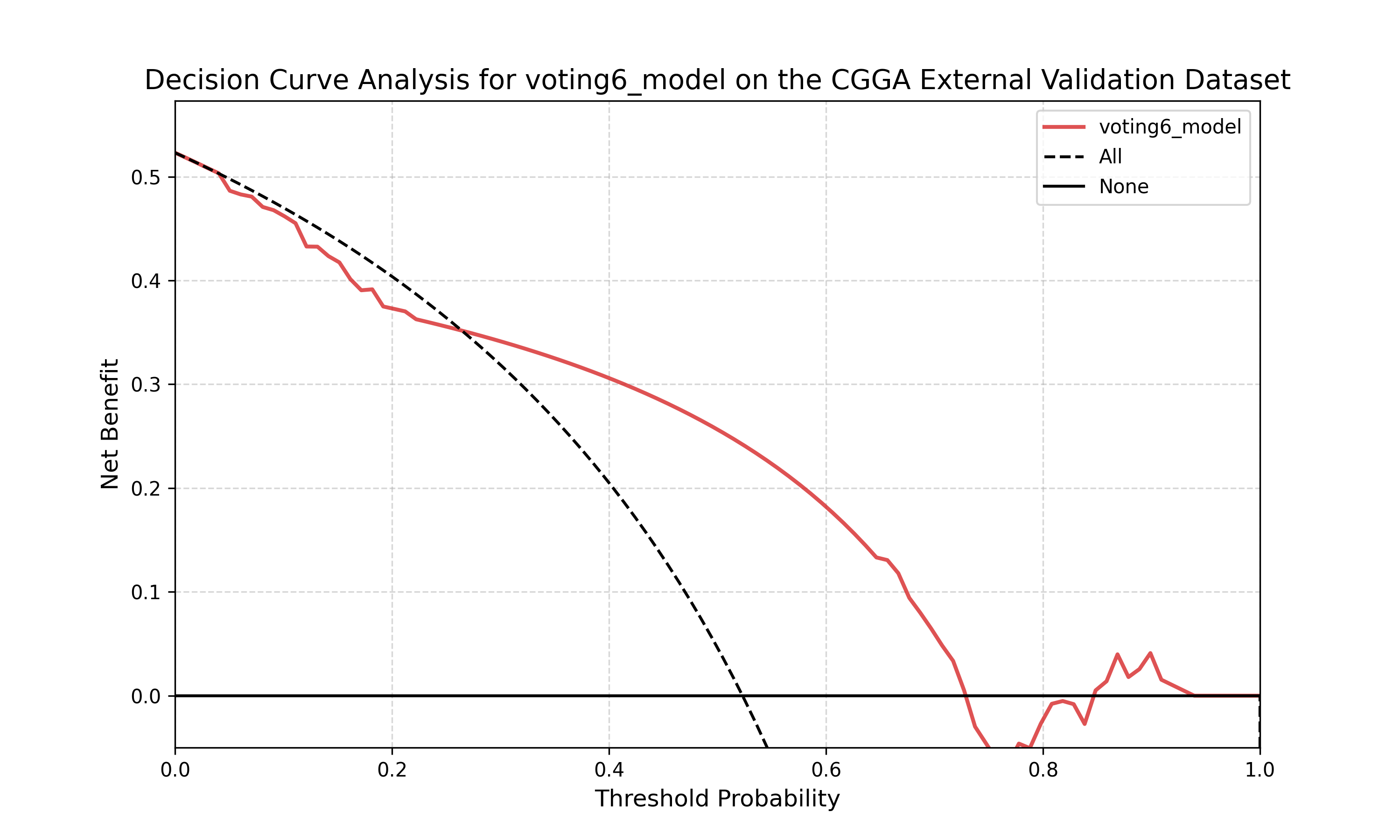

Supplement: S6 File — (ZIP) [file pone.0314831.s016.zip › S6 File/dca_curve_voting6_model.png]

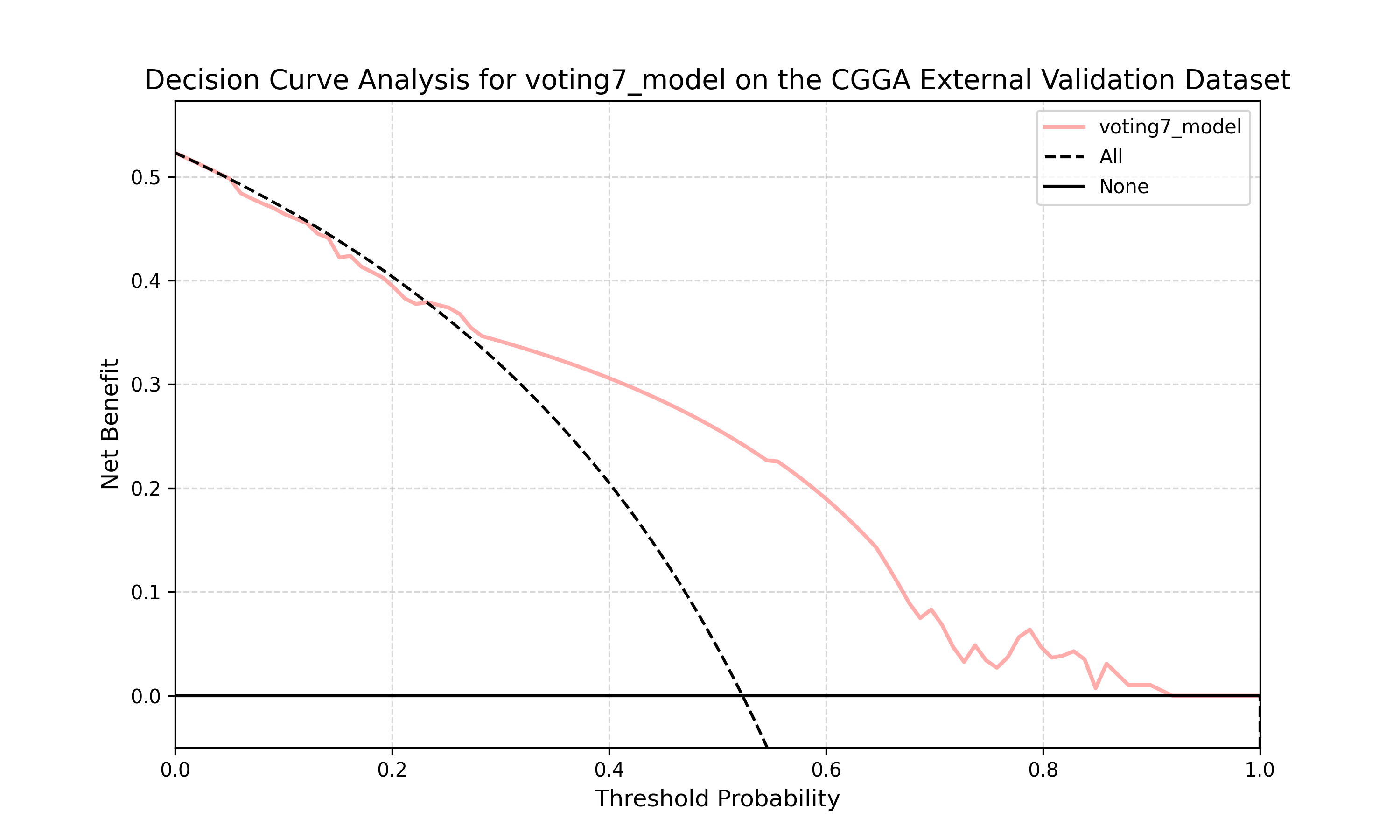

Supplement: S6 File — (ZIP) [file pone.0314831.s016.zip › S6 File/dca_curve_voting7_model.png]

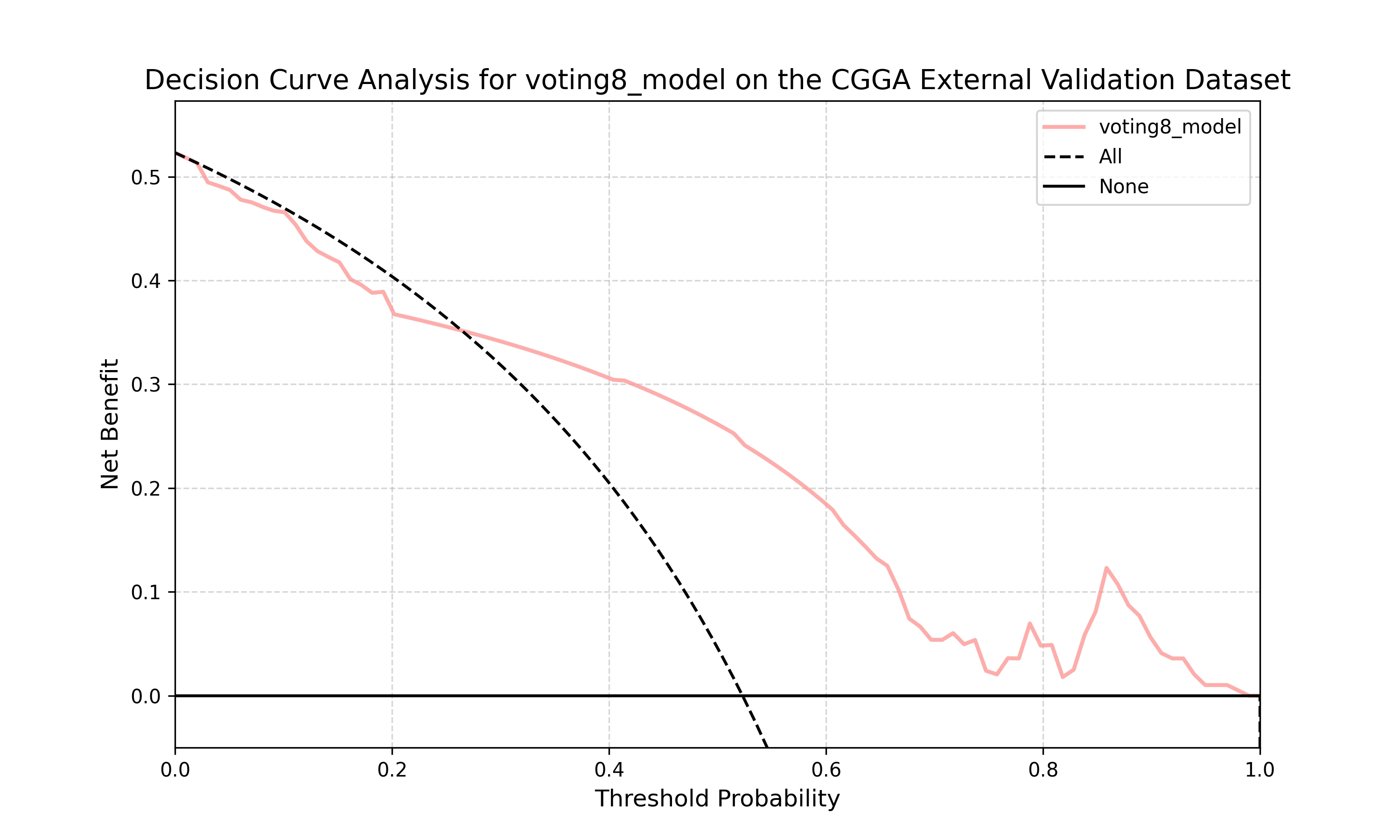

Supplement: S6 File — (ZIP) [file pone.0314831.s016.zip › S6 File/dca_curve_voting8_model.png]

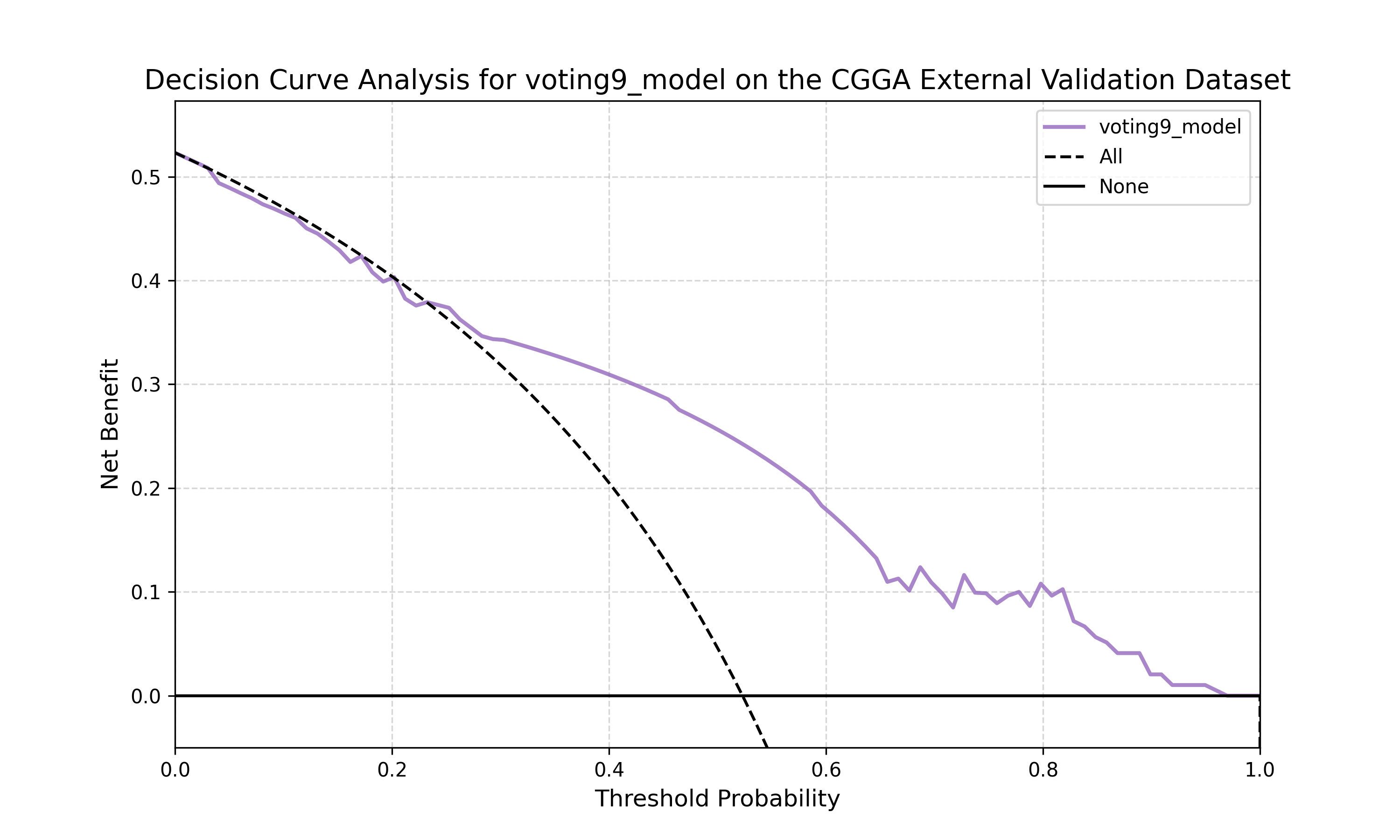

Supplement: S6 File — (ZIP) [file pone.0314831.s016.zip › S6 File/dca_curve_voting9_model.png]

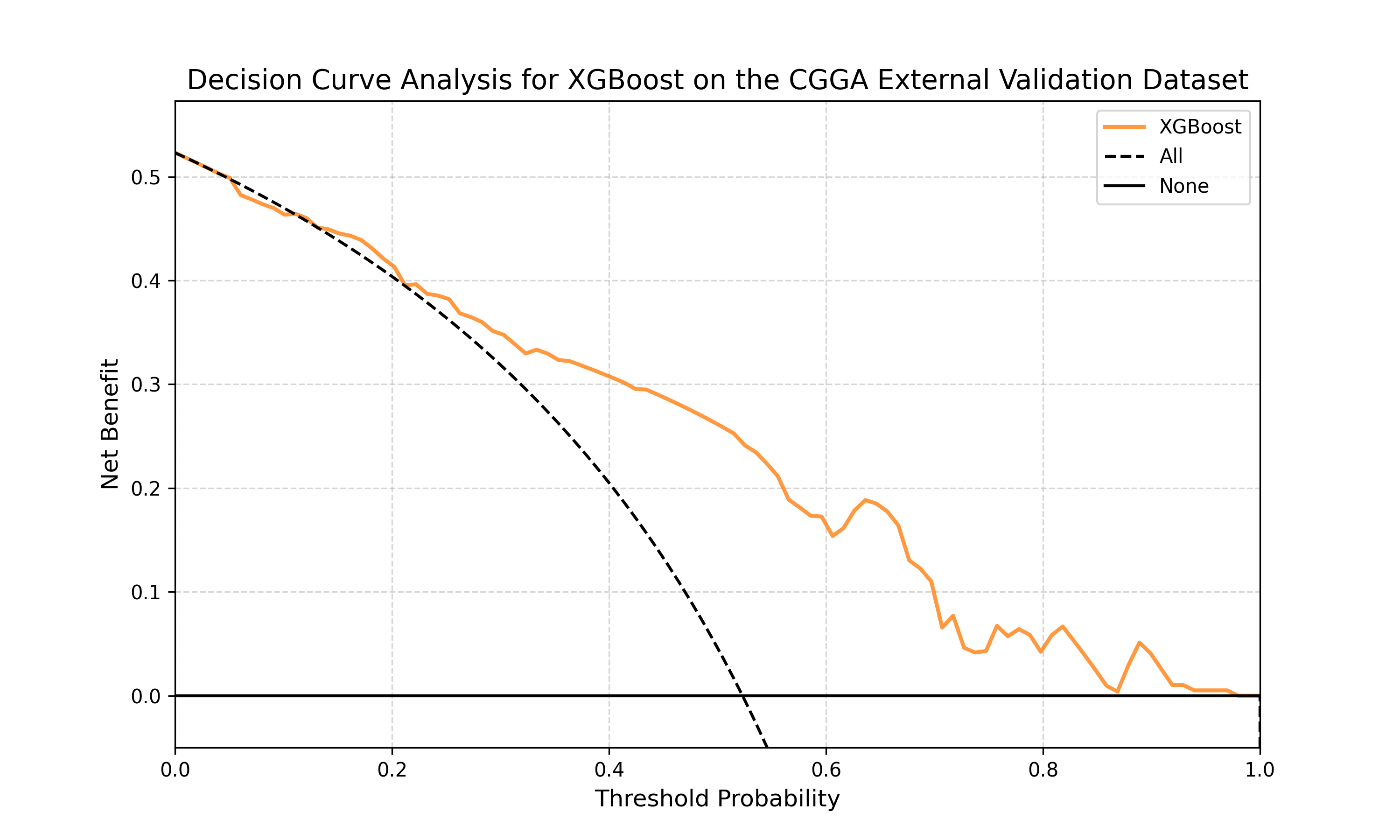

Supplement: S6 File — (ZIP) [file pone.0314831.s016.zip › S6 File/dca_curve_XGBoost.png]

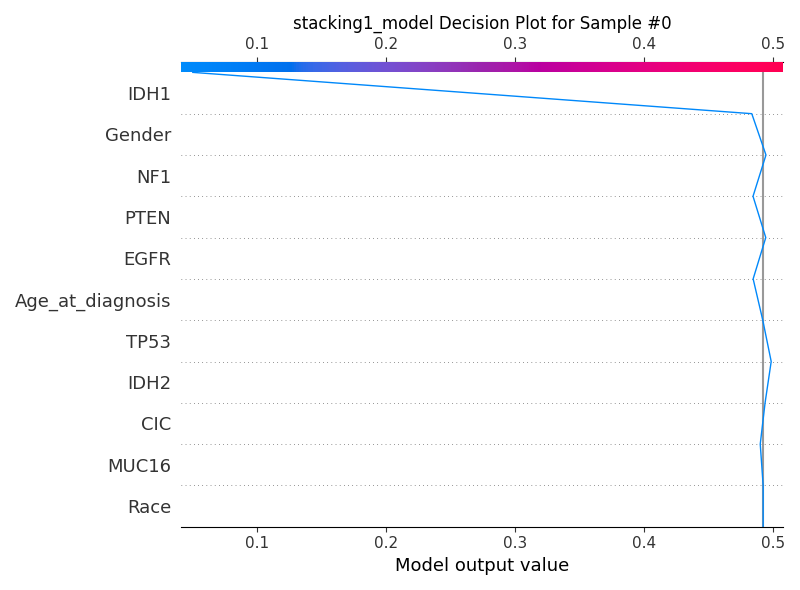

Supplement: S7 File — (ZIP) [file pone.0314831.s017.zip › S7 File/stacking1_model_decision_plot.png]

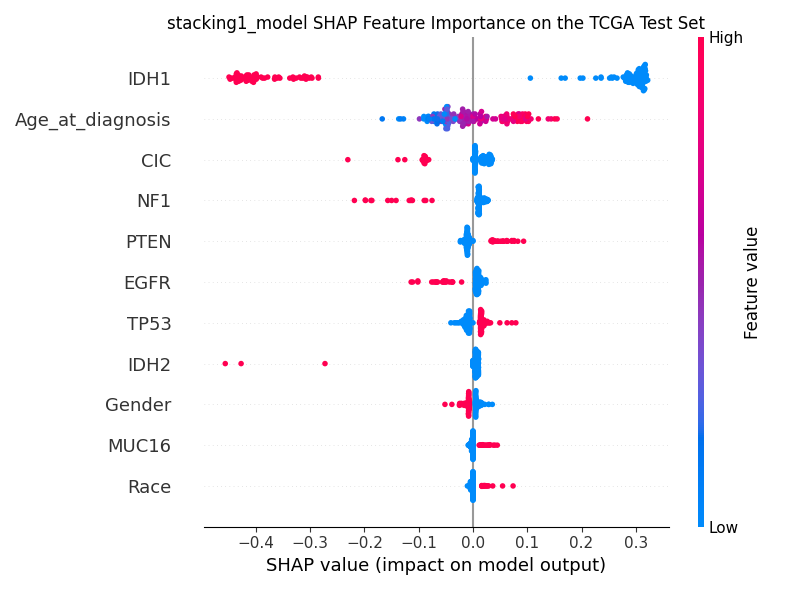

Supplement: S7 File — (ZIP) [file pone.0314831.s017.zip › S7 File/stacking1_model_feature_importance.png]

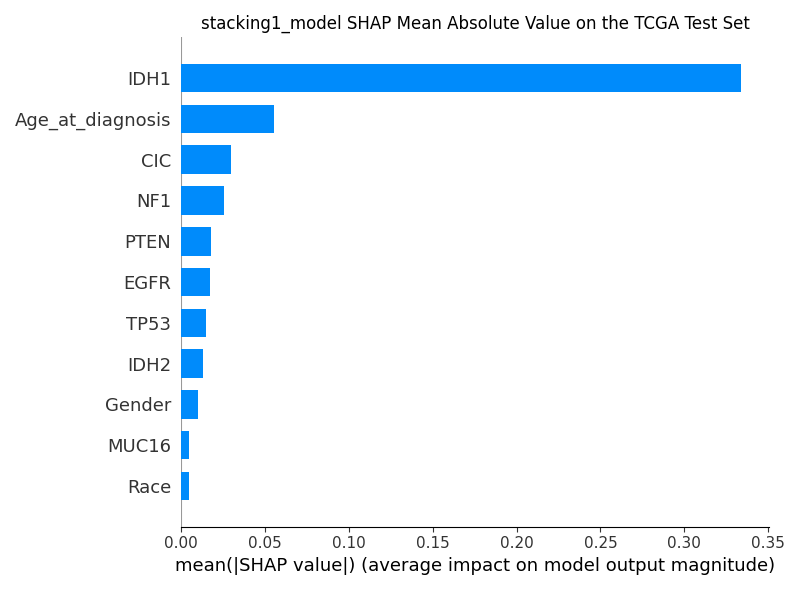

Supplement: S7 File — (ZIP) [file pone.0314831.s017.zip › S7 File/stacking1_model_feature_importance_bar.png]

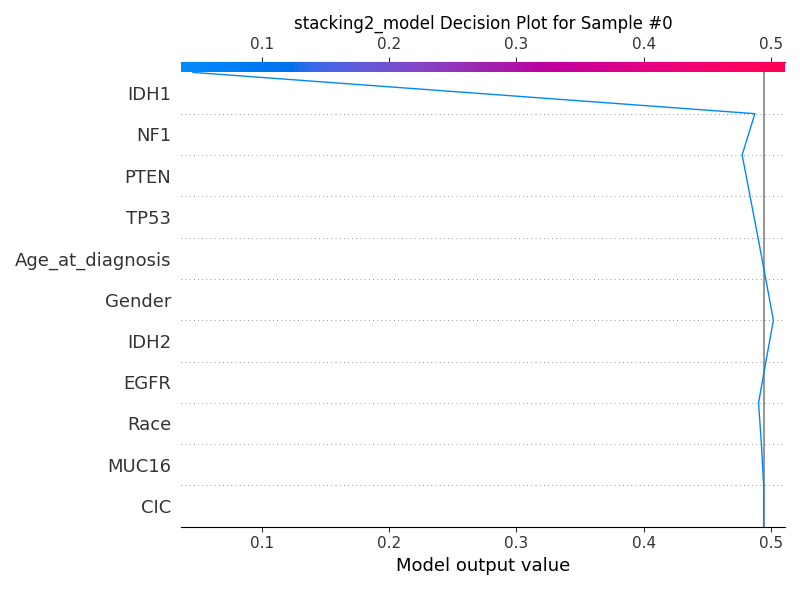

Supplement: S7 File — (ZIP) [file pone.0314831.s017.zip › S7 File/stacking2_model_decision_plot.png]

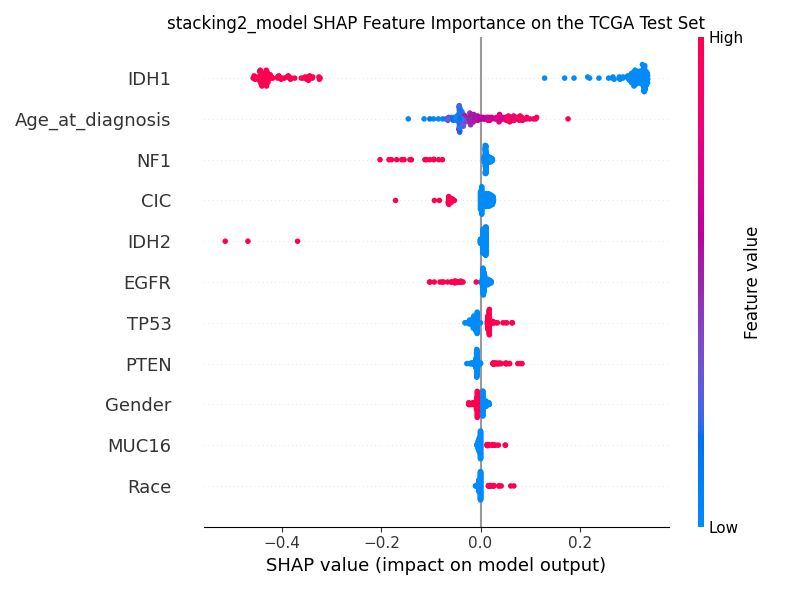

Supplement: S7 File — (ZIP) [file pone.0314831.s017.zip › S7 File/stacking2_model_feature_importance.png]

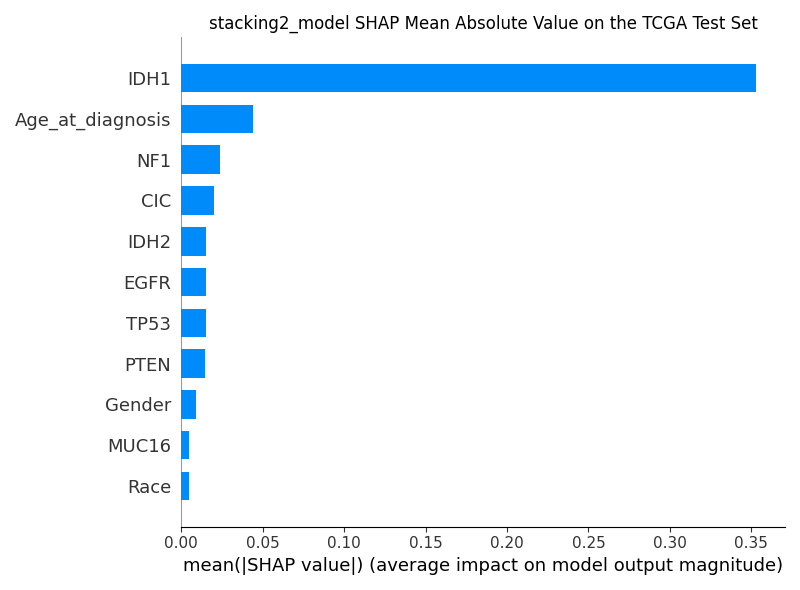

Supplement: S7 File — (ZIP) [file pone.0314831.s017.zip › S7 File/stacking2_model_feature_importance_bar.png]

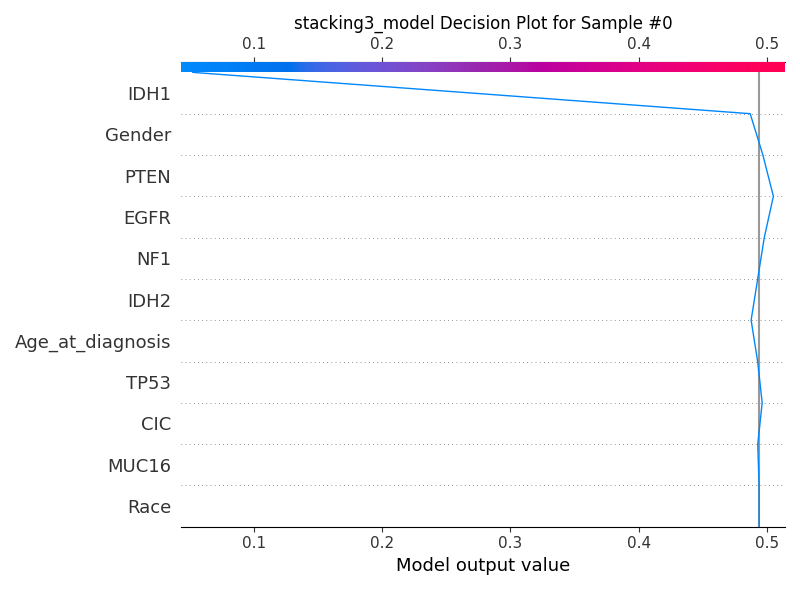

Supplement: S7 File — (ZIP) [file pone.0314831.s017.zip › S7 File/stacking3_model_decision_plot.png]

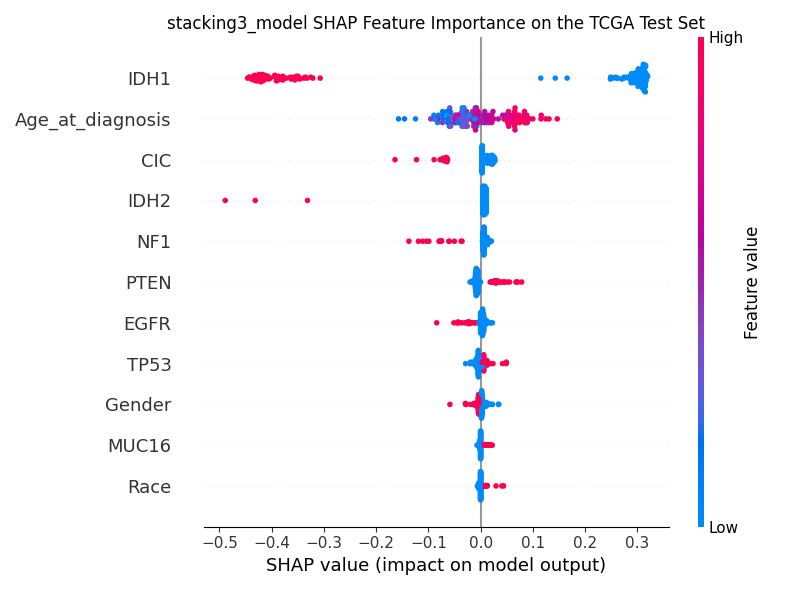

Supplement: S7 File — (ZIP) [file pone.0314831.s017.zip › S7 File/stacking3_model_feature_importance.png]

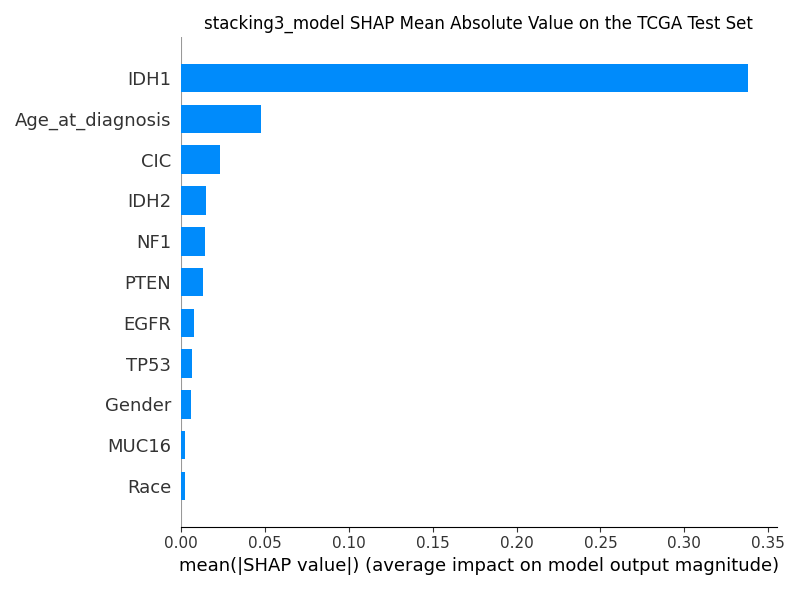

Supplement: S7 File — (ZIP) [file pone.0314831.s017.zip › S7 File/stacking3_model_feature_importance_bar.png]

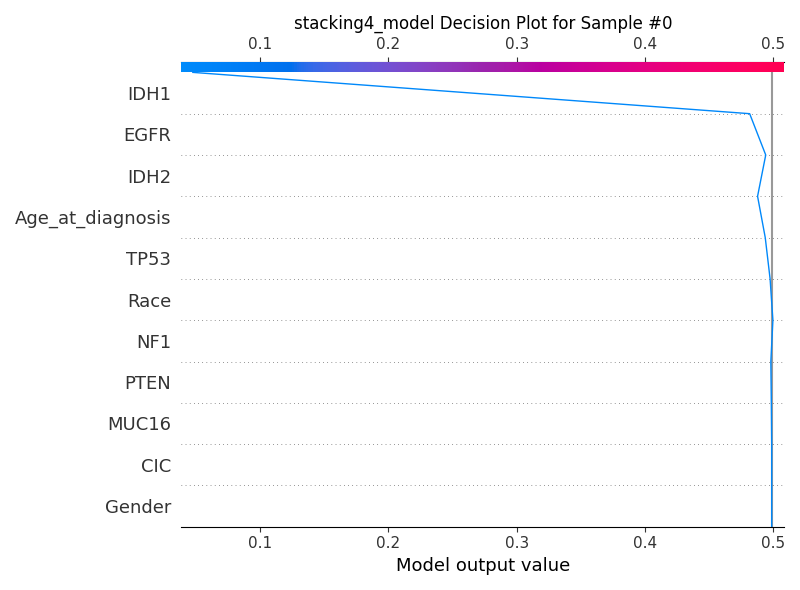

Supplement: S7 File — (ZIP) [file pone.0314831.s017.zip › S7 File/stacking4_model_decision_plot.png]

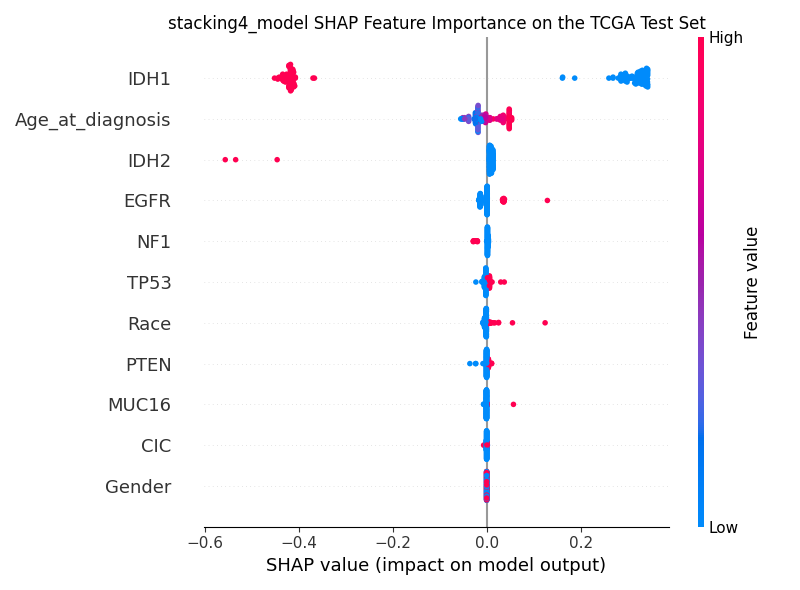

Supplement: S7 File — (ZIP) [file pone.0314831.s017.zip › S7 File/stacking4_model_feature_importance.png]

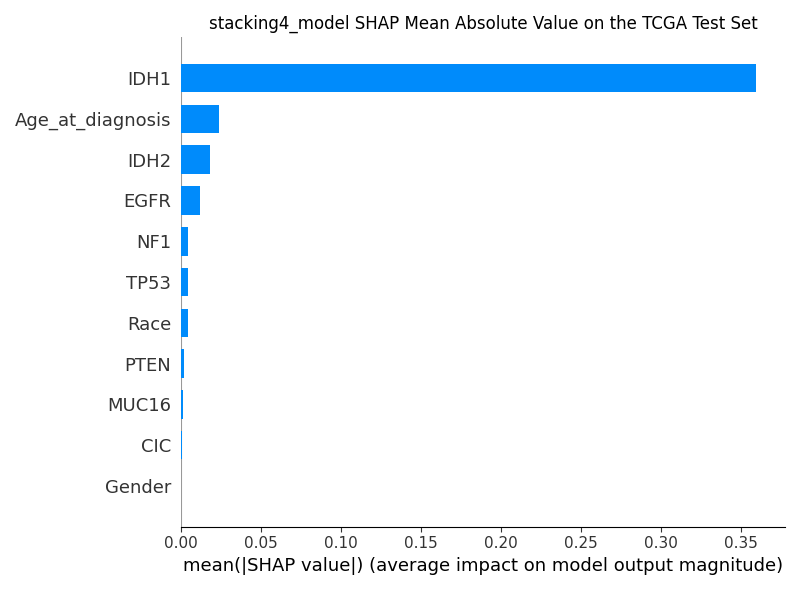

Supplement: S7 File — (ZIP) [file pone.0314831.s017.zip › S7 File/stacking4_model_feature_importance_bar.png]

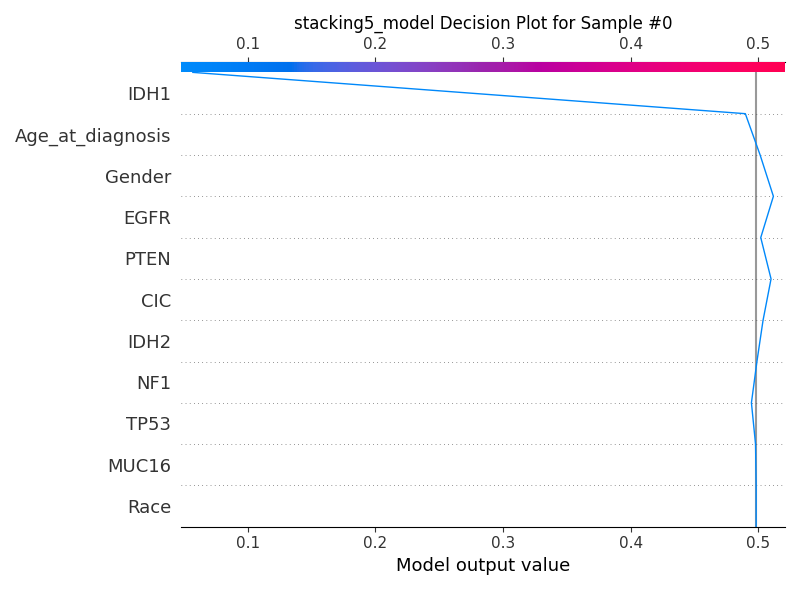

Supplement: S7 File — (ZIP) [file pone.0314831.s017.zip › S7 File/stacking5_model_decision_plot.png]

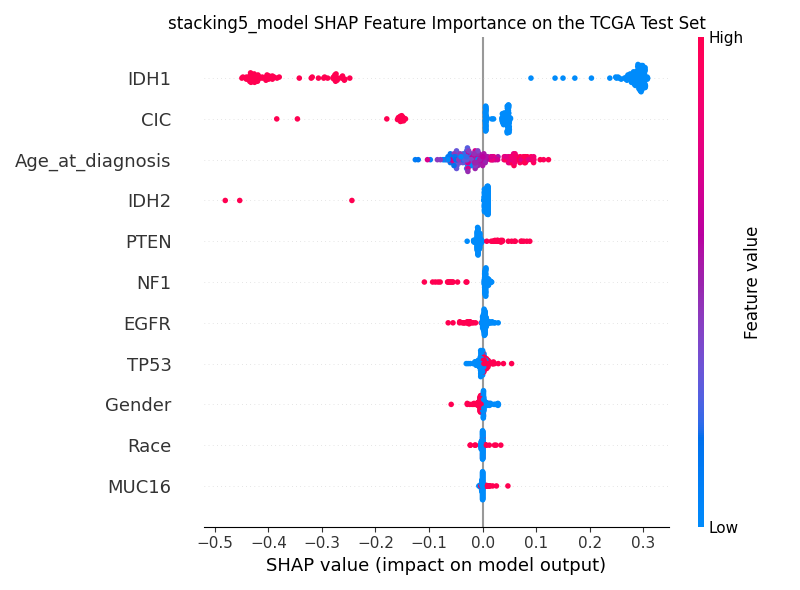

Supplement: S7 File — (ZIP) [file pone.0314831.s017.zip › S7 File/stacking5_model_feature_importance.png]

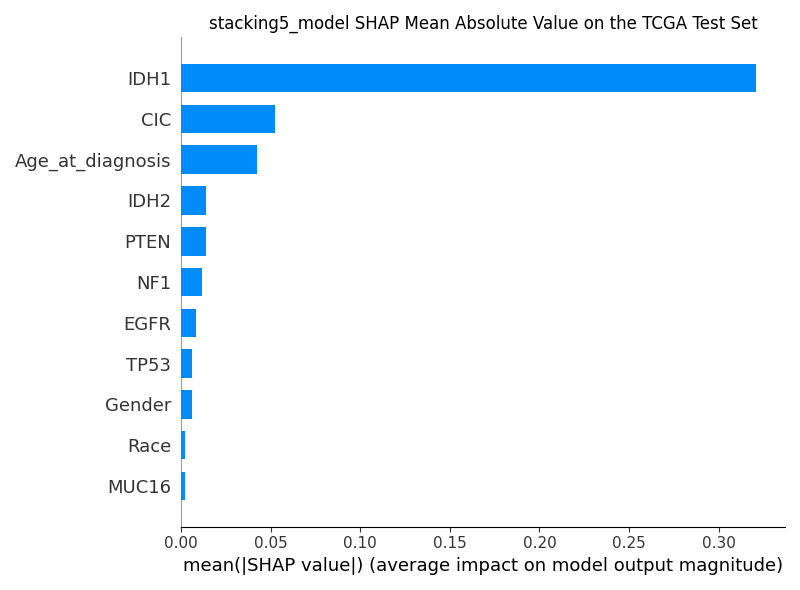

Supplement: S7 File — (ZIP) [file pone.0314831.s017.zip › S7 File/stacking5_model_feature_importance_bar.png]

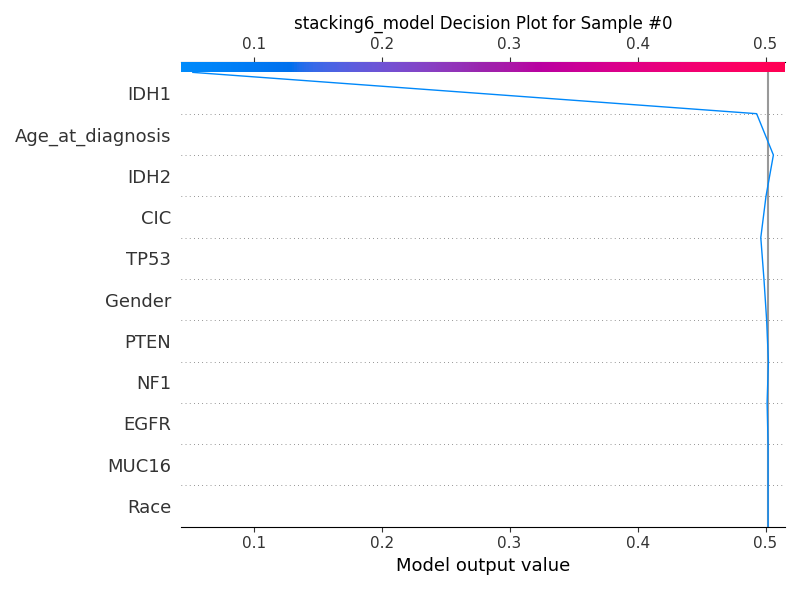

Supplement: S7 File — (ZIP) [file pone.0314831.s017.zip › S7 File/stacking6_model_decision_plot.png]

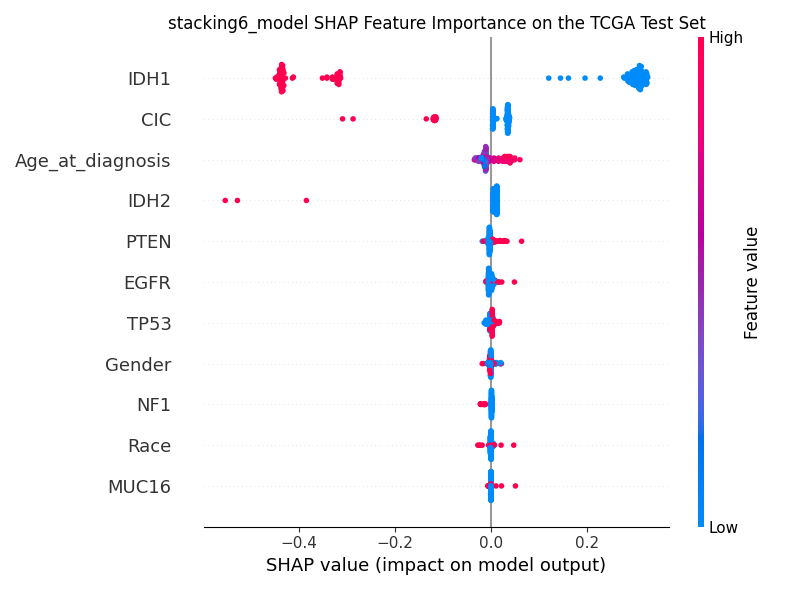

Supplement: S7 File — (ZIP) [file pone.0314831.s017.zip › S7 File/stacking6_model_feature_importance.png]

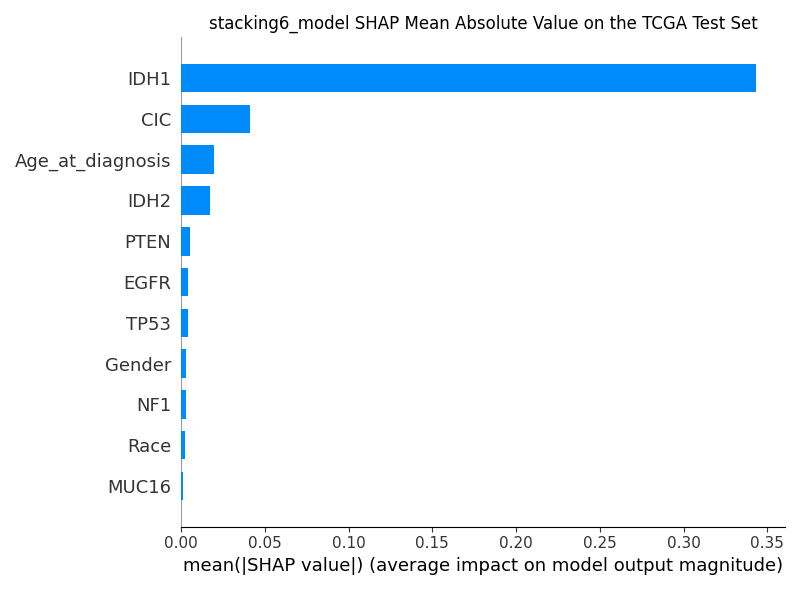

Supplement: S7 File — (ZIP) [file pone.0314831.s017.zip › S7 File/stacking6_model_feature_importance_bar.png]

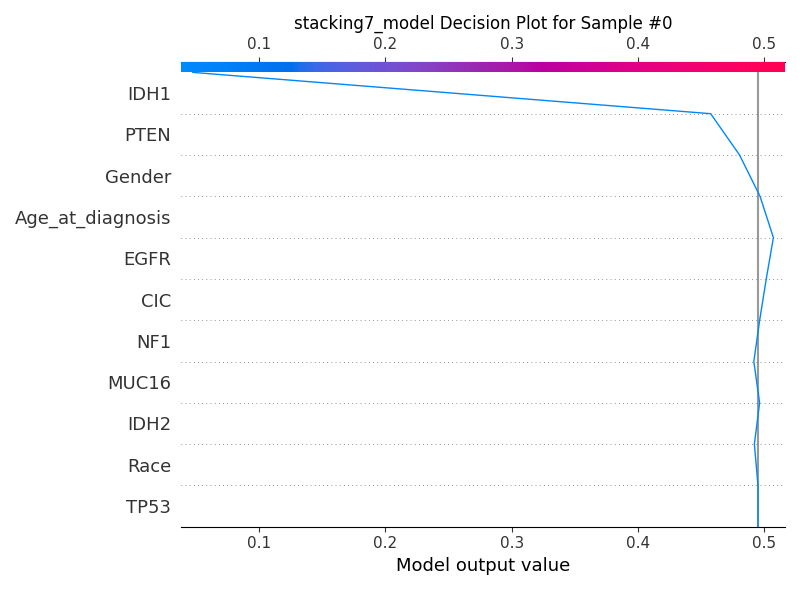

Supplement: S7 File — (ZIP) [file pone.0314831.s017.zip › S7 File/stacking7_model_decision_plot.png]

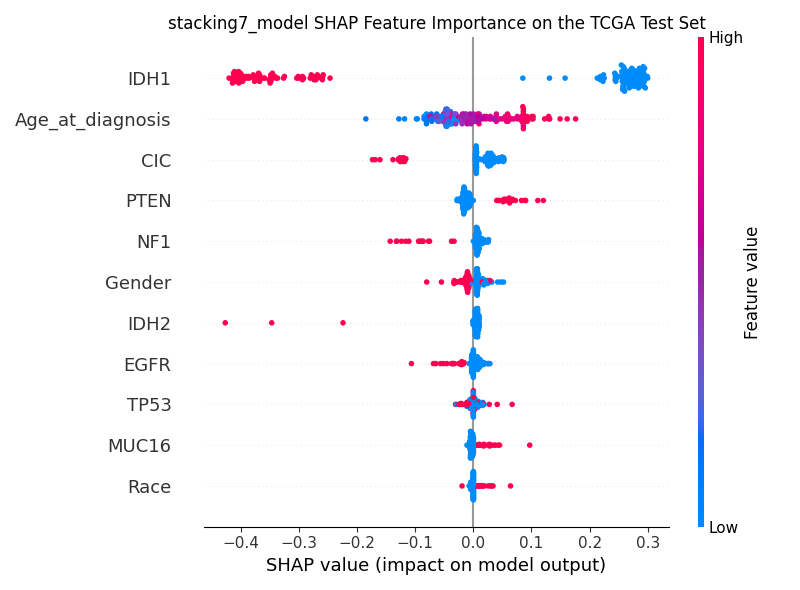

Supplement: S7 File — (ZIP) [file pone.0314831.s017.zip › S7 File/stacking7_model_feature_importance.png]

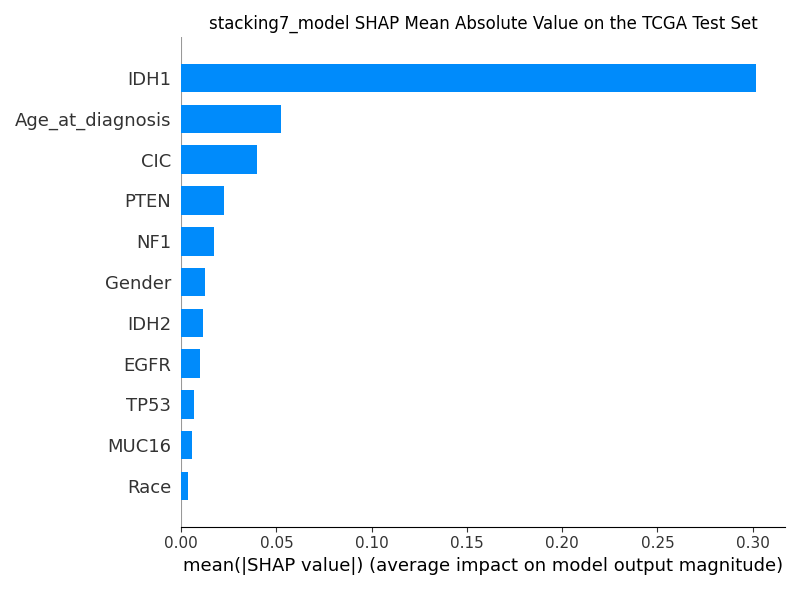

Supplement: S7 File — (ZIP) [file pone.0314831.s017.zip › S7 File/stacking7_model_feature_importance_bar.png]

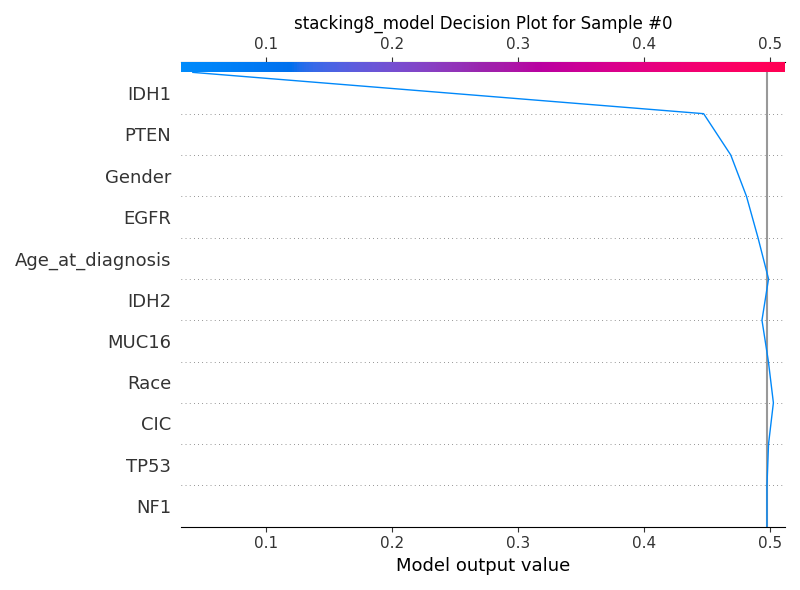

Supplement: S7 File — (ZIP) [file pone.0314831.s017.zip › S7 File/stacking8_model_decision_plot.png]

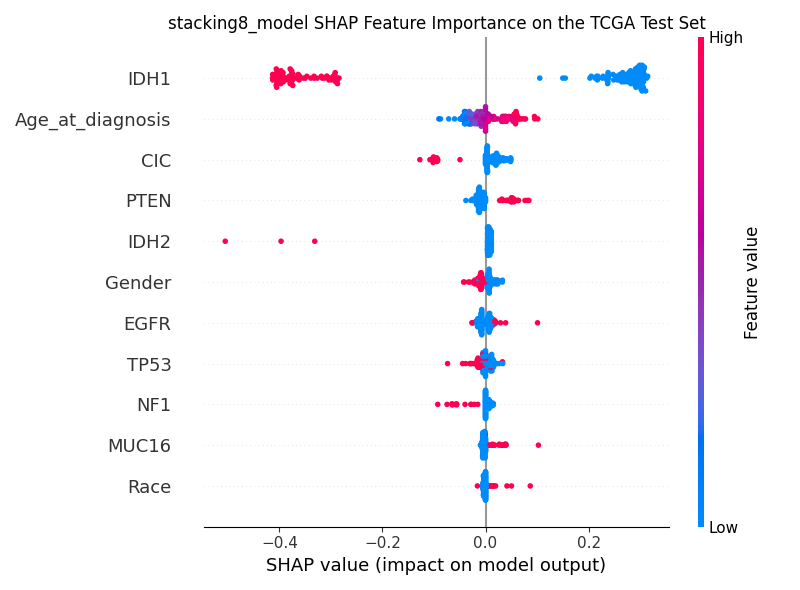

Supplement: S7 File — (ZIP) [file pone.0314831.s017.zip › S7 File/stacking8_model_feature_importance.png]

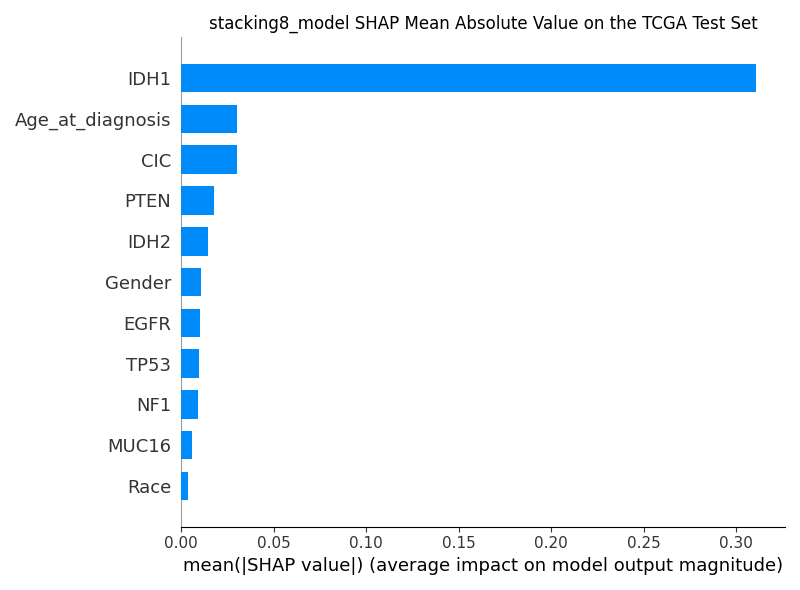

Supplement: S7 File — (ZIP) [file pone.0314831.s017.zip › S7 File/stacking8_model_feature_importance_bar.png]

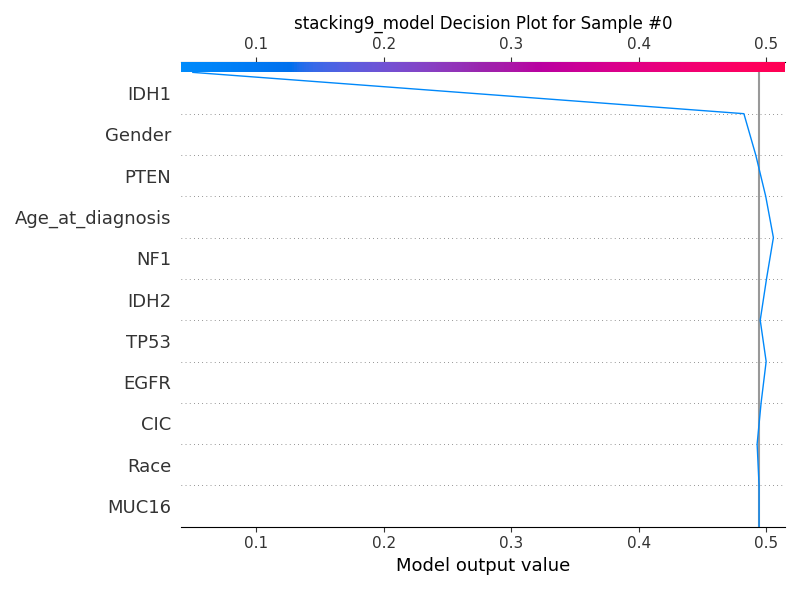

Supplement: S7 File — (ZIP) [file pone.0314831.s017.zip › S7 File/stacking9_model_decision_plot.png]

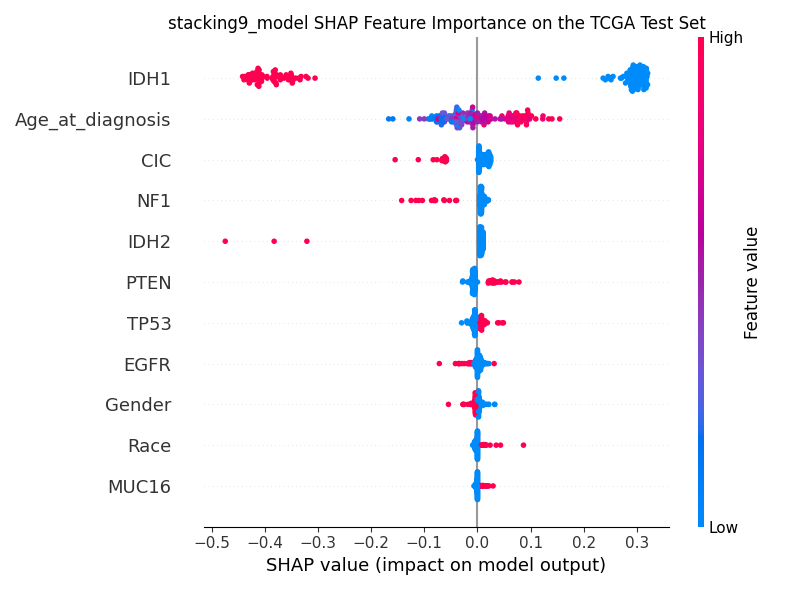

Supplement: S7 File — (ZIP) [file pone.0314831.s017.zip › S7 File/stacking9_model_feature_importance.png]

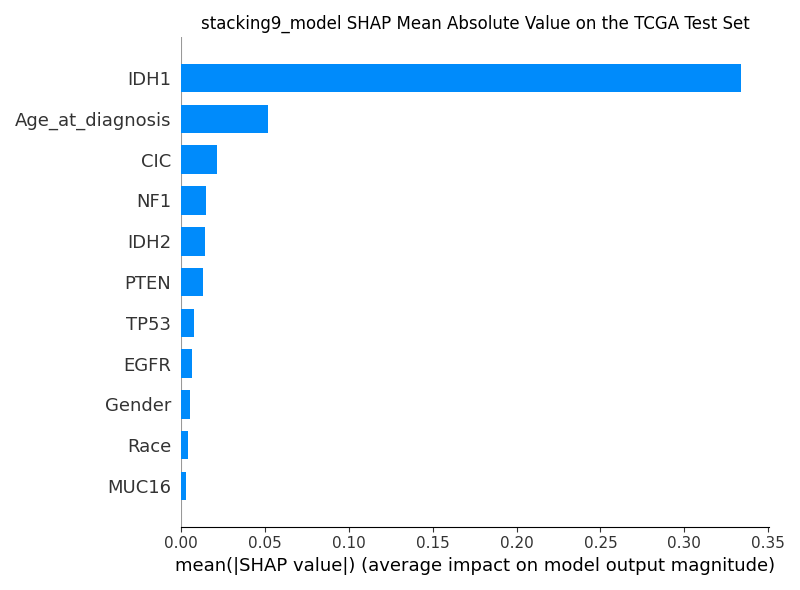

Supplement: S7 File — (ZIP) [file pone.0314831.s017.zip › S7 File/stacking9_model_feature_importance_bar.png]
