# Supplementary figures and images for: Machine learning-based prediction of glioma grading (part 3 of 5)
Source: PLoS One. 2025 Dec 26;20(12):e0314831. doi: 10.1371/journal.pone.0314831 (PMC12742763; doi:10.1371/journal.pone.0314831)

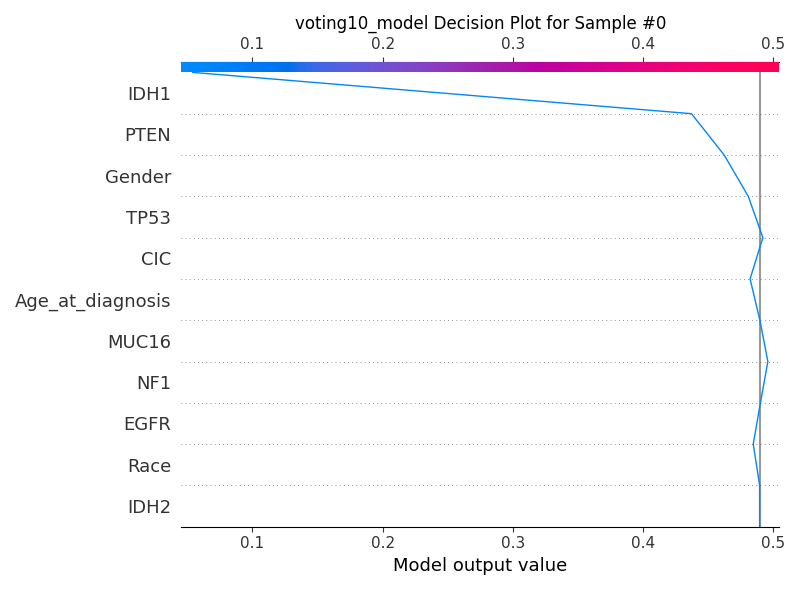

Supplement: S7 File — (ZIP) [file pone.0314831.s017.zip › S7 File/voting10_model_decision_plot.png]

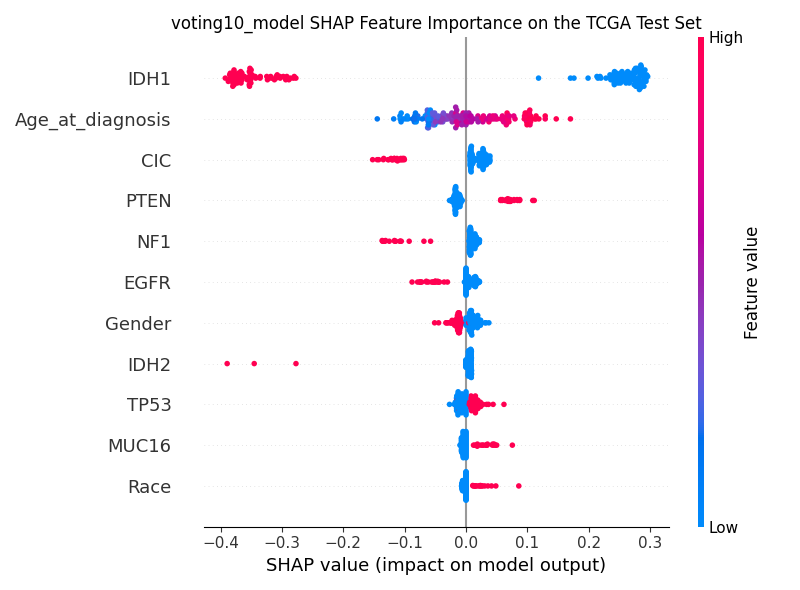

Supplement: S7 File — (ZIP) [file pone.0314831.s017.zip › S7 File/voting10_model_feature_importance.png]

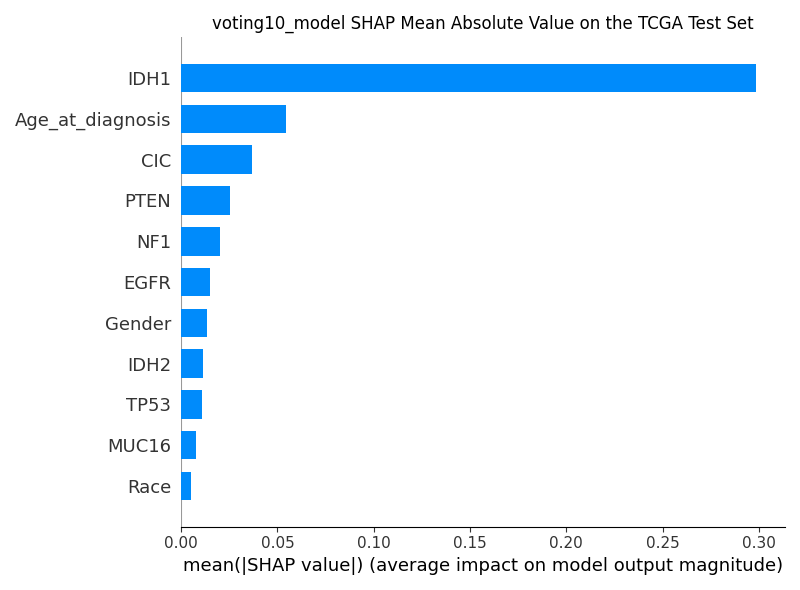

Supplement: S7 File — (ZIP) [file pone.0314831.s017.zip › S7 File/voting10_model_feature_importance_bar.png]

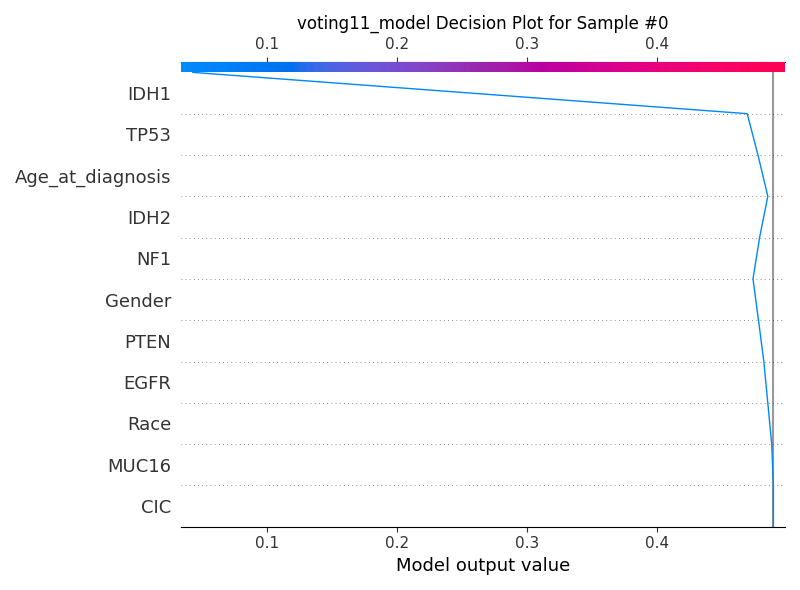

Supplement: S7 File — (ZIP) [file pone.0314831.s017.zip › S7 File/voting11_model_decision_plot.png]

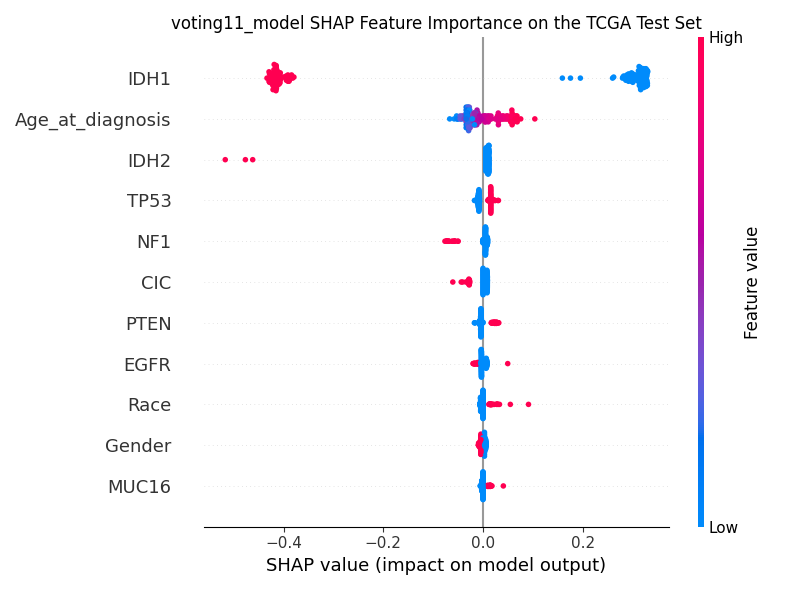

Supplement: S7 File — (ZIP) [file pone.0314831.s017.zip › S7 File/voting11_model_feature_importance.png]

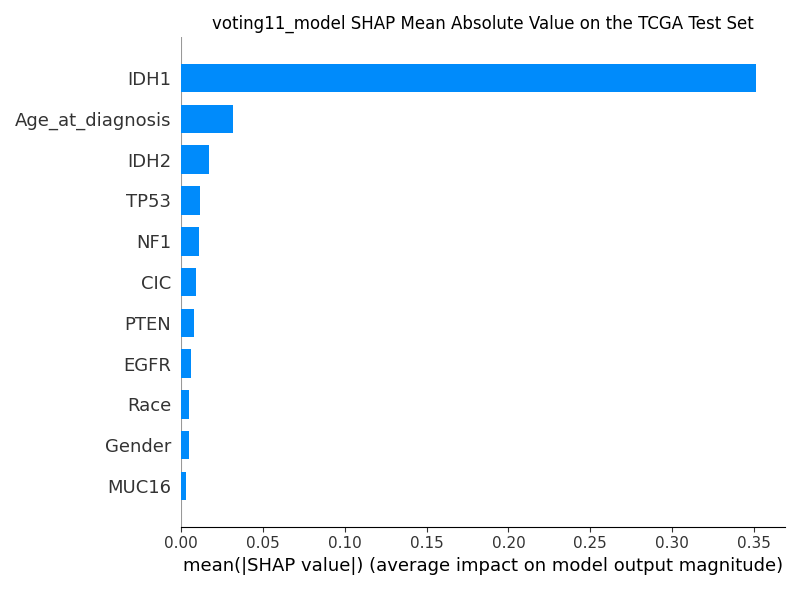

Supplement: S7 File — (ZIP) [file pone.0314831.s017.zip › S7 File/voting11_model_feature_importance_bar.png]

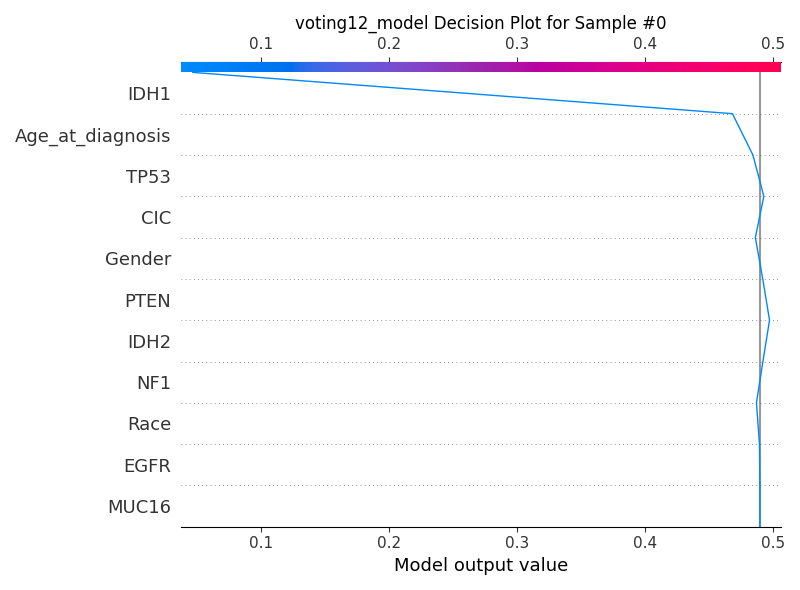

Supplement: S7 File — (ZIP) [file pone.0314831.s017.zip › S7 File/voting12_model_decision_plot.png]

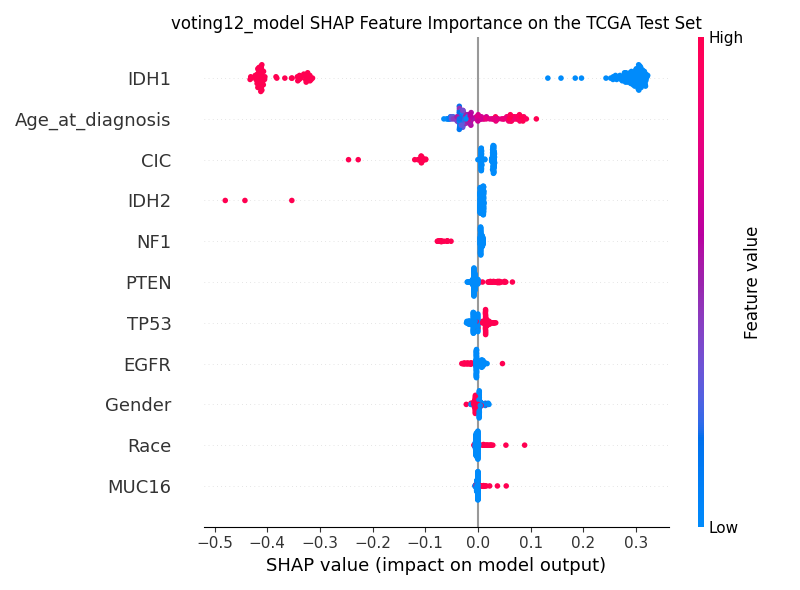

Supplement: S7 File — (ZIP) [file pone.0314831.s017.zip › S7 File/voting12_model_feature_importance.png]

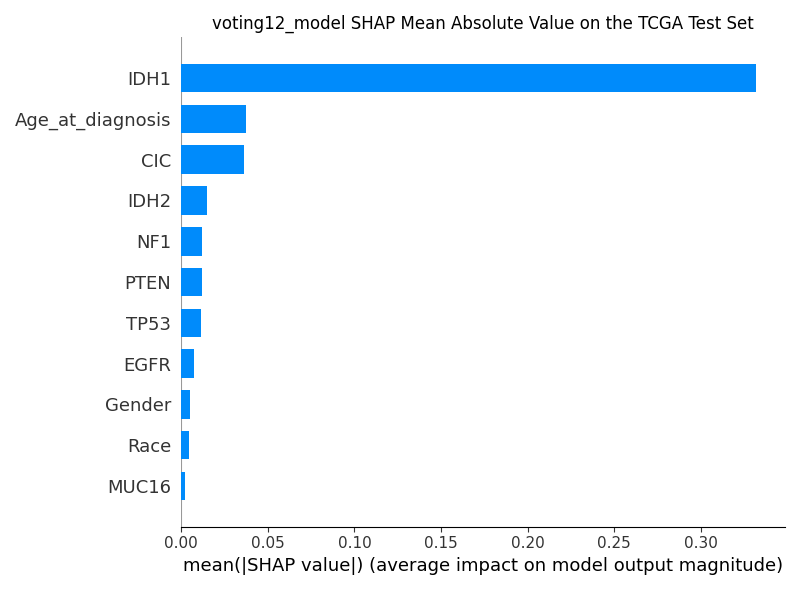

Supplement: S7 File — (ZIP) [file pone.0314831.s017.zip › S7 File/voting12_model_feature_importance_bar.png]

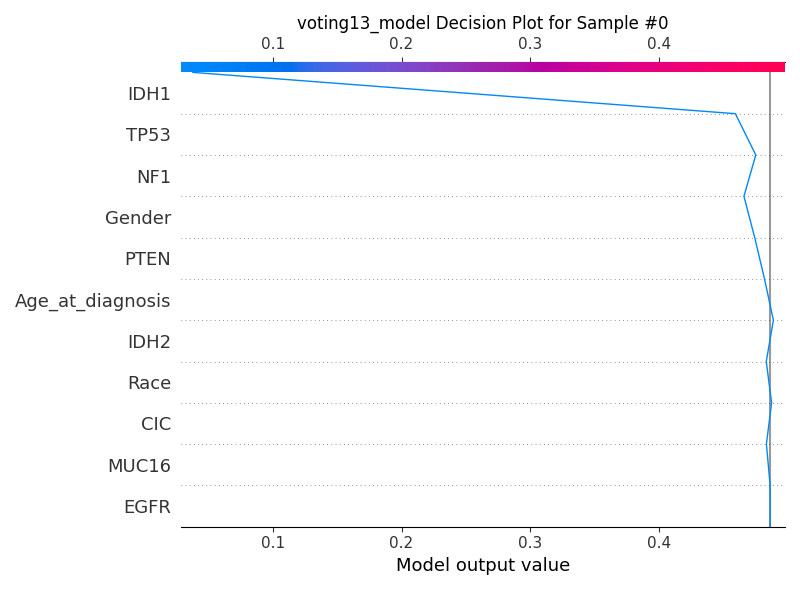

Supplement: S7 File — (ZIP) [file pone.0314831.s017.zip › S7 File/voting13_model_decision_plot.png]

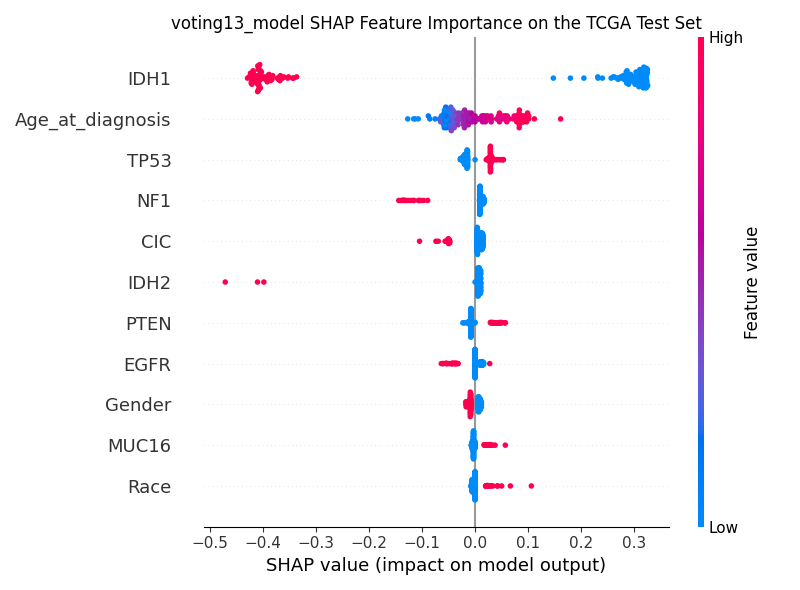

Supplement: S7 File — (ZIP) [file pone.0314831.s017.zip › S7 File/voting13_model_feature_importance.png]

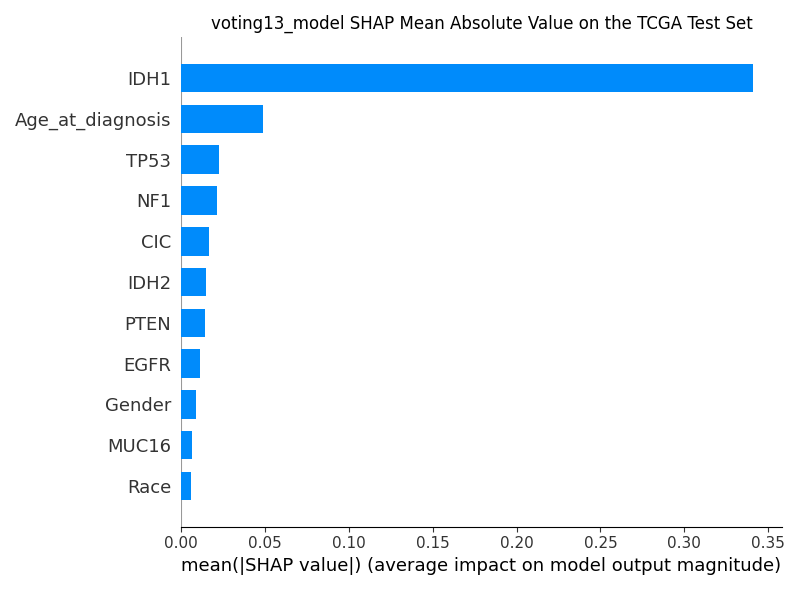

Supplement: S7 File — (ZIP) [file pone.0314831.s017.zip › S7 File/voting13_model_feature_importance_bar.png]

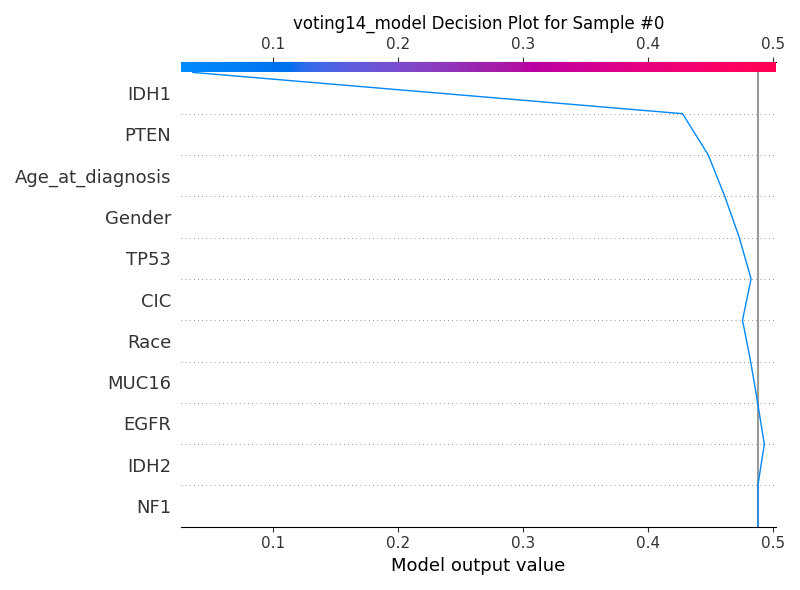

Supplement: S7 File — (ZIP) [file pone.0314831.s017.zip › S7 File/voting14_model_decision_plot.png]

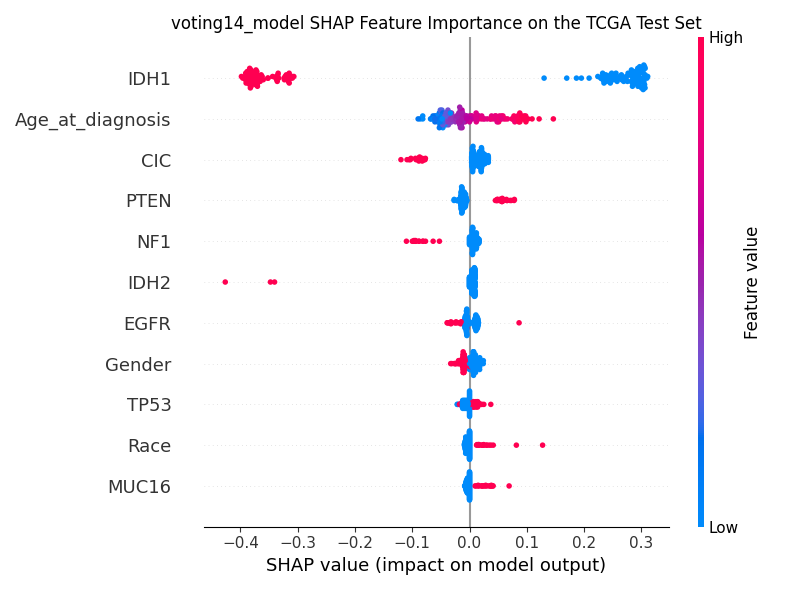

Supplement: S7 File — (ZIP) [file pone.0314831.s017.zip › S7 File/voting14_model_feature_importance.png]

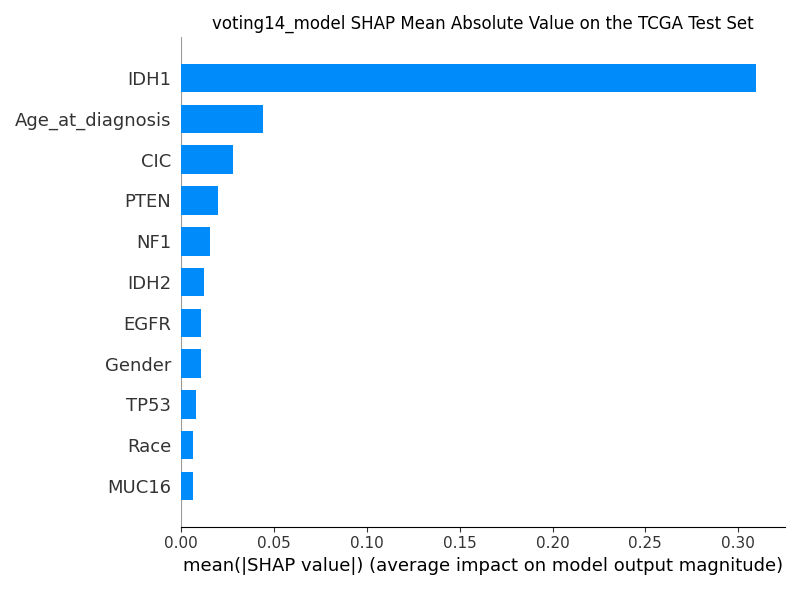

Supplement: S7 File — (ZIP) [file pone.0314831.s017.zip › S7 File/voting14_model_feature_importance_bar.png]

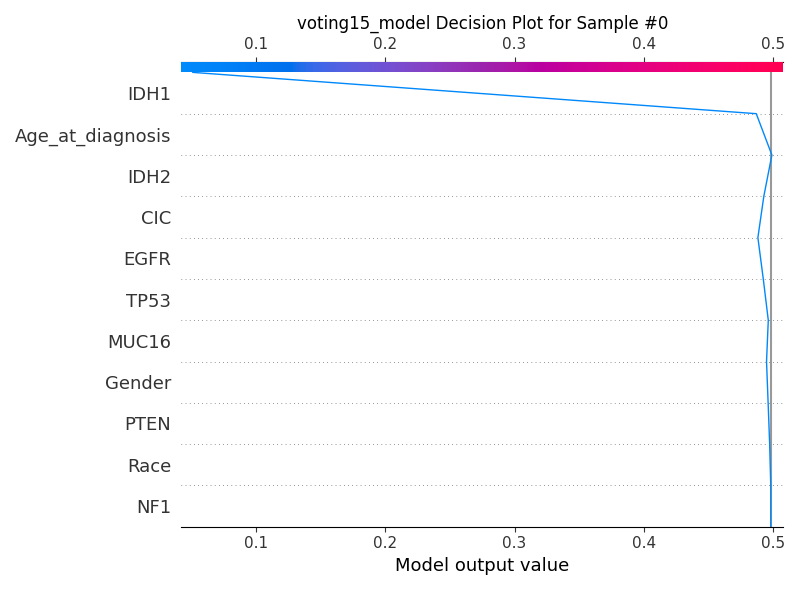

Supplement: S7 File — (ZIP) [file pone.0314831.s017.zip › S7 File/voting15_model_decision_plot.png]

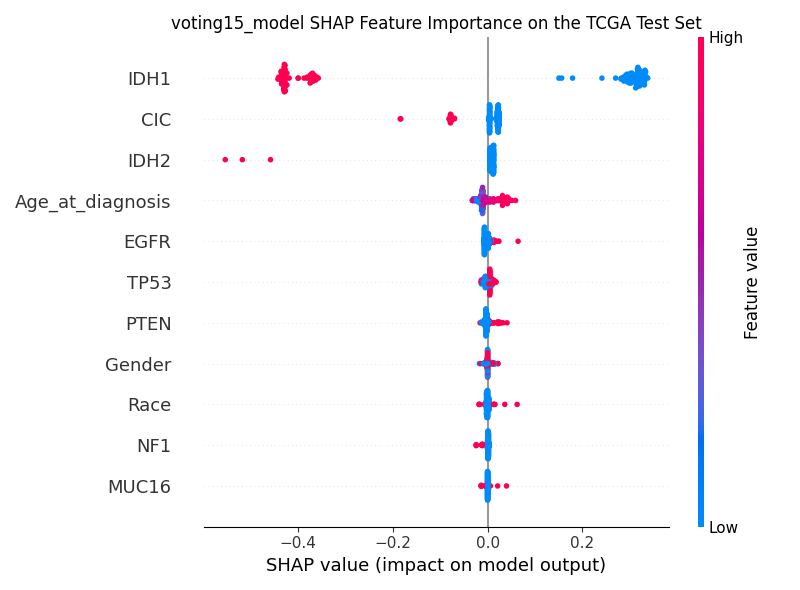

Supplement: S7 File — (ZIP) [file pone.0314831.s017.zip › S7 File/voting15_model_feature_importance.png]

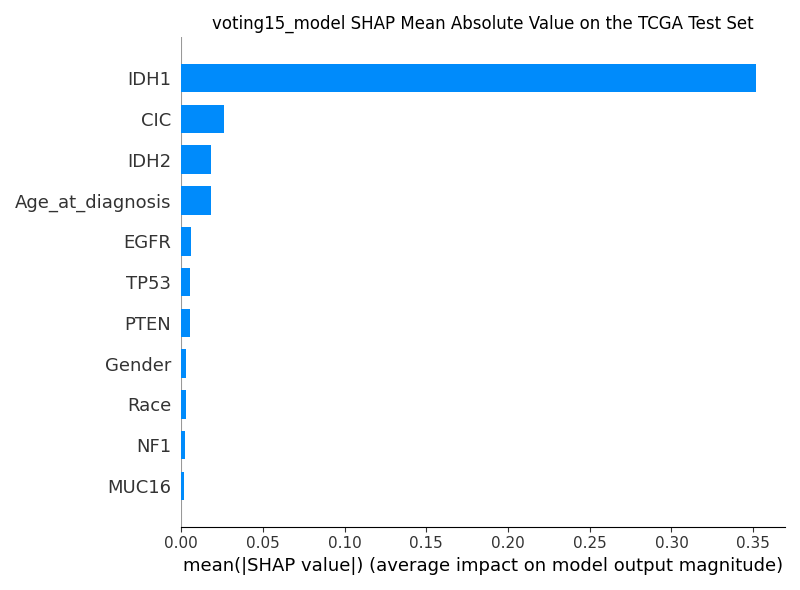

Supplement: S7 File — (ZIP) [file pone.0314831.s017.zip › S7 File/voting15_model_feature_importance_bar.png]

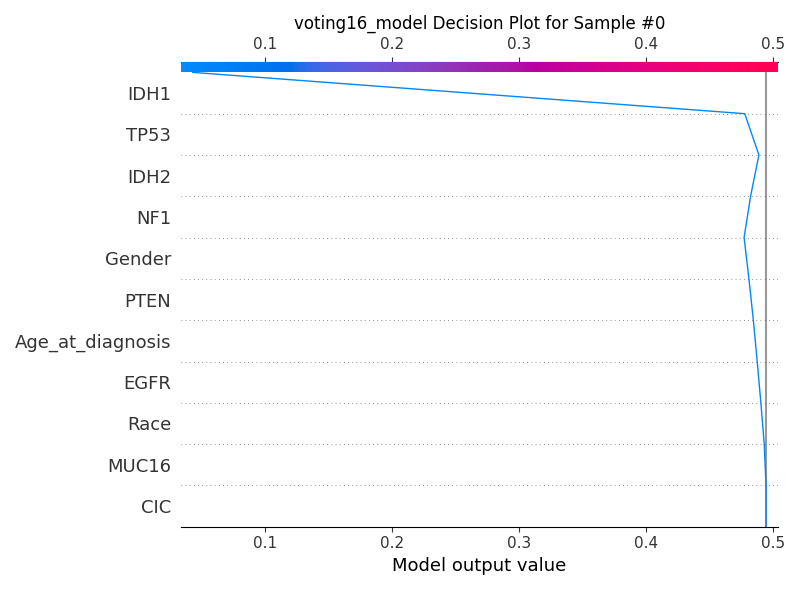

Supplement: S7 File — (ZIP) [file pone.0314831.s017.zip › S7 File/voting16_model_decision_plot.png]

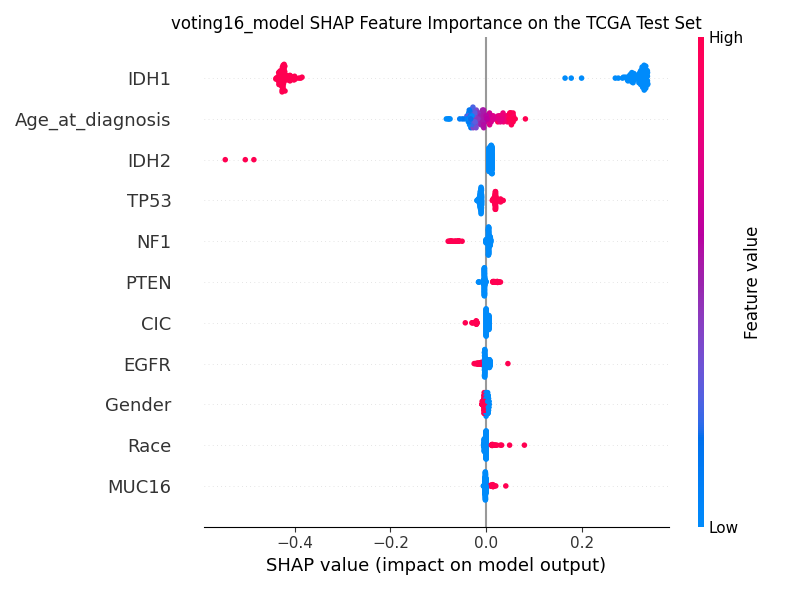

Supplement: S7 File — (ZIP) [file pone.0314831.s017.zip › S7 File/voting16_model_feature_importance.png]

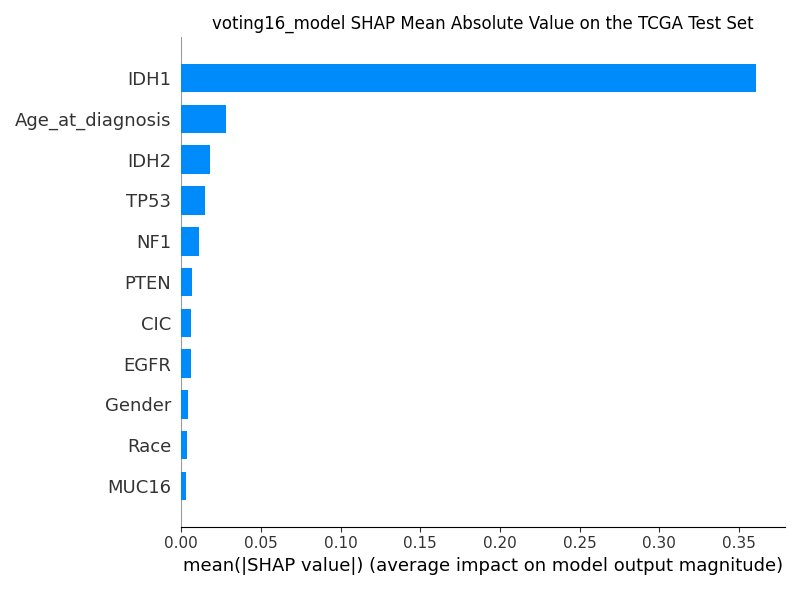

Supplement: S7 File — (ZIP) [file pone.0314831.s017.zip › S7 File/voting16_model_feature_importance_bar.png]

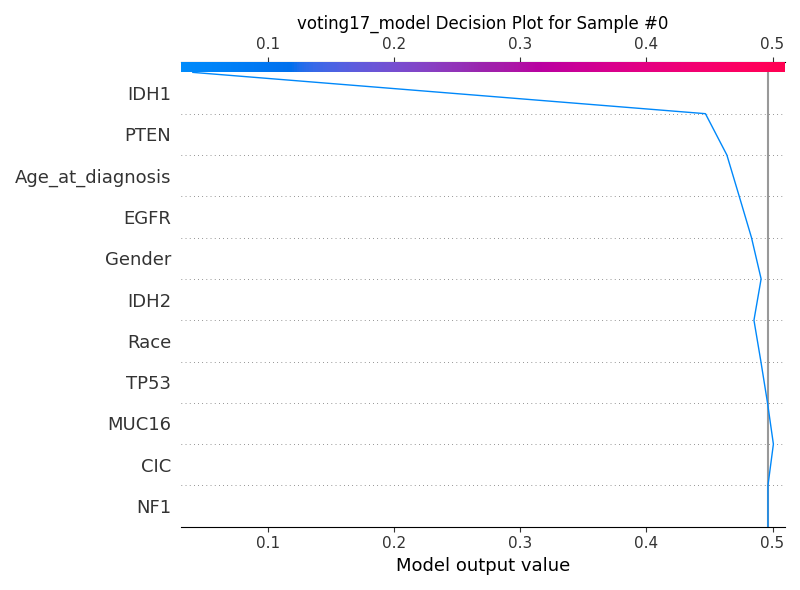

Supplement: S7 File — (ZIP) [file pone.0314831.s017.zip › S7 File/voting17_model_decision_plot.png]

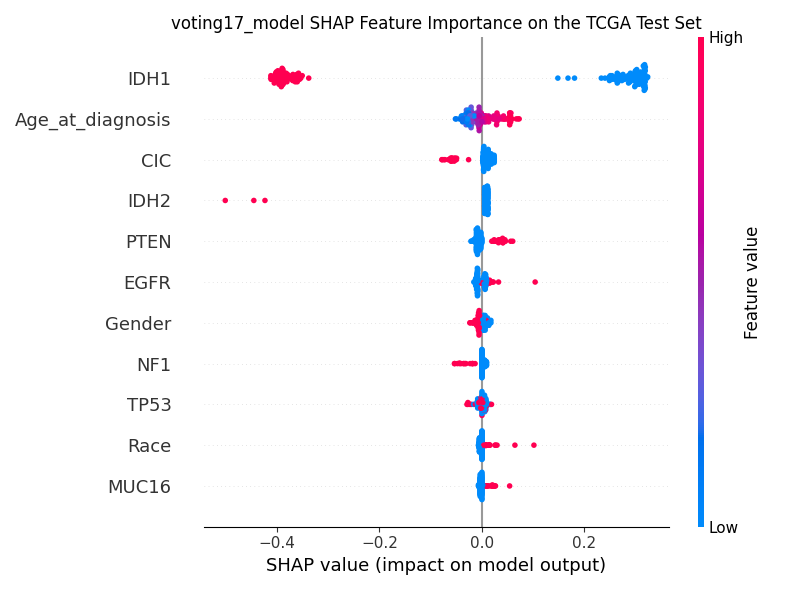

Supplement: S7 File — (ZIP) [file pone.0314831.s017.zip › S7 File/voting17_model_feature_importance.png]

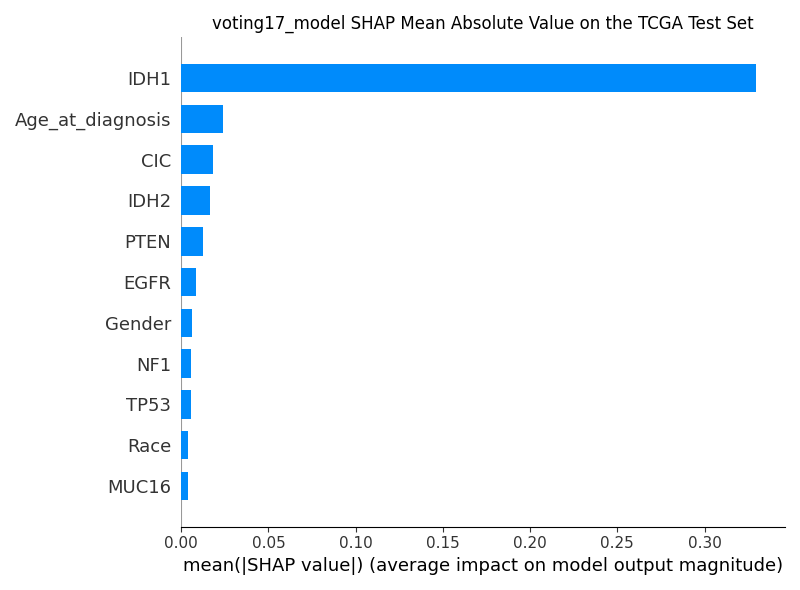

Supplement: S7 File — (ZIP) [file pone.0314831.s017.zip › S7 File/voting17_model_feature_importance_bar.png]

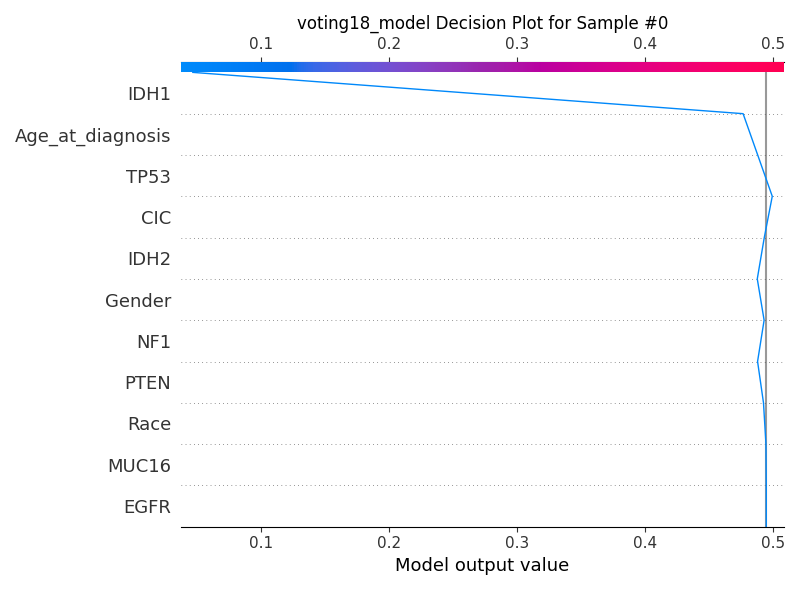

Supplement: S7 File — (ZIP) [file pone.0314831.s017.zip › S7 File/voting18_model_decision_plot.png]

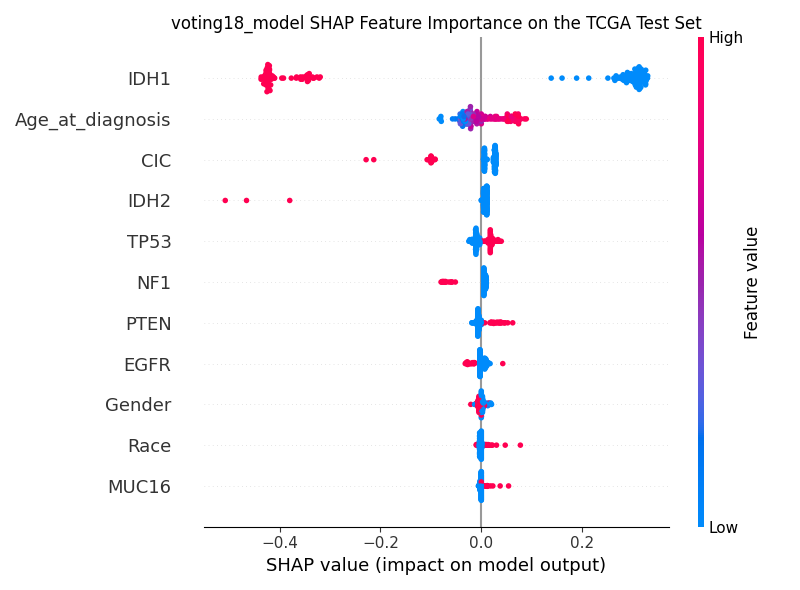

Supplement: S7 File — (ZIP) [file pone.0314831.s017.zip › S7 File/voting18_model_feature_importance.png]

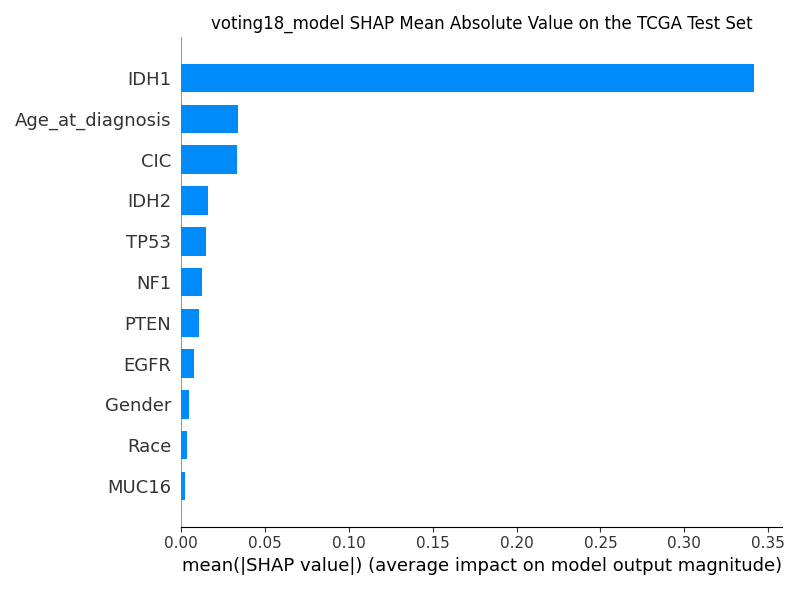

Supplement: S7 File — (ZIP) [file pone.0314831.s017.zip › S7 File/voting18_model_feature_importance_bar.png]

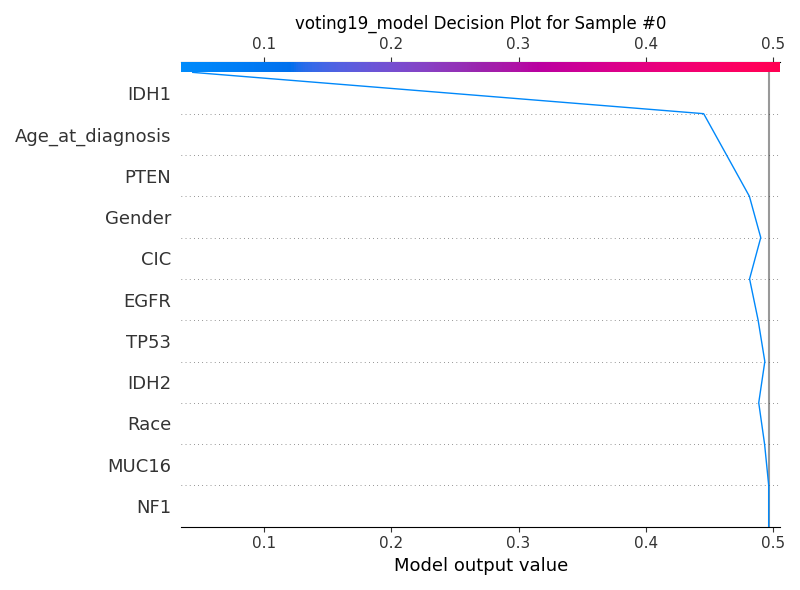

Supplement: S7 File — (ZIP) [file pone.0314831.s017.zip › S7 File/voting19_model_decision_plot.png]

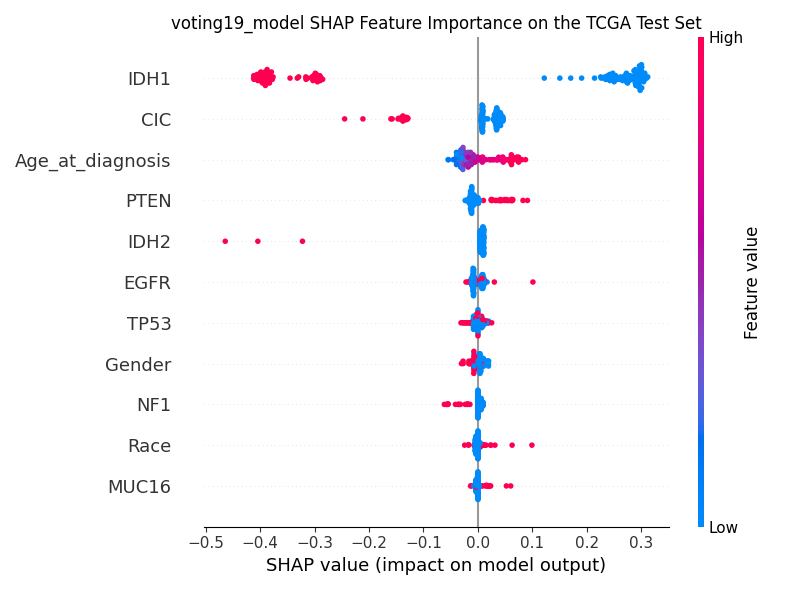

Supplement: S7 File — (ZIP) [file pone.0314831.s017.zip › S7 File/voting19_model_feature_importance.png]

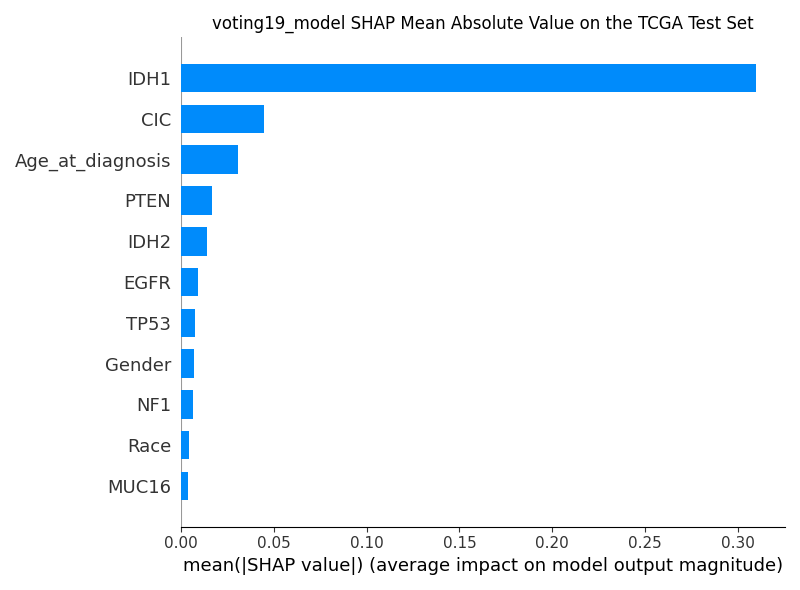

Supplement: S7 File — (ZIP) [file pone.0314831.s017.zip › S7 File/voting19_model_feature_importance_bar.png]

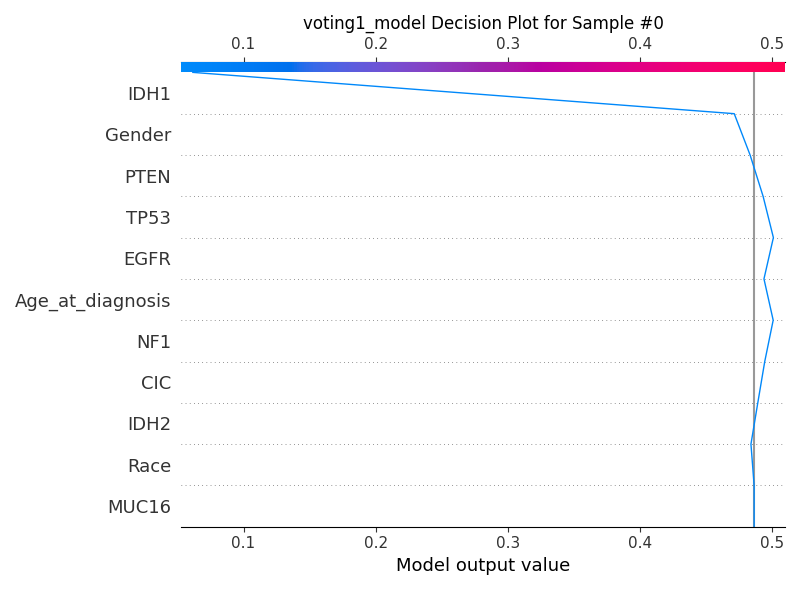

Supplement: S7 File — (ZIP) [file pone.0314831.s017.zip › S7 File/voting1_model_decision_plot.png]

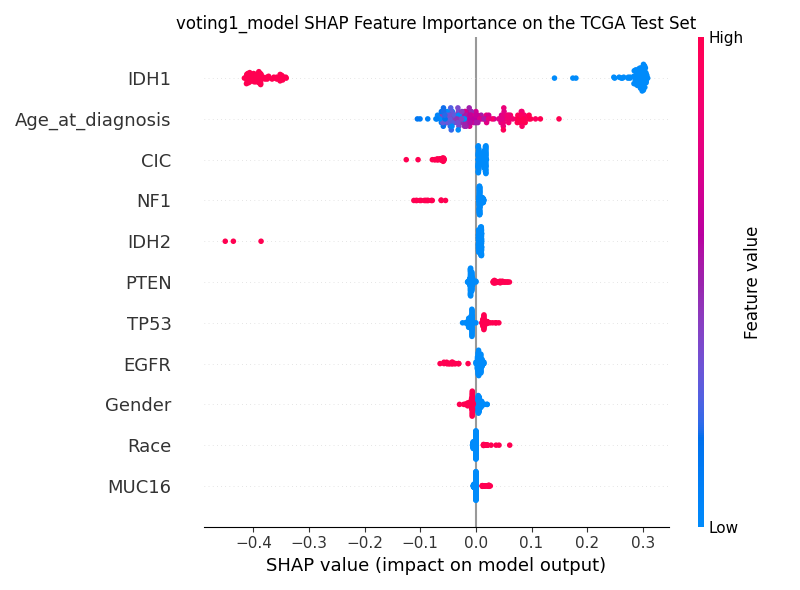

Supplement: S7 File — (ZIP) [file pone.0314831.s017.zip › S7 File/voting1_model_feature_importance.png]

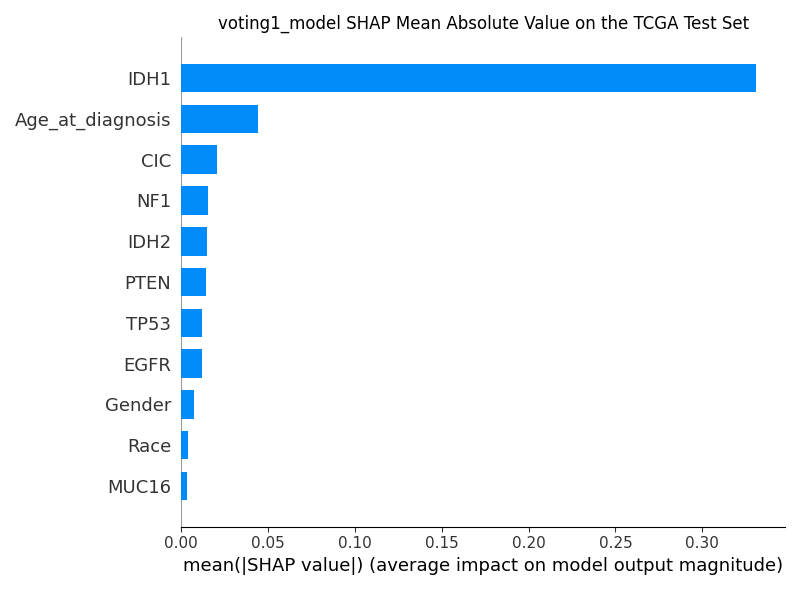

Supplement: S7 File — (ZIP) [file pone.0314831.s017.zip › S7 File/voting1_model_feature_importance_bar.png]

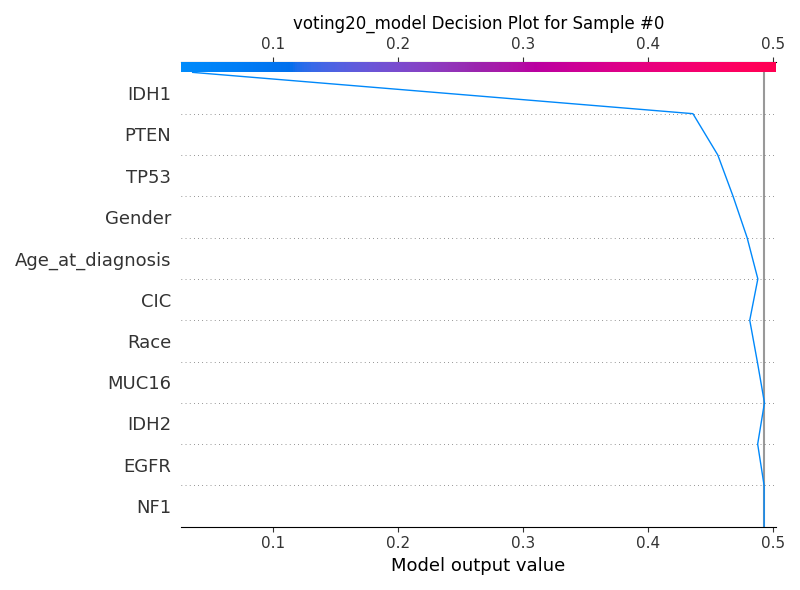

Supplement: S7 File — (ZIP) [file pone.0314831.s017.zip › S7 File/voting20_model_decision_plot.png]

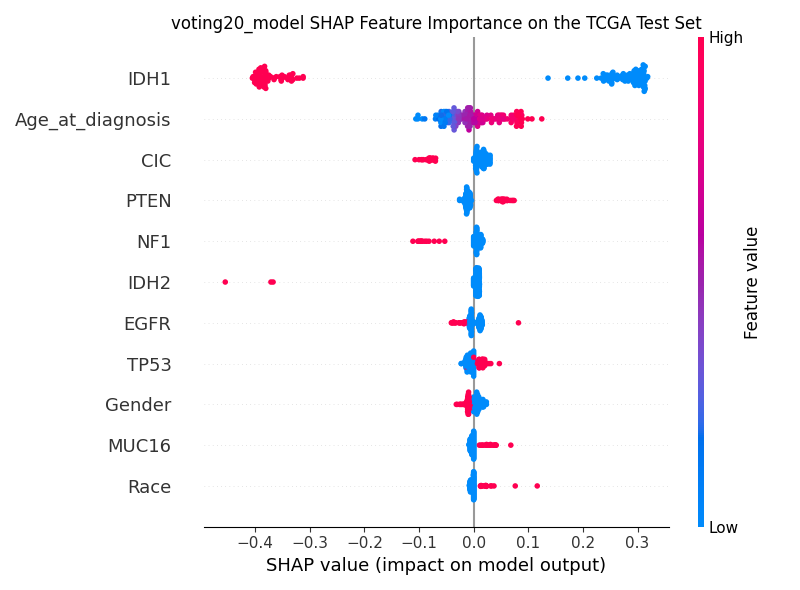

Supplement: S7 File — (ZIP) [file pone.0314831.s017.zip › S7 File/voting20_model_feature_importance.png]

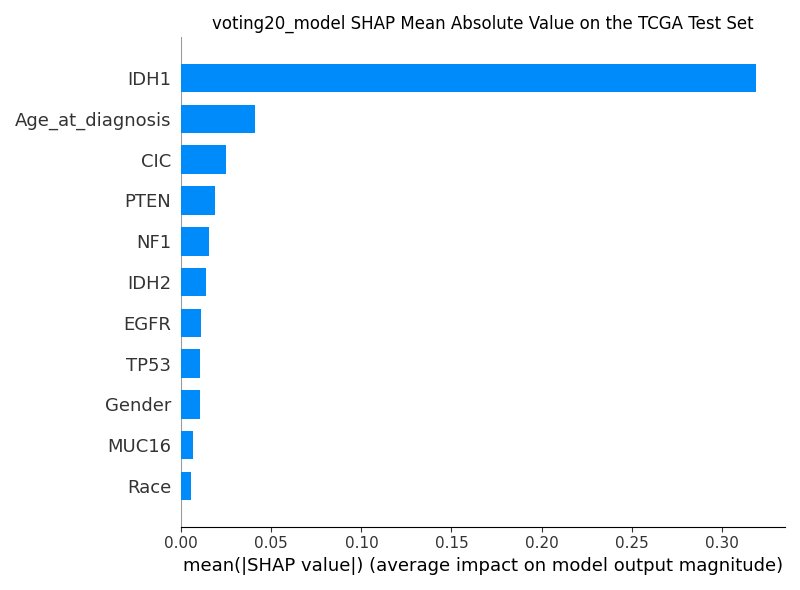

Supplement: S7 File — (ZIP) [file pone.0314831.s017.zip › S7 File/voting20_model_feature_importance_bar.png]

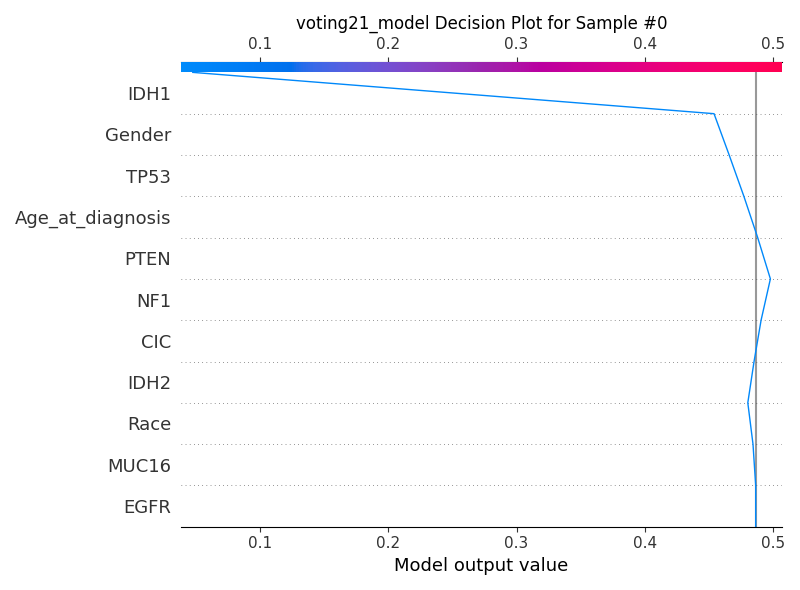

Supplement: S7 File — (ZIP) [file pone.0314831.s017.zip › S7 File/voting21_model_decision_plot.png]

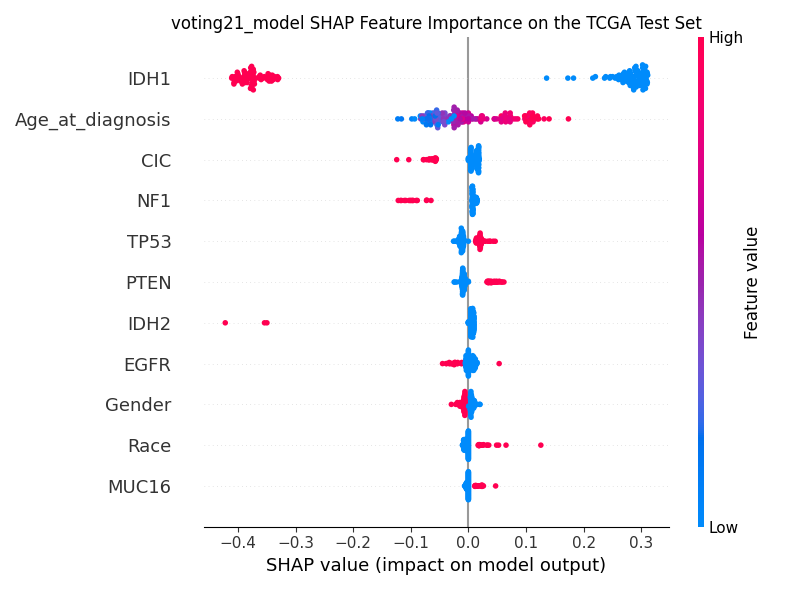

Supplement: S7 File — (ZIP) [file pone.0314831.s017.zip › S7 File/voting21_model_feature_importance.png]

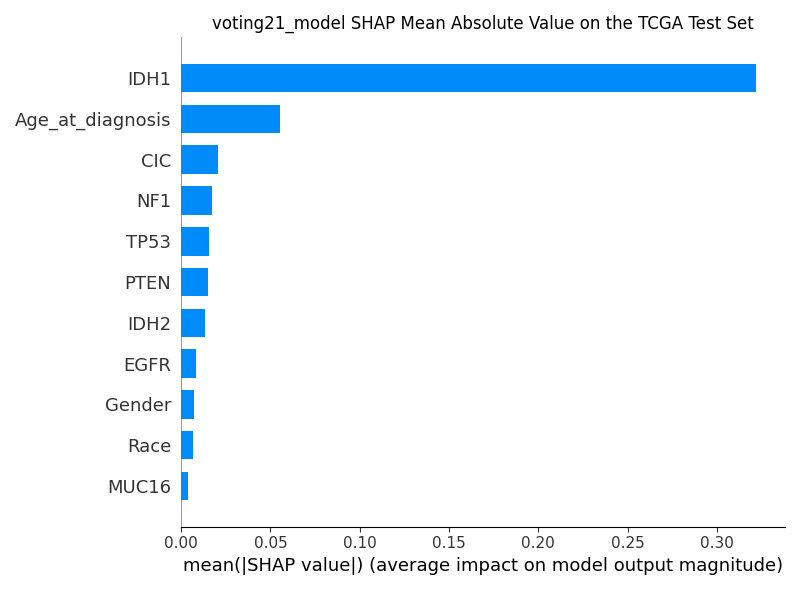

Supplement: S7 File — (ZIP) [file pone.0314831.s017.zip › S7 File/voting21_model_feature_importance_bar.png]

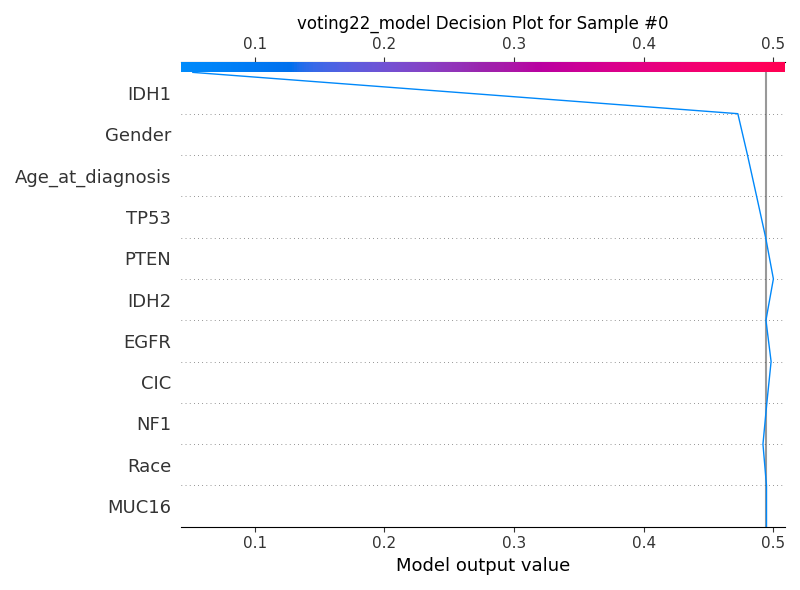

Supplement: S7 File — (ZIP) [file pone.0314831.s017.zip › S7 File/voting22_model_decision_plot.png]

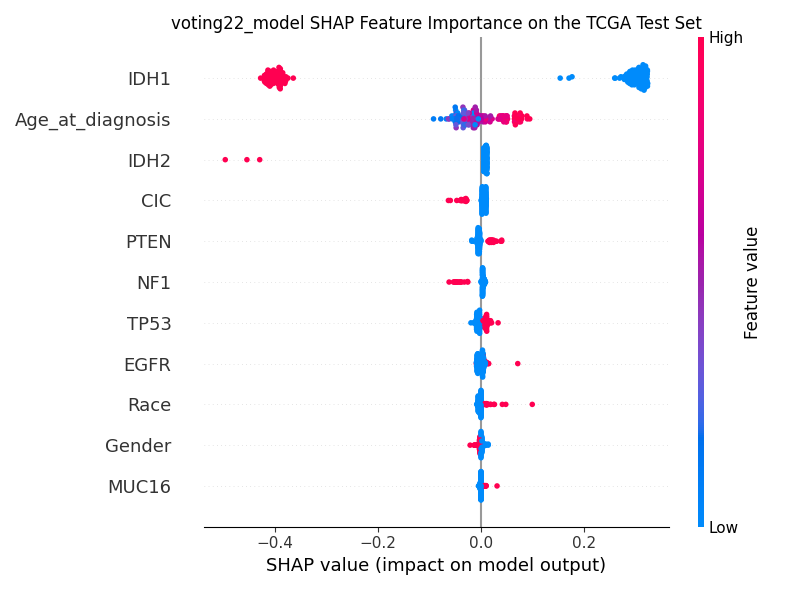

Supplement: S7 File — (ZIP) [file pone.0314831.s017.zip › S7 File/voting22_model_feature_importance.png]

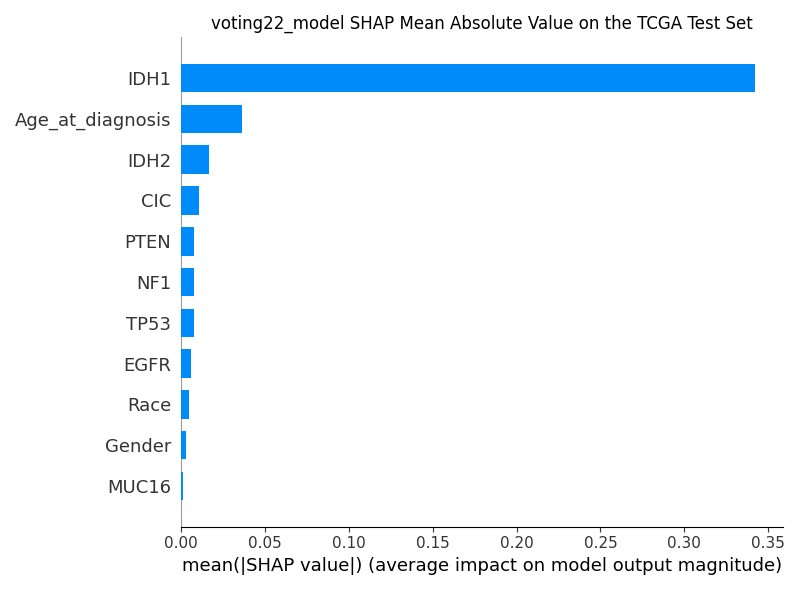

Supplement: S7 File — (ZIP) [file pone.0314831.s017.zip › S7 File/voting22_model_feature_importance_bar.png]

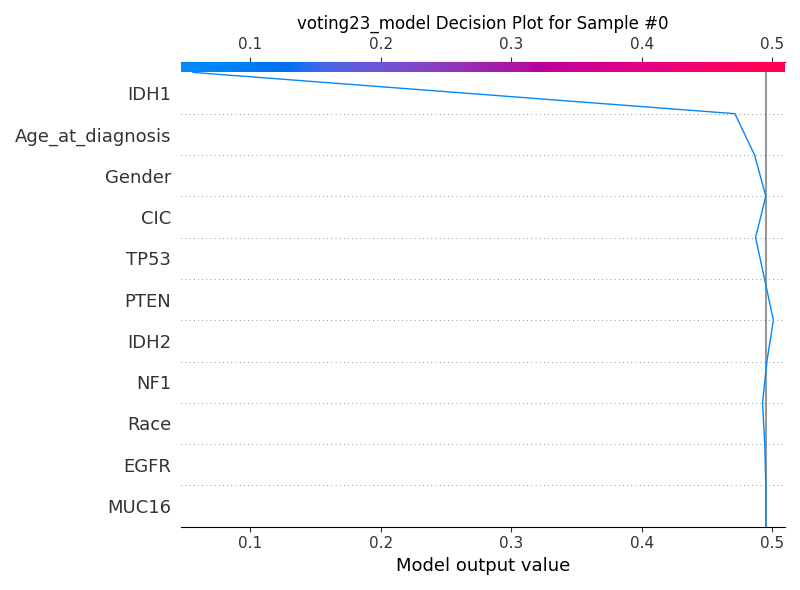

Supplement: S7 File — (ZIP) [file pone.0314831.s017.zip › S7 File/voting23_model_decision_plot.png]

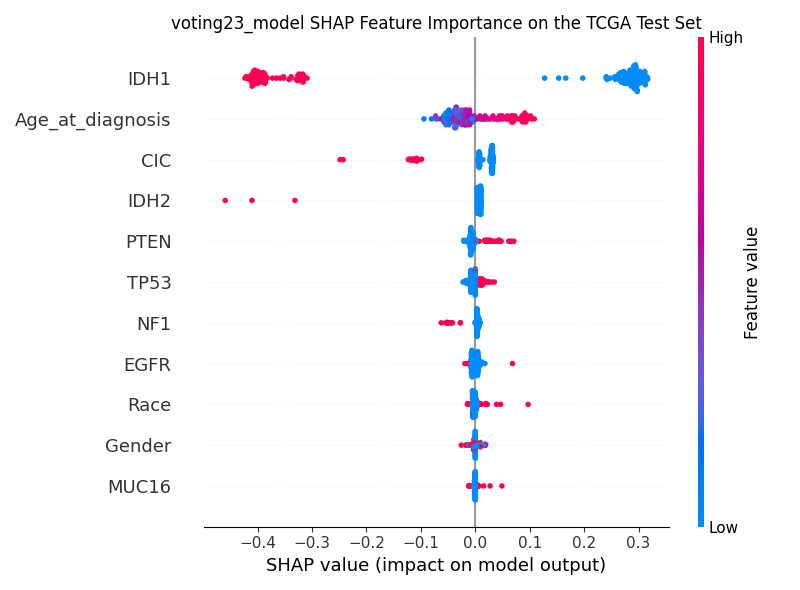

Supplement: S7 File — (ZIP) [file pone.0314831.s017.zip › S7 File/voting23_model_feature_importance.png]

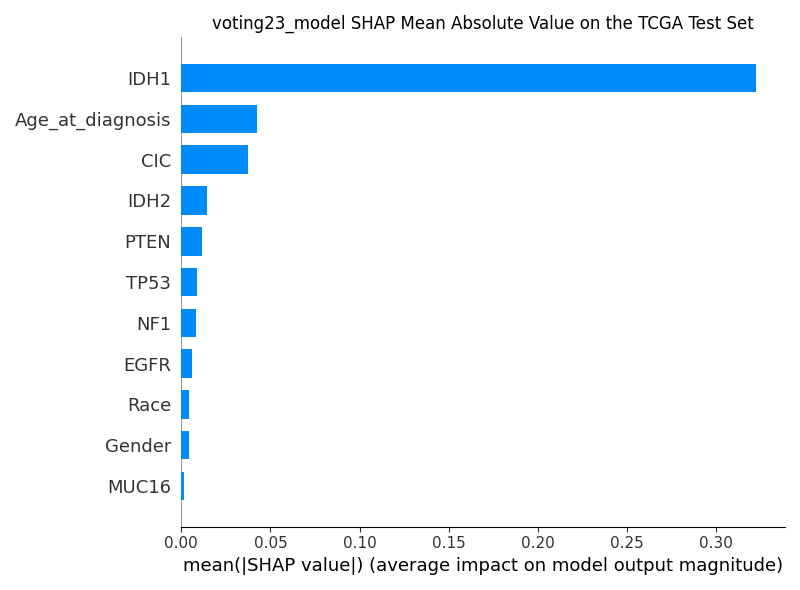

Supplement: S7 File — (ZIP) [file pone.0314831.s017.zip › S7 File/voting23_model_feature_importance_bar.png]

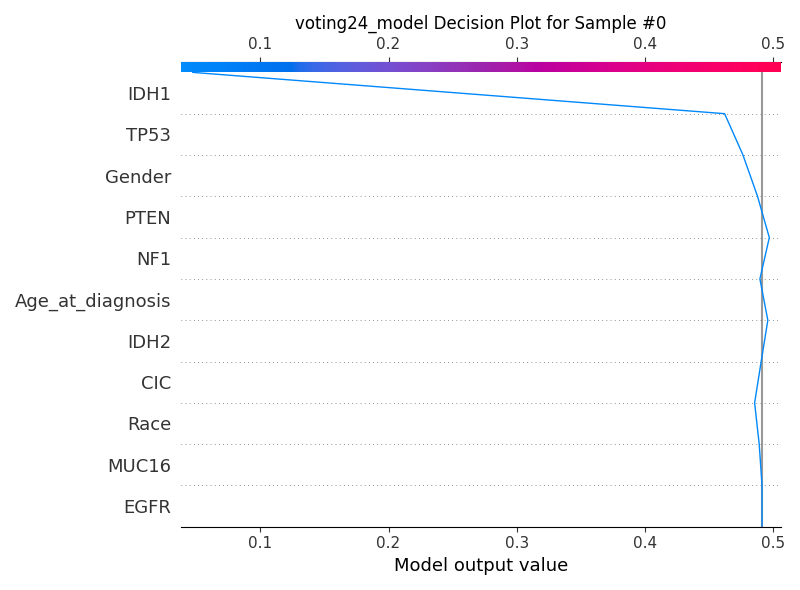

Supplement: S7 File — (ZIP) [file pone.0314831.s017.zip › S7 File/voting24_model_decision_plot.png]

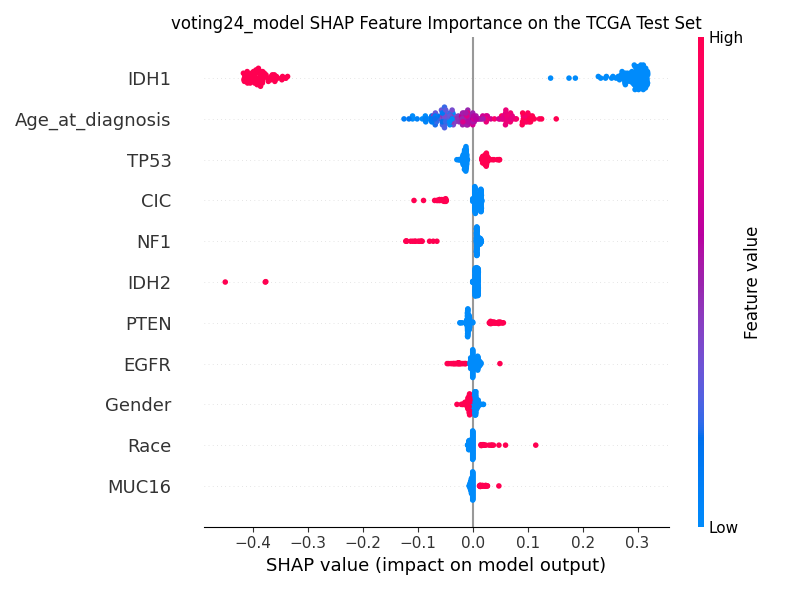

Supplement: S7 File — (ZIP) [file pone.0314831.s017.zip › S7 File/voting24_model_feature_importance.png]

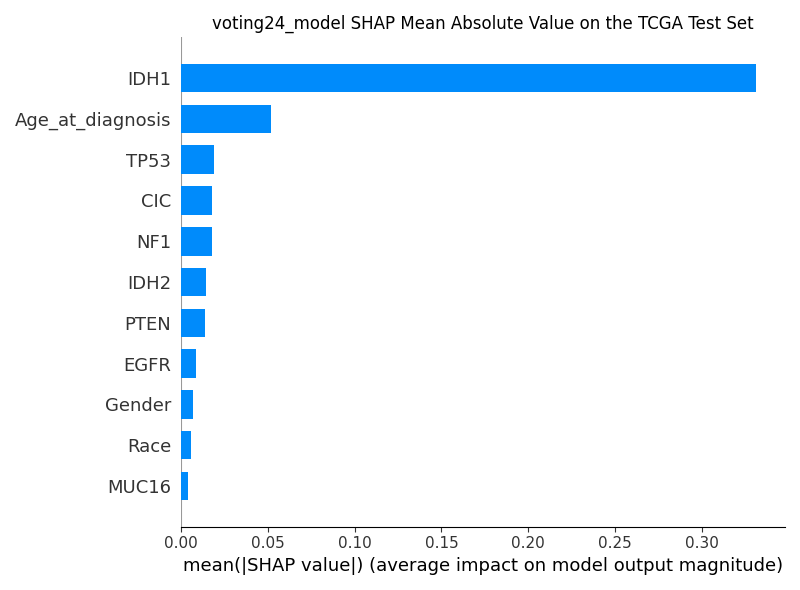

Supplement: S7 File — (ZIP) [file pone.0314831.s017.zip › S7 File/voting24_model_feature_importance_bar.png]

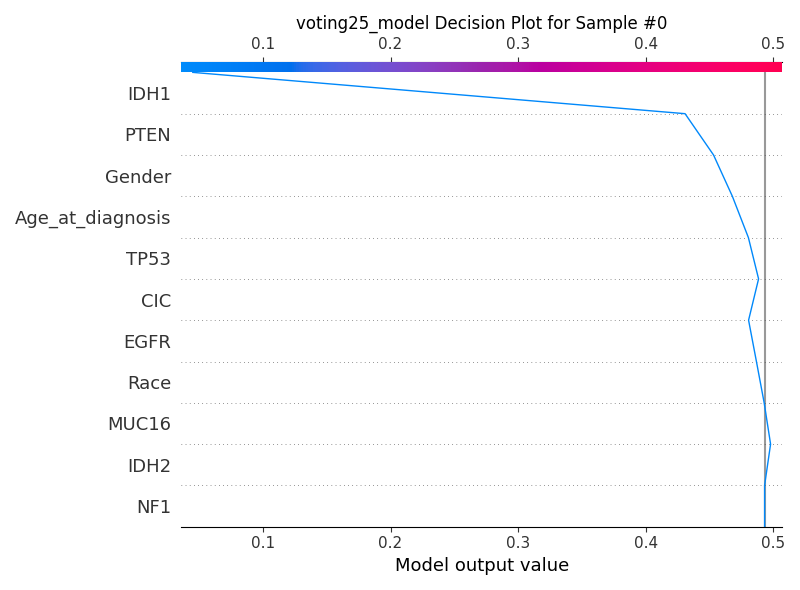

Supplement: S7 File — (ZIP) [file pone.0314831.s017.zip › S7 File/voting25_model_decision_plot.png]

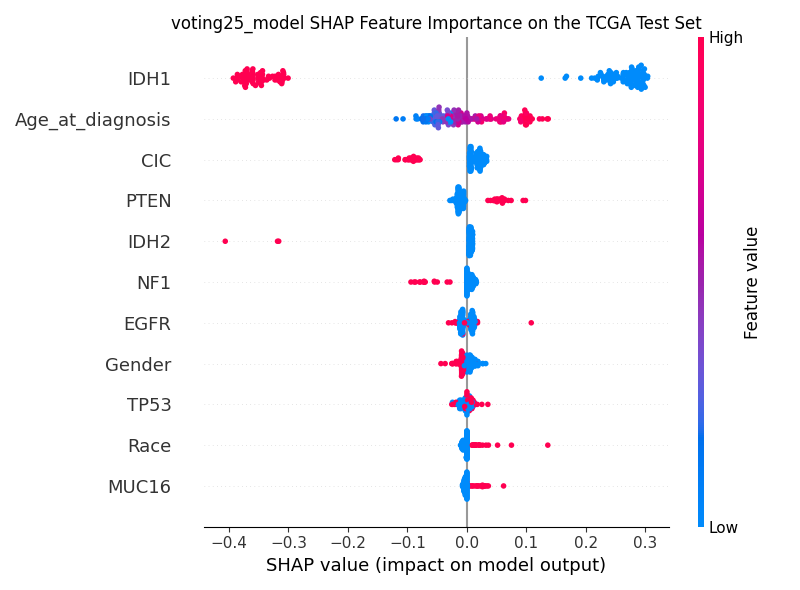

Supplement: S7 File — (ZIP) [file pone.0314831.s017.zip › S7 File/voting25_model_feature_importance.png]

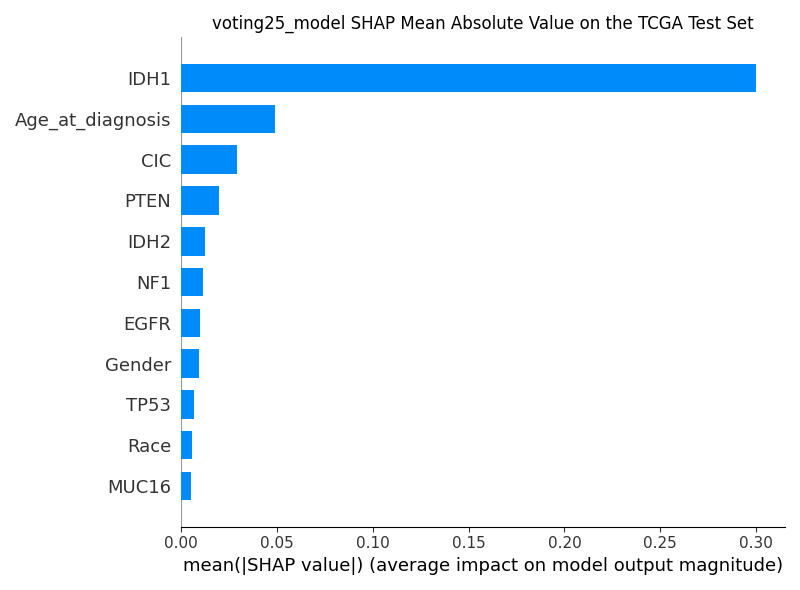

Supplement: S7 File — (ZIP) [file pone.0314831.s017.zip › S7 File/voting25_model_feature_importance_bar.png]

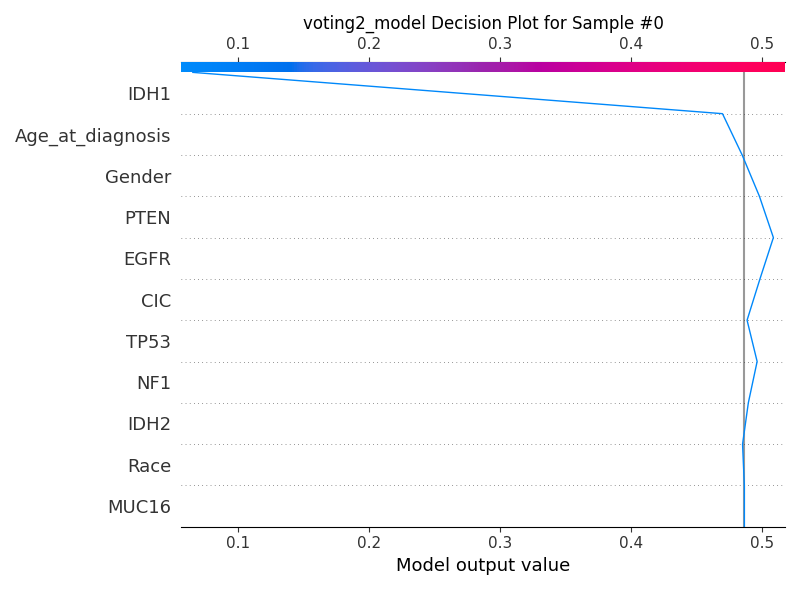

Supplement: S7 File — (ZIP) [file pone.0314831.s017.zip › S7 File/voting2_model_decision_plot.png]

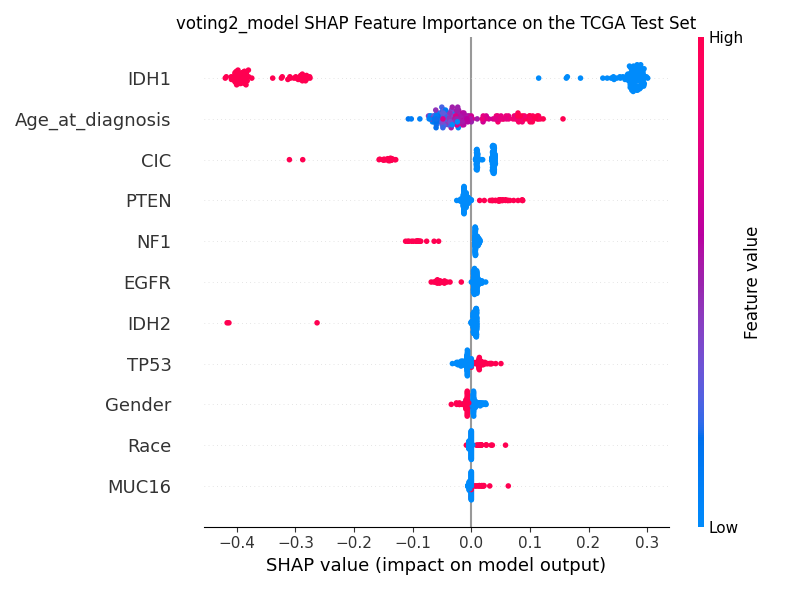

Supplement: S7 File — (ZIP) [file pone.0314831.s017.zip › S7 File/voting2_model_feature_importance.png]

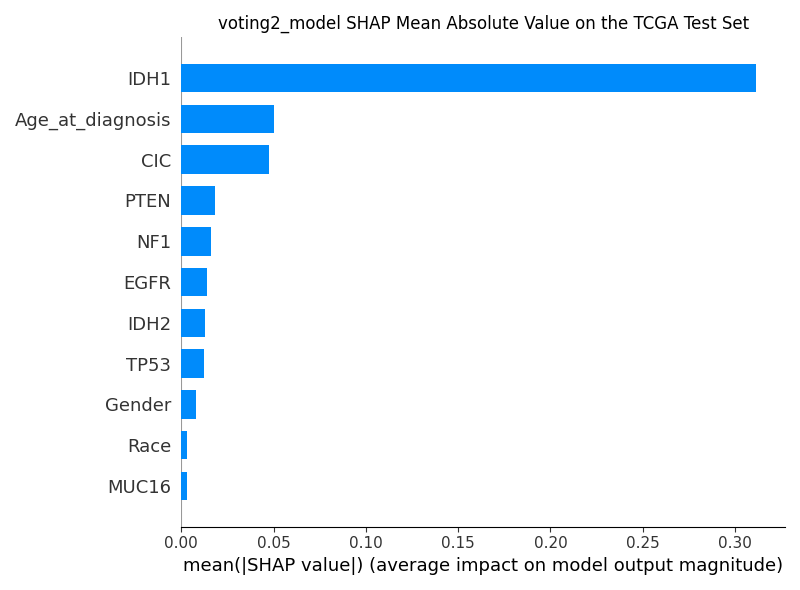

Supplement: S7 File — (ZIP) [file pone.0314831.s017.zip › S7 File/voting2_model_feature_importance_bar.png]

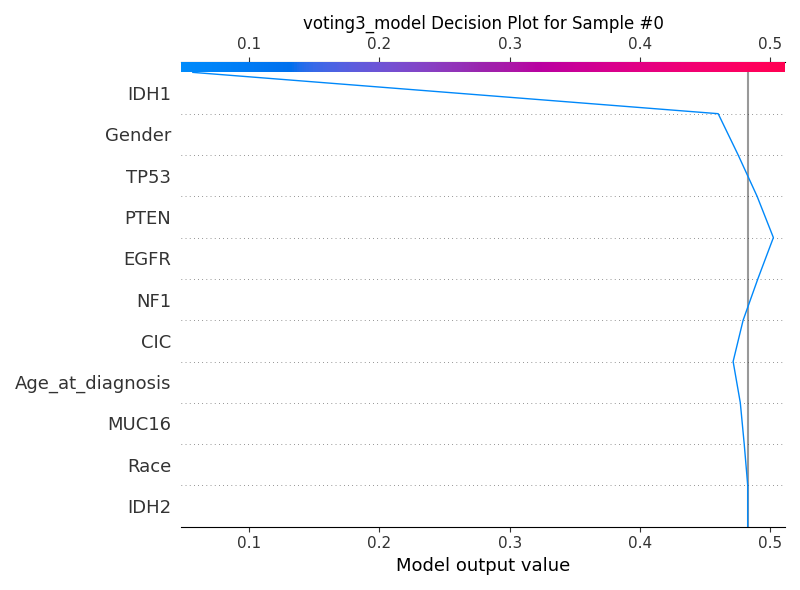

Supplement: S7 File — (ZIP) [file pone.0314831.s017.zip › S7 File/voting3_model_decision_plot.png]

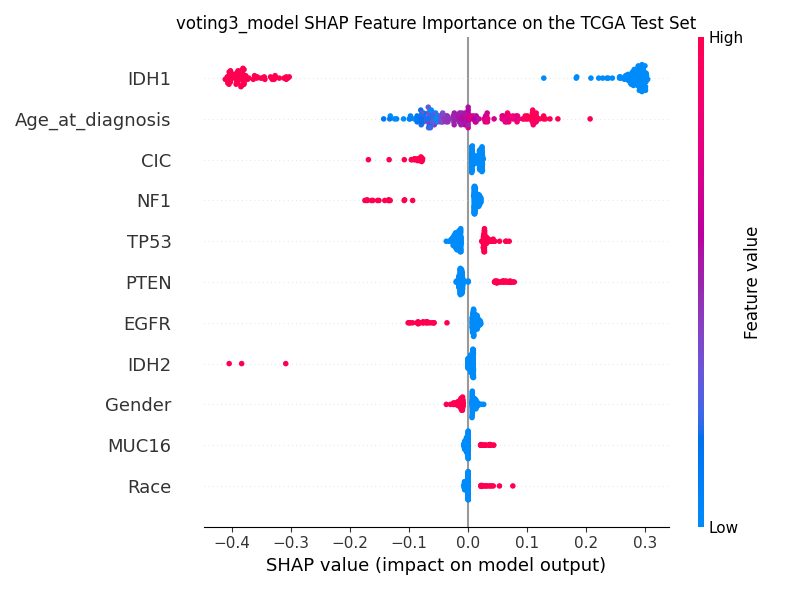

Supplement: S7 File — (ZIP) [file pone.0314831.s017.zip › S7 File/voting3_model_feature_importance.png]

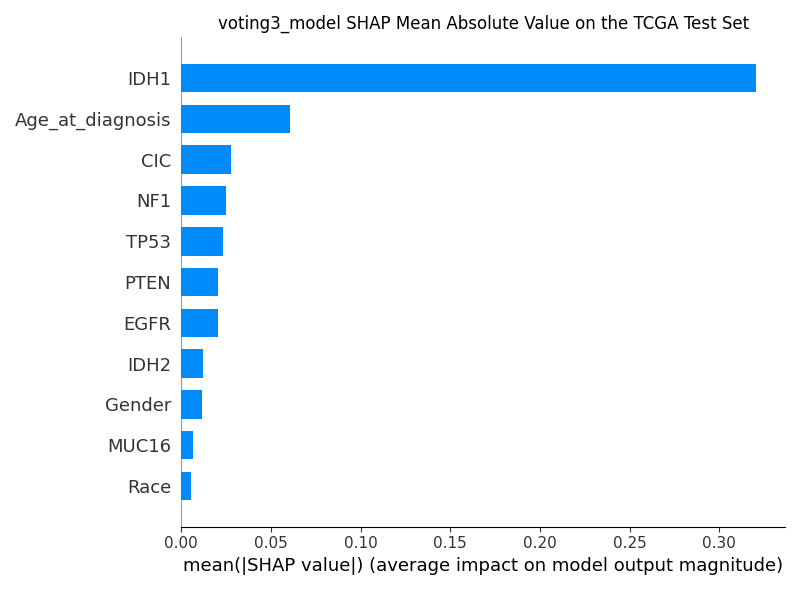

Supplement: S7 File — (ZIP) [file pone.0314831.s017.zip › S7 File/voting3_model_feature_importance_bar.png]

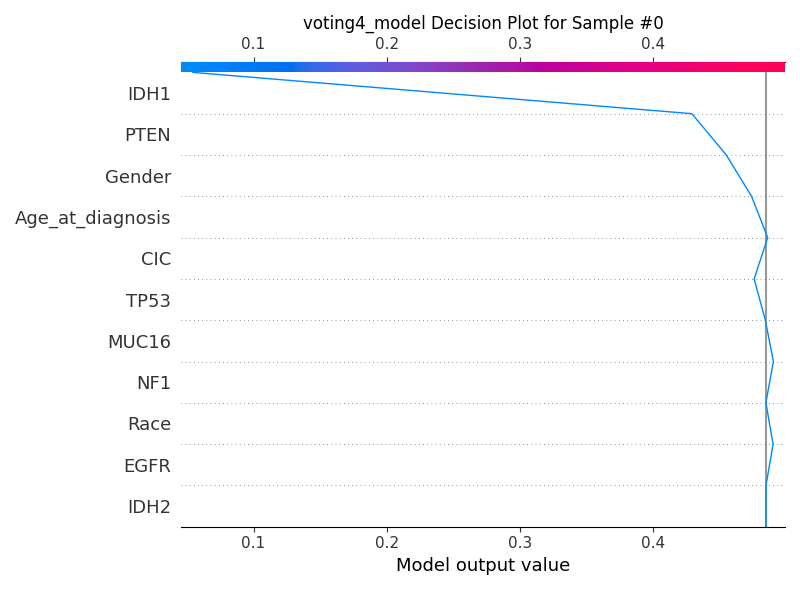

Supplement: S7 File — (ZIP) [file pone.0314831.s017.zip › S7 File/voting4_model_decision_plot.png]

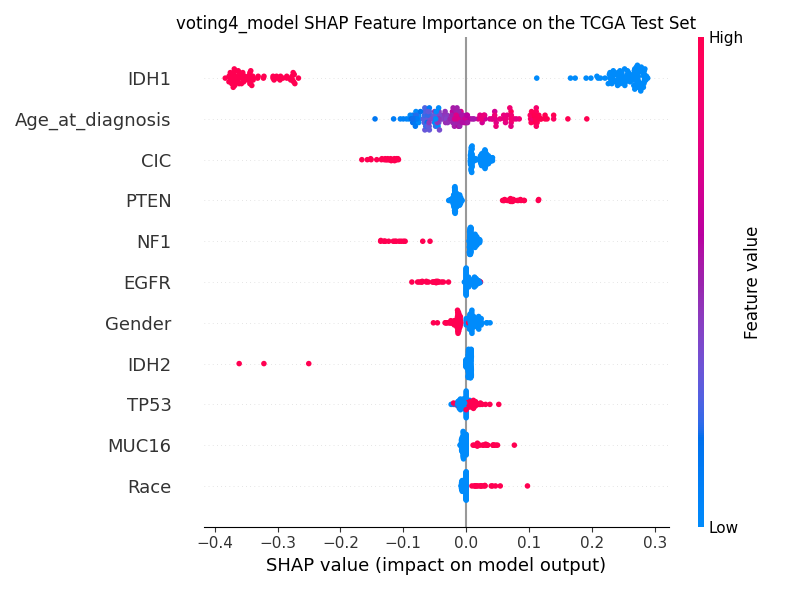

Supplement: S7 File — (ZIP) [file pone.0314831.s017.zip › S7 File/voting4_model_feature_importance.png]

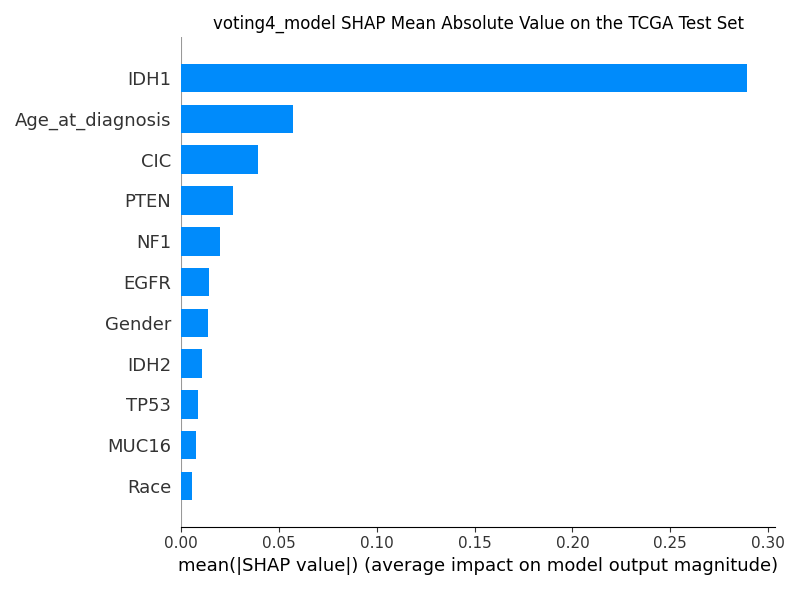

Supplement: S7 File — (ZIP) [file pone.0314831.s017.zip › S7 File/voting4_model_feature_importance_bar.png]

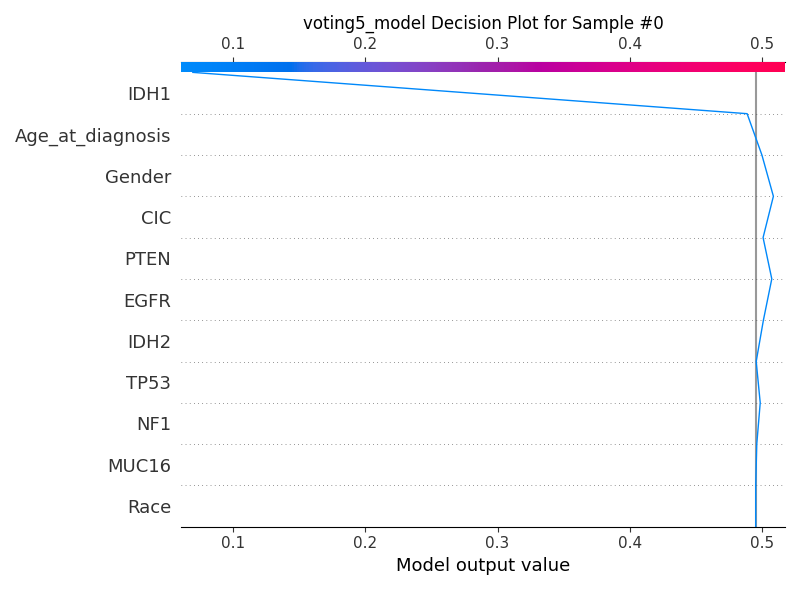

Supplement: S7 File — (ZIP) [file pone.0314831.s017.zip › S7 File/voting5_model_decision_plot.png]

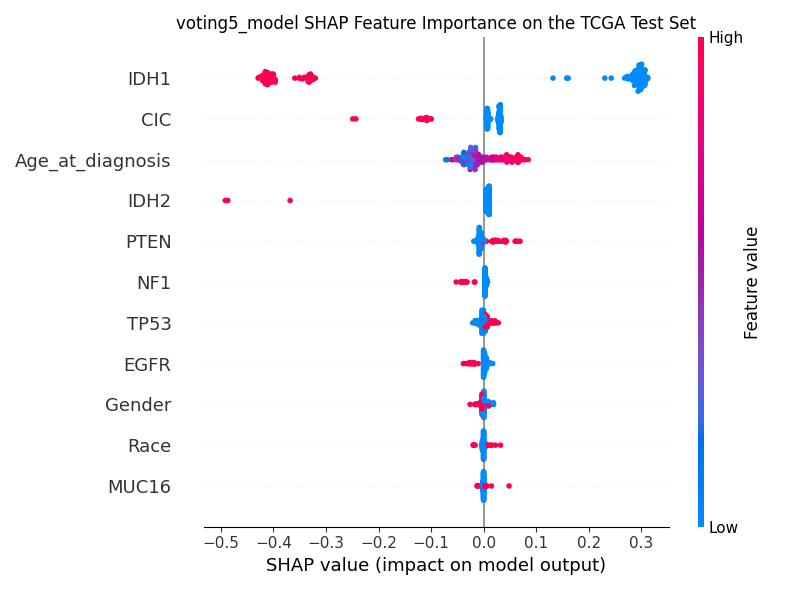

Supplement: S7 File — (ZIP) [file pone.0314831.s017.zip › S7 File/voting5_model_feature_importance.png]

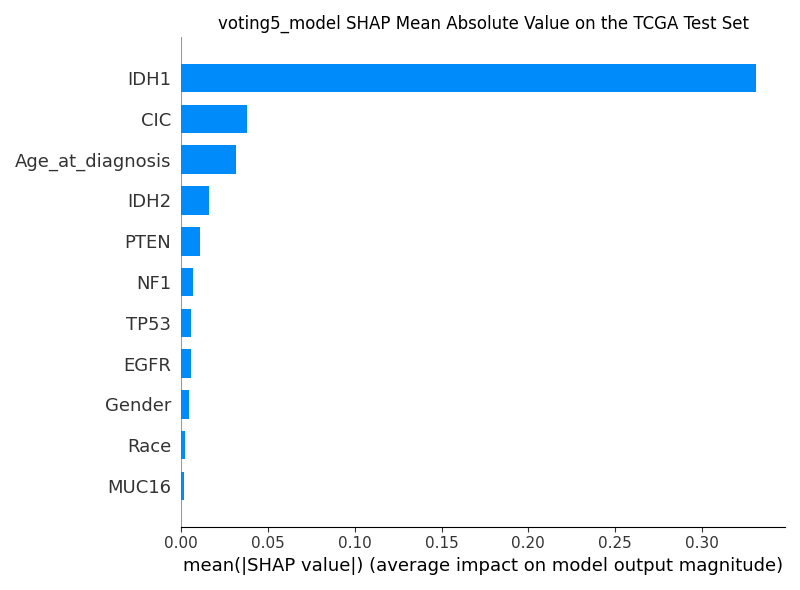

Supplement: S7 File — (ZIP) [file pone.0314831.s017.zip › S7 File/voting5_model_feature_importance_bar.png]

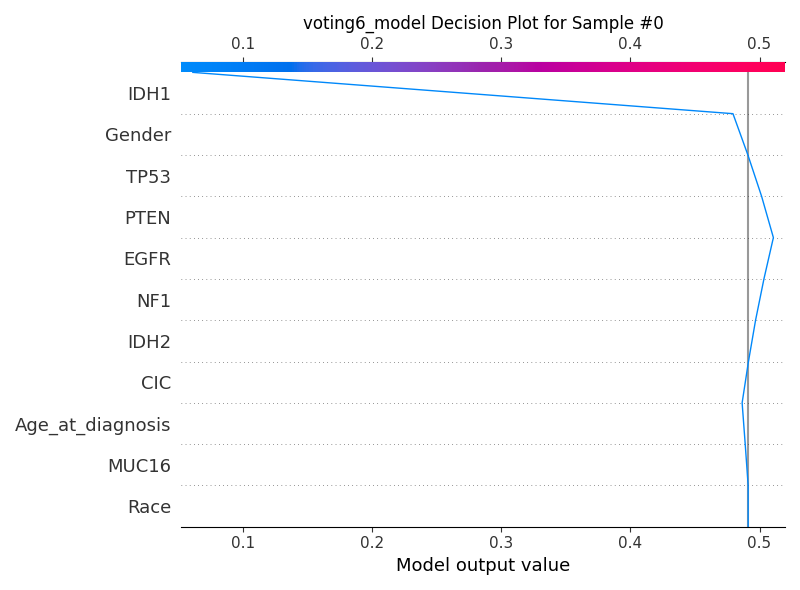

Supplement: S7 File — (ZIP) [file pone.0314831.s017.zip › S7 File/voting6_model_decision_plot.png]

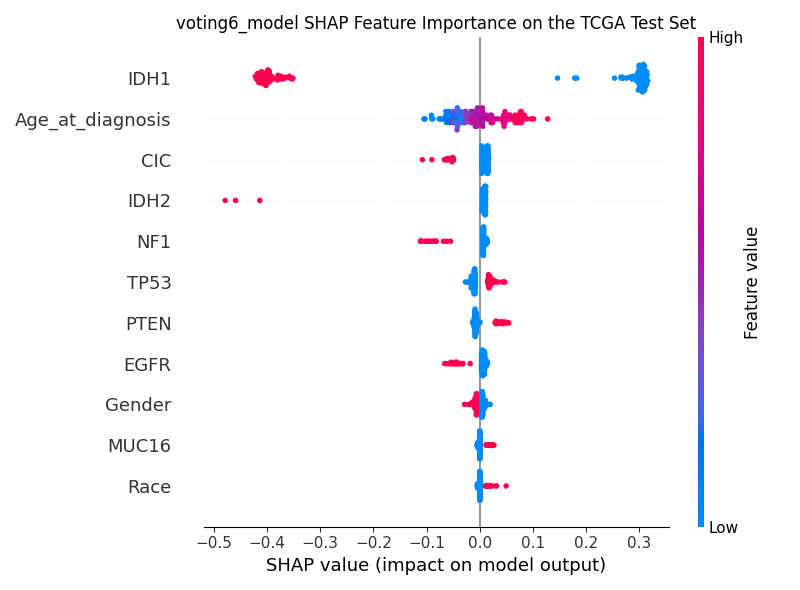

Supplement: S7 File — (ZIP) [file pone.0314831.s017.zip › S7 File/voting6_model_feature_importance.png]

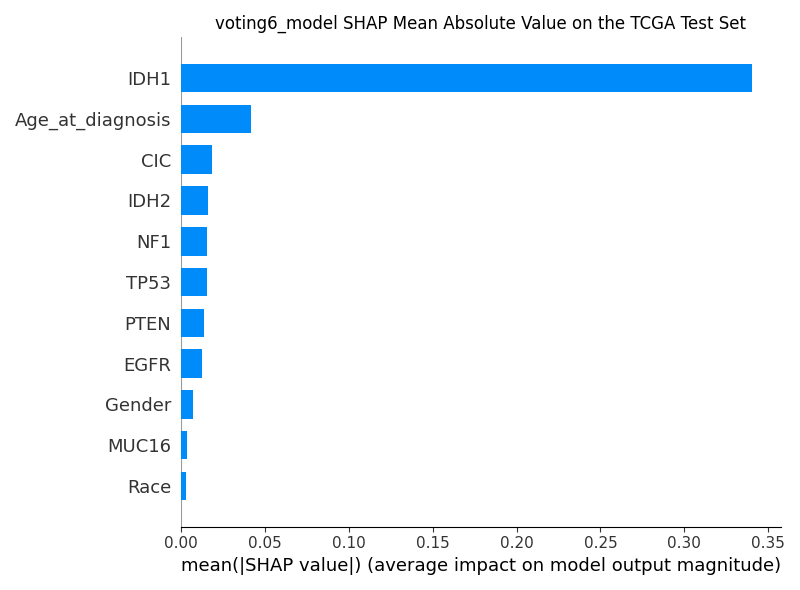

Supplement: S7 File — (ZIP) [file pone.0314831.s017.zip › S7 File/voting6_model_feature_importance_bar.png]

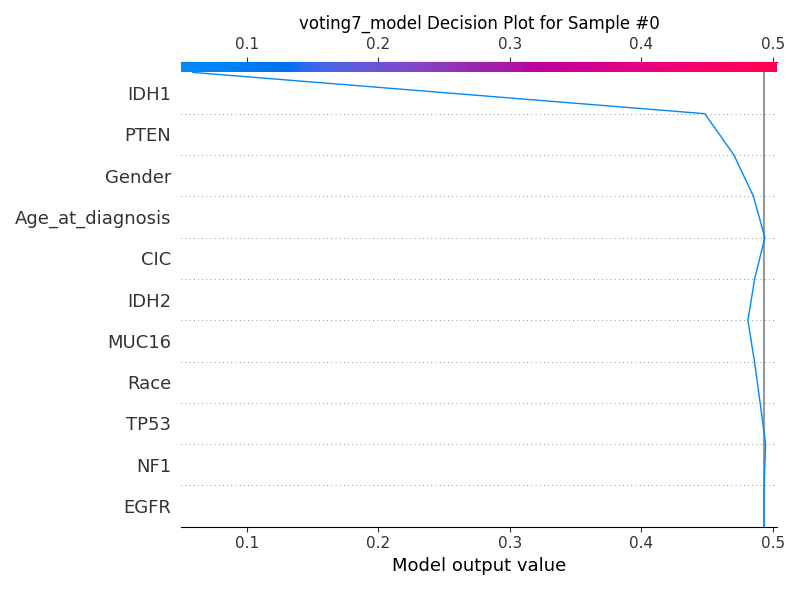

Supplement: S7 File — (ZIP) [file pone.0314831.s017.zip › S7 File/voting7_model_decision_plot.png]

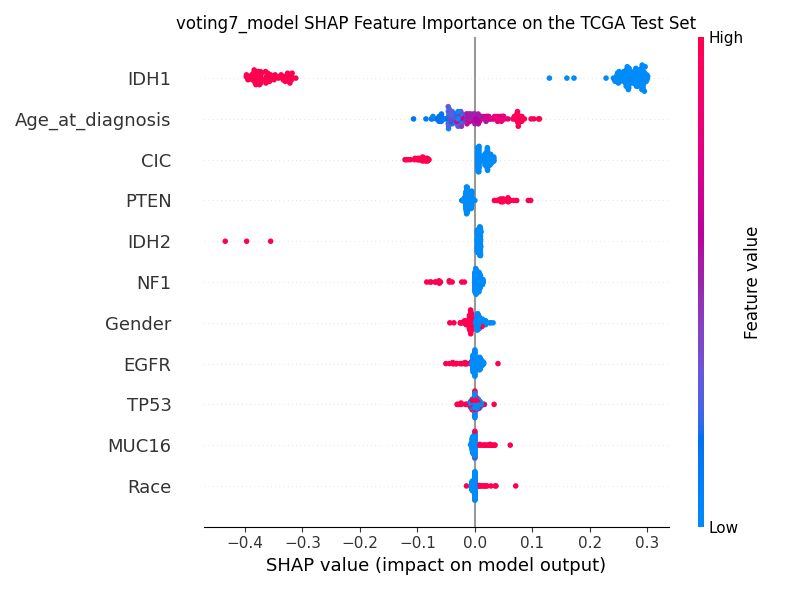

Supplement: S7 File — (ZIP) [file pone.0314831.s017.zip › S7 File/voting7_model_feature_importance.png]

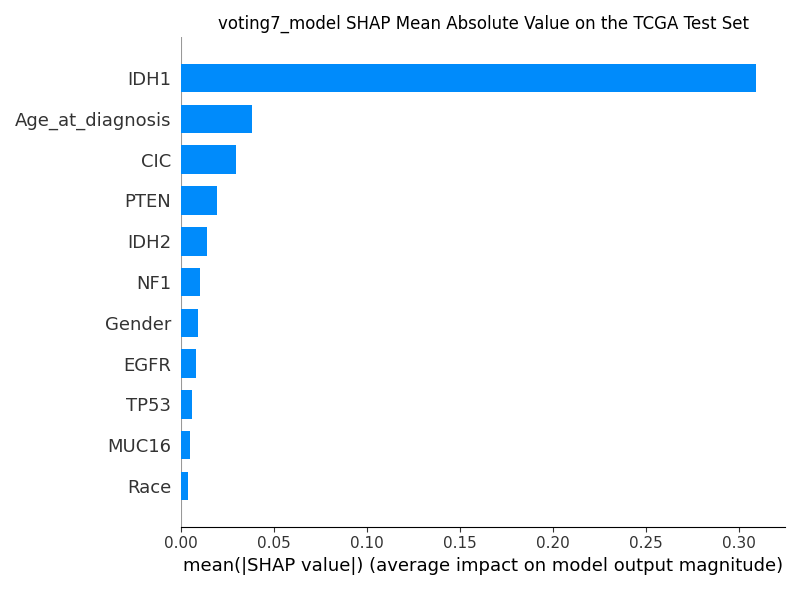

Supplement: S7 File — (ZIP) [file pone.0314831.s017.zip › S7 File/voting7_model_feature_importance_bar.png]

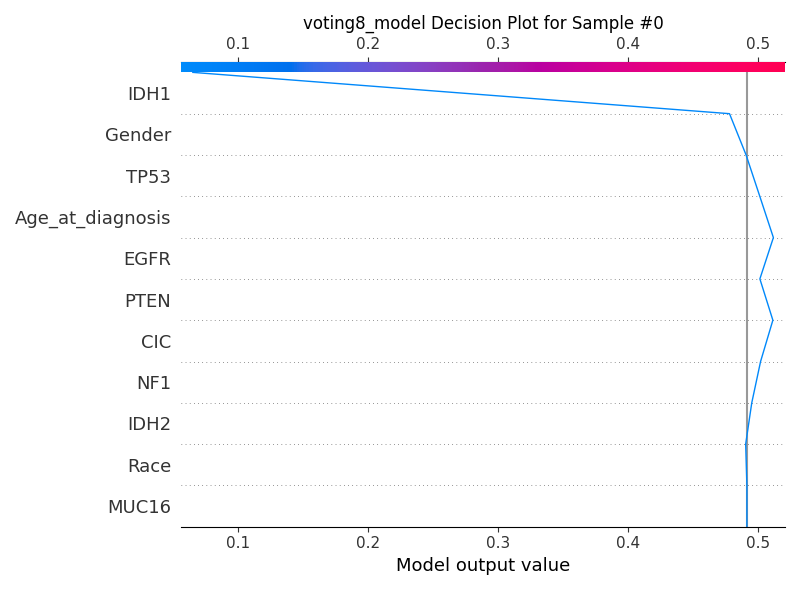

Supplement: S7 File — (ZIP) [file pone.0314831.s017.zip › S7 File/voting8_model_decision_plot.png]

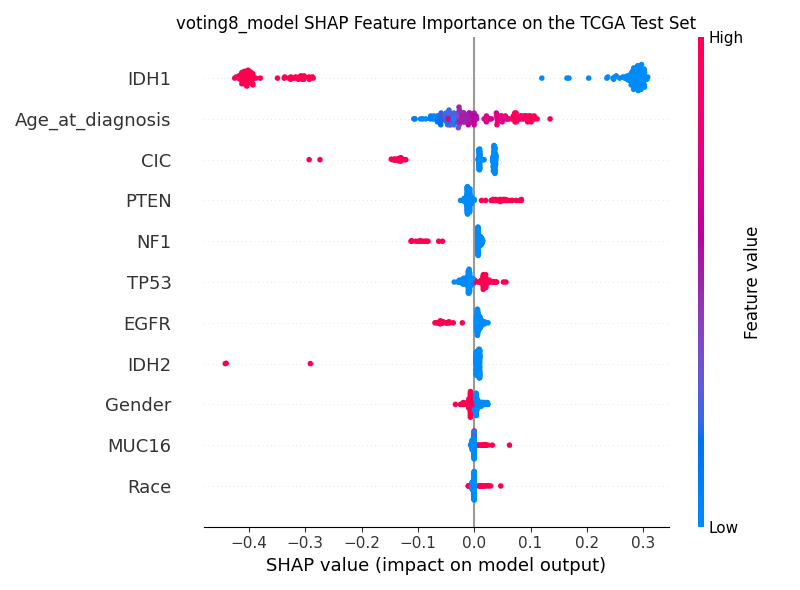

Supplement: S7 File — (ZIP) [file pone.0314831.s017.zip › S7 File/voting8_model_feature_importance.png]

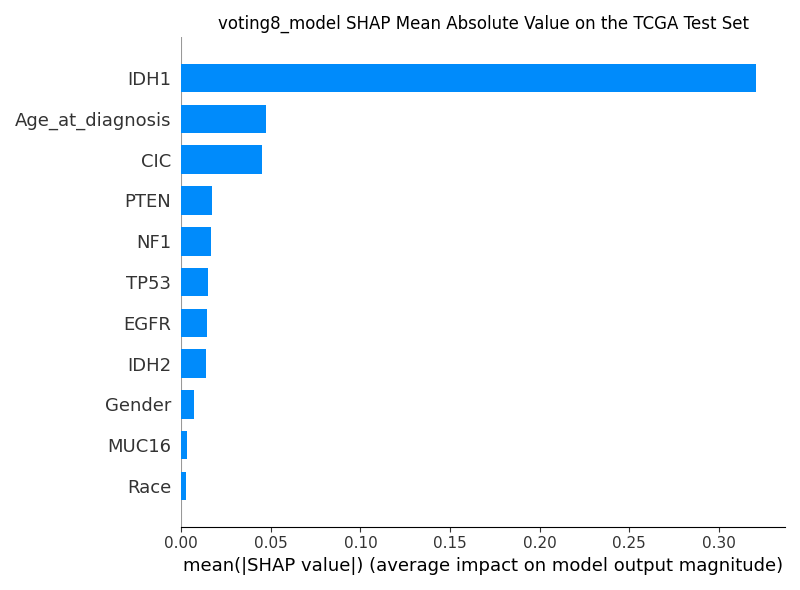

Supplement: S7 File — (ZIP) [file pone.0314831.s017.zip › S7 File/voting8_model_feature_importance_bar.png]

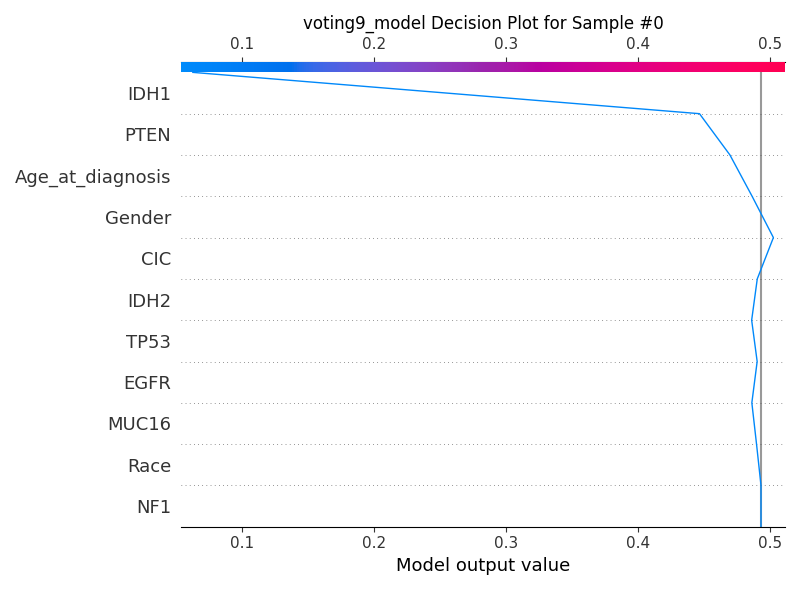

Supplement: S7 File — (ZIP) [file pone.0314831.s017.zip › S7 File/voting9_model_decision_plot.png]

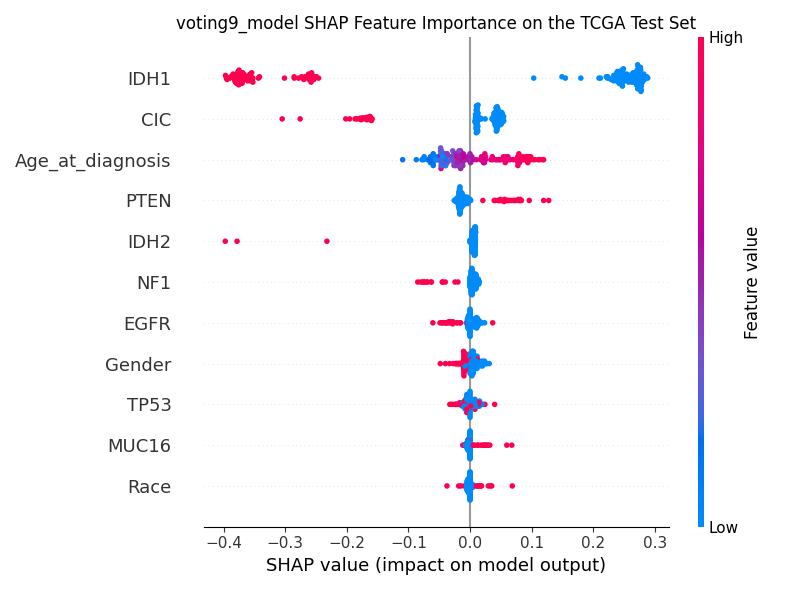

Supplement: S7 File — (ZIP) [file pone.0314831.s017.zip › S7 File/voting9_model_feature_importance.png]

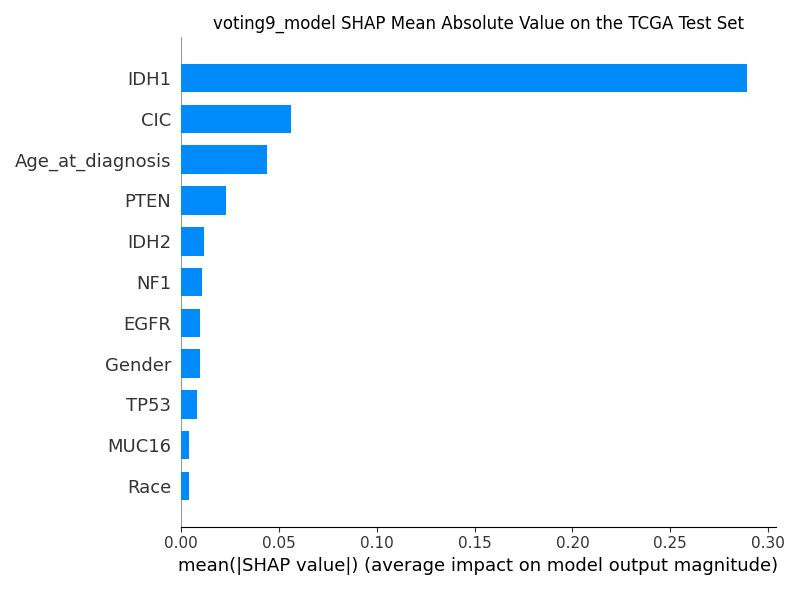

Supplement: S7 File — (ZIP) [file pone.0314831.s017.zip › S7 File/voting9_model_feature_importance_bar.png]

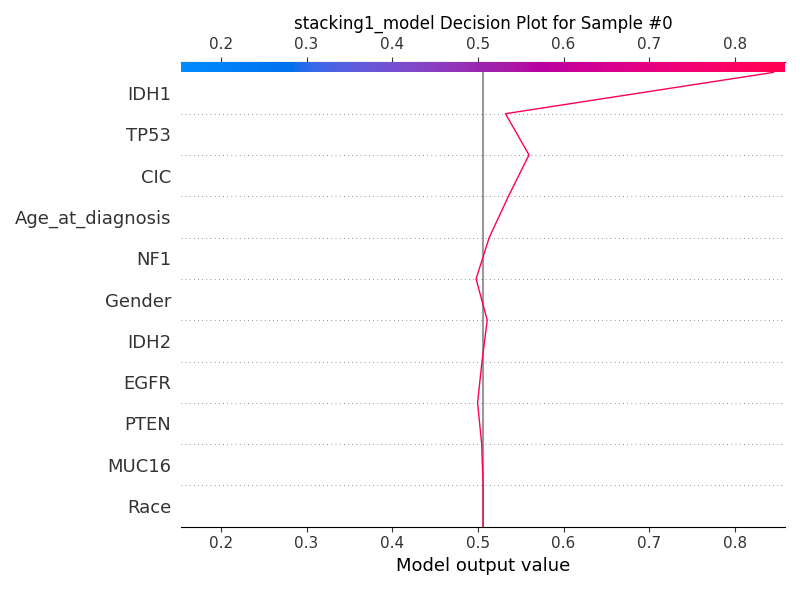

Supplement: S8 File — (ZIP) [file pone.0314831.s018.zip › S8 File/stacking1_model_decision_plot.png]

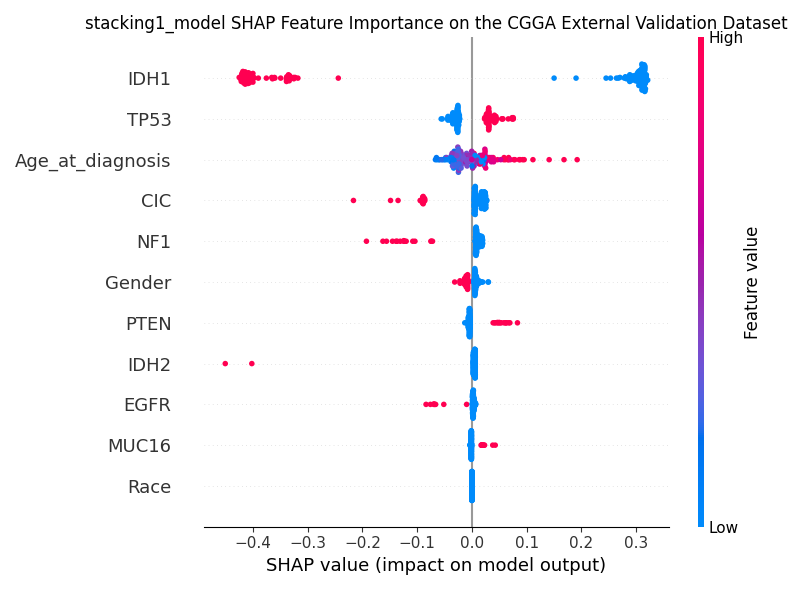

Supplement: S8 File — (ZIP) [file pone.0314831.s018.zip › S8 File/stacking1_model_feature_importance.png]

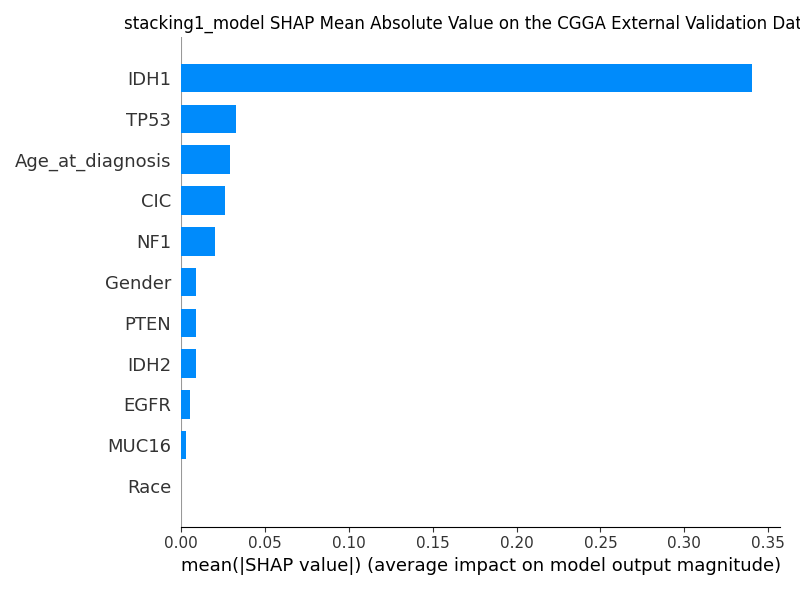

Supplement: S8 File — (ZIP) [file pone.0314831.s018.zip › S8 File/stacking1_model_feature_importance_bar.png]

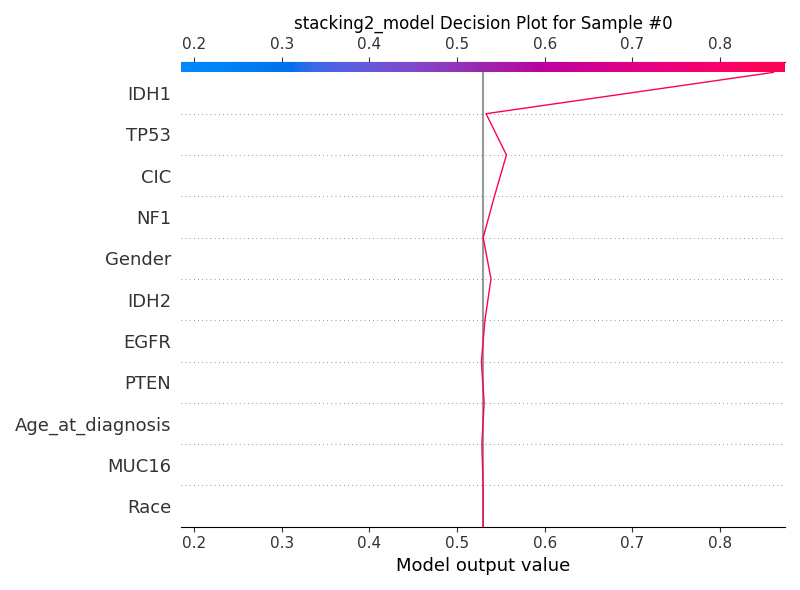

Supplement: S8 File — (ZIP) [file pone.0314831.s018.zip › S8 File/stacking2_model_decision_plot.png]

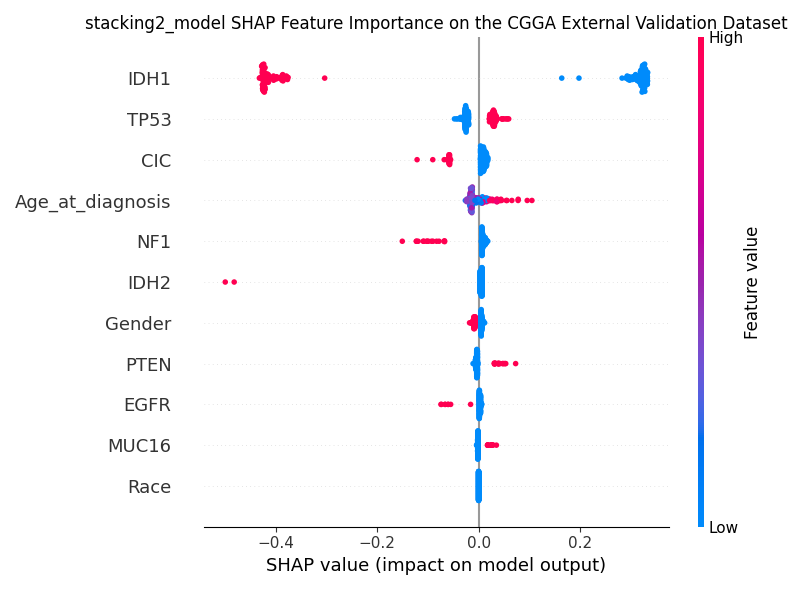

Supplement: S8 File — (ZIP) [file pone.0314831.s018.zip › S8 File/stacking2_model_feature_importance.png]

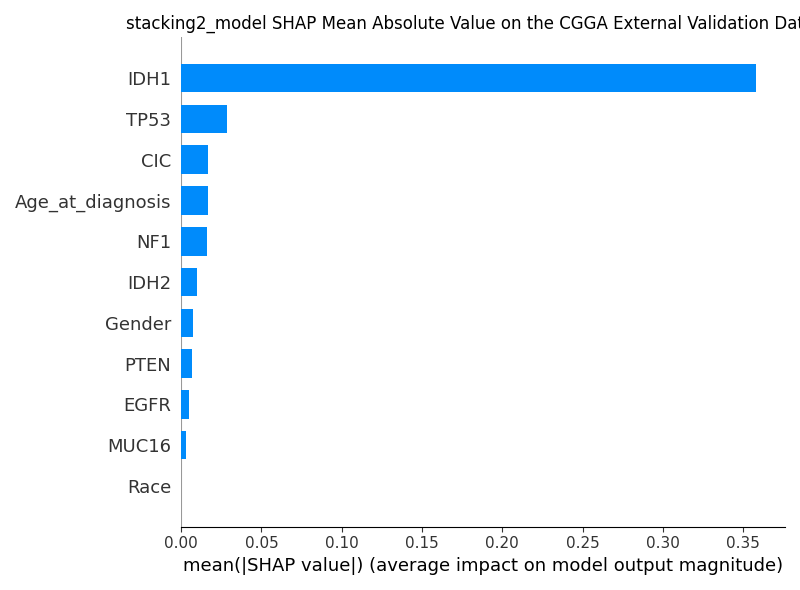

Supplement: S8 File — (ZIP) [file pone.0314831.s018.zip › S8 File/stacking2_model_feature_importance_bar.png]

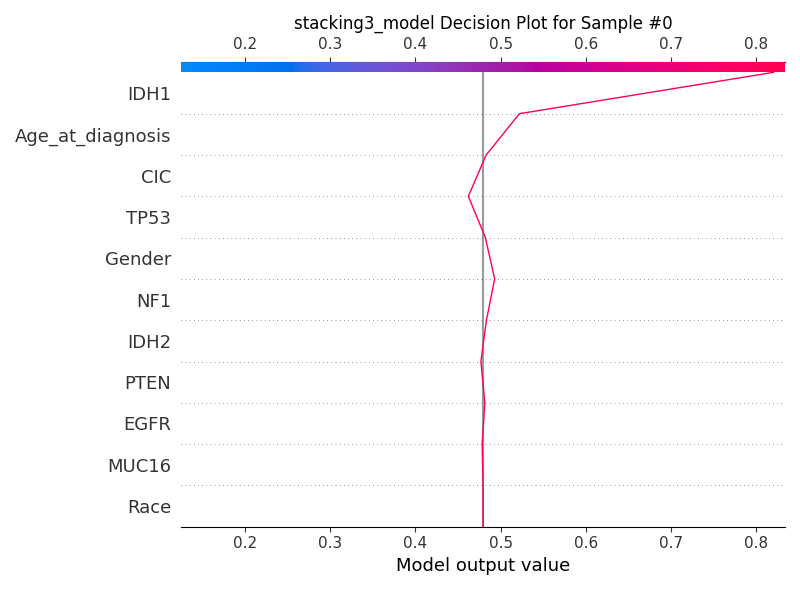

Supplement: S8 File — (ZIP) [file pone.0314831.s018.zip › S8 File/stacking3_model_decision_plot.png]

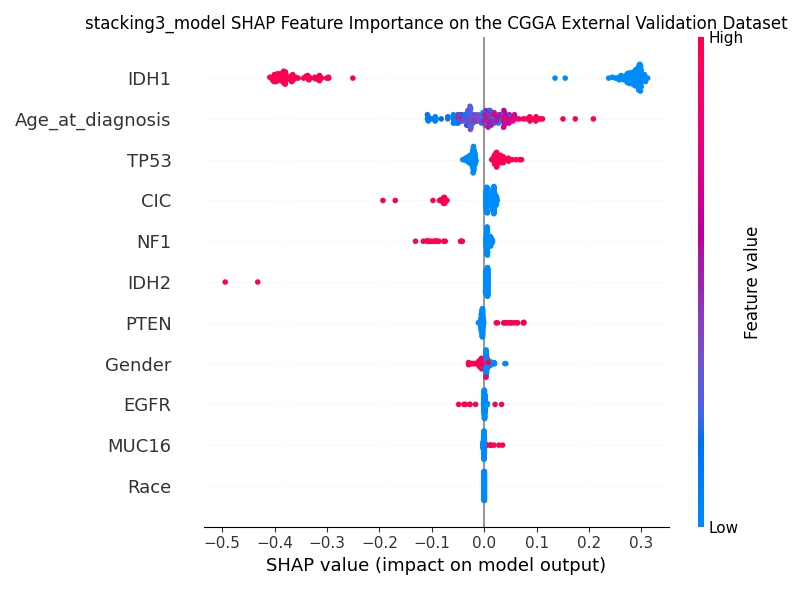

Supplement: S8 File — (ZIP) [file pone.0314831.s018.zip › S8 File/stacking3_model_feature_importance.png]

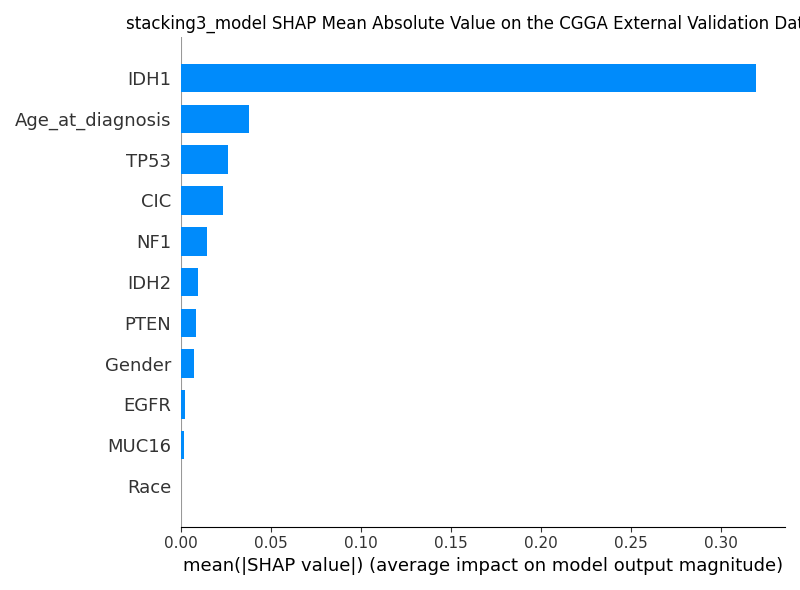

Supplement: S8 File — (ZIP) [file pone.0314831.s018.zip › S8 File/stacking3_model_feature_importance_bar.png]

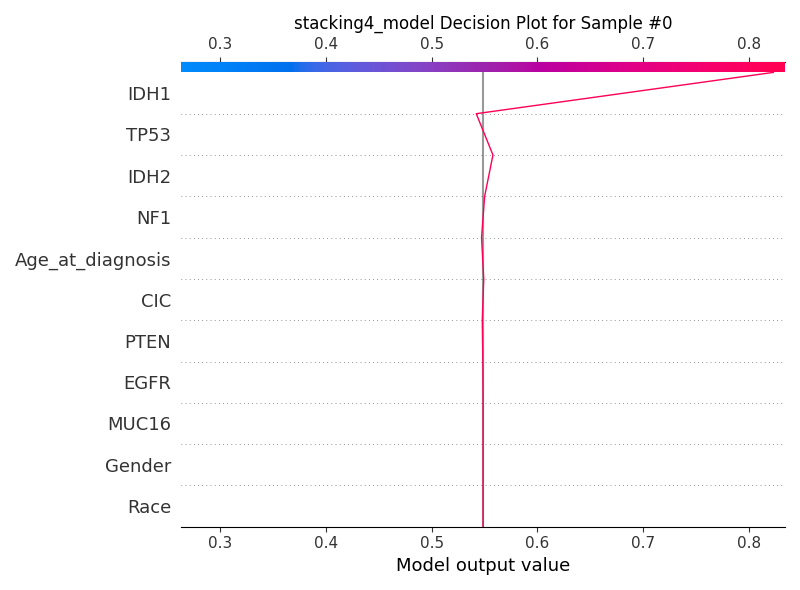

Supplement: S8 File — (ZIP) [file pone.0314831.s018.zip › S8 File/stacking4_model_decision_plot.png]

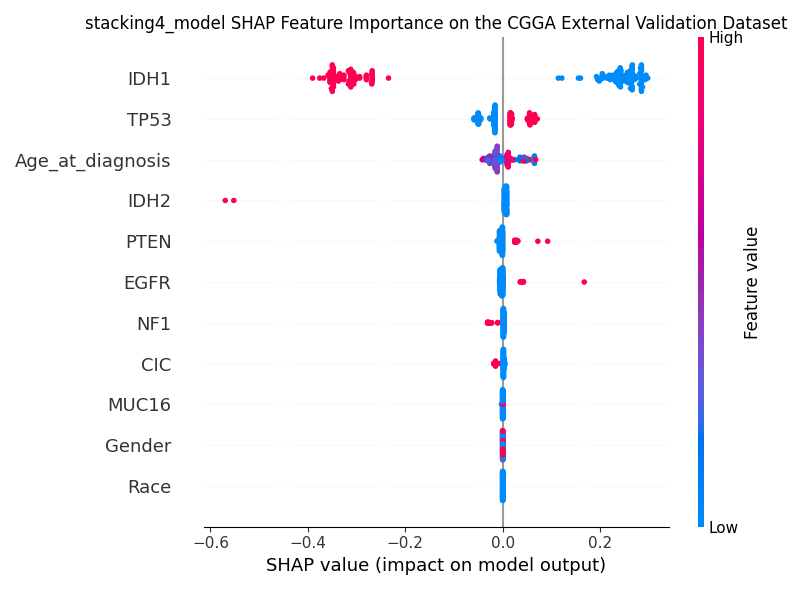

Supplement: S8 File — (ZIP) [file pone.0314831.s018.zip › S8 File/stacking4_model_feature_importance.png]

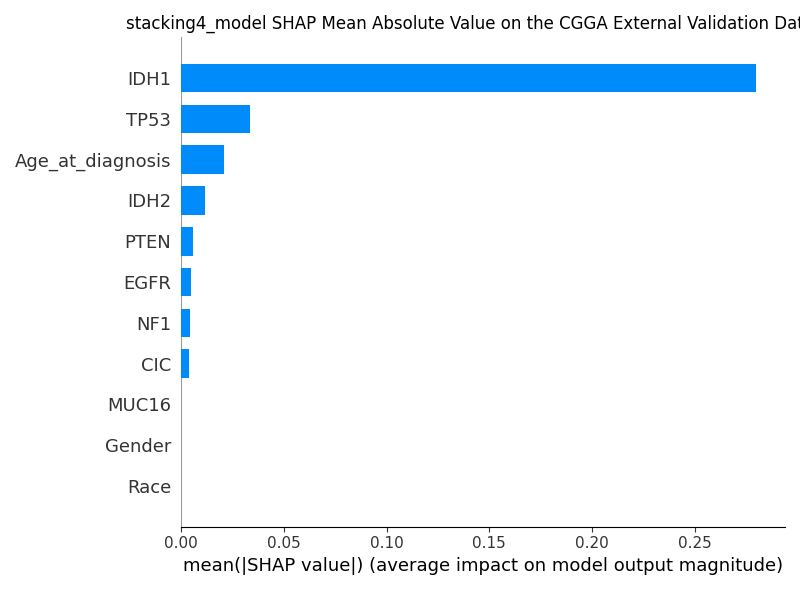

Supplement: S8 File — (ZIP) [file pone.0314831.s018.zip › S8 File/stacking4_model_feature_importance_bar.png]

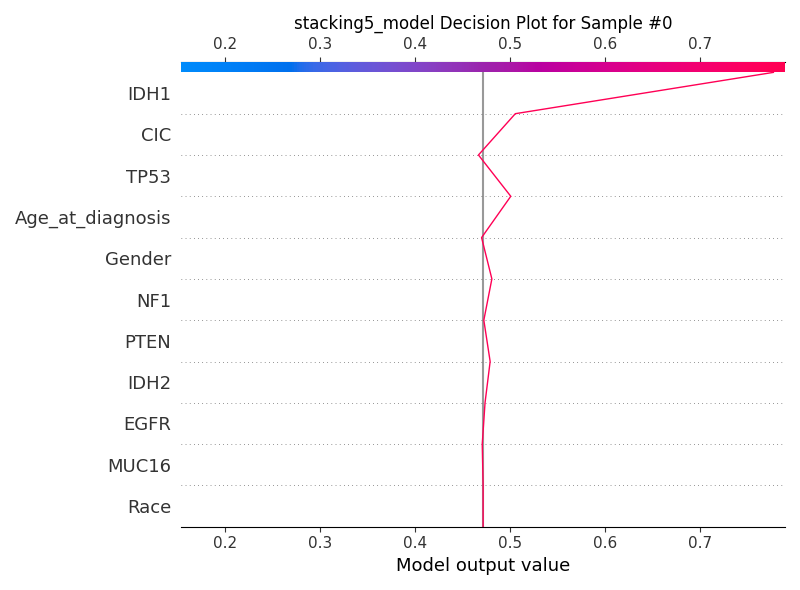

Supplement: S8 File — (ZIP) [file pone.0314831.s018.zip › S8 File/stacking5_model_decision_plot.png]

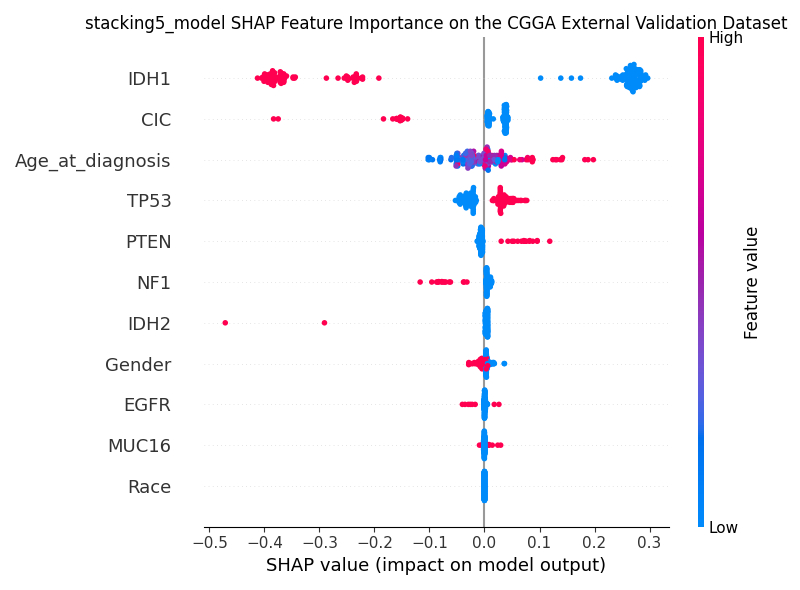

Supplement: S8 File — (ZIP) [file pone.0314831.s018.zip › S8 File/stacking5_model_feature_importance.png]

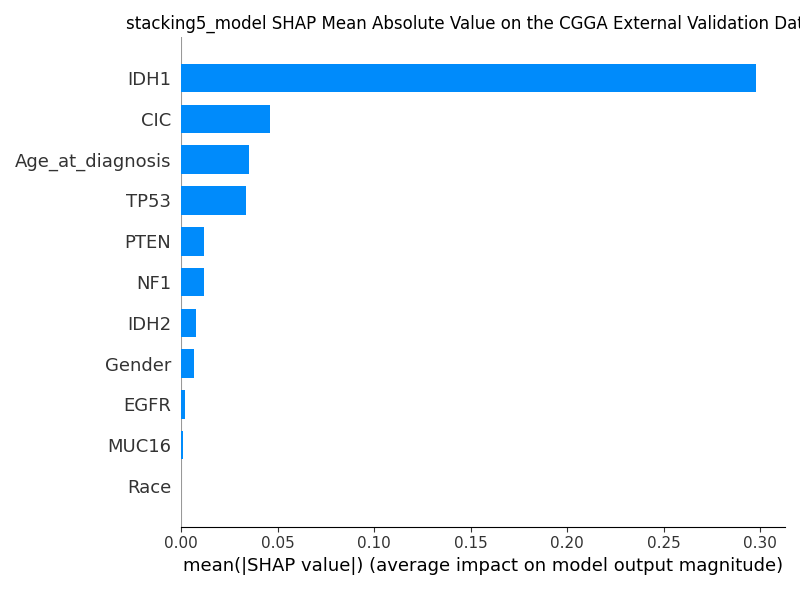

Supplement: S8 File — (ZIP) [file pone.0314831.s018.zip › S8 File/stacking5_model_feature_importance_bar.png]

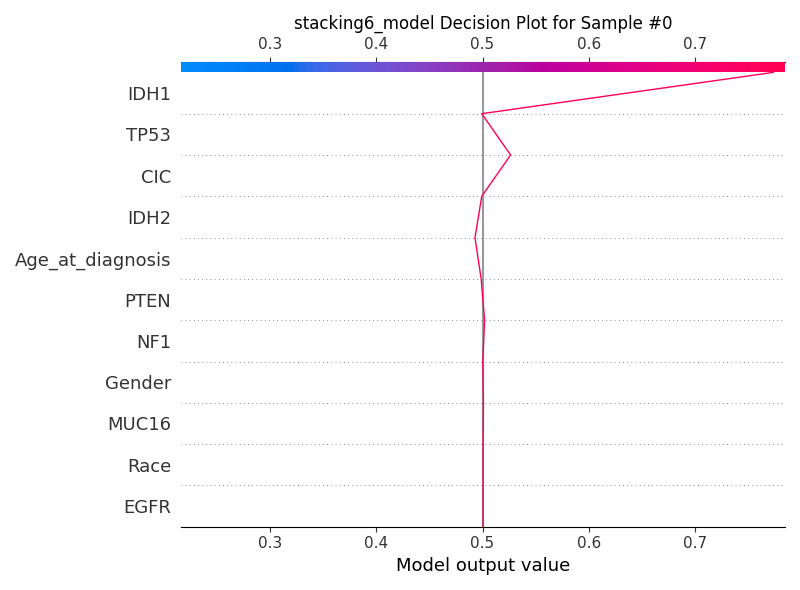

Supplement: S8 File — (ZIP) [file pone.0314831.s018.zip › S8 File/stacking6_model_decision_plot.png]

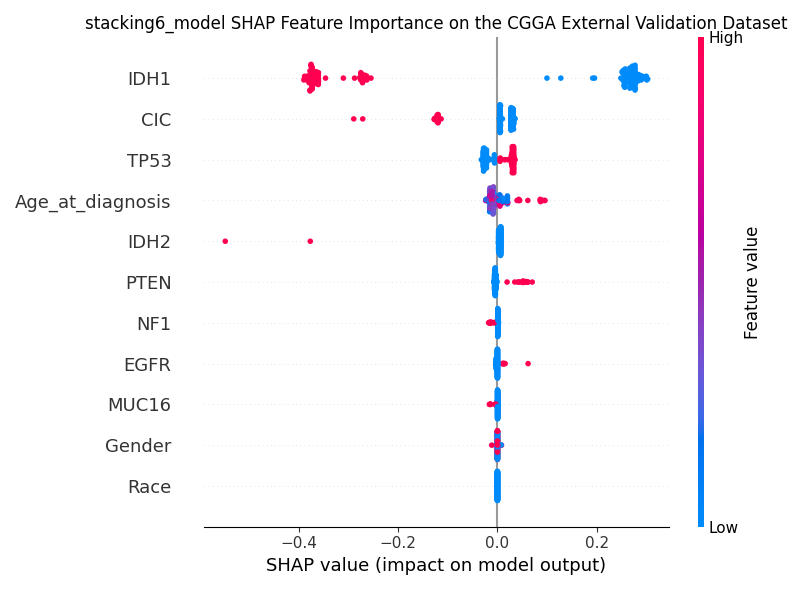

Supplement: S8 File — (ZIP) [file pone.0314831.s018.zip › S8 File/stacking6_model_feature_importance.png]

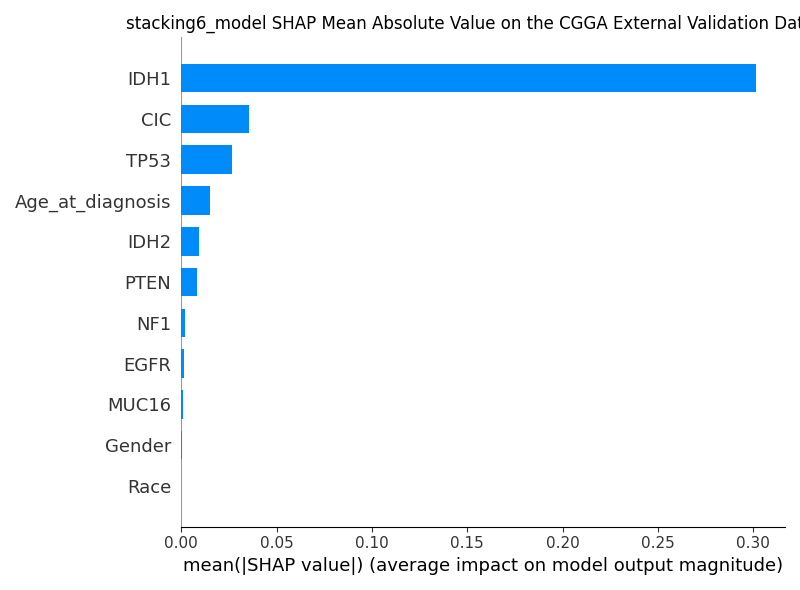

Supplement: S8 File — (ZIP) [file pone.0314831.s018.zip › S8 File/stacking6_model_feature_importance_bar.png]

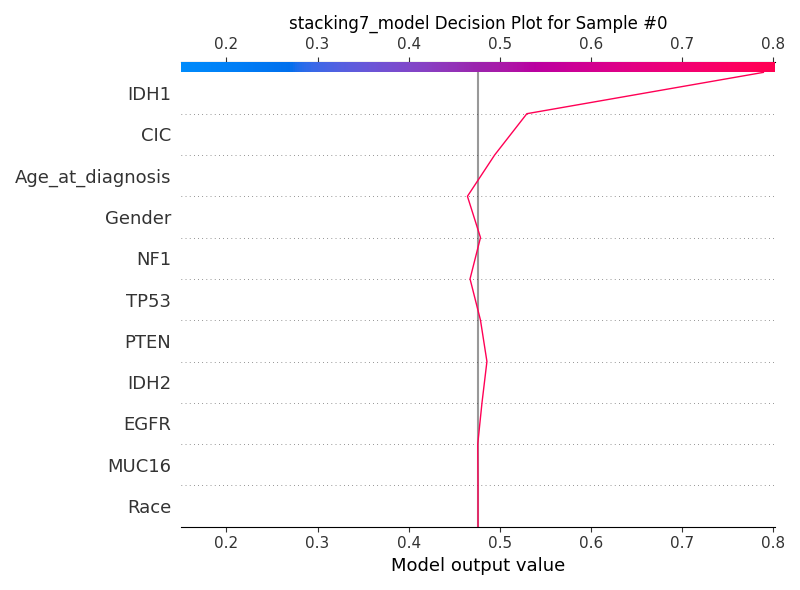

Supplement: S8 File — (ZIP) [file pone.0314831.s018.zip › S8 File/stacking7_model_decision_plot.png]

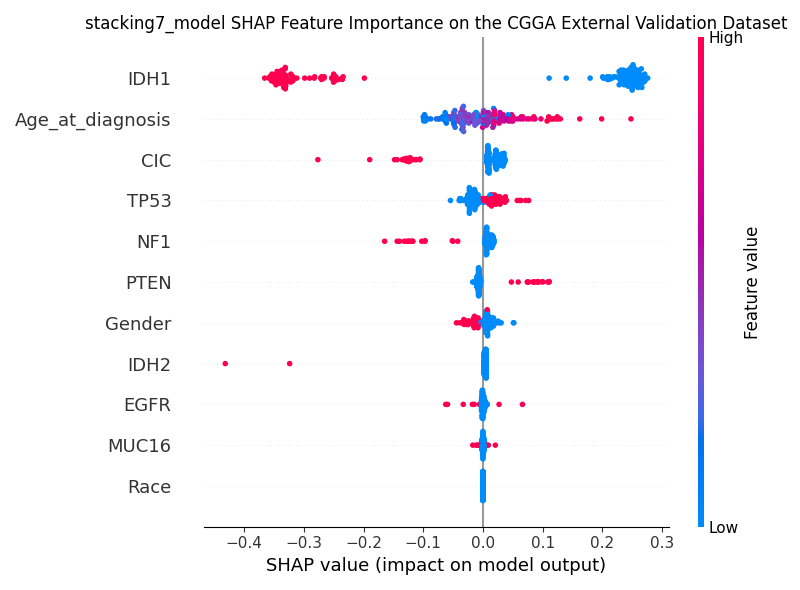

Supplement: S8 File — (ZIP) [file pone.0314831.s018.zip › S8 File/stacking7_model_feature_importance.png]

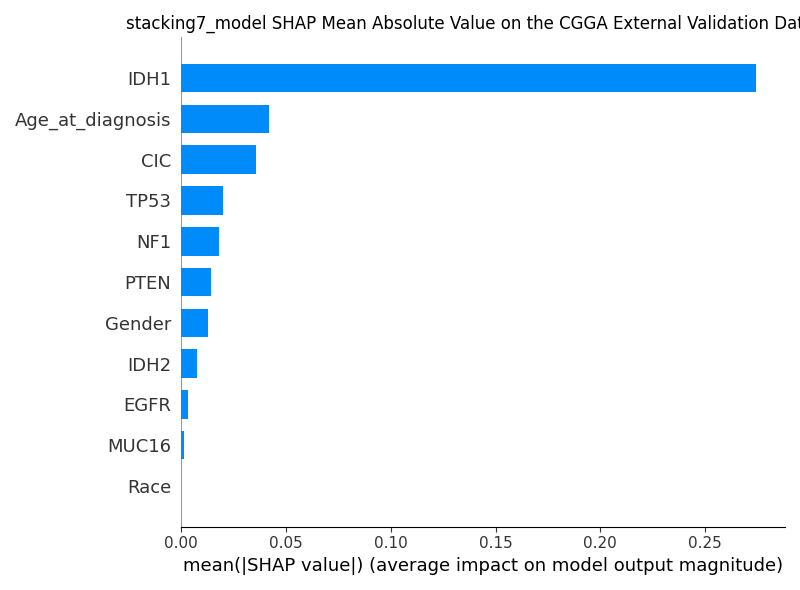

Supplement: S8 File — (ZIP) [file pone.0314831.s018.zip › S8 File/stacking7_model_feature_importance_bar.png]

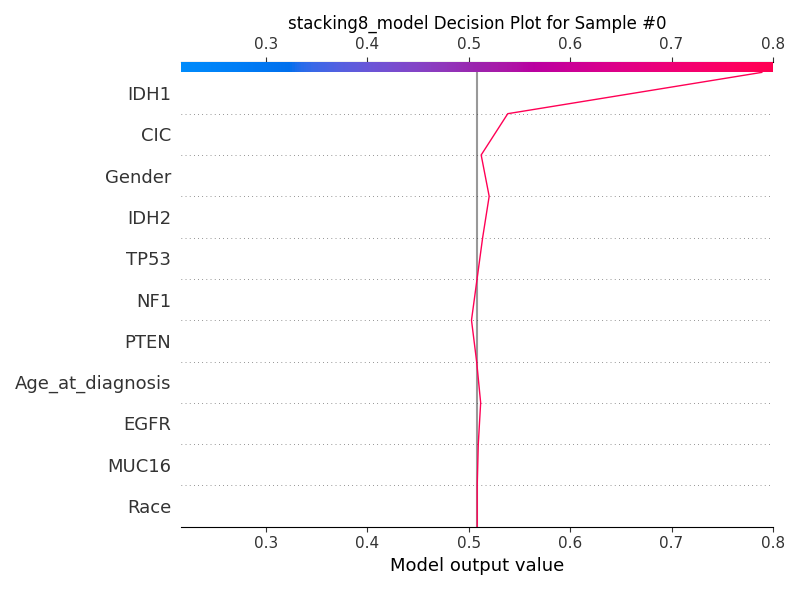

Supplement: S8 File — (ZIP) [file pone.0314831.s018.zip › S8 File/stacking8_model_decision_plot.png]

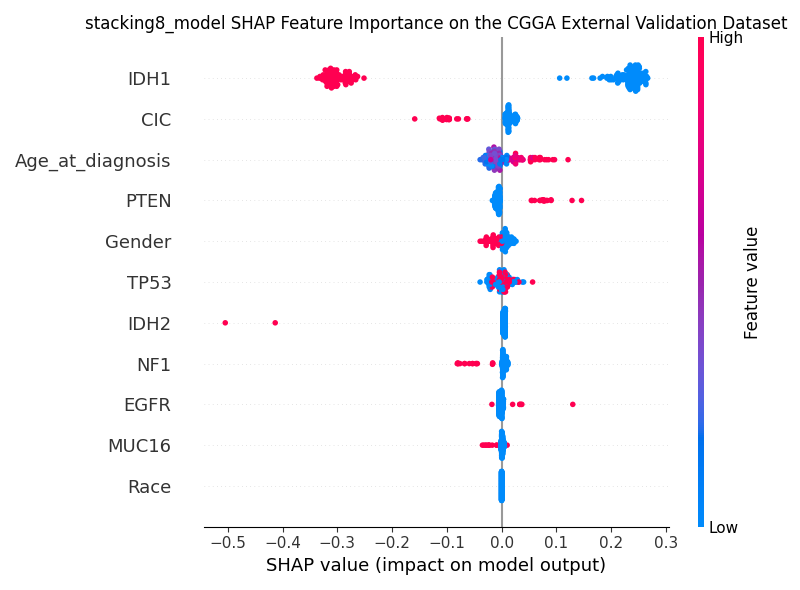

Supplement: S8 File — (ZIP) [file pone.0314831.s018.zip › S8 File/stacking8_model_feature_importance.png]

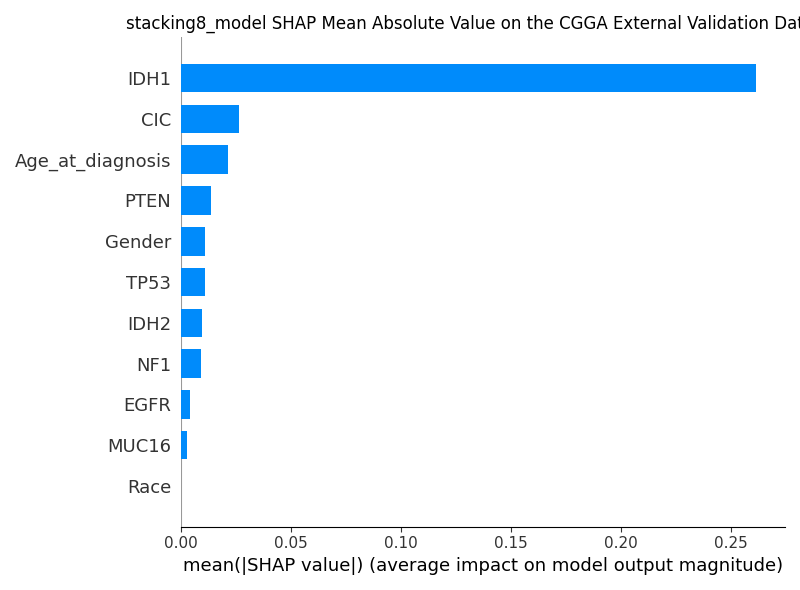

Supplement: S8 File — (ZIP) [file pone.0314831.s018.zip › S8 File/stacking8_model_feature_importance_bar.png]

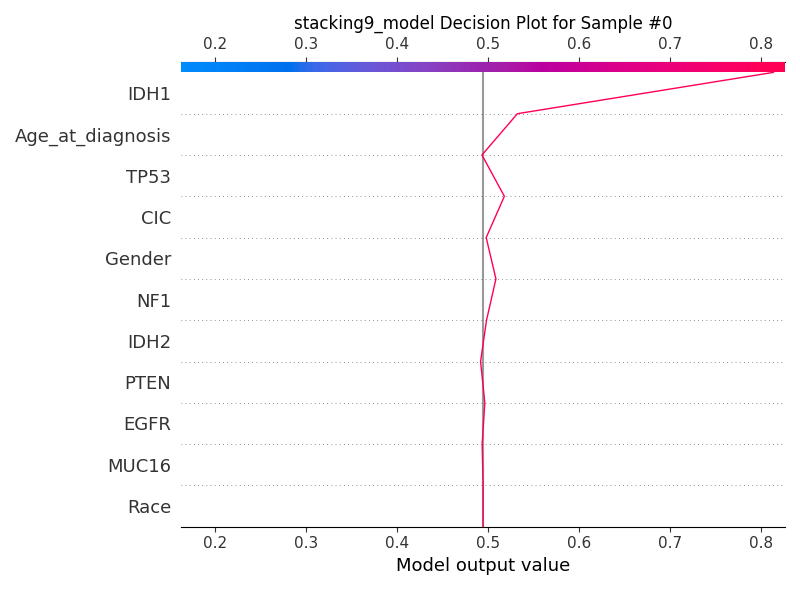

Supplement: S8 File — (ZIP) [file pone.0314831.s018.zip › S8 File/stacking9_model_decision_plot.png]
