# Supplementary figures and images for: Machine learning-based prediction of glioma grading (part 5 of 5)
Source: PLoS One. 2025 Dec 26;20(12):e0314831. doi: 10.1371/journal.pone.0314831 (PMC12742763; doi:10.1371/journal.pone.0314831)

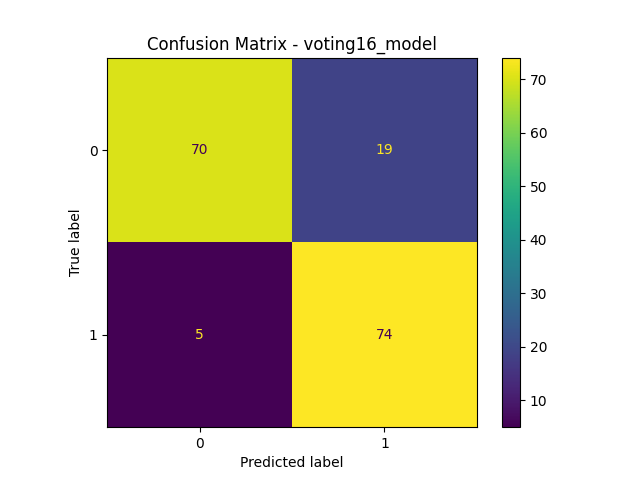

Supplement: S9 File — (ZIP) [file pone.0314831.s019.zip › S9 File/confusion_matrix_voting16_model.png]

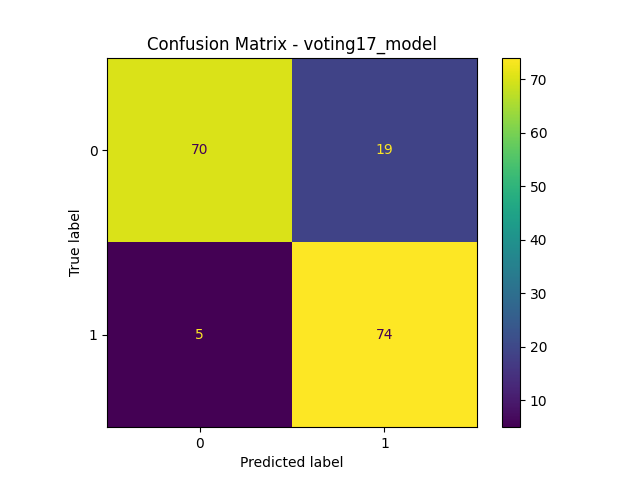

Supplement: S9 File — (ZIP) [file pone.0314831.s019.zip › S9 File/confusion_matrix_voting17_model.png]

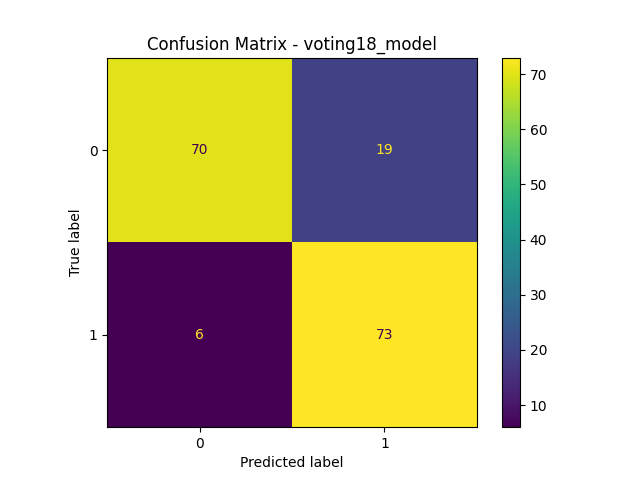

Supplement: S9 File — (ZIP) [file pone.0314831.s019.zip › S9 File/confusion_matrix_voting18_model.png]

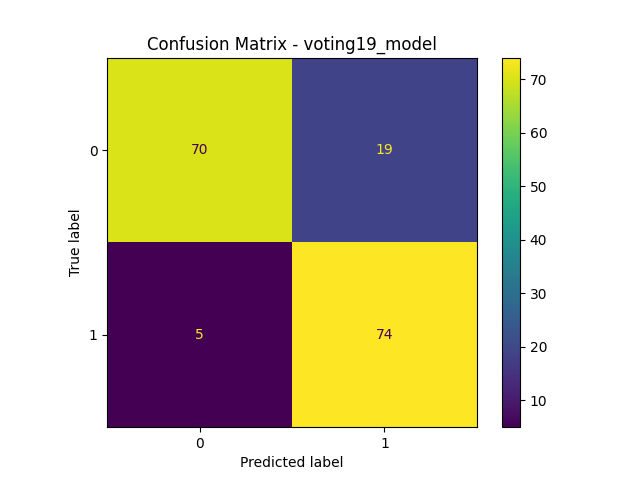

Supplement: S9 File — (ZIP) [file pone.0314831.s019.zip › S9 File/confusion_matrix_voting19_model.png]

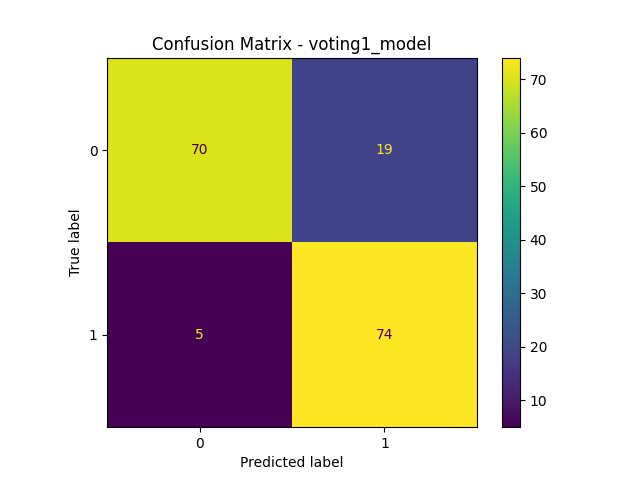

Supplement: S9 File — (ZIP) [file pone.0314831.s019.zip › S9 File/confusion_matrix_voting1_model.png]

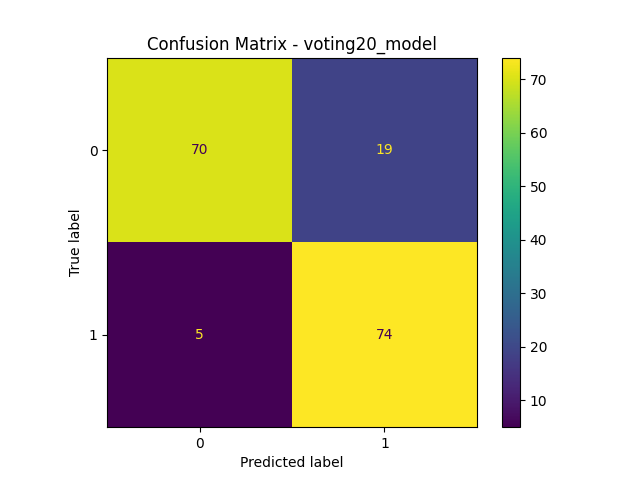

Supplement: S9 File — (ZIP) [file pone.0314831.s019.zip › S9 File/confusion_matrix_voting20_model.png]

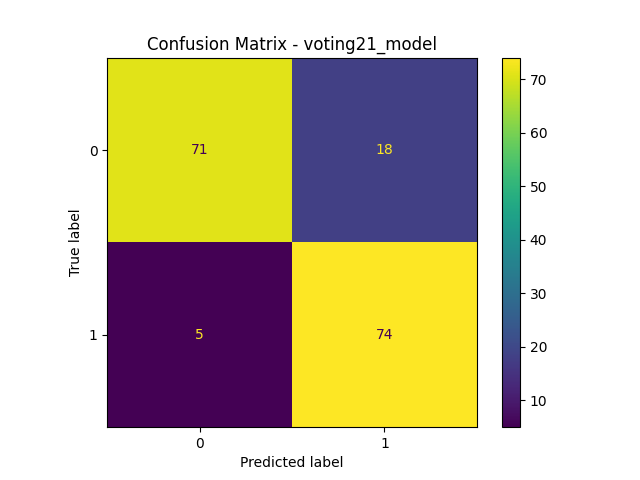

Supplement: S9 File — (ZIP) [file pone.0314831.s019.zip › S9 File/confusion_matrix_voting21_model.png]

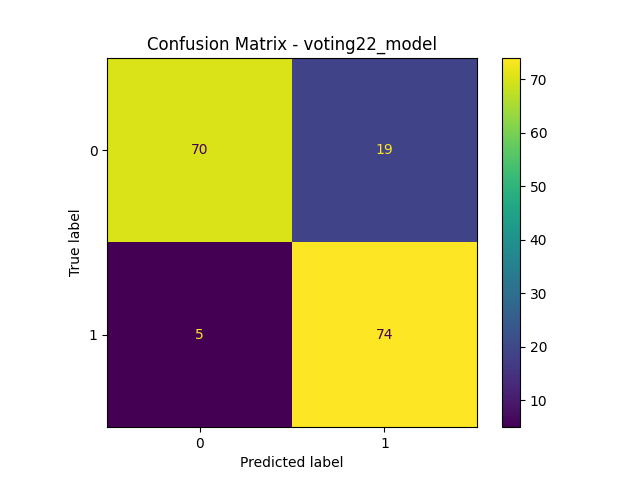

Supplement: S9 File — (ZIP) [file pone.0314831.s019.zip › S9 File/confusion_matrix_voting22_model.png]

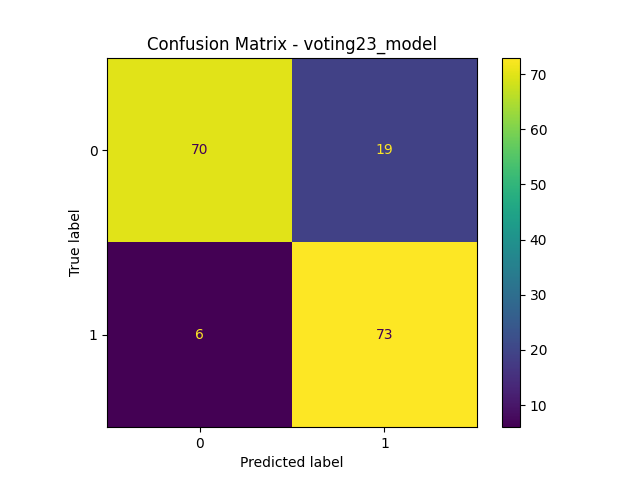

Supplement: S9 File — (ZIP) [file pone.0314831.s019.zip › S9 File/confusion_matrix_voting23_model.png]

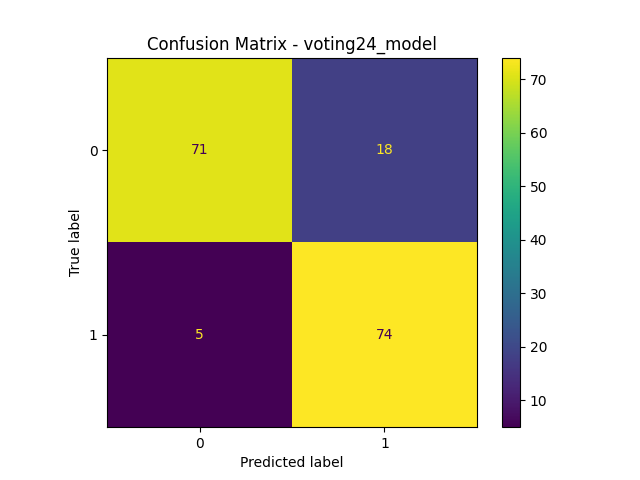

Supplement: S9 File — (ZIP) [file pone.0314831.s019.zip › S9 File/confusion_matrix_voting24_model.png]

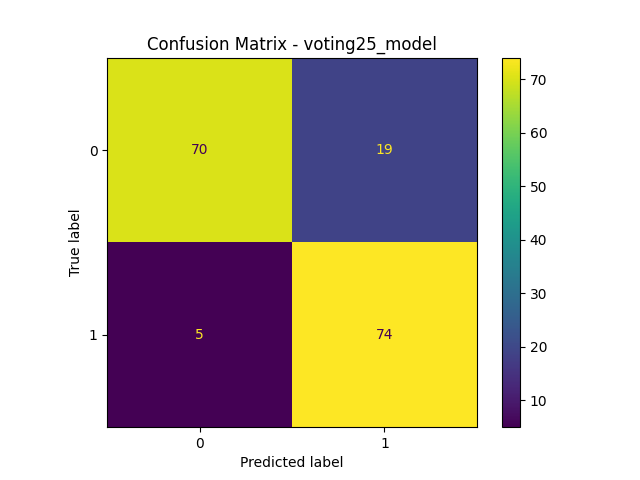

Supplement: S9 File — (ZIP) [file pone.0314831.s019.zip › S9 File/confusion_matrix_voting25_model.png]

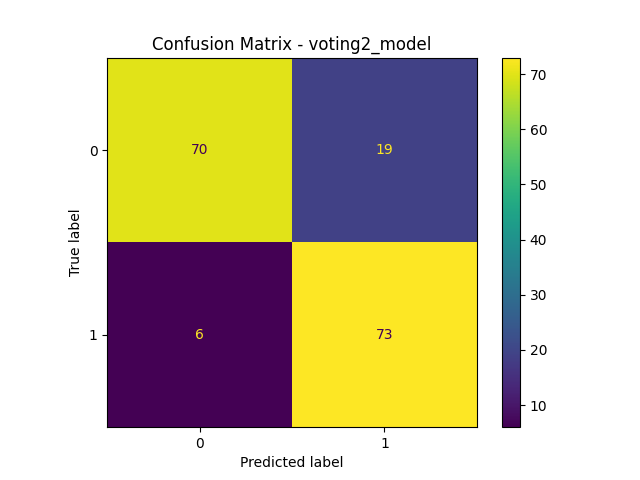

Supplement: S9 File — (ZIP) [file pone.0314831.s019.zip › S9 File/confusion_matrix_voting2_model.png]

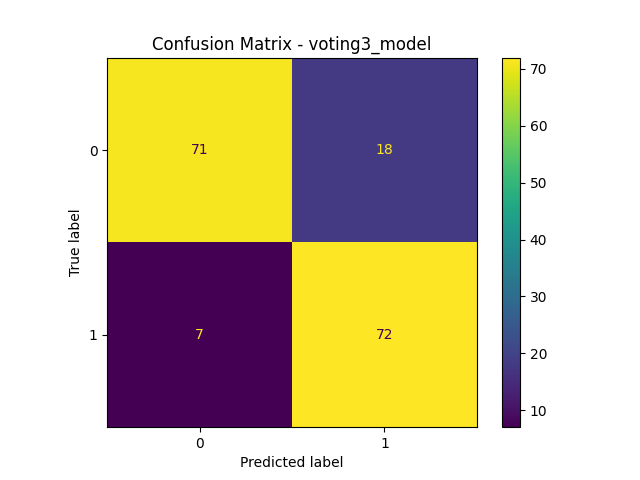

Supplement: S9 File — (ZIP) [file pone.0314831.s019.zip › S9 File/confusion_matrix_voting3_model.png]

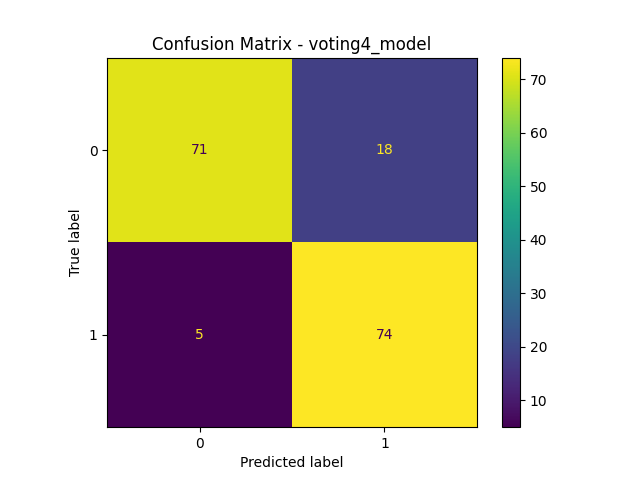

Supplement: S9 File — (ZIP) [file pone.0314831.s019.zip › S9 File/confusion_matrix_voting4_model.png]

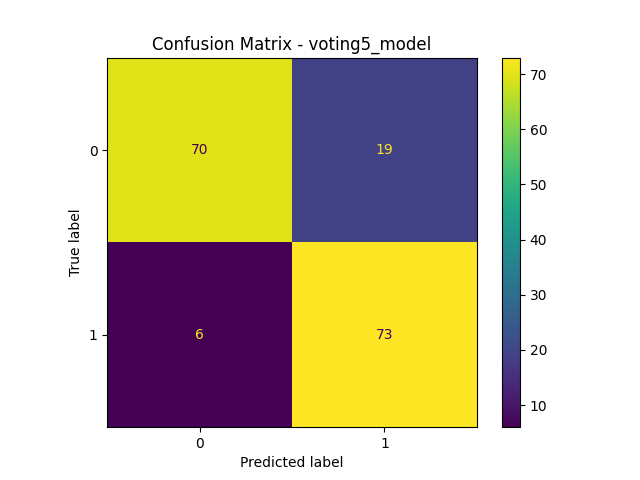

Supplement: S9 File — (ZIP) [file pone.0314831.s019.zip › S9 File/confusion_matrix_voting5_model.png]

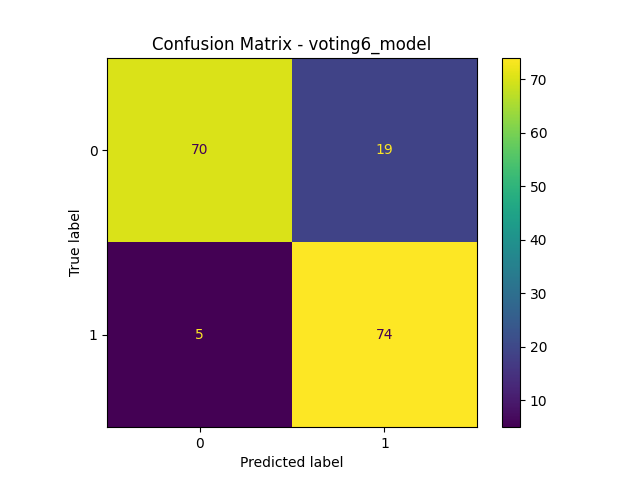

Supplement: S9 File — (ZIP) [file pone.0314831.s019.zip › S9 File/confusion_matrix_voting6_model.png]

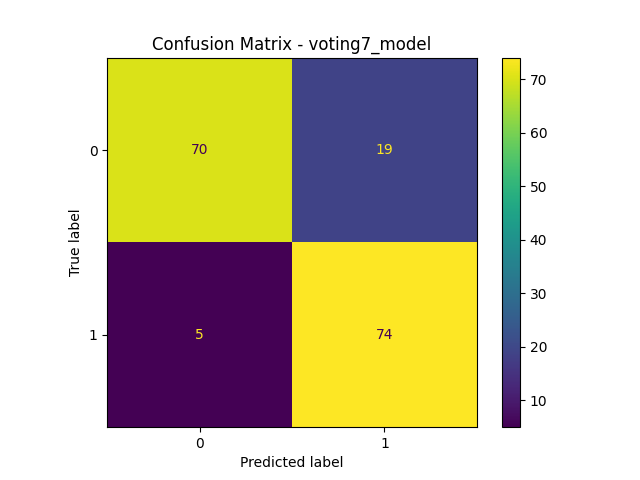

Supplement: S9 File — (ZIP) [file pone.0314831.s019.zip › S9 File/confusion_matrix_voting7_model.png]

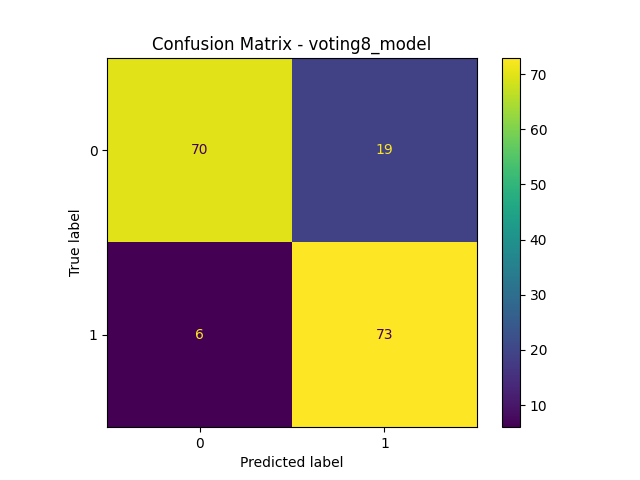

Supplement: S9 File — (ZIP) [file pone.0314831.s019.zip › S9 File/confusion_matrix_voting8_model.png]

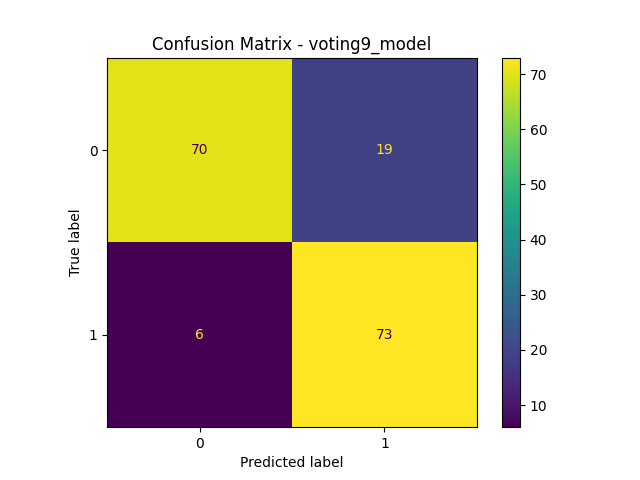

Supplement: S9 File — (ZIP) [file pone.0314831.s019.zip › S9 File/confusion_matrix_voting9_model.png]

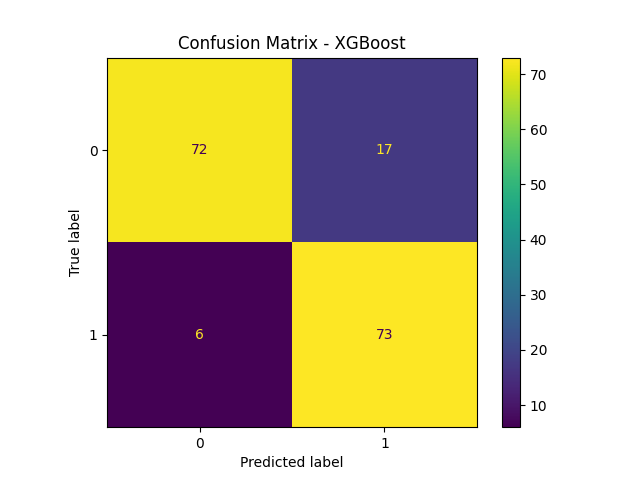

Supplement: S9 File — (ZIP) [file pone.0314831.s019.zip › S9 File/confusion_matrix_XGBoost.png]

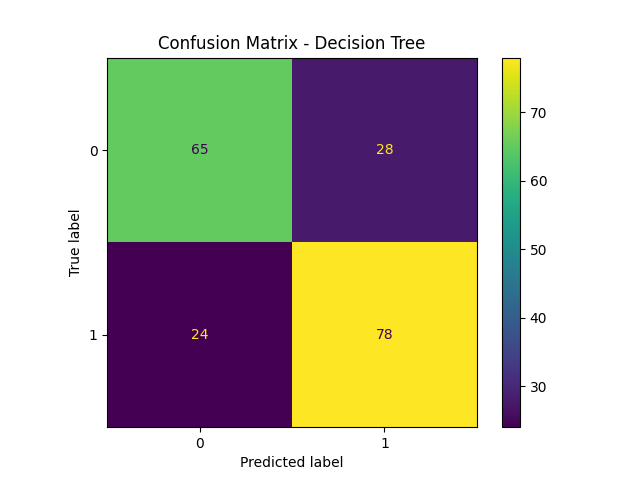

Supplement: S10 File — (ZIP) [file pone.0314831.s020.zip › S10 File/confusion_matrix_Decision Tree.png]

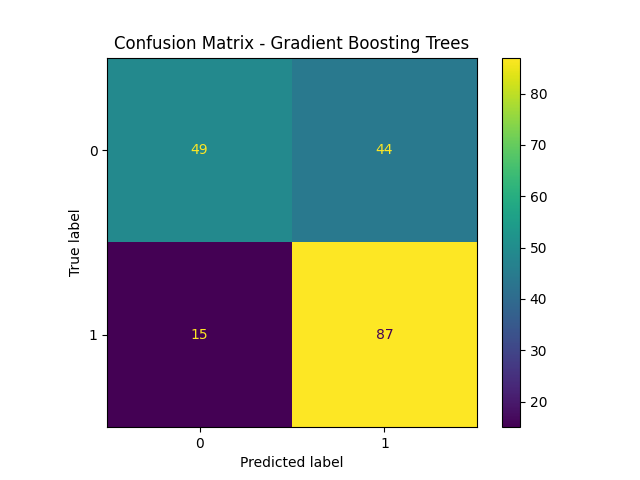

Supplement: S10 File — (ZIP) [file pone.0314831.s020.zip › S10 File/confusion_matrix_Gradient Boosting Trees.png]

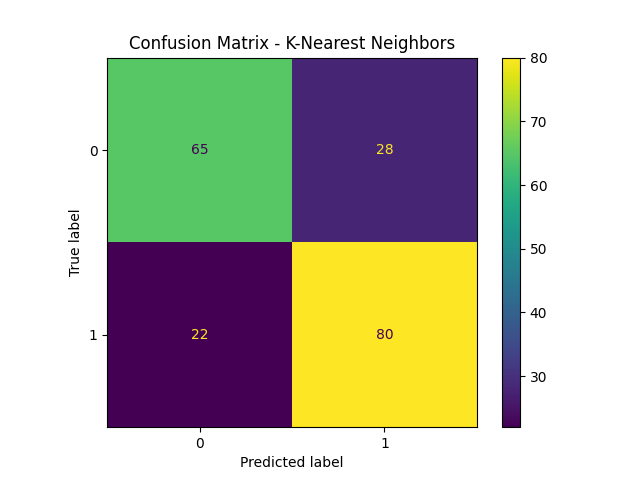

Supplement: S10 File — (ZIP) [file pone.0314831.s020.zip › S10 File/confusion_matrix_K-Nearest Neighbors.png]

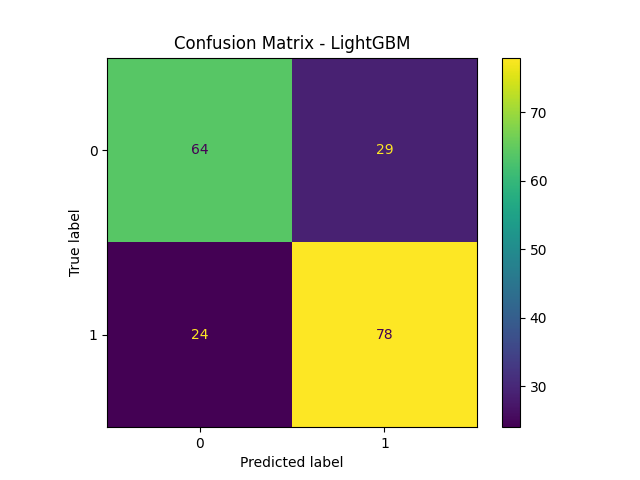

Supplement: S10 File — (ZIP) [file pone.0314831.s020.zip › S10 File/confusion_matrix_LightGBM.png]

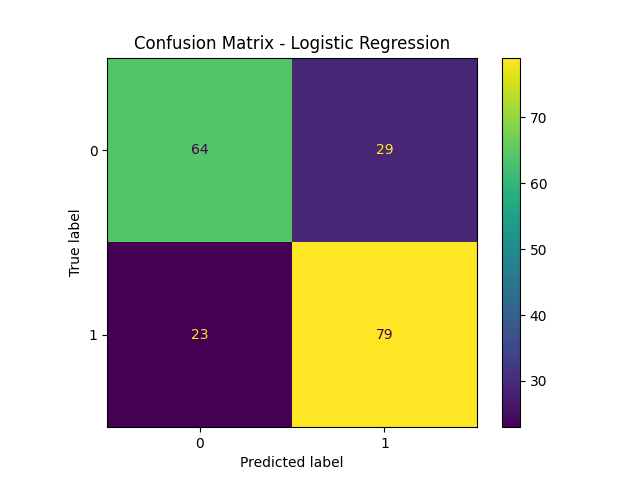

Supplement: S10 File — (ZIP) [file pone.0314831.s020.zip › S10 File/confusion_matrix_Logistic Regression.png]

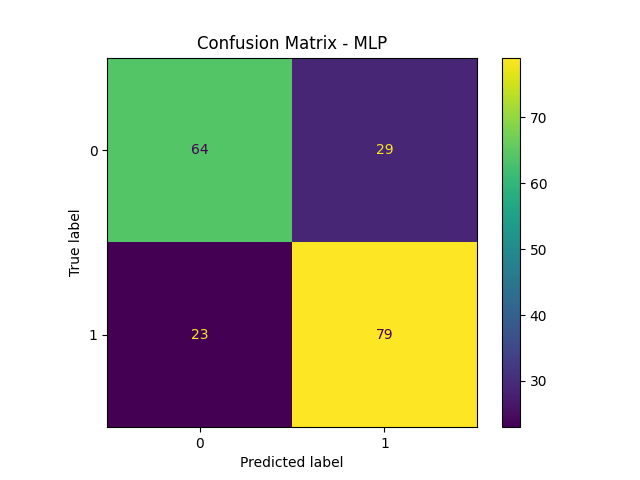

Supplement: S10 File — (ZIP) [file pone.0314831.s020.zip › S10 File/confusion_matrix_MLP.png]

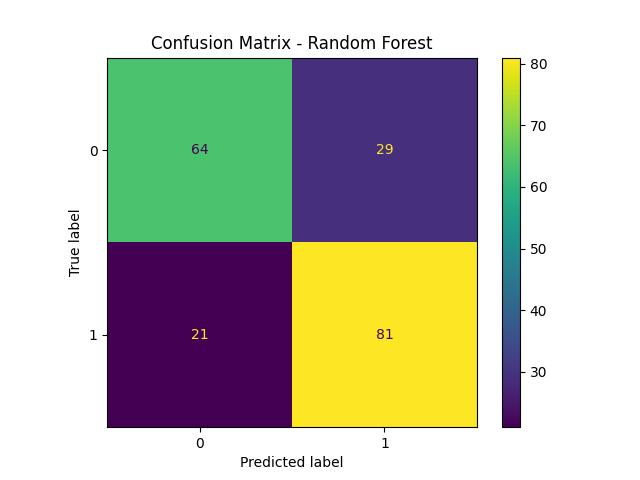

Supplement: S10 File — (ZIP) [file pone.0314831.s020.zip › S10 File/confusion_matrix_Random Forest.png]

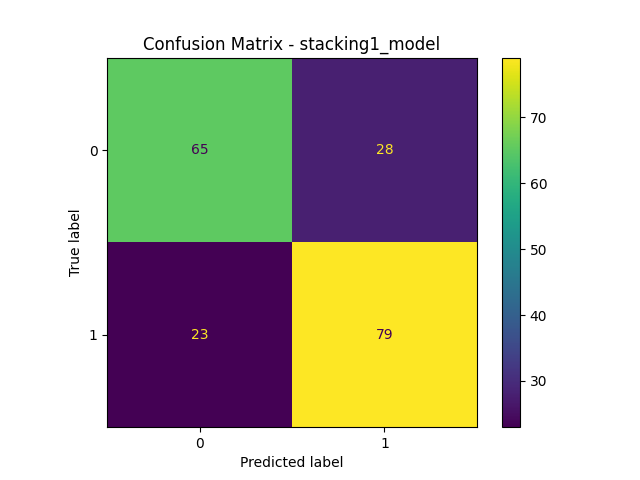

Supplement: S10 File — (ZIP) [file pone.0314831.s020.zip › S10 File/confusion_matrix_stacking1_model.png]

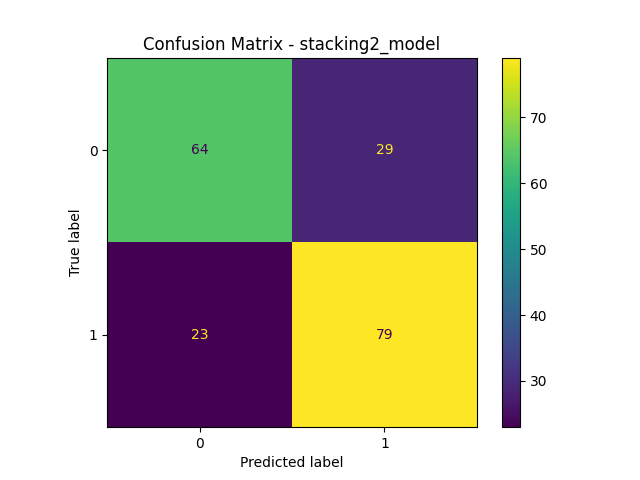

Supplement: S10 File — (ZIP) [file pone.0314831.s020.zip › S10 File/confusion_matrix_stacking2_model.png]

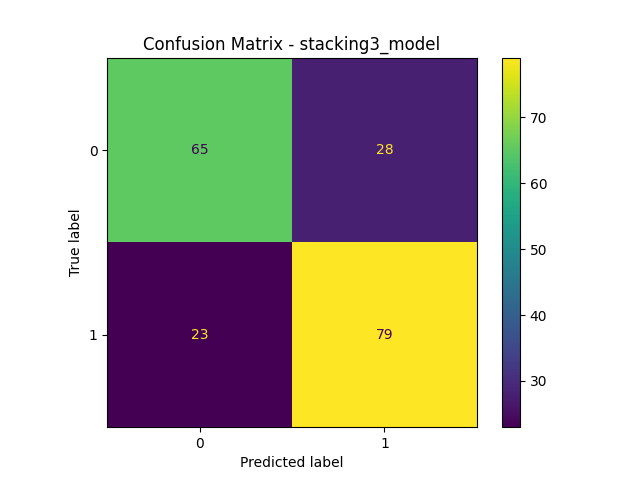

Supplement: S10 File — (ZIP) [file pone.0314831.s020.zip › S10 File/confusion_matrix_stacking3_model.png]

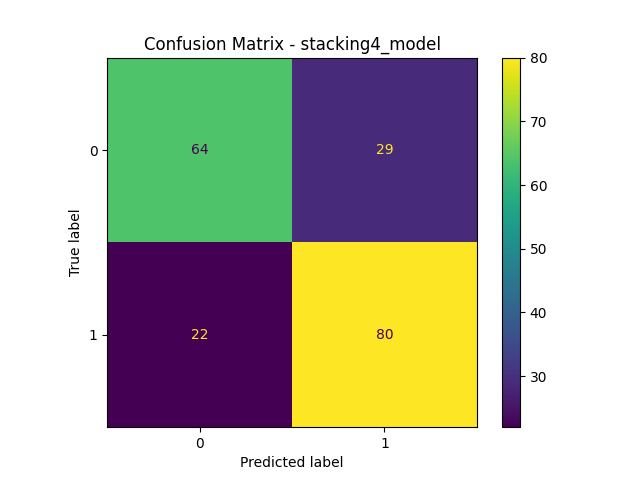

Supplement: S10 File — (ZIP) [file pone.0314831.s020.zip › S10 File/confusion_matrix_stacking4_model.png]

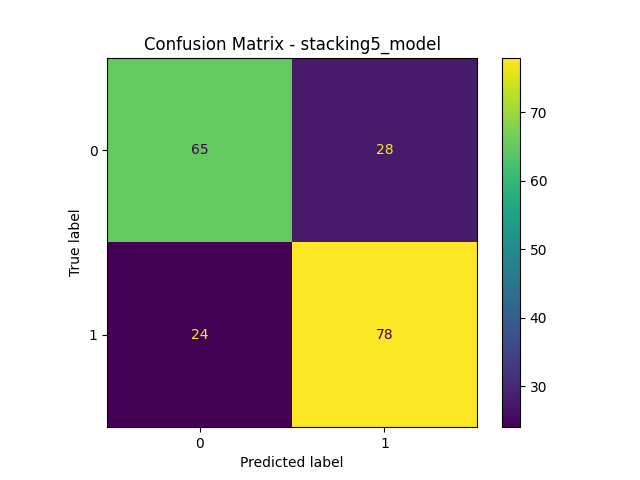

Supplement: S10 File — (ZIP) [file pone.0314831.s020.zip › S10 File/confusion_matrix_stacking5_model.png]

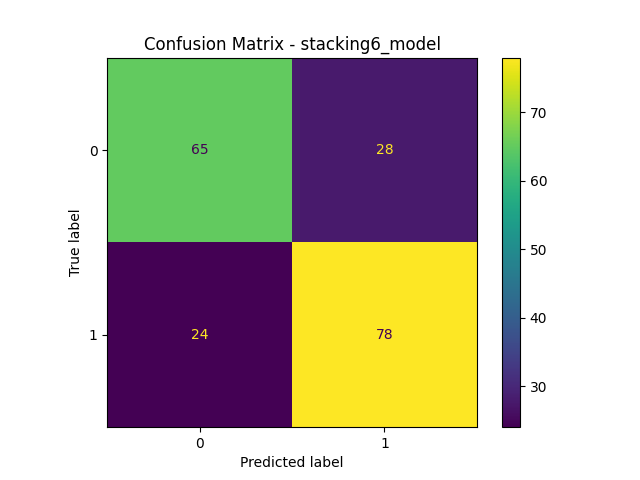

Supplement: S10 File — (ZIP) [file pone.0314831.s020.zip › S10 File/confusion_matrix_stacking6_model.png]

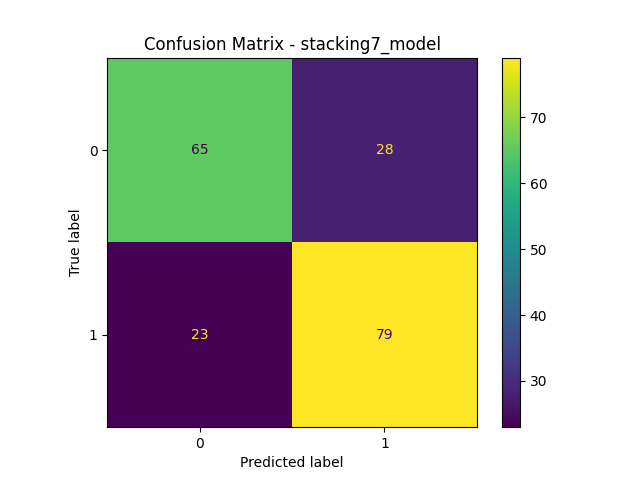

Supplement: S10 File — (ZIP) [file pone.0314831.s020.zip › S10 File/confusion_matrix_stacking7_model.png]

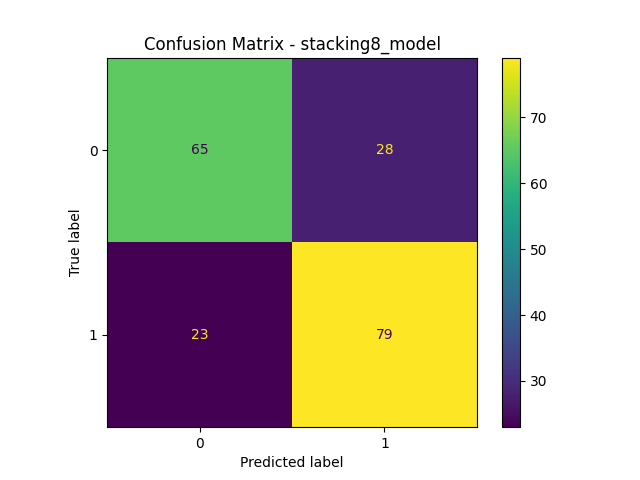

Supplement: S10 File — (ZIP) [file pone.0314831.s020.zip › S10 File/confusion_matrix_stacking8_model.png]

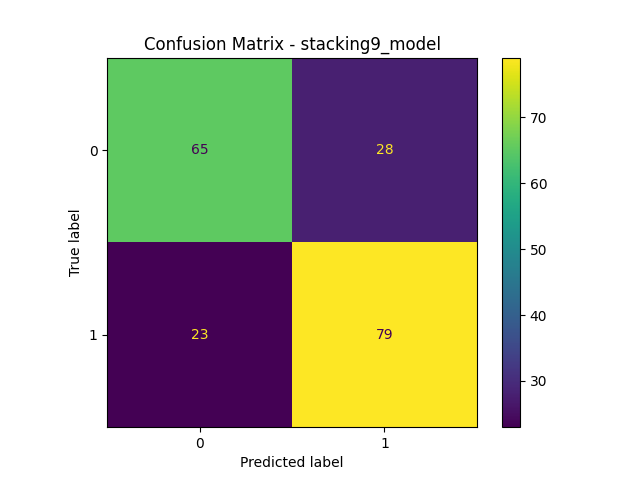

Supplement: S10 File — (ZIP) [file pone.0314831.s020.zip › S10 File/confusion_matrix_stacking9_model.png]

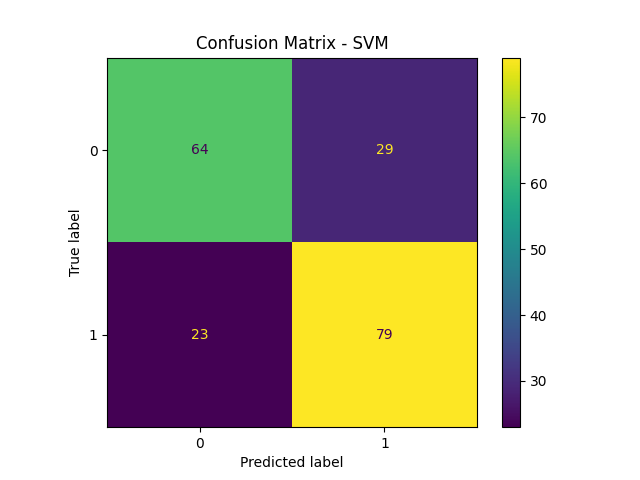

Supplement: S10 File — (ZIP) [file pone.0314831.s020.zip › S10 File/confusion_matrix_SVM.png]

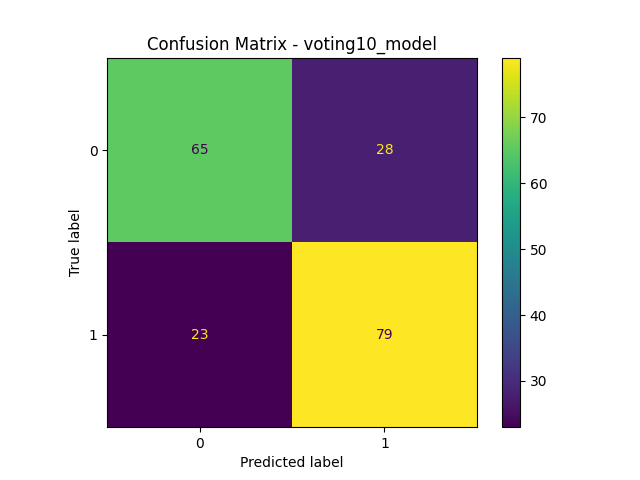

Supplement: S10 File — (ZIP) [file pone.0314831.s020.zip › S10 File/confusion_matrix_voting10_model.png]

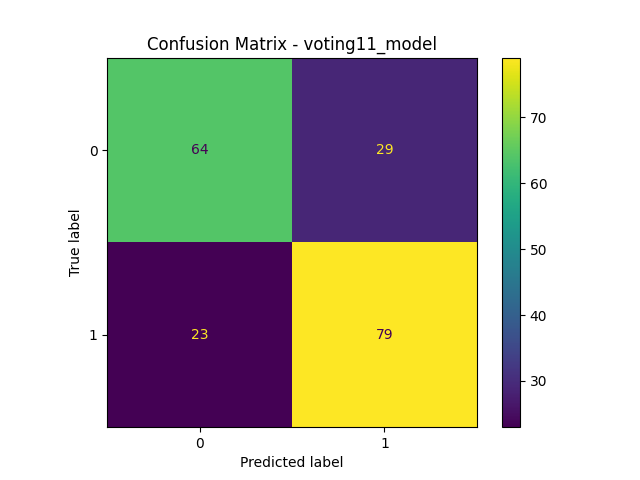

Supplement: S10 File — (ZIP) [file pone.0314831.s020.zip › S10 File/confusion_matrix_voting11_model.png]

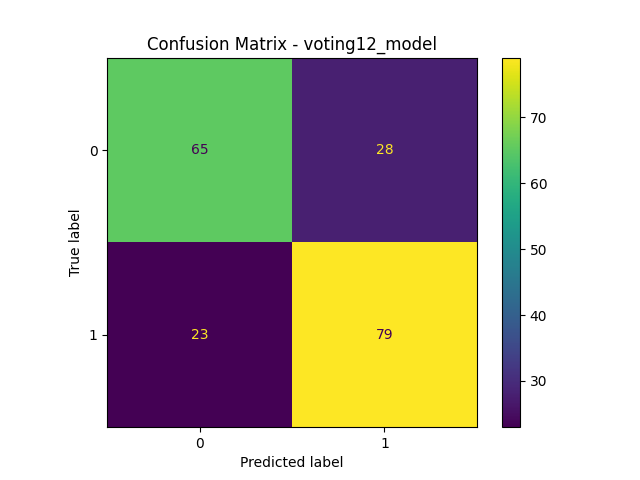

Supplement: S10 File — (ZIP) [file pone.0314831.s020.zip › S10 File/confusion_matrix_voting12_model.png]

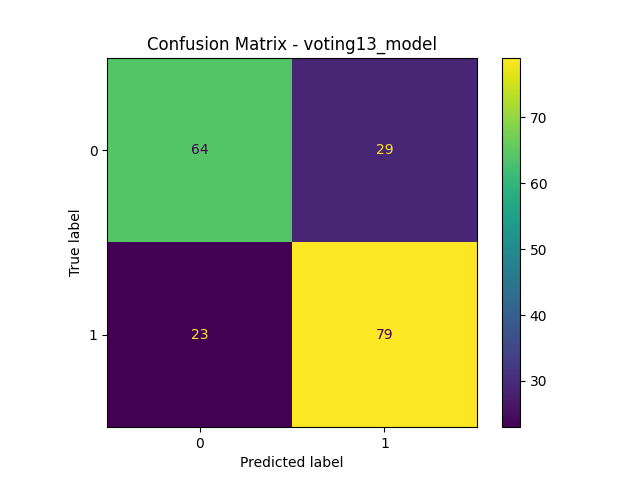

Supplement: S10 File — (ZIP) [file pone.0314831.s020.zip › S10 File/confusion_matrix_voting13_model.png]

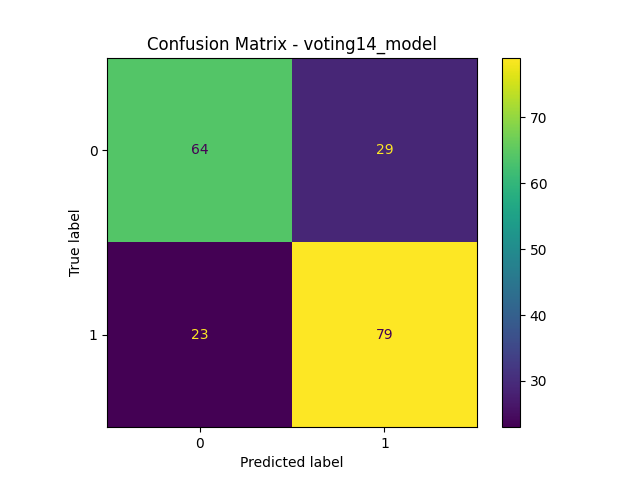

Supplement: S10 File — (ZIP) [file pone.0314831.s020.zip › S10 File/confusion_matrix_voting14_model.png]

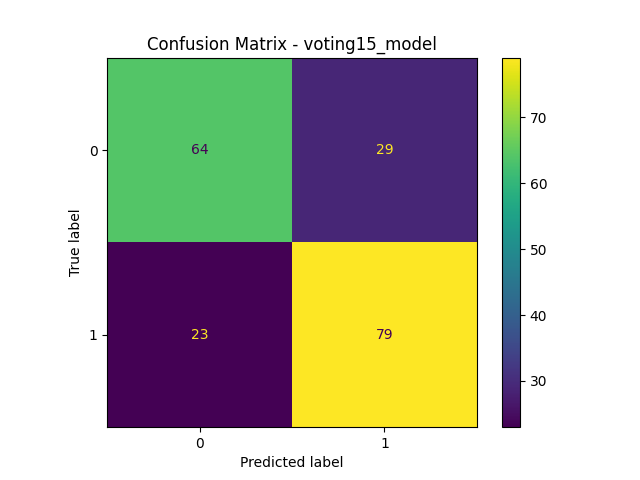

Supplement: S10 File — (ZIP) [file pone.0314831.s020.zip › S10 File/confusion_matrix_voting15_model.png]

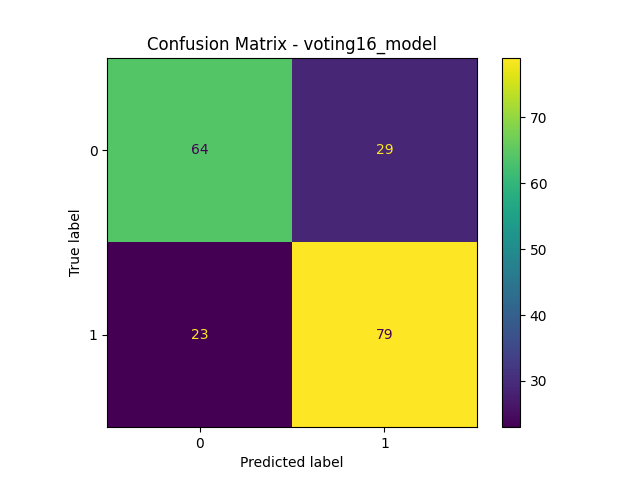

Supplement: S10 File — (ZIP) [file pone.0314831.s020.zip › S10 File/confusion_matrix_voting16_model.png]

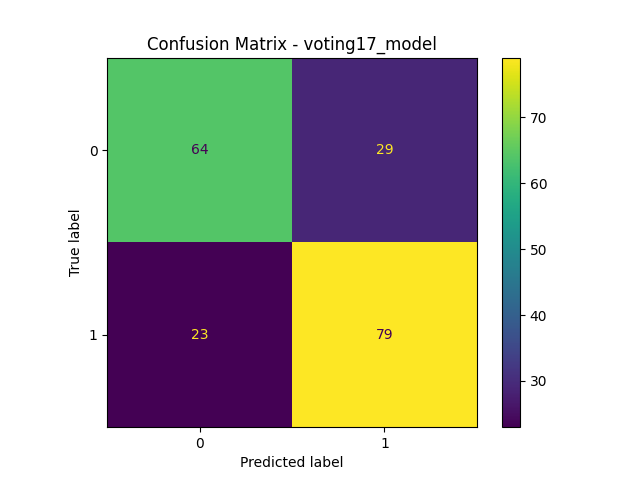

Supplement: S10 File — (ZIP) [file pone.0314831.s020.zip › S10 File/confusion_matrix_voting17_model.png]

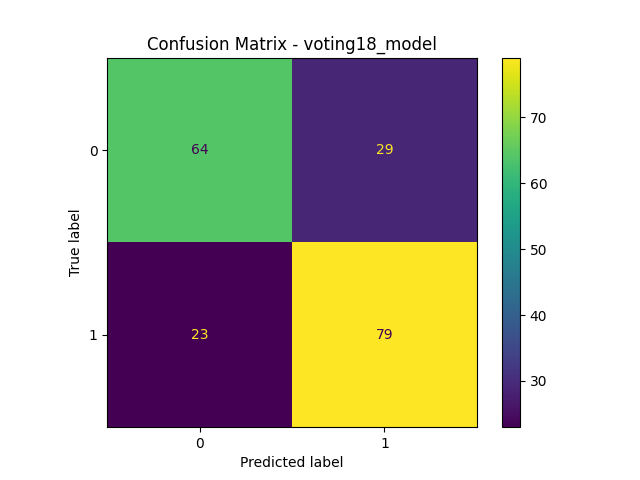

Supplement: S10 File — (ZIP) [file pone.0314831.s020.zip › S10 File/confusion_matrix_voting18_model.png]

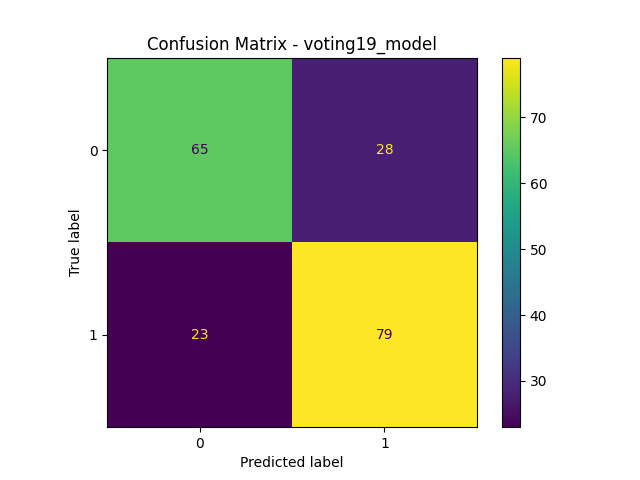

Supplement: S10 File — (ZIP) [file pone.0314831.s020.zip › S10 File/confusion_matrix_voting19_model.png]

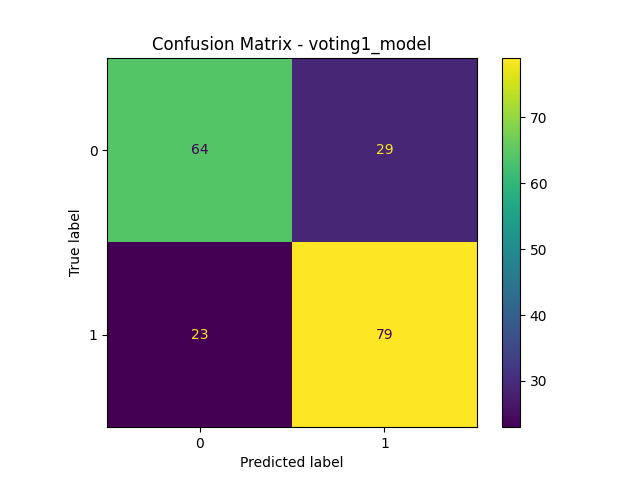

Supplement: S10 File — (ZIP) [file pone.0314831.s020.zip › S10 File/confusion_matrix_voting1_model.png]

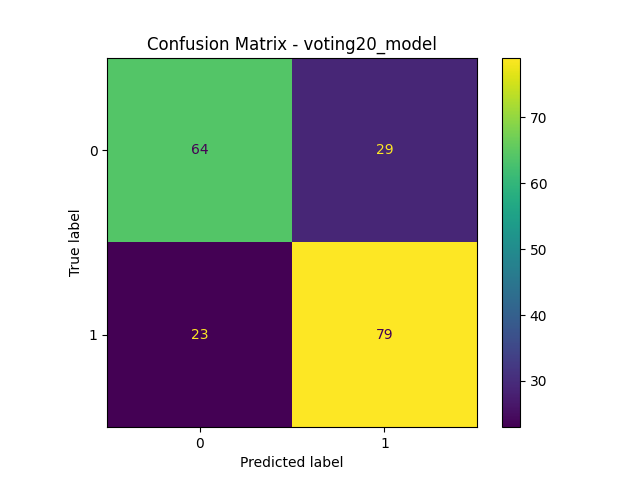

Supplement: S10 File — (ZIP) [file pone.0314831.s020.zip › S10 File/confusion_matrix_voting20_model.png]

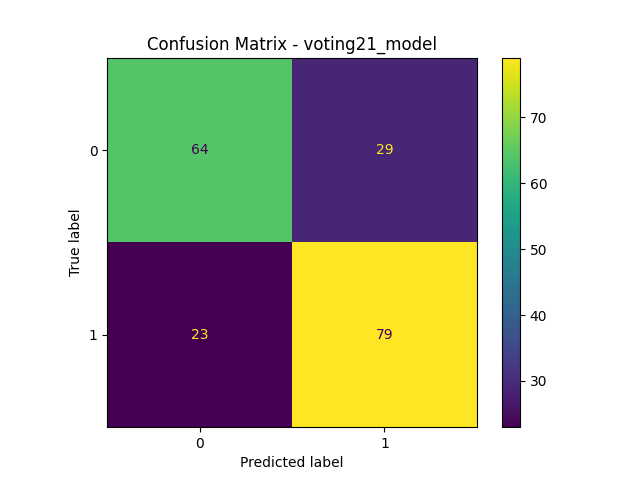

Supplement: S10 File — (ZIP) [file pone.0314831.s020.zip › S10 File/confusion_matrix_voting21_model.png]

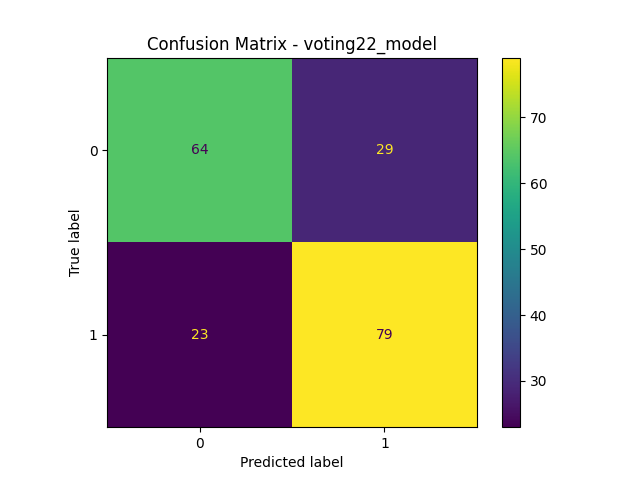

Supplement: S10 File — (ZIP) [file pone.0314831.s020.zip › S10 File/confusion_matrix_voting22_model.png]

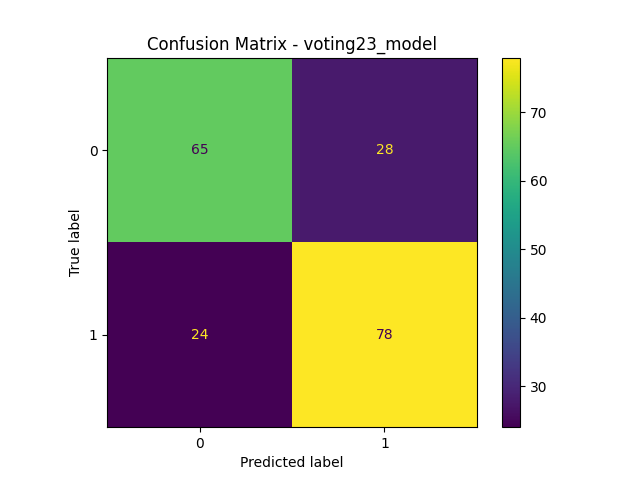

Supplement: S10 File — (ZIP) [file pone.0314831.s020.zip › S10 File/confusion_matrix_voting23_model.png]

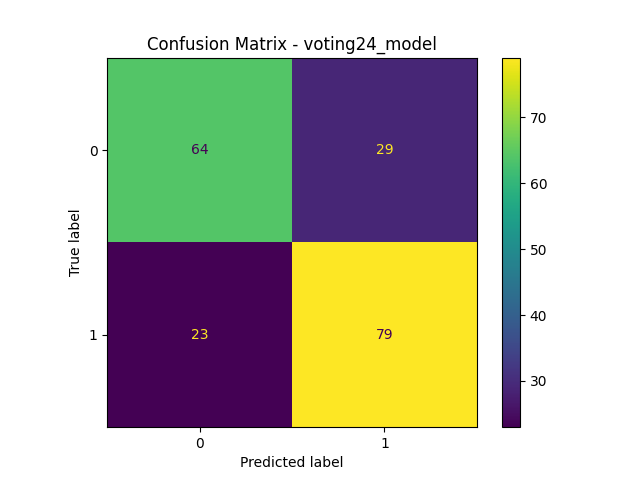

Supplement: S10 File — (ZIP) [file pone.0314831.s020.zip › S10 File/confusion_matrix_voting24_model.png]

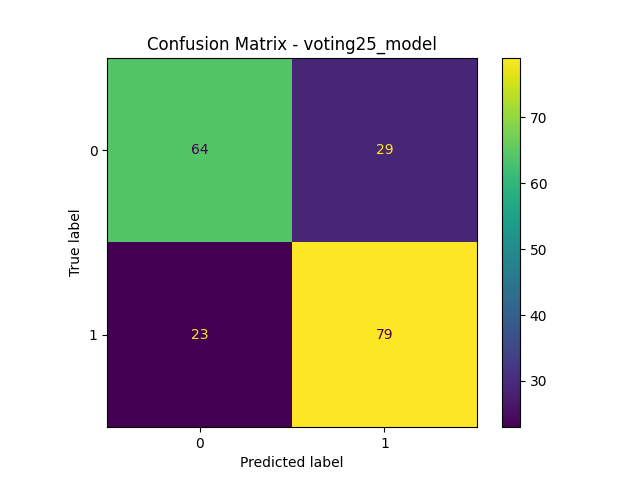

Supplement: S10 File — (ZIP) [file pone.0314831.s020.zip › S10 File/confusion_matrix_voting25_model.png]

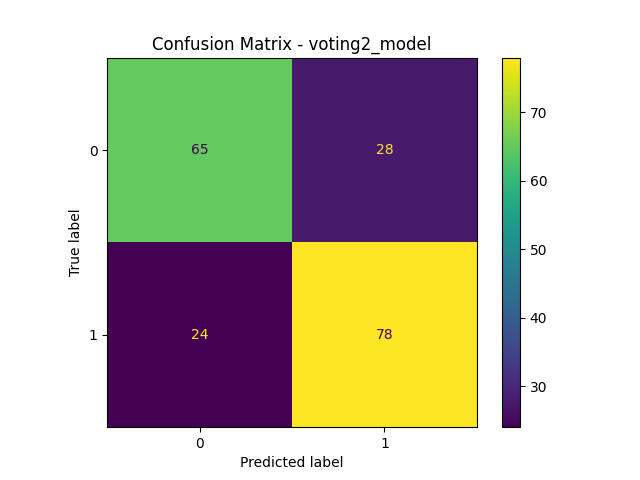

Supplement: S10 File — (ZIP) [file pone.0314831.s020.zip › S10 File/confusion_matrix_voting2_model.png]

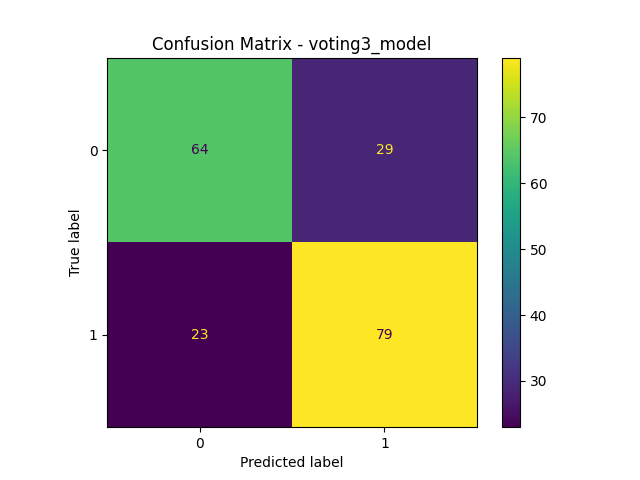

Supplement: S10 File — (ZIP) [file pone.0314831.s020.zip › S10 File/confusion_matrix_voting3_model.png]

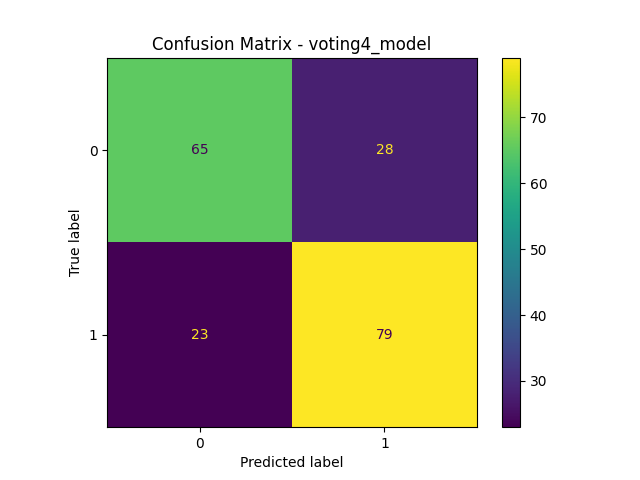

Supplement: S10 File — (ZIP) [file pone.0314831.s020.zip › S10 File/confusion_matrix_voting4_model.png]

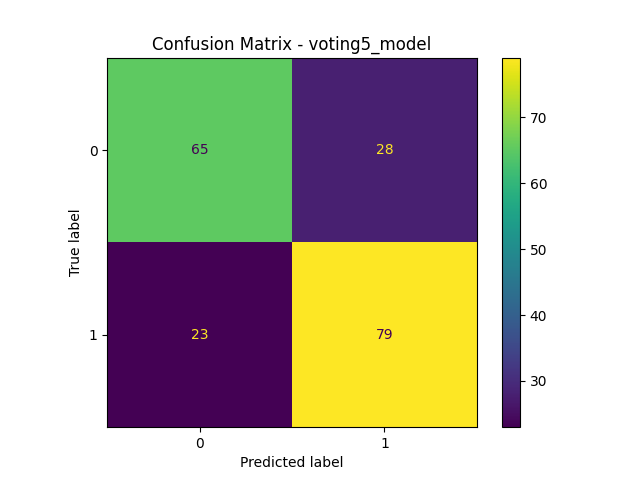

Supplement: S10 File — (ZIP) [file pone.0314831.s020.zip › S10 File/confusion_matrix_voting5_model.png]

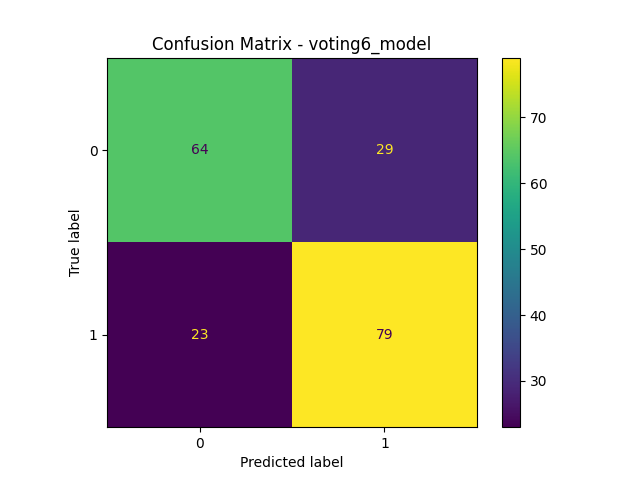

Supplement: S10 File — (ZIP) [file pone.0314831.s020.zip › S10 File/confusion_matrix_voting6_model.png]

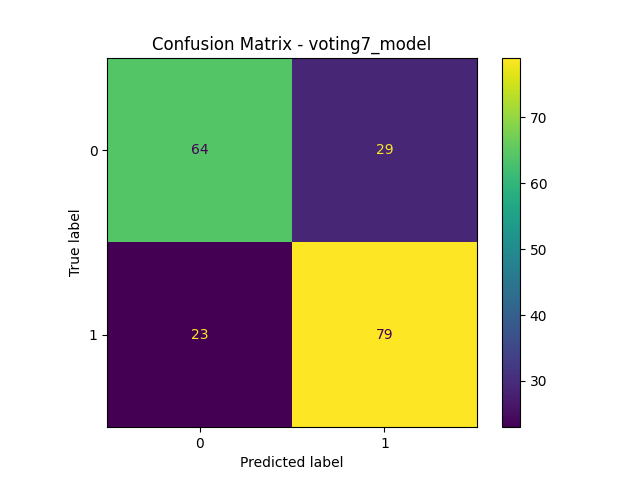

Supplement: S10 File — (ZIP) [file pone.0314831.s020.zip › S10 File/confusion_matrix_voting7_model.png]

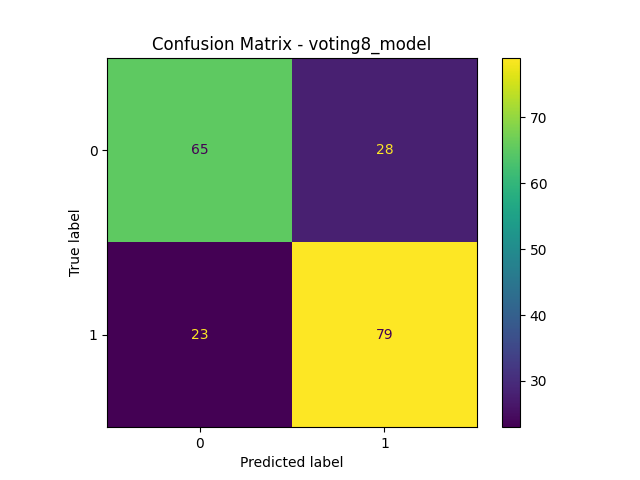

Supplement: S10 File — (ZIP) [file pone.0314831.s020.zip › S10 File/confusion_matrix_voting8_model.png]

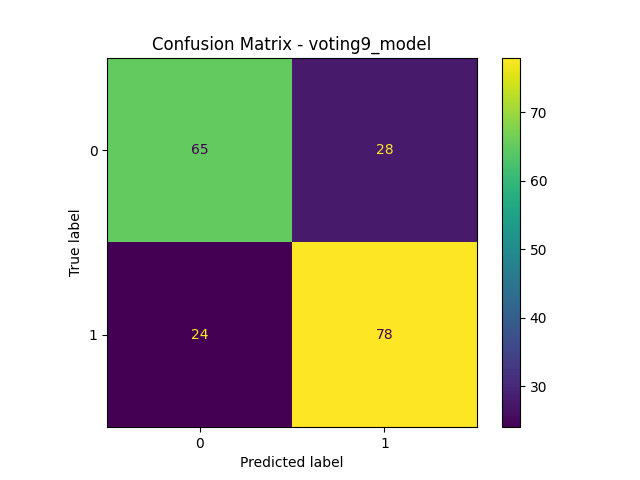

Supplement: S10 File — (ZIP) [file pone.0314831.s020.zip › S10 File/confusion_matrix_voting9_model.png]

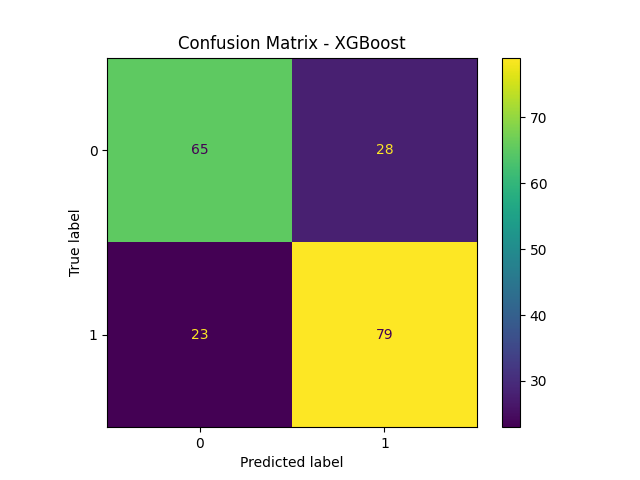

Supplement: S10 File — (ZIP) [file pone.0314831.s020.zip › S10 File/confusion_matrix_XGBoost.png]
